# Visible-Light-Mediated Rose Bengal- or [Ru(bpy)_3_]^2+^-Catalyzed Radical [4 + 2] Cycloaddition: An Efficient Route to Tetrahydrocarbazoles

**DOI:** 10.1021/acsomega.5c00416

**Published:** 2025-03-07

**Authors:** Cody Bishir, Abbey Hubbard, Liangyong Mei

**Affiliations:** Department of Chemistry and Biochemistry, University of North Florida, Jacksonville, Florida 32224, United States

## Abstract

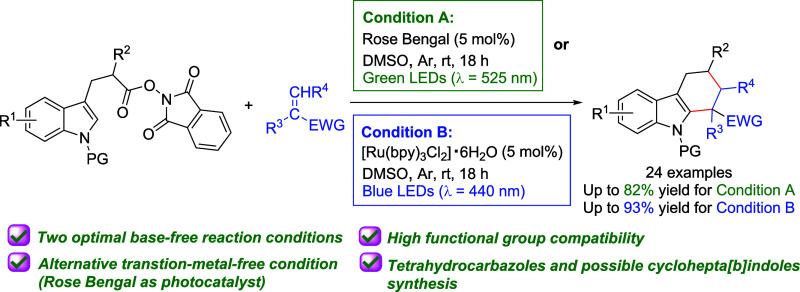

A visible-light-induced Rose Bengal- or [Ru(bpy)_3_]^2+^-catalyzed radical [4 + 2] cycloaddition of redox-active indole *N*-hydroxyphthalimide esters with electron-deficient alkenes has been developed. This base-free protocol provides a facile and powerful route for the synthesis of functionalized and biologically significant tetrahydrocarbazoles under mild conditions. On one hand, when an organic photocatalyst—Rose Bengal was employed under green light, the desired tetrahydrocarbazoles were obtained in up to 82% yield. On the other hand, the reaction yield increased to up to 93% in the presence of [Ru(bpy)_3_Cl_2_]·6H_2_O under blue light. The success of the gram-scale and transformation experiments, as well as the photopromoted radical [5 + 2] cycloaddition further highlight the practicality and robustness of this protocol. Mechanistic studies also support the generation of a crucial alkyl radical intermediate.

## Introduction

**Figure 1:**
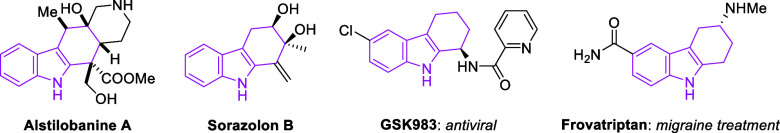
Natural alkaloids and pharmaceutical compounds with a tetracarbazole core structure.

Tetrahydrocarbazole scaffold widely exists in a range of naturally occurring alkaloids (e.g., Alstilobanine A and Sorazolon B) and pharmaceutical compounds (Figure 1),^1^ many of which exhibit significant medicinal properties and biological activities. For example, GSK983 is antiviral and Frovatriptan is used for migraine treatment.^2^ Great endeavors have been made toward its synthesis from synthetic and medicinal chemists and a myriad of attractive methods have been developed.^3^ The Fischer indole synthesis or Borsche–Drechsel cyclization using phenylhydrazines and ketones represents one of the most traditional and reliable approaches.^3a,4^ Other common strategies include Friedel–Crafts type reactions of substituted indoles,^5^ Lewis acid-catalyzed [3 + 3] annulation of cyclopropanes,^6^ Diels–Alder reaction or transition-metal (TM)-catalyzed [4 + 2] cycloaddition,^7^ TM or newly developed electro-catalyzed C–C or C–N formations of indole substrates^8^ etc. Despite the impressive progress in the past, each of these protocols has its own disadvantages such as limited functional group tolerance, high reaction temperature, or the employment of expensive TMs. Therefore, developing mild and versatile methods for the construction of such framework is still highly desired, which could serve as complementary approaches.

**Scheme 1:**
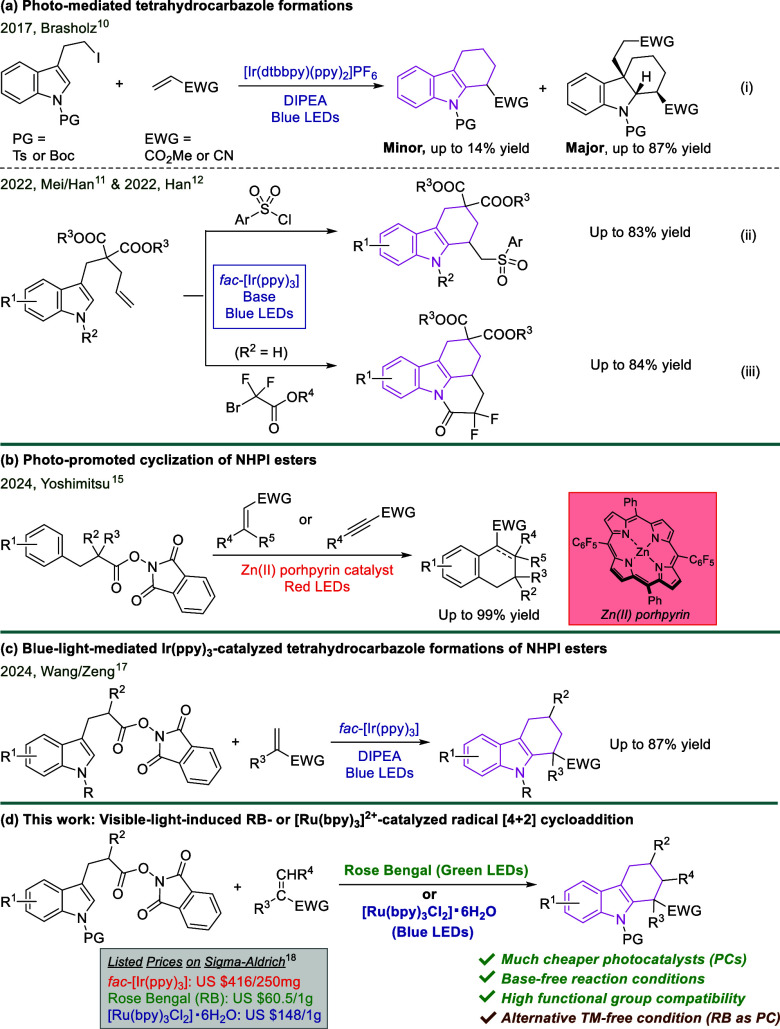
Synthesis of Tetrahydrocarbazoles through a Visible-Light-Mediated RB- or [Ru(bpy)_3_]^2+^-Catalyzed Radical [4 + 2] Cycloaddition; (a) Previous Works on Photo-Induced Tetrahydrocarbazole Formations; (b) A Photo-Mediated Cyclization of NHPI Esters; (c) A Concurrent Work; (d) This Work

Recent years, due to the renewable, nontoxic, readily available, and sustainable nature of visible light, photoredox catalysis has caught tremendous attention from researchers.^9^ By combining visible light with catalysts, an array of attractive transformations have been achieved successfully under mild conditions, including the construction of tetrahydrocarbazole derivatives.^9^ In 2017, Brasholz et al. revealed an exquisite work on a photoredox-induced Ir-catalyzed dearomative radical [4 + 2] cyclization/1,4-addition cascade (Scheme 1a-i).^10^ This protocol provided the desired highly functionalized hexahydro-*1H*-carbazoles as the major products in up to 87% yield along with tetrahydrocarbazoles as the minor byproducts in up to 14% yield. Later, the Mei and Han group introduced a delicate strategy for the synthesis of functionalized tetrahydrocarbazoles by discovering a visible-light-mediated tandem sulfonylation/cyclization of indole tethered alkenes and sulfonyl chlorides (Scheme 1a-ii).^11^ As the follow-up work, in 2022, Han and co-workers also reported the assembly of tetracyclic tetrahydrocarbazoles via a visible-light-promoted alkylation/cyclization/intramolecular aminolysis cascade of the same indole substrates with ethyl bromodifluoroacetates (Scheme 1a-iii).^12^ Despite the significant progress to date, these approaches either gave rise to tetrahydrocarbazoles as minor byproducts in low yields or provided tetrahydrocarbazole products with the limited alkyl groups at C1 position. Furthermore, an expensive iridium(III) complex was required for all the above strategies, which is neither economical nor environmental-friendly (Scheme 1a). Therefore, chemists began to search for a more general and milder catalytic system as an alternative route to construct such scaffold.

Meanwhile, *N*-hydroxyphthalimide (NHPI) esters have been broadly explored as intriguing radical precursors since their initial discoveries as redox-active esters in photocatalysis by the groups of Okada and Oda.^13^ After generating the alkyl radicals in the presence of a photocatalyst (PC), they can undergo numerous coupling reactions with different alkenes, alkynes, and aromatic rings.^14^ A representative example is the development of a red-light-promoted Zn(II) porphyrin-catalyzed radical cascade reaction by the Yoshimitsu group in 2024 (Scheme 1b),^15^ which afforded a spectrum of tetralin and dialin derivatives by reacting a NHPI ester with an electron-deficient alkene or alkyne.

To fulfill the goal of developing a milder approach for the construction of tetrahydrocarbazole skeleton as well as our ongoing efforts in photoinduced indole derivatives synthesis,^16^ we designed and synthesized a series of indole substrates with a dangling redox-active NHPI ester at C3 position. We deduced that after generating a radical intermediate in situ in the presence of a PC, it could undergo an intermolecular radical addition with an electron-deficient alkene, which then can further form the intramolecular radical [*x* + 2] cycloaddition product. Meanwhile, a concurrent work was reported by Wang, Zeng and co-workers (Scheme 1c).^17^ In their delicate work, a blue-light-mediated Ir(ppy)_3_-catalyzed decarboxylation/addition/cyclization cascade was developed, providing a convenient and effective route to synthesize tetrahydrocarbazoles under mild conditions. Herein, we present a visible-light-induced Rose Bengal (RB)- or Ru(bpy)_3_^2+^-catalyzed radical [4 + 2] cycloaddition of redox-active indole NHPI esters and electron-deficient alkenes (Scheme 1d). Though our approach features the same mechanism as the elegant method of Wang and Zeng, there are several major and minor differences. First, a much cheaper PC–Rose Bengal or [Ru(bpy)_3_Cl_2_]·6H_2_O was employed in our protocol,^18^ which represents a milder and more environmental-friendly approach especially when Rose Bengal was employed. Second, no additional base was needed in this work. Lastly, we were able to expand the terminal alkene substrates to internal ones, as well as tetrahydrocarbazole products to the possible [5 + 2] cycloaddition product.

## Results and Dicussion

Initial tests using *tert-butyl* 3-(3-((1,3-dioxoisoindolin-2-yl)oxy)-3-oxopropyl)-1H-indole-1-carboxylate **1a** and methyl vinyl ketone **2a** as model substrates in dimethyl sulfoxide (DMSO) under Ar atmosphere at room temperature were aimed at optimizing the reaction conditions and the results are summarized in Table 1. Based on the reported reduction potentials of alkyl NHPI esters (*E*_red_ = −1.20 to −1.37 V vs in CH_3_CN),^13a,17,19^ we started our examination with *fac*-Ir(ppy)_3_ (*E*_IV_/*_III_ = −1.73 V vs SCE in CH_3_CN)^9a,20^ under purple LEDs (λ_max_ = 390 nm). To our delight, the reaction proceeded smoothly, affording the desired product **3a** in 86% isolated yield (entry 1). When replacing *fac*-Ir(ppy)_3_ with the less expensive [Ru(bpy)_3_Cl_2_]·6H_2_O, **3a** was obtained in 93% yield under blue LEDs (λ_max_ = 440 nm) (entry 2). Notably, the investigation of other reported organic PCs including 4-C_Z_IPN, Rose Bengal, Eosin Y, tetraphenylporphyrin (TPP) and an inexpensive zinc phthalocyanine (ZnPc) revealed that the reaction with Rose Bengal under green LEDs (λ_max_ = 525 nm) resulted in the best outcome (74% yield) (entry 4 vs 3–7). This result was particularly encouraging since not only does it represent a TM-free catalytic system but also utilizes a relatively lower-energy and less health-risk green light. Thus, by employing Rose Bengal as the PC under green LEDs (λ_max_ = 525 nm), we further screened the solvents, reaction concentration and the equivalents of **2a**. Unfortunately, changing the solvent from DMSO to other common solvents such as tetrahydrofuran (THF), dichloromethane (DCM), toluene, CH_3_CN, *N*,*N*-dimethyl formamide (DMF), acetone, or nitromethane (MeNO_2_) did not improve the reaction outcome (entry 4 vs 8–14). Additionally, no obvious boost of the reaction yield when the reaction concentration increased from 0.1 to 0.2 M (entry 15) or the equivalents of **2a** increased from 2.0 to 5.0 (entry 16). Since [Ru(bpy)_3_Cl_2_]·6H_2_O produced the highest yield and Rose Bengal is an organic dye, we decided that both the use of Rose Bengal under green light (entry 4) and [Ru(bpy)_3_Cl_2_]·6H_2_O under blue light in DMSO (entry 2) are the optimal reaction conditions. Several control experiments have also been conducted. The reaction yield dropped to 16% when it was open to air (entry 17), suggesting the possible side reactions involving oxygen. Additionally, no reaction was observed when the reaction was carried out either in the absence of a PC under blue light or in the presence of [Ru(bpy)_3_Cl_2_]·6H_2_O in dark (entries 18 and 19). Moreover, less than 5% of **3a** was detected when the reaction was run in the presence of [Ru(bpy)_3_Cl_2_]·6H_2_O under white light (23 W CFL) (entry 20). All these control experiments indicated the essential roles for both the PC and appropriate visible light. It is also noteworthy that the oxidation potential of Rose Bengal (*E*_RB_^•+^/*_RB_ = −1.33 V vs SCE in MeOH),^9b,21^ which explains its catalytic efficiency in this reaction since the value is comparable to the reported ones of alkyl NHPI esters (*E*_red_ = −1.20 to – 1.37 V vs in CH_3_CN). Though the oxidation potential of [Ru(bpy)_3_Cl_2_]·6H_2_O was measured as *E*_III_/*_II_ = −0.81 V vs SCE in CH_3_CN,^9a,22^ it still catalyzed this radical [4 + 2] cycloaddition efficiently, presumably due to the solvent effect.

**Table 1:**
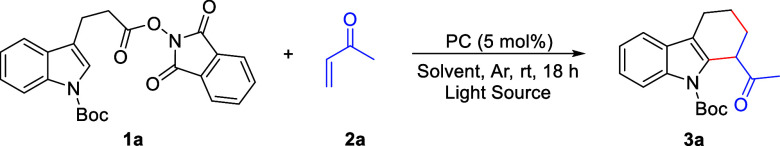
Optimization of the Reaction Conditions^a^

entry^a^	PC	light source	solvent	yield [%]^b^
1	*fac*-lr(ppy)_3_	390 nm	DMSO	86
2	[Ru(bpy)_3_Cl_2_]-6H_2_O	440 nm	DMSO	93
3	4-CzlPN	440 nm	DMSO	messy
4	Rose Bengal	525 nm	DMSO	74
5	Eosin Y	525 nm	DMSO	38
6	TPP	640 nm	DMSO	26
7	ZnPc	640 nm	DMSO	N.R.^c^
8	Rose Bengal	525 nm	THF	32
9	Rose Bengal	525 nm	DCM	<10
10	Rose Bengal	525 nm	toluene	N.R.
11	Rose Bengal	525 nm	CH_3_CN	trace
12	Rose Bengal	525 nm	DMF	71
13	Rose Bengal	525 nm	acetone	35
14	Rose Bengal	525 nm	CH_3_NO_2_	N.R.
15^d^	Rose Bengal	525 nm	DMSO	74
16^e^	Rose Bengal	525 nm	DMSO	77
17^f^	[Ru(bpy)_3_Cl_2_]-6H_2_O	440 nm	DMSO	16
18		440 nm	DMSO	N.R.
19	[Ru(bpy)_3_Cl_2_]-6H_2_O	in dark	DMSO	N.R.
20	[Ru(bpy)_3_Cl_2_]-6H_2_O	White Light^g^	DMSO	<5

a The reaction was conducted with **1a** (0.1 mmol), **2a** (0.2 mmol) and PC (5 mol %) in solvent (1.0 mL).

b Isolated yield.

c No reaction.

d 0.5 mL of DMSO was used.

e 0.5 mmol of **2a** was used.

f Open to air.

g 23 W CFL.

With both optimal reaction conditions in hand, we next sought to explore the substrate scope of electron deficient alkenes and the results are shown in Table 2. In general, condition A with Rose Bengal under green light furnished the desired [4 + 2] cycloaddition products **3b–3l** in 14–76% yields, while condition B with [Ru(bpy)_3_Cl_2_]·6H_2_O under blue light consistently provided a higher reaction yield. In detail, as expected, the monosubstituted alkenes with some common electron-withdrawing groups (EWGs) such as ester (**2b**), cyano (**2c**), and sulfonyl (**2d**) were amenable to this reaction, delivering tetrahydrocarbazole derivatives **3b** (76% vs 88%), **3c** (71% vs 91%), and **3d** (58% vs 85%) successfully. Acrylic acid (**2e**) and acrylamide (**2f**) also provided the corresponding products **3e** (44% vs 79%) and **3f** (73% vs 80%) smoothly. To our delight, styrene (**2g**) was also compatible with this approach when DMF was utilized as the solvent, providing the desired adduct **3g** in 14 and 46% yield, respectively. Albeit low reaction yields, **2g** represents an successful example of substrates with a weak EWG, which highlights the generality of this protocol. Moreover, 1,1-disubstituted alkene substrates (**2h** & **2i**) were also competent to produce **3h** and **3i** in 58–87% yields. Additionally, internal alkenes (**2j–2l**) were suitable for this protocol. While 2-cyclohexen-1-one (**2j**) gave rise to the corresponding *cis*-tetrahydrocarbazole **3j** in good yield,^23^ methyl crotonate (**2k**) provided product **3k** with two inseparable diastereomers (3.2/1 vs 4.2/1 d.r. values).^24^ When dimethyl fumarate (**2l**) and dimethyl maleate (**2l′**) were tested, **3l** was obtained in moderate yields and diastereoselectivities. Interestingly, both of **2l** and **2l′** furnished the same major tetrahydrocarbazole isomer of **3l**.^24^ It is also noteworthy that a range of diverse functional groups including ketone (**3a**&**3j**), ester (**3b**, **3h**, **3k**&**3l**), cyano (**3c**), sulfonyl (**3d**), carboxylic acid (**3e**), amide (**3f**), and aldehyde (**3i**) are well-tolerated, which renders their postmodifications feasible. Lastly, an array of other radicophiles including succinimides, quinones, ethyl propiolate, 3-butyn-2-one, propynoic acid, and dimethyl acetylenedicarboxylate were also examined. Unfortunately, either no reaction or messy reaction outcome was observed (Table S1).

**Table 2:**
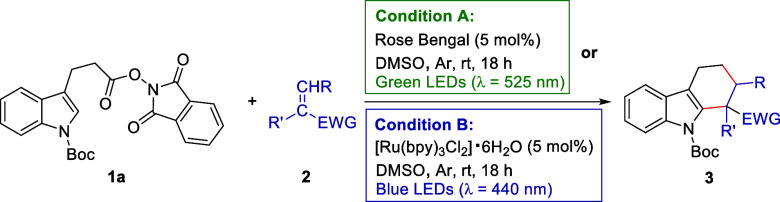

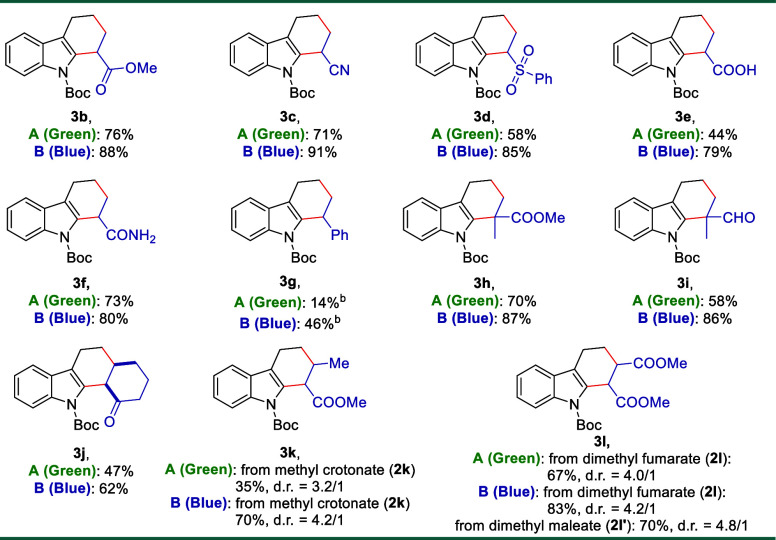
Scope of Electron Deficient Alkenes^a^

a The reaction was conducted with 1a (0.1 mmol), 2 (0.2 mmol) and PC (5 mol %) in DMSO (1.0 mL). The d.r. value was determined by the ^1^H NMR.

b DMF (1.0 mL) was used as the solvent.

Having established the scope of electron deficient alkenes in this visible-light-promoted radical [4 + 2] cycloaddition, we continued to expand the scope of the redox-active indole NHPI esters (Table 3). Overall, condition A with Rose Bengal under green light afforded the desired tetrahydrocarbazoles **3m–3x** in 43–82% yields and condition B with [Ru(bpy)_3_Cl_2_]·6H_2_O under blue light gave rise to **3m–3x** in 56–89% yields. When substrates **1** bearing a different N-protecting group such as methyl (**1b**) and benzyl (**1c**) were evaluated, the desired **3m** (44% vs 62%) and **3n** (43% vs 56%) were isolated successfully. Subsequently, the influence of substituents at different positions of the indole ring was explored. A wide range of electronically different substituents (R^1^) at the C4, C5, C6, or C7 position did not affect the reaction outcomes significantly, smoothly delivering the corresponding **3o–3w** in 51–82% yields under standard condition A and 60–89% yields under standard condition B, respectively. It is worth mentioning that substrates **1** bearing an electron-withdrawing group such as cyano (**3p**) and halide (**3q**, **3t**, **3u** & **3w**) groups produced relatively lower yields compared to the ones with an electron-donating group such as methyl (**3o**, **3s**, & **3v**) and methoxy (**3r**) groups, presumably due to the formation of less stable radical intermediates. Additionally, substrates possessing a substituent at the C7 position of the indole ring (**3v** & **3w**) tended to result in lower yields, which could be due to the steric effect of the nearby Boc group. Moreover, the expected product **3x** with two inseparable diastereomers were obtained in high yield (76% vs 86%) when **1m** with a methyl group at α position of the NHPI ester group was employed.^24^ However, substrate **1p** without any N-protecting group led to messy outcome and no desired [4 + 2] cycloaddition product was isolated (Table S1).

**Table 3:**
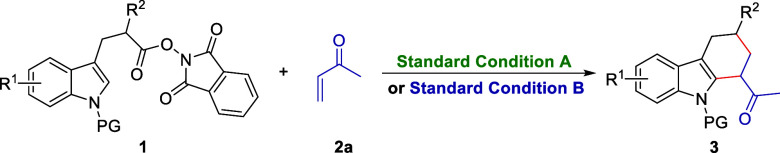

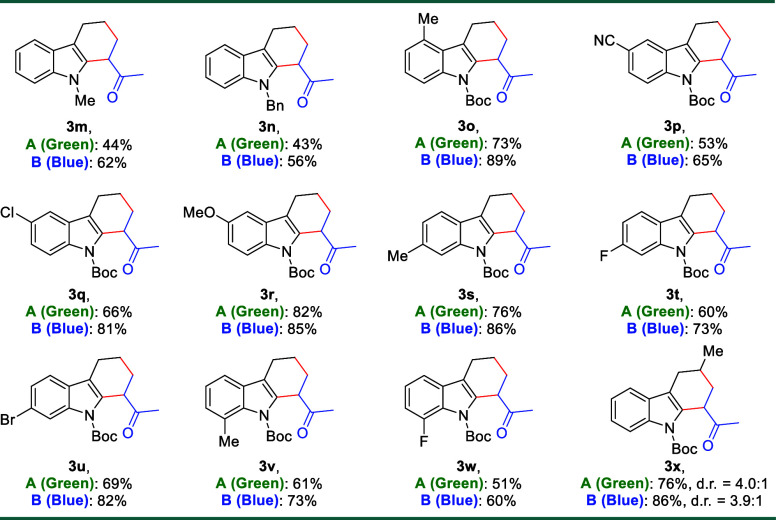
Scope of Redox-Active Indole NHPI Esters^a^

a The reaction was conducted with **1** (0.1 mmol), **2a** (0.2 mmol) and PC (5 mol %) in DMSO (1.0 mL). The d.r. value was determined by the ^1^H NMR.

**Scheme 2:**
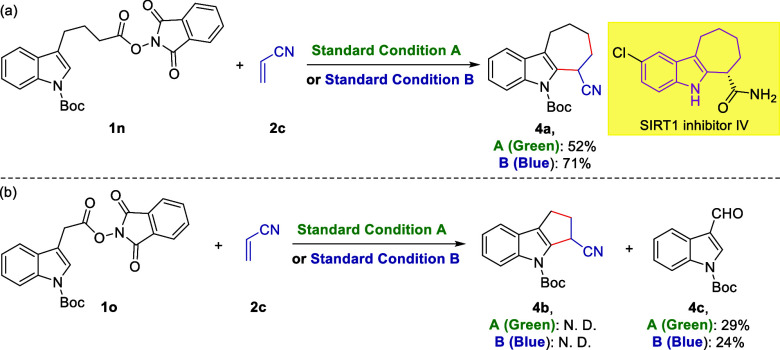
Visible-Light-Mediated Radical [5 + 2] Cycloaddition

To further investigate the possible visible-light-induced radical [x+2] cycloaddition, indole substrate **1n** with a shorter carbon and **1o** with a longer carbon tether a dangling NHPI ester group were examined under both standard conditions (Scheme 2). Gratifyingly, cyclohepta[*b*]indole **4a** was isolated in 52 and 71% yield, respectively (Scheme 2a). Notably, the core cyclohepta[*b*]indole is also a privileged framework in numerous drug molecules (e.g., SIRT1 inhibitor IV).^25^ Therefore, being able to realize this photomediated [5 + 2] cycloaddition is of great significance. However, on the contrary, **1o** failed to form the anticipated [3 + 2] product **4b**. Instead, aldehyde **4c** was obtained in 24 and 29% yield, respectively (Scheme 2b). We infer that due to the relatively higher stability of benzyl radical compared to a normal alkyl radical, it tended to react with O_2_ in the solvent or DMSO directly instead of substrate **2c**.

**Scheme 3:**
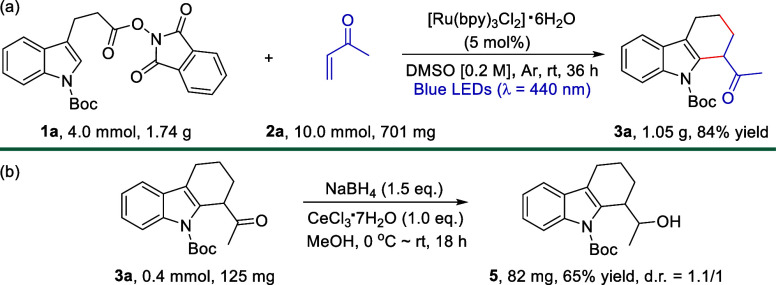
Gram-Scale Reaction and Transformation

To illustrate the synthetic utility of the present protocol, a gram-scale reaction and transformation of tetrahydrocarbazole **3a** were conducted (Scheme 3). To our delight, the reaction of **1a** in a 4.0 mmol scale (1.74 g) with **2a** (10.0 mmol, 2.5 equiv) in DMSO (20 mL) allowed the formation of **3a** in 84% isolated yield (1.05 g) when [Ru(bpy)_3_Cl_2_]·6H_2_O (5 mol %) was employed under blue light (Scheme 3a), which is relatively comparable to the yield (93%) of 0.1 mmol scale reaction. Furthermore, **3a** could be easily converted into alcohol **5** in 65% yield with a 1.1/1 d.r. value through a simple treatment with NaBH_4_ and CeCl_3_·7H_2_O in MeOH (Scheme 3b). Both experimental results highlighted the practicality and robustness of this approach.

**Scheme 4:**
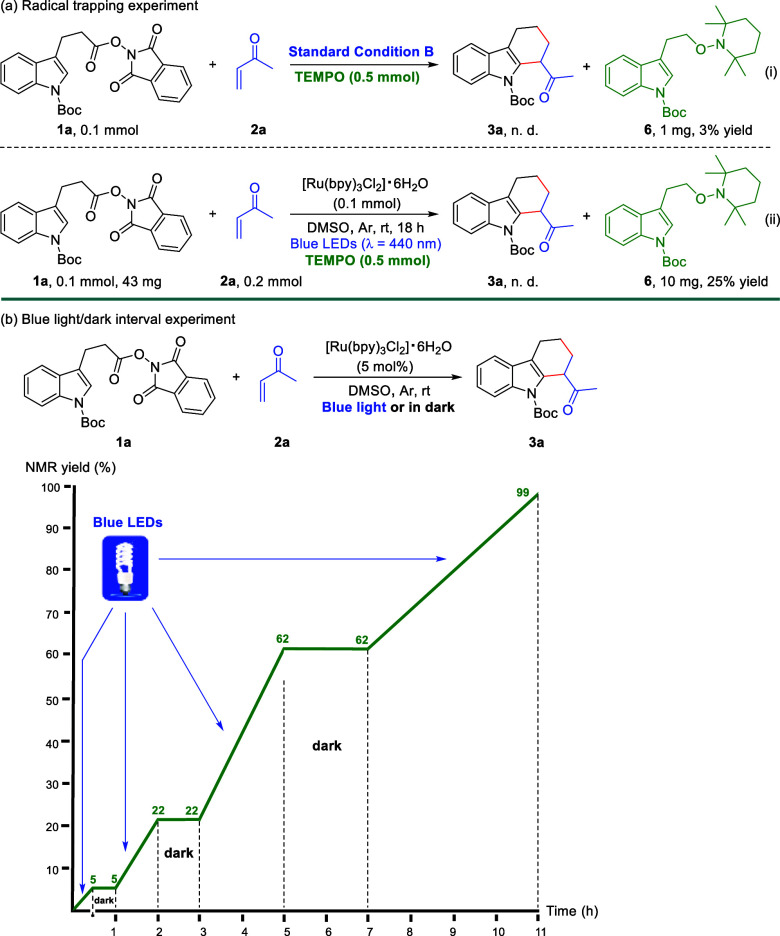
Control Experiments

To gain insight into the possible reaction mechanism, several control experiments were carried out (Scheme 4). When the reaction of **1a** and **2a** was performed under the standard condition B in the presence of 5.0 equiv of TEMPO, the desired product **3a** was not detected, while the TEMPO trapped adduct **6** was isolated in 3% yield (Scheme 4a-i). In addition, increasing [Ru(bpy)_3_Cl_2_]·6H_2_O to 1.0 equiv continued the suppression of **3a** formation while boosting the yield of **6** to 25% (Scheme 4a-ii). The isolation of **6** reveals the generation of a key alkyl radical species from **1a** through the well-studied decarboxylation process.^13−15,17^ In addition, the detection of aldehyde product **4c** from substrate **1o** further supported the formation of this alkyl radical intermediate (Scheme 2b). To verify the possibility of a radical chain propagation mechanism,^26^ a blue light/dark interval experiment under standard condition B was conducted (Scheme 4b). The results showed that the reaction of **1a** and **2a** only proceeded in the presence of blue light, further suggesting that this reaction is not likely to involve in a radical chain propagation pathway.^26^

**Scheme 5:**
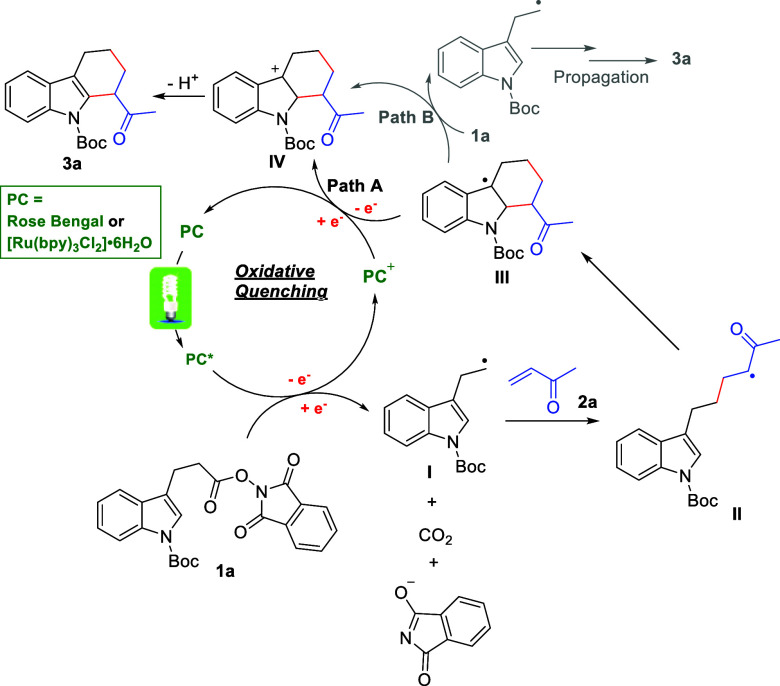
Plausible Reaction Mechanism

Based on our experimental observations and the previously reported studies,^13−15,17^ a plausible mechanism involving oxidative quenching was proposed (Scheme 5). First, PC (RB or [Ru(bpy)_3_Cl_2_]·6H_2_O) is excited to PC* under the irradiation of visible light, which reduces the redox-active indole NHPI ester **1a** to generate an alkyl radical species **I**, CO_2_, and phthalimide anion via a single electron transfer (SET) process. Then, the alkyl radical **I** adds to the radicophile **2a** to deliver another carbon radical intermediate **II**, which further undergoes intramolecular radical addition onto the C-2 position of indole ring. This step is favorable due to the formation of a relatively more stable benzylic radical **III**. Subsequently, another SET process between radical **III** and PC^+^ yields a benzyl carbocation intermediate **IV** while regenerating the ground-state PC (Path A). Lastly, a rapid aromatization process through deprotonation affords the desired tetrahydrocarbazole product **3a**. The isolation of compounds **4c** and **6** further supports the formation of the key radical species **I** (Schemes 2b & 4a). A possible reason for the low yield of **3g** could be due to the formation of relatively stable benzyl radical intermediate **II**, which disfavors the follow-up formation of radical **III**. Though it is also possible to oxidize radical **III** into carbocation **IV** through an alternative radical chain propagation mechanism (Path B),^26^ the requirement of constant irradiation of visible light indicates that it is not likely to be the major pathway (Scheme 4b). Alternatively, once the radical intermediate **III** is formed, an mechanism involving reductive quenching for [Ru(bpy)_3_Cl_2_]·6H_2_O is also possible (Scheme S1).

## Conclusion

In summary, we have disclosed a photoinduced radical [4 + 2] cycloaddition of redox-active indole NHPI esters and electron deficient alkenes in the presence of Rose Bengal under green light or [Ru(bpy)_3_Cl_2_]·6H_2_O under blue light. A series of tetrahydrocarbazoles with diverse functional groups have been synthesized in moderate to high yields under mild and base-free conditions. Notably, when Rose Bengal was employed as the PC, the protocol represents an economical and environmental-friendly catalytic system. The reaction mechanism features an oxidative quenching followed by a tandem decarboxylation/radical addition/intramolecular cyclization process. The amenable capability for the development of a [5 + 2] cycloaddition analog, scale-up reaction, and transformation experiment further highlights the synthetic potentials of this protocol. It could serve as a facile and complementary approach for the construction of tetrahydrocarbazoles and possibly cyclohepta[*b*]indoles to the reported methods.

## Experimental Section

### General Information and Methods

Unless otherwise specified, all reactions were started in oven-overnight-dried Schlenk flasks or tubes with a magnetic stir bar in an Ar filled glovebox (MBRAUN LABstar pro), which were then stirred on the bench outside under Ar atmosphere. ^1^H, ^13^C and ^19^F NMR spectra were recorded on a Bruker Ascend 500 MHz NMR spectrometers in CDCl_3_ using TMS as internal standard or the solvent signals as the standards. The chemical shifts are shown in δ scales. Multiplicities of ^1^H NMR signals are designated as s (singlet), d (doublet), t (triplet), q (quartet), dd (doublet of doublets), m (multiplet), etc. Compounds were drawn by using ChemDraw 21.0.0 and NMR spectra were processed by using MestReNova. HRMS spectra were recorded with a Bruker Impact II mass spectrometer. All chemicals and solvents were purchased from Fisher Scientific or Avantor. Anhydrous organic solvents purchased from Fisher Scientific were under N_2_ atmosphere, which were opened and used in an Ar filled glovebox only. Commercially obtained reagents were used without further purification. All reactions were monitored by TLC silica gel 60 F254 (EMD Millipore). *R*_f_ values are estimated. Flash column chromatography was carried out using Silica gel 60, 0.036–0.071 mm (215–400 mesh) (Thermo Scientific) at increased pressure. An oil bath is used as the heat source when heating is required for the reaction.

The reaction was set up as described by our previous work.^16^ Two 25 mL Schlenk tubes are placed in a water bath (Kemtech crystallizing dish) at the center of a magnetic stirrer. Two parallel Blue or Green LED lamps (KSPR160L-440 nm, Blue LED, Kessil LED Lights or KSPR160L-525 nm, Green LED, Kessil LED Lights) are placed perpendicular to the side of the two Schlenk tubes, so that they are equally exposed to the lights. The magnetic stirrer/water bath/LED lamps are surrounded by a cardboard box covered with aluminum foil to increase the light reflections. A small fan near the water bath is always on when the reaction is running. The combination of the water bath and fan is to offset the heat generated from the LED lamps and keep the reaction around room temperature (21–23 °C). Distance of fan to water bath ≈ 15 cm. Distance of LED lamps to Schlenk tubes ≈ 7 cm. Product **4c**(27) is a known compound in literature.

### General Procedure for the Synthesis of Tetrahydrocarbazoles **3** or Compounds **4**

In an Ar glovebox, the substrate **1** (0.1 mmol, 1.0 equiv) and PC (0.005 mmol, 5 mol %) were added to an oven-dried (overnight) Schlenk tube containing a stirring bar, followed by adding anhydrous DMSO (1.0 mL) and **2** (0.2 mmol, 2.0 equiv). The Schlenk tube was then sealed, removed from the glovebox, and the mixture was stirred at room temperature under Green LEDs (λ_max_ = 525 nm) or Blue LEDs (λ_max_ = 440 nm) irradiation. After 18 h, the reaction mixture was monitored by TLC. The crude product was purified by flash chromatography (FC) on silica gel (eluent: Hexanes/EtOAc = 50/1–1/1) to yield the desired product **3** or **4**.

#### *tert*-Butyl 1-Acetyl-1,2,3,4-tetrahydro-9*H*-carbazole-9-carboxylate (**3a**)

Yield (0.1 mmol scale, **Green**: 23 mg, 74% yield; **Blue**: 29 mg, 93% yield). A colorless solid. *R*_f_ = 0.5 (Hexanes/EtOAc = 4/1). FC (Hexanes/EtOAc = 10/1). ^1^H NMR (500 MHz, CDCl_3_): δ 7.91 (d, *J* = 7.5 Hz, 1H, ArH), 7.35 (d, *J* = 6.5 Hz, 1H, ArH), 7.20–7.16 (m, 1H, ArH), 7.13 (dd, *J* = 7.5 Hz, 7.5 Hz, 1H, ArH), 4.29 (dd, *J* = 7.0 Hz, 5.0 Hz, 1H, CH), 2.67 (ddd, *J* = 16.5, 5.5, 5.0 Hz, 1H, CH_2_), 2.59–2.52 (m, 1H, CH_2_), 2.19 (s, 3H, Me), 2.07–1.98 (m, 1H, CH_2_), 1.98–1.90 (m, 1H, CH_2_), 1.81–1.70 (m, 2H, CH_2_), 1.56 (s, 9H, Me). ^13^C{^1^H} NMR (126 MHz, CDCl_3_): δ 208.9, 150.7, 135.6, 133.0, 129.5, 124.0, 122.5, 119.0, 118.2, 115.7, 83.9, 48.6, 28.4, 28.2, 27.2, 21.0, 19.8. HRMS (ESI) calcd for C_19_H_24_NO_3_^1+^ (M^+^ + 1) requires, 314.1751; found, 314.1749.

#### 9-(*tert*-Butyl) 1-Methyl 1,2,3,4-Tetrahydro-9*H*-carbazole-1,9-dicarboxylate (**3b**)

Yield (0.1 mmol scale, **Green**: 25 mg, 76% yield; **Blue**: 29 mg, 88% yield). A colorless oily solid. *R*_f_ = 0.5 (Hexanes/EtOAc = 4/1). FC (Hexanes/EtOAc = 10/1). ^1^H NMR (500 MHz, CDCl_3_): δ 8.02 (d, *J* = 8.0 Hz, 1H, ArH), 7.35 (d, *J* = 7.5 Hz, 1H, ArH), 7.25–7.08 (m, 2H, ArH), 4.21 (dd, *J* = 5.5 Hz, 5.5 Hz, 1H, CH), 3.61 (s, 3H, OMe), 2.68 (ddd, *J* = 16.5, 5.5, 5.0 Hz, 1H, CH_2_), 2.55 (ddd, *J* = 16.0, 8.0, 8.0 Hz, 1H, CH_2_), 2.17–2.11 (m, 1H, CH_2_), 2.09–2.02 (m, 1H, CH_2_), 1.88–1.66 (m, 2H, CH_2_), 1.56 (s, 9H, Me). ^13^C{^1^H} NMR (126 MHz, CDCl_3_): δ 174.3, 150.6, 135.9, 131.7, 129.3, 124.2, 122.4, 118.6, 118.1, 115.7, 83.8, 52.0, 42.0, 28.2, 28.2, 21.0, 20.1. HRMS (ESI) calcd for C_19_H_24_NO_4_^1+^ (M^+^ + 1) requires, 330.1700; found, 330.1696.

#### *tert*-Butyl 1-Cyano-1,2,3,4-tetrahydro-9*H*-carbazole-9-carboxylate (**3c**)

Yield (0.1 mmol scale, **Green**: 21 mg, 71% yield; **Blue**: 27 mg, 91% yield). A white solid. *R*_f_ = 0.5 (Hexanes/EtOAc = 4/1). FC (Hexanes/EtOAc = 10/1). ^1^H NMR (500 MHz, CDCl_3_): δ 8.10 (d, *J* = 8.5 Hz, 1H, ArH), 7.36 (d, *J* = 8.0 Hz, 1H, ArH), 7.30–7.23 (m, 1H, ArH), 7.20–7.16 (m, 1H, ArH), 4.42 (dd, *J* = 4.0, 3.5 Hz, 1H, CH), 2.81–2.71 (m, 1H, CH_2_), 2.60–2.49 (m, 1H, CH_2_), 2.35–2.24 (m, 1H, CH_2_), 2.05–1.91 (m, 3H, CH_2_), 1.65 (s, 9H, Me). ^13^C{^1^H} NMR (126 MHz, CDCl_3_): δ 150.0, 136.1, 128.6, 127.0, 125.2, 122.9, 120.5, 119.6, 118.4, 115.9, 85.0, 28.2, 28.2, 28.0, 20.5, 19.3. HRMS (ESI) calcd for C_18_H_20_NaN_2_O_2_^1+^ (M^+^ + Na) requires 319.1417; found, 319.1418.

#### *tert*-Butyl 1-(Phenylsulfonyl)-1,2,3,4-tetrahydro-9*H*-carbazole-9-carboxylate (**3d**)

Yield (0.1 mmol scale, **Green**: 24 mg, 58% yield; **Blue**: 35 mg, 85% yield). A white solid. *R*_f_ = 0.4 (Hexanes/EtOAc = 4/1). FC (Hexanes/EtOAc = 8/1). ^1^H NMR (500 MHz, CDCl_3_): δ 7.94 (d, *J* = 8.5 Hz, 1H, ArH), 7.75 (d, *J* = 7.5 Hz, 2H, ArH), 7.52 (dd, *J* = 7.5, 7.5 Hz, 1H, ArH), 7.45–7.35 (m, 3H, ArH), 7.25 (dd, *J* = 7.5, 7.5 Hz, 1H, ArH), 7.16 (dd, *J* = 7.5, 7.5 Hz, 1H, ArH), 5.52 (d, *J* = 5.5 Hz, 1H, CH), 2.68 (dd, *J* = 16.5, 5.5 Hz, 1H, CH_2_), 2.58–2.47 (m, 1H, CH_2_), 2.30 (dd, *J* = 14.5, 2.0 Hz, 1H, CH_2_), 2.20–2.05 (m, 1H, CH_2_), 2.03–1.92 (m, 1H, CH_2_), 1.72 (dd, *J* = 16.5, 6.0 Hz, 1H, CH_2_), 1.62 (s, 9H, Me). ^13^C{^1^H} NMR (126 MHz, CDCl_3_): δ 150.7, 139.0, 136.9, 133.5, 129.0, 128.9, 128.4, 125.2, 125.2, 123.0, 122.4, 118.7, 115.4, 84.2, 59.6, 28.3, 24.3, 19.8, 17.1. HRMS (ESI) calcd for C_23_H_25_NaNO_4_S^1+^ (M^+^ + Na) requires, 434.1397; found, 434.1401.

#### 9-(*tert*-Butoxycarbonyl)-2,3,4,9-tetrahydro-1*H*-carbazole-1-carboxylic Acid (**3e**)

Yield (0.1 mmol scale, **Green**: 14 mg, 44% yield; **Blue**: 25 mg, 79% yield). A colorless solid. *R*_f_ = 0.3 (Hexanes/EtOAc = 2/1). FC (Hexanes/EtOAc = 3/1–2/1). ^1^H NMR (500 MHz, CDCl_3_): δ 8.01 (d, *J* = 8.0 Hz, 1H, ArH), 7.36 (d, *J* = 6.5 Hz, 1H, ArH), 7.23–7.18 (m, 1H, ArH), 7.14 (ddd, *J* = 8.0, 7.5, 1.0 Hz, 1H, ArH), 4.24 (dd, *J* = 6.5, 4.0 Hz, 1H, CH), 2.70 (ddd, *J* = 16.5, 5.0, 5.0 Hz, 1H, CH_2_), 2.56 (ddd, *J* = 16.5, 8.0, 8.0 Hz, 1H, CH_2_), 2.24–2.15 (m, 1H, CH_2_), 2.12–2.03 (m, 1H, CH_2_), 1.88–1.74 (m, 2H, CH_2_), 1.57 (s, 9H, Me). ^13^C{^1^H} NMR (126 MHz, CDCl_3_): δ 179.6, 150.7, 135.9, 131.0, 129.1, 124.3, 122.5, 118.9, 118.2, 115.7, 84.1, 41.7, 28.2, 28.0, 21.0, 19.9. HRMS (ESI) calcd for C_18_H_22_NO_4_^1+^ (M^+^ + 1) requires, 316.1543; found, 316.1545.

#### *tert*-Butyl 1-Carbamoyl-1,2,3,4-tetrahydro-9*H*-carbazole-9-carboxylate (**3f**)

Yield (0.1 mmol scale, **Green**: 23 mg, 73% yield; **Blue**: 25 mg, 80% yield). A white solid. *R*_f_ = 0.4 (Hexanes/EtOAc = 1/1). FC (Hexanes/EtOAc = 1.5/1–1/1). ^1^H NMR (500 MHz, CDCl_3_): δ 8.10 (d, *J* = 8.0 Hz, 1H, ArH), 7.38 (d, *J* = 7.5 Hz, 1H, ArH), 7.24 (ddd, *J* = 8.0, 7.5, 1.0 Hz, 1H, ArH), 7.16 (dd, *J* = 7.5, 1.0 Hz, 1H, ArH), 5.70 (br s, 1H, NH_2_), 5.37 (br s, 1H, NH_2_), 4.10 (dd, *J* = 5.0, 4.5 Hz, 1H, CH), 2.73 (ddd, *J* = 16.5, 6.0, 5.0 Hz, 1H, CH_2_), 2.63–2.51 (m, 1H, CH_2_), 2.31–2.21 (m, 1H, CH_2_), 2.02–1.93 (m, 1H, CH_2_), 1.90–1.75 (m, 2H, CH_2_), 1.58 (s, 9H, Me). ^13^C{^1^H} NMR (126 MHz, CDCl_3_): δ 176.3, 150.3, 136.3, 131.7, 129.0, 124.7, 122.8, 119.5, 118.3, 115.9, 84.6, 43.2, 28.5, 28.2, 21.0, 19.3. HRMS (ESI) calcd for C_18_H_23_N_2_O_3_^1+^ (M^+^ + 1) requires, 315.1703; found, 315.1699.

#### *tert*-Butyl 1-Phenyl-1,2,3,4-tetrahydro-9*H*-carbazole-9-carboxylate (**3g**)

Yield (0.1 mmol scale, **Green**: 5 mg, 14% yield; **Blue**: 16 mg, 46% yield). A colorless oily solid. *R*_f_ = 0.8 (Hexanes/EtOAc = 15/1). FC (Hexanes/EtOAc = 50/1). ^1^H NMR (500 MHz, CDCl_3_): δ 8.11 (d, *J* = 8.5 Hz, 1H, ArH), 7.40 (d, *J* = 7.5 Hz, 1H, ArH), 7.24–7.13 (m, 4H, ArH), 7.06 (dd, *J* = 7.5, 7.5 Hz, 1H, ArH), 6.88 (d, *J* = 7.0 Hz, 2H, ArH), 4.66 (dd, *J* = 5.0, 4.5 Hz, 1H, CH), 2.73 (ddd, *J* = 16.5, 6.0, 3.0 Hz, 1H, CH_2_), 2.63–2.56 (m, 1H, CH_2_), 2.22–2.12 (m, 1H, CH_2_), 2.00–1.92 (m, 1H, CH_2_), 1.70–1.61 (m, 1H, CH_2_), 1.56–1.49 (m, 1H, CH_2_), 1.22 (s, 9H, Me). ^13^C NMR (126 MHz, CDCl_3_): δ 150.2, 145.4, 136.6, 135.1, 129.4, 128.0, 127.9, 125.8, 124.0, 122.4, 118.9, 117.9, 115.6, 83.2, 40.6, 33.6, 27.8, 21.3, 17.9. HRMS (ESI) calcd for C_23_H_25_NaNO_2_^1+^ (M^+^+Na) requires, 370.1777; found, 370.1781.

#### 9-(*tert*-Butyl) 1-Methyl 1-Methyl-1,2,3,4-tetrahydro-9*H*-carbazole-1,9-dicarboxylate (**3h**)

Yield (0.1 mmol scale, **Green**: 24 mg, 70% yield; **Blue**: 30 mg, 87% yield). A colorless oily solid. *R*_f_ = 0.5 (Hexanes/EtOAc = 4/1). FC (Hexanes/EtOAc = 12/1). ^1^H NMR (500 MHz, CDCl_3_): δ 7.91 (d, *J* = 7.5 Hz, 1H, ArH), 7.35 (dd, *J* = 7.5, 1.5 Hz, 1H, ArH), 7.19 (ddd, *J* = 7.5, 7.5, 1.5 Hz, 1H, ArH), 7.14 (ddd, *J* = 7.5, 7.5, 1.5 Hz, 1H, ArH), 3.54 (s, 3H, OMe), 2.68 (ddd, *J* = 16.0, 6.0, 3.0 Hz, 1H, CH_2_), 2.58 (ddd, *J* = 16.0, 10.0, 5.0 Hz, 1H, CH_2_), 2.02–1.76 (m, 4H, CH_2_), 1.60 (s, 3H, Me), 1.59 (s, 9H, Me). ^13^C{^1^H} NMR (126 MHz, CDCl_3_): δ 176.9, 150.4, 137.1, 135.6, 129.4, 124.1, 122.5, 118.3, 117.5, 115.9, 83.9, 51.9, 45.0, 37.9, 28.2, 23.5, 21.3, 18.7. HRMS (ESI) calcd for C_20_H_25_NaNO_4_^1+^ (M^+^+Na) requires, 366.1676; found, 366.1674.

#### *tert*-Butyl 1-Formyl-1-methyl-1,2,3,4-tetrahydro-9*H*-carbazole-9-carboxylate (**3i**)

Yield (0.1 mmol scale, **Green**: 18 mg, 58% yield; **Blue**: 27 mg, 86% yield). A white solid. *R*_f_ = 0.6 (Hexanes/EtOAc = 4/1). FC (Hexanes/EtOAc = 20/1). ^1^H NMR (500 MHz, CDCl_3_): δ 9.53 (s, 1H, CHO), 7.90 (d, *J* = 8.0 Hz, 1H, ArH), 7.37 (d, *J* = 7.0 Hz, 1H, ArH), 7.22 (ddd, *J* = 8.0, 7.0, 1.5 Hz, 1H, ArH), 7.16 (ddd, *J* = 8.0, 7.0, 1.5 Hz, 1H, ArH), 2.70 (ddd, *J* = 16.0, 4.5, 2.0 Hz, 1H, CH_2_), 2.62–2.52 (m, 1H, CH_2_), 1.96–1.87 (m, 1H, CH_2_), 1.84–1.73 (m, 2H, CH_2_), 1.59 (s, 10H, CH_2_, Me), 1.49 (s, 3H, Me). ^13^C{^1^H} NMR (126 MHz, CDCl_3_): δ 200.6, 150.6, 136.2, 135.6, 129.4, 124.5, 122.7, 119.6, 118.4, 115.8, 84.6, 48.3, 34.3, 28.2, 21.3, 20.8, 18.4. HRMS (ESI) calcd for C_19_H_24_NO_3_^1+^ (M^+^ + 1) requires, 314.1751; found, 314.1749.

#### *tert*-Butyl 1-Oxo-1,2,3,4,4a,5,6,11b-octahydro-11*H*-benzo[*a*]carbazole-11-carboxylate (**3j**)

Yield (0.1 mmol scale, **Green**: 16 mg, 47% yield; **Blue**: 21 mg, 62% yield). A colorless oily solid. *R*_f_ = 0.5 (Hexanes/EtOAc = 4/1). FC (Hexanes/EtOAc = 15/1). ^1^H NMR (500 MHz, CDCl_3_): δ 8.03 (d, *J* = 8.0 Hz, 1H, ArH), 7.32 (d, *J* = 7.5 Hz, 1H, ArH), 7.19–7.16 (m, 1H, ArH), 7.12 (ddd, *J* = 7.5, 7.5, 1.5 Hz, 1H, ArH), 4.43 (d, *J* = 5.5 Hz, 1H, CH), 2.74 (ddd, *J* = 16.5, 4.5, 3.0 Hz, 1H, CH_2_), 2.58–2.45 (m, 3H, CH_2_, CH), 2.39–2.34 (m, 1H, CH_2_), 2.15–2.04 (m, 1H, m, 1H, CH_2_), 2.02–1.94 (m, 1H, CH_2_), 1.92–1.79 (m, 2H, CH_2_), 1.63–1.59 (m, 2H, CH_2_), 1.54 (s, 9H, Me). ^13^C{^1^H} NMR (126 MHz, CDCl_3_): δ 209.3, 150.5, 136.4, 132.7, 129.2, 123.8, 122.3, 117.9, 117.4, 115.9, 83.3, 51.8, 42.1, 39.8, 31.2, 28.3, 25.0, 23.6, 21.1. HRMS (ESI) calcd for C_21_H_26_NO_3_^1+^ (M^+^ + 1) requires 340.1907; found, 340.1908.

#### 9-(*tert*-Butyl) 1-Methyl 2-Methyl-1,2,3,4-tetrahydro-9*H*-carbazole-1,9-dicarboxylate (**3k**)

Yield (0.1 mmol scale, Green: 12 mg, 35% yield, d.r. = 3.2/1 & Blue: 24 mg, 70% yield, d.r. = 4.2/1). A colorless oily solid. *R*_f_ = 0.6 (Hexanes/EtOAc = 4/1). FC (Hexanes/EtOAc = 20/1). ^1^H NMR (500 MHz, CDCl_3_): δ 7.99 (d, *J* = 8.5 Hz, 1H, ArH), 7.37 (d, *J* = 7.5 Hz, 1H, ArH), 7.23–7.18 (m, 1H, ArH), 7.15 (dd, *J* = 7.5, 7.5 Hz, 1H, ArH), 3.89 (d, *J* = 4.5 Hz, 1H, CH), 3.60 (s, 3H, Me), 2.70–2.54 (m, 2H, CH_2_), 2.41–2.29 (m, 1H, CH), 1.87–1.78 (m, 1H, CH_2_), 1.63–1.55 (m, 10H, Me, CH_2_), 1.11 (d, *J* = 7.0 Hz, 3H, Me). ^13^C{^1^H} NMR (126 MHz, CDCl_3_): δ 174.1, 150.7, 136.0, 131.3, 129.2, 124.1, 122.4, 118.2, 117.9, 115.7, 83.8, 51.9, 49.1, 32.8, 28.2, 27.0, 19.3, 18.0. HRMS (ESI) calcd for C_20_H_25_NaNO_4_^1+^ (M^+^ + Na) requires, 366.1676; found, 366.1672.

#### 9-(*tert*-Butyl) 1,2-Dimethyl 1,2,3,4-Tetrahydro-9*H*-carbazole-1,2,9-tricarboxylate (**3l**)

Yield (0.1 mmol scale; From dimethyl fumarate (**2l**), **Green**: 26 mg, 67% yield, d.r. = 4.0/1 & **Blue**: 32 mg, 83% yield, d.r. = 4.2/1; From dimethyl mealate (**2l′**)*,***Blue**: 27 mg, 70% yield, d.r. = 4.8/1). A colorless oily solid. *R*_f_ = 0.4 (Hexanes/EtOAc = 4/1). FC (Hexanes/EtOAc = 8/1). ^1^H NMR (500 MHz, CDCl_3_): δ 8.02 (d, *J* = 8.5 Hz, 1H, ArH), 7.34 (d, *J* = 7.5 Hz, 1H, ArH), 7.21 (ddd, *J* = 8.5, 7.5, 1.5 Hz, 1H, ArH), 7.14 (ddd, *J* = 7.5, 7.5, 1.5 Hz, 1H, ArH), 4.81 (d, *J* = 3.5 Hz, 1H, CH), 3.62 (s, 6H, overlapped Me), 3.24 (ddd, *J* = 6.0, 3.5, 3.5 Hz, 1H, CH), 2.72–2.57 (m, 2H, CH_2_), 2.30–2.21 (m, 1H, CH_2_), 1.95–1.86 (m, 1H, CH_2_), 1.57 (s, 9H, Me). ^13^C{^1^H} NMR (126 MHz, CDCl_3_): δ 173.3, 173.0, 150.5, 136.1, 130.2, 128.9, 124.3, 122.5, 118.2, 117.6, 115.7, 84.0, 52.3, 52.3, 43.6, 43.4, 28.2, 22.4, 18.5. HRMS (ESI) calcd for C_21_H_25_NO_6_^1+^ (M^+^ + 1) requires, 388.1755; found, 388.1758.

#### 1-(9-Methyl-2,3,4,9-tetrahydro-1*H*-carbazol-1-yl)ethan-1-one (**3m**)

Yield (0.1 mmol scale, **Green**: 10 mg, 44% yield; **Blue**: 14 mg, 62% yield). A light yellow solid. *R*_f_ = 0.4 (Hexanes/EtOAc = 4/1). FC (Hexanes/EtOAc = 8/1). ^1^H NMR (500 MHz, CDCl_3_): δ 7.45 (d, *J* = 8.0 Hz, 1H, ArH), 7.19 (dd, *J* = 8.0, 8.0 Hz, 1H, ArH), 7.13 (ddd, *J* = 8.0, 7.0, 1.0 Hz, 1H, ArH), 7.02 (ddd, *J* = 8.0, 7.0, 1.0 Hz, 1H, ArH), 3.77 (dd, *J* = 7.0, 5.5 Hz, 1H, CH), 3.44 (s, 3H, Me), 2.75 (ddd, *J* = 16.0, 5.5, 5.5 Hz, 1H, CH_2_), 2.72–2.64 (m, 1H, CH_2_), 2.16–1.99 (m, 5H, Me, CH_2_), 1.83–1.77 (m, 2H, CH_2_). ^13^C{^1^H} NMR (126 MHz, CDCl_3_): δ 209.9, 137.3, 132.5, 126.8, 121.6, 119.0, 118.4, 111.5, 108.9, 47.9, 29.6, 27.7, 27.4, 21.0, 21.0. HRMS (ESI) calcd for C_15_H_18_NO^1+^ (M^+^ + 1) requires, 228.1383; found, 228.1383.

#### 1-(9-Benzyl-2,3,4,9-tetrahydro-1*H*-carbazol-1-yl)ethan-1-one (**3n**)

Yield (0.1 mmol scale, **Green**: 13 mg, 43% yield; **Blue**: 17 mg, 56% yield). A white solid. *R*_f_ = 0.5 (Hexanes/EtOAc = 4/1). FC (Hexanes/EtOAc = 10/1). ^1^H NMR (500 MHz, CDCl_3_): δ 7.48 (ddd, *J* = 7.5, 1.5, 1.0 Hz, 1H, ArH), 7.21–7.10 (m, 4H, ArH), 7.08 (ddd, *J* = 8.0, 6.5, 1.5 Hz, 1H, ArH), 7.03 (ddd, *J* = 8.0, 7.5, 1.0 Hz, 1H, ArH), 6.90–6.83 (m, 2H, ArH), 5.22 (d, *J* = 17.0 Hz, 1H, CH_2_), 4.90 (d, *J* = 17.0 Hz, 1H, CH_2_), 3.63 (dd, *J* = 6.5, 5.0 Hz, 1H, CH), 2.80 (ddd, *J* = 15.9, 5.0, 4.5 Hz, 1H, CH_2_), 2.75–2.67 (m, 1H, CH_2_), 2.09–1.99 (m, 2H, CH_2_), 1.96 (s, 3H, Me), 1.82–1.75 (m, 2H, CH_2_). ^13^C{^1^H} NMR (126 MHz, CDCl_3_): δ 209.7, 137.6, 137.3, 132.5, 128.8, 127.4, 127.1, 126.2, 121.9, 119.2, 118.5, 112.3, 109.5, 47.7, 46.8, 27.9, 27.4, 21.0, 20.6. HRMS (ESI) calcd for C_21_H_22_NO^1+^ (M^+^ + 1) requires, 304.1696; found, 304.1693.

#### *tert*-Butyl 1-Acetyl-5-methyl-1,2,3,4-tetrahydro-9*H*-carbazole-9-carboxylate (**3o**)

Yield (0.1 mmol scale, **Green**: 24 mg, 73% yield; **Blue**: 29 mg, 89% yield). A colorless oily solid. *R*_f_ = 0.5 (Hexanes/EtOAc = 4/1). FC (Hexanes/EtOAc = 10/1). ^1^H NMR (500 MHz, CDCl_3_): δ 7.78 (d, *J* = 8.5 Hz, 1H, ArH), 7.03 (dd, *J* = 8.5, 7.0 Hz, 1H, ArH), 6.85 (d, *J* = 7.0 Hz, 1H, ArH), 4.30 (dd, *J* = 6.5, 5.0 Hz, 1H, CH), 3.03–2.94 (ddd, *J* = 16.0, 5.5, 5.0 Hz, 1H, CH_2_), 2.88–2.78 (m, 1H, CH_2_), 2.55 (s, 3H, Me), 2.19 (s, 3H, Me), 2.02–1.93 (m, 1H, CH_2_), 1.93–1.85 (m, 1H, CH_2_), 1.79–1.69 (m, 2H, CH_2_), 1.55 (s, 9H, Me). ^13^C{^1^H} NMR (126 MHz, CDCl_3_): δ 209.0, 150.7, 135.8, 132.6, 130.6, 128.1, 124.3, 123.7, 119.6, 113.5, 83.9, 48.7, 28.4, 28.2, 26.5, 24.3, 20.3, 20.2. HRMS (ESI) calcd for C_20_H_26_NO_3_^1+^ (M^+^ + 1) requires, 328.1907; found, 328.1905.

#### *tert*-Butyl 1-Acetyl-6-cyano-1,2,3,4-tetrahydro-9*H*-carbazole-9-carboxylate (**3p**)

Yield (0.1 mmol scale, **Green**: 18 mg, 53% yield; **Blue**: 22 mg, 65% yield). A white solid. *R*_f_ = 0.3 (Hexanes/EtOAc = 4/1). FC (Hexanes/EtOAc = 6/1). ^1^H NMR (500 MHz, CDCl_3_): δ 7.96 (d, *J* = 8.5 Hz, 1H, ArH), 7.67 (d, *J* = 1.5 Hz, 1H, ArH), 7.43 (dd, *J* = 8.5, 1.5 Hz, 1H, ArH), 4.35 (dd, *J* = 6.5, 5.0 Hz, 1H, CH), 2.67 (ddd, *J* = 16.5, 5.5, 5.0 Hz, 1H, CH_2_), 2.59–2.50 (m, 1H, CH_2_), 2.24 (s, 3H, Me), 2.011–2.02 (m, 1H, CH_2_), 2.01–1.94 (m, 1H, CH_2_), 1.84–1.69 (m, 2H, CH_2_), 1.57 (s, 9H, Me). ^13^C{^1^H} NMR (126 MHz, CDCl_3_): δ 208.0, 150.0, 137.5, 135.5, 129.6, 127.1, 122.9, 119.9, 118.7, 116.4, 105.7, 85.2, 48.4, 28.4, 28.2, 26.7, 20.7, 19.4. HRMS (ESI) calcd for C_20_H_23_N_2_O_3_^1+^ (M^+^ + 1) requires, 339.1703; found, 339.1700.

#### *tert*-Butyl 1-Acetyl-6-chloro-1,2,3,4-tetrahydro-9*H*-carbazole-9-carboxylate (**3q**)

Yield (0.1 mmol scale, **Green**: 23 mg, 66% yield; **Blue**: 28 mg, 81% yield). A white solid. *R*_f_ = 0.5 (Hexanes/EtOAc = 4/1). FC (Hexanes/EtOAc = 10/1). ^1^H NMR (500 MHz, CDCl_3_): δ 7.81 (d, *J* = 9.0 Hz, 1H, ArH), 7.31 (d, *J* = 2.0 Hz, 1H, ArH), 7.12 (dd, *J* = 9.0, 2.0 Hz, 1H, ArH), 4.30 (dd, *J* = 6.5, 5.0 Hz, 1H, CH), 2.62 (ddd, *J* = 16.5, 5.5, 5.0 Hz, 1H, CH_2_), 2.54–2.46 (m, 1H, CH_2_), 2.20 (s, 3H, Me), 2.06–2.00 (m, 1H, CH_2_), 1.97–1.91 (m, 1H, CH_2_), 1.81–1.66 (m, 2H, CH_2_), 1.55 (s, 9H, Me). ^13^C{^1^H} NMR (126 MHz, CDCl_3_): δ 208.4, 150.3, 134.4, 133.9, 130.7, 128.1, 124.0, 118.4, 117.8, 116.7, 84.3, 48.5, 28.4, 28.2, 26.9, 20.8, 19.6. HRMS (ESI) calcd for C_19_H_23_ClNO_3_^1+^ (M^+^ + 1) requires, 348.1361; found, 348.1357.

#### *tert*-Butyl 1-Acetyl-6-methoxy-1,2,3,4-tetrahydro-9*H*-carbazole-9-carboxylate (**3r**)

Yield (0.1 mmol scale, **Green**: 28 mg, 82% yield; **Blue**: 29 mg, 85% yield). A white solid. *R*_f_ = 0.5 (Hexanes/EtOAc = 4/1). FC (Hexanes/EtOAc = 10/1). ^1^H NMR (500 MHz, CDCl_3_): δ 7.79 (d, *J* = 9.0 Hz, 1H, ArH), 6.84–6.75 (m, 2H, ArH), 4.27 (dd, *J* = 6.5, 4.5 Hz, 1H, CH), 3.78 (s, 3H, OMe), 2.64 (dd, *J* = 16.0, 5.5, 4.5 Hz, 1H, CH_2_), 2.55–2.48 (m, 1H, CH_2_), 2.18 (s, 3H, Me), 2.06–1.97 (m, 1H, CH_2_), 1.97–1.89 (m, 1H, CH_2_), 1.81–1.69 (m, 2H, CH_2_), 1.55 (s, 9H, Me). ^13^C{^1^H} NMR (126 MHz, CDCl_3_): δ 208.9, 155.8, 150.6, 133.7, 130.3, 130.2, 118.8, 116.4, 112.3, 101.1, 83.7, 55.8, 48.6, 28.4, 28.2, 27.1, 21.0, 19.8. HRMS (ESI) calcd for C_20_H_26_NO_4_^1+^ (M^+^ + 1) requires, 344.1856; found, 344.1852.

#### *tert*-Butyl 1-Acetyl-7-methyl-1,2,3,4-tetrahydro-9*H*-carbazole-9-carboxylate (**3s**)

Yield (0.1 mmol scale, **Green**: 25 mg, 76% yield; **Blue**: 28 mg, 86% yield). A white solid. *R*_f_ = 0.5 (Hexanes/EtOAc = 4/1). FC (Hexanes/EtOAc = 10/1). ^1^H NMR (500 MHz, CDCl_3_): δ 7.78 (br s, 1H, ArH), 7.23 (d, *J* = 8.0 Hz, 1H, ArH), 6.97 (dd, *J* = 8.0, 1.5 Hz, 1H, ArH), 4.24 (dd, *J* = 6.5, 5.0 Hz, 1H, CH), 2.69–2.61 (ddd, *J* = 16.0, 6.0, 4.5 Hz, 1H, CH_2_), 2.58–2.49 (m, 1H, CH_2_), 2.39 (s, 3H, Me), 2.15 (s, 3H, Me), 2.06–1.97 (m, 1H, CH_2_), 1.96–1.88 (m, 1H, CH_2_), 1.80–1.66 (m, 2H, CH_2_), 1.56 (s, 9H, Me). ^13^C{^1^H} NMR (126 MHz, CDCl_3_): δ 209.1, 150.7, 136.0, 133.8, 132.2, 127.2, 123.8, 118.9, 117.8, 116.1, 83.8, 48.7, 28.4, 28.2, 27.3, 22.2, 21.1, 19.9. HRMS (ESI) calcd for C_20_H_26_NO_3_^1+^ (M^+^ + 1) requires, 328.1907; found, 328.1909.

#### *tert*-Butyl 1-Acetyl-7-fluoro-1,2,3,4-tetrahydro-9*H*-carbazole-9-carboxylate (**3t**)

Yield (0.1 mmol scale, **Green**: 20 mg, 60% yield; **Blue**: 24 mg, 73% yield). A white solid. *R*_f_ = 0.5 (Hexanes/EtOAc = 4/1). FC (Hexanes/EtOAc = 10/1). ^1^H NMR (500 MHz, CDCl_3_): δ 7.62 (dd, *J* = 8.5, 2.0 Hz, 1H, ArH), 7.25 (dd, *J* = 8.5, 5.5 Hz, 1H, ArH), 6.89 (ddd, *J* = 8.5, 8.5, 2.0 Hz, 1H, ArH), 4.29 (dd, *J* = 6.0, 5.0 Hz, 1H, CH), 2.64 (ddd, *J* = 16.5, 5.5, 5.0 Hz, 1H, CH_2_), 2.56–2.49 (m, 1H, CH_2_), 2.20 (s, 3H, Me), 2.06–1.97 (m, 1H, CH_2_), 1.96–1.89 (m, 1H, CH_2_), 1.80–1.68 (m, 2H, CH_2_), 1.56 (s, 9H, Me). ^13^C{^1^H} NMR (126 MHz, CDCl_3_): δ 208.69, 160.79 (d, *J* = 238.9 Hz), 150.36, 135.66 (d, *J* = 12.7 Hz), 133.18 (d, *J* = 3.8 Hz), 125.79, 118.72, 118.58 (d, *J* = 9.8 Hz), 110.44 (d, *J* = 23.9 Hz), 103.3 (d, *J* = 29.1 Hz), 84.39, 48.50, 28.39, 28.19, 26.91, 20.94, 19.68. ^19^F NMR (471 MHz, CDCl_3_): δ −118.05. HRMS (ESI) calcd for C_19_H_23_FNO_3_^1+^ (M^+^ + 1) requires, 332.1656; found, 332.1658.

#### *tert*-Butyl 1-Acetyl-7-bromo-1,2,3,4-tetrahydro-9*H*-carbazole-9-carboxylate (**3u**)

Yield (0.1 mmol scale, **Green**: 27 mg, 69% yield; **Blue**: 32 mg, 82% yield). A white solid. *R*_f_ = 0.5 (Hexanes/EtOAc = 4/1). FC (Hexanes/EtOAc = 10/1). ^1^H NMR (500 MHz, CDCl_3_): δ 8.10 (d, *J* = 1.5 Hz, 1H, ArH), 7.25 (dd, *J* = 8.5, 1.5 Hz, 1H, ArH), 7.20 (d, *J* = 8.5, 1H, ArH), 4.28 (dd, *J* = 6.5, 5.0 Hz, 1H, CH), 2.64 (ddd, *J* = 17.0, 5.5, 5.0 Hz, 1H, CH_2_), 2.56–2.47 (m, 1H, CH_2_), 2.20 (s, 3H, Me), 2.08–1.98 (m, 1H, CH_2_), 1.98–1.90 (m, 1H, CH_2_), 1.79–1.69 (m, 2H, CH_2_), 1.57 (s, 9H, Me). ^13^C{^1^H} NMR (126 MHz, CDCl_3_): δ 208.4, 150.2, 136.2, 133.6, 128.3, 125.6, 119.2, 119.0, 118.8, 117.6, 84.6, 48.5, 28.4, 28.2, 26.9, 20.9, 19.6. HRMS (ESI) calcd for C_19_H_22_BrNaNO_3_^1+^ (M^+^+Na) requires, 414.0675; found, 414.0676.

#### *tert*-Butyl 1-Acetyl-8-methyl-1,2,3,4-tetrahydro-9*H*-carbazole-9-carboxylate (**3v**)

Yield (0.1 mmol scale, **Green**: 20 mg, 61% yield; **Blue**: 24 mg, 73% yield). A colorless oily solid. *R*_f_ = 0.5 (Hexanes/EtOAc = 4/1). FC (Hexanes/EtOAc = 10/1). ^1^H NMR (500 MHz, CDCl_3_): δ 7.21 (d, *J* = 7.5 Hz, 1H, ArH), 7.07 (dd, *J* = 7.5, 7.5 Hz, 1H, ArH), 6.99 (d, *J* = 7.5 Hz, 1H, ArH), 4.06 (dd, *J* = 5.5, 5.5 Hz, 1H, CH), 2.67 (ddd, *J* = 16.0, 5.5, 4.5 Hz, 1H, CH_2_), 2.59–2.50 (m, 1H, CH_2_), 2.41 (s, 3H, Me), 2.16 (s, 3H, Me), 2.06–1.99 (m, 2H, CH_2_), 1.83–1.75 (m, 1H, CH_2_), 1.73–1.61 (m, 1H, CH_2_), 1.50 (s, 9H, Me). ^13^C{^1^H} NMR (126 MHz, CDCl_3_): δ 209.4, 151.1, 135.5, 133.5, 130.4, 127.0, 124.7, 122.7, 118.0, 115.9, 84.4, 48.4, 28.7, 27.9, 27.5, 21.2, 21.1, 19.9. HRMS (ESI) calcd for C_20_H_26_NO_3_^1+^ (M^+^ + 1) requires, 328.1907; found, 328.1906.

#### *tert*-Butyl 1-Acetyl-8-fluoro-1,2,3,4-tetrahydro-9*H*-carbazole-9-carboxylate (**3w**)

Yield (0.1 mmol scale, **Green**: 17 mg, 51% yield; **Blue**: 20 mg, 60% yield). A white solid. *R*_f_ = 0.5 (Hexanes/EtOAc = 4/1). FC (Hexanes/EtOAc = 10/1). ^1^H NMR (500 MHz, CDCl_3_): δ 7.12 (dd, *J* = 7.5, 1.0 Hz, 1H, ArH), 7.06 (ddd, *J* = 7.5, 7.5, 4.0 Hz, 1H, ArH), 6.88 (ddd, *J* = 12.5, 7.5, 1.0 Hz, 1H, ArH), 4.24 (dd, *J* = 6.5, 5.0 Hz, 1H, CH), 2.65 (ddd, *J* = 17.0, 5.5, 5.0 Hz, 1H, CH_2_), 2.58–2.48 (m, 1H, CH_2_), 2.23 (s, 3H, Me), 2.08–2.00 (m, 1H, CH_2_), 1.99–1.91 (m, 1H, CH_2_), 1.81–1.68 (m, 2H, CH_2_), 1.50 (s, 9H, Me). ^13^C{^1^H} NMR (126 MHz, CDCl_3_): δ 208.5, 150.3, 150.1 (d, *J* = 252.1 Hz), 134.4, 133.2 (d, *J* = 3.3 Hz), 123.2 (d, *J* = 7.4 Hz), 122.6 (d, *J* = 8.8 Hz), 118.4 (d, *J* = 1.9 Hz), 114.0 (d, *J* = 3.5 Hz), 111.1 (d, *J* = 22.2 Hz), 84.0, 48.0, 28.5, 27.7, 26.8, 21.1, 19.8. ^19^F NMR (471 MHz, CDCl_3_): δ −117.00. HRMS (ESI) calcd for C_19_H_23_FNO_3_^1+^ (M^+^ + 1) requires, 332.1656; found, 332.1651.

#### *tert*-Butyl 1-Acetyl-3-methyl-1,2,3,4-tetrahydro-9*H*-carbazole-9-carboxylate (**3x**)

Yield (0.1 mmol scale; **Green**: 25 mg, 76% yield, d.r. = 4.0/1; **Blue**: 28 mg, 86% yield, d.r. = 3.9/1). A white solid. *R*_f_ = 0.5 (Hexanes/EtOAc = 4/1). FC (Hexanes/EtOAc = 10/1). ^1^H NMR (500 MHz, CDCl_3_): δ 7.90 (d, *J* = 8.0 Hz, 1H, ArH), 7.35 (d, *J* = 7.0 Hz, 1H, ArH), 7.22–7.16 (m, 1H, ArH), 7.14 (ddd, *J* = 7.5, 7.0, 1.0 Hz, 1H, ArH), 4.31–4.22 (m, 1H, CH), 2.82–2.67 (m, 1H, CH_2_), 2.17 (m, 5H, Me, CH_2_), 1.9–1.80 (m, 1H, CH), 1.56 (s, 9H, Me), 1.30–1.22 (m, 1H, CH_2_), 1.09 (d, *J* = 6.5 Hz, 3H, Me). ^13^C{^1^H} NMR (126 MHz, CDCl_3_): δ 208.6, 150.9, 135.8, 133.0, 129.2, 124.1, 122.5, 119.5, 118.2, 115.6, 84.1, 50.4, 36.4, 29.6, 29.0, 28.2, 27.6, 21.7. HRMS (ESI) calcd for C_20_H_26_NO_4_^1+^ (M^+^ + 1) requires, 328.1907; found, 328.1904.

#### *tert*-Butyl 6-Cyano-7,8,9,10-tetrahydrocyclohepta[*b*]indole-5(6*H*)-carboxylate (**4a**)

Yield (0.1 mmol scale, **Green**: 16 mg, 52% yield, **Blue**: 22 mg, 71% yield). A colorless oily solid. *R*_f_ = 0.5 (Hexanes/EtOAc = 4/1). FC (Hexanes/EtOAc = 10/1). ^1^H NMR (500 MHz, CDCl_3_): δ 7.96 (d, *J* = 8.5 Hz, 1H, ArH), 7.41 (d, *J* = 7.0 Hz, 1H, ArH), 7.24 (ddd, *J* = 8.5, 7.0, 1.5 Hz, 1H, ArH), 7.18 (ddd, *J* = 7.0, 7.0, 1.5 Hz, 1H, ArH), 5.29 (dd, *J* = 6.0, 2.5 Hz, 1H, CH), 2.85 (dd, *J* = 16.0, 6.5 Hz, 1H, CH_2_), 2.80–2.71 (m, 1H, CH_2_), 2.27–2.18 (m, 1H, CH_2_), 2.18–2.08 (m, 1H, CH_2_), 2.07–1.97 (m, 2H, CH_2_), 1.93–1.83 (m, 1H, CH_2_), 1.64 (s, 9H, Me), 1.59–1.54 (m, 1H, CH_2_). ^13^C{^1^H} NMR (126 MHz, CDCl_3_): δ 149.6, 134.3, 130.0, 128.2, 123.9, 123.7, 121.8, 118.0, 117.4, 114.7, 83.9, 28.5, 28.4, 27.2, 25.5, 25.2, 21.7. HRMS (ESI) calcd for C_19_H_23_N_2_O_2_^1+^ (M^+^ + 1) requires, 311.1754; found, 311.1757.

#### *tert*-Butyl 3-Formyl-1*H*-indole-1-carboxylate (**4c**)

Yield (0.1 mmol scale, **Green**: 7 mg, 29% yield; **Blue**: 6 mg, 24% yield). A light yellow solid. *R*_f_ = 0.4 (Hexanes/EtOAc = 4/1). FC (Hexanes/EtOAc = 10/1). ^1^H NMR (500 MHz, CDCl_3_): δ 10.02 (s, 1H, CHO), 8.21 (d, *J* = 7.5, 1.5 Hz, 1H, ArH), 8.15 (s, 1H, ArH), 8.07 (d, *J* = 8.0 Hz, 1H, ArH), 7.34 (ddd, *J* = 8.0, 7.5, 1.5 Hz, 1H, ArH), 7.29 (ddd, *J* = 7.5, 7.5, 1.5 Hz, 1H, ArH), 1.64 (s, 9H, Me). ^13^C{^1^H} NMR (126 MHz, CDCl_3_): δ 185.8, 148.8, 136.5, 136.0, 126.1, 126.1, 124.6, 122.2, 121.6, 115.2, 85.7, 28.1.

### Experimental Procedure for the Gram-Scale Reaction

In a Ar glovebox, the substrate **1** (1.74 g, 4.0 mmol, 1.0 equiv) and [Ru(bpy)_3_Cl_2_]·6H_2_O (0.2 mmol, 5 mol %) were added to an oven-dried (overnight) Schlenk flask containing a stirring bar, followed by adding anhydrous DMSO (20.0 mL) and **2** (701 mg, 10.0 mmol, 2.5 equiv). The Schlenk flask was then sealed, removed from the glovebox, and the mixture was stirred at room temperature under Blue LED (λ_max_ = 440 nm) irradiation for 36 h. Water (30 mL) was added to the reaction mixture, which was then extracted with EtOAc (3x, 30 mL each). The combined organic layer was washed with water, brine and dried over anhydrous Na_2_SO_4_. After concentrating under reduced pressure on a RotaVap, the crude product was purified by flash column chromatography (FC) on silica gel (eluent: Hexanes/EtOAc = 12/1) to provide the desired product **3a** as a colorless solid (1.05 g, 84% yield).

### Experimental Procedure for the Transformation of **3a**

To a solution of **3a** (125 mg, 0.4 mmol, 1.0 equiv) and CeCl_3_·7H_2_O (149 mg, 0.4 mmol, 1.0 equiv) in MeOH (2.0 mL) was added NaBH_4_ (23 mg, 0.6 mmol, 1.5 equiv) portionwisely at 0 °C. The mixture was warmed to room temperature and stirred for 18 h. The reaction was quenched with 3 drops of 10% aq. HCl, which was followed by the addition of water. The solution was then extracted with extracted with EtOAc (3×). The combined organic layer was washed with brine and dried over anhydrous Na_2_SO_4_. After concentrating under reduced pressure on a RotaVap, the crude product was purified by flash column chromatography (FC) on silica gel (eluent: Hexanes/EtOAc = 10/1–8/1) to provide the desired product **5** as a colorless oily solid (82 mg, 64% yield, d.r. = 1.1/1).

#### *tert*-Butyl 1-(1-Hydroxyethyl)-1,2,3,4-tetrahydro-9*H*-carbazole-9-carboxylate (**5**)

Yield (0.4 mmol scale, 82 mg, 65% yield, d.r. = 1.1/1). A colorless oily solid. *R*_f_ = 0.5 (Hexanes/EtOAc = 3/1). FC (Hexanes/EtOAc = 10/1–8/1). ^1^H NMR (500 MHz, CDCl_3_): δ 7.98 (d, *J* = 7.5 Hz, 1H, ArH), 7.32 (dd, *J* = 11.0, 1.5 Hz, 1H, ArH), 7.22–7.08 (m, 2H, ArH), 4.20 (qd, *J* = 6.5, 3.0 Hz, 1H, CH), 3.56–3.47 (m, 1H, CH), 2.75–2.47 (m, 2H, CH_2_), 2.34–1.95 (m, 2H, CH_2_, OH), 1.89–1.66 (m, 3H, CH_2_), 1.59 (s, 9H, Me), 1.20 (d, *J* = 6.5 Hz, 3H, Me). ^13^C{^1^H} NMR (126 MHz, CDCl_3_): δ 151.0, 137.1, 135.8, 129.6, 124.0, 122.6, 118.0, 117.6, 115.7, 83.7, 40.1, 28.3, 23.9, 20.9, 20.4, 19.7. HRMS (ESI) calcd for C_19_H_26_NO_3_^1+^ (M^+^ + 1) requires, 316.1907; found, 316.1909.

### Experimental Procedure for the Radical Trapping Experiments

In an Ar glovebox, **1a** (0.1 mmol, 1.0 equiv) and [Ru(bpy)_3_Cl_2_]·6H_2_O (0.005 mmol, 5 mol % or 0.1 mmol, 1 equiv) were added to an oven-dried (overnight) Schlenk tube containing a stirring bar, followed by adding anhydrous DMSO (1.0 mL), **2a** (0.2 mmol, 2.0 equiv) and TEMPO (0.5 mmol, 5.0 equiv) The Schlenk tube was then sealed, removed from the glovebox, and the mixture was stirred at room temperature under Blue LED (λ_max_ = 440 nm) irradiation. After 18 h, the reaction mixture was monitored by TLC. The crude product was purified by flash chromatography (FC) on silica gel (eluent: Hexanes/EtOAc = 40/1) to yield the desired product **6**.

#### *tert*-Butyl 3-(2-((2,2,6,6-Tetramethylpiperidin-1-yl)oxy)ethyl)-1*H*-indole-1-carboxylate (**6**)

Yield (0.1 mmol scale, 10 mg, 25% yield). A colorless oil. *R*_f_ = 0.5 (Hexanes/EtOAc = 10/1). FC (Hexanes/EtOAc = 40/1). ^1^H NMR (500 MHz, CDCl_3_): δ 8.07–8.05 (m, 1H, ArH), 7.48 (d, *J* = 8.0 Hz, 1H, ArH), 7.36 (s, 1H, ArH), 7.23 (ddd, *J* = 8.5, 7.0, 1.5 Hz, 1H, ArH), 7.16 (ddd, *J* = 7.5, 7.5, 1.5 Hz, 1H, ArH), 3.97 (t, *J* = 7.0 Hz, 2H, CH_2_), 2.84 (t, *J* = 7.0 Hz, 2H, CH_2_), 1.59 (s, 9H, Me), 1.39–1.36 (m, 4H, CH_2_), 1.29–1.19 (m, 2H, CH_2_), 1.08 (s, 6H, Me), 1.04 (s, 6H, Me). ^13^C{^1^H} NMR (126 MHz, CDCl_3_): δ 149.8, 135.4, 130.9, 124.2, 123.1, 122.3, 119.1, 118.2, 115.1, 83.2, 76.1, 59.7, 39.6, 33.1, 28.3, 24.3, 20.2, 17.2. HRMS (ESI) calcd for C_24_H_36_N_2_O_3_^1+^ (M^+^ + 1) requires, 401.2799; found, 401.2803.

### Experimental Procedure for the Blue Light/Dark Interval Experiment

In a Ar glovebox, **1a** (0.05 mmol, 1.0 equiv) and [Ru(bpy)_3_Cl_2_]·6H_2_O (0.0025 mmol, 5 mol %) were added to an oven-dried (overnight) NMR tube containing a stirring bar, followed by adding degassed DMSO-*d*_6_ (1.0 mL), **2a** (0.1 mmol, 2.0 equiv) and trimethoxybenzene (0.05 mmol, 1.0 equiv). The NMR tube was then sealed, removed from the glovebox, and the mixture was stirred at room temperature under Blue LED (λ_max_ = 440 nm) irradiation or in dark. After the indicated time, the reaction mixture was monitored by ^1^H NMR to determine the yield of **3a** directly.

## Supporting Information Available

Experimental procedures including substrate synthesis, background and control experiments, characterization data, and NMR spectra as well as the spectrum graph for KSPR160L-440 nm, Blue LED, Kessil LED Lights & KSPR160L-525 nm, Green LED, Kessil LED Lights (PDF)
