# Supplementary material for: Visible-Light-Mediated Rose Bengal- or [Ru(bpy)3]2+-Catalyzed Radical [4 + 2] Cycloaddition: An Efficient Route to Tetrahydrocarbazoles
Source: ACS Omega. 2025 Mar 7;10(10):10713–23. doi: 10.1021/acsomega.5c00416 (PMC11923644; doi:10.1021/acsomega.5c00416)
Supplement: Supplementary file 1 — ao5c00416_si_001.pdf [file ao5c00416_si_001.pdf]

Supporting Information

Visible-light-mediated Rose Bengal- or $[Ru(bpy)_3]^{2+}$-catalyzed radical [4+2] cycloaddition: an efficient route to tetrahydrocarbazoles

*Cody Bishir, Abbey Hubbard, and Liangyong Mei**

Department of Chemistry and Biochemistry, University of North Florida, Jacksonville, Florida 32224, United States
l.mei@unf.edu

CONTENTS

1. Spectrum graphs for KSPR160L-440 Blue LED (440 nm) & KSPR160L-525 Green LED (525 nm)……………………………………..…….……………………………..………...............S2
2. Reaction setup for photocatalysis ……….…….……………………………………..…..….......S3
3. General procedure for the synthesis of redox-active indole N-hydroxyphthalimide esters **1**….S4
4. Unsucessful substrates for photo-mediated radical [4+2] cycloaddtion.……………............S13
5. Blue light/dark interval experiment ………………………...……………..……………..……...S14
6. Alternative mechanism for $[Ru(bpy)_3]^{2+}$-catalyzed radical [4+2] cycloaddition ……….…...S15
7. NMR spectra charts for compounds **1**& **3**-**6**…………….……..………………..………...…..S16
8. References……….……..…………………………………………………..………...…..S66

1. Spectrum graphs for KSPR160L-440 Blue LED (440 nm) & KSPR160L-525 Green LED (525 nm)

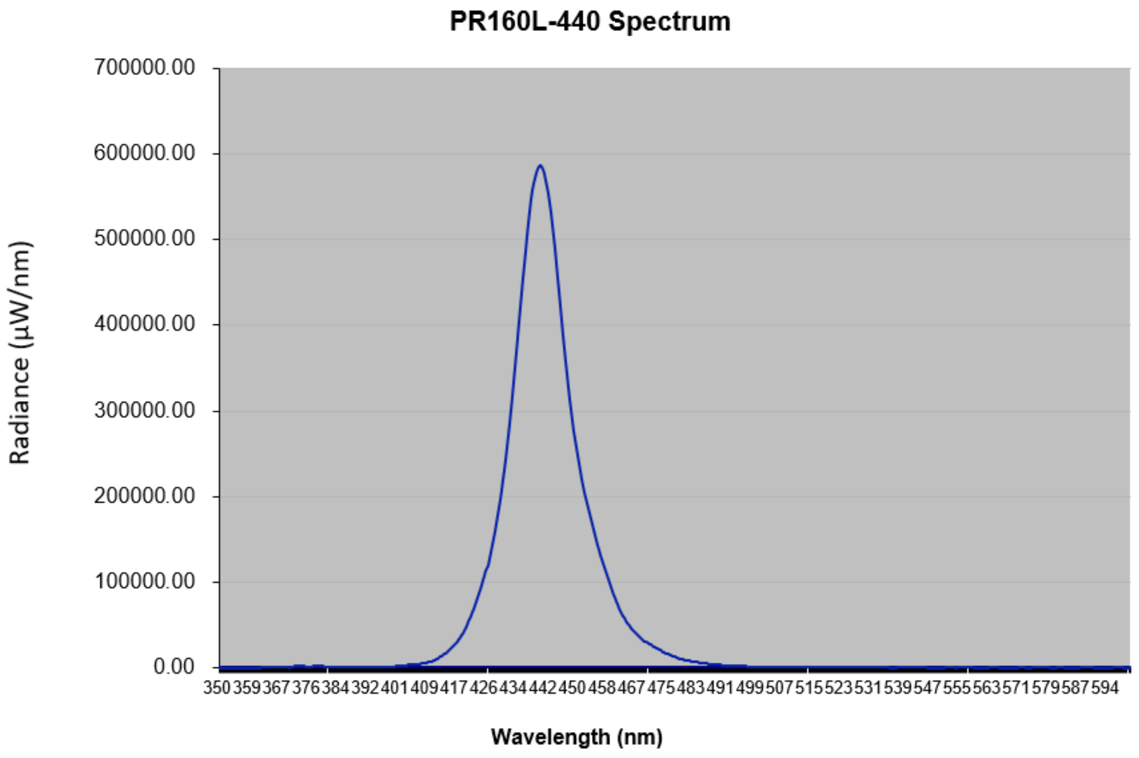

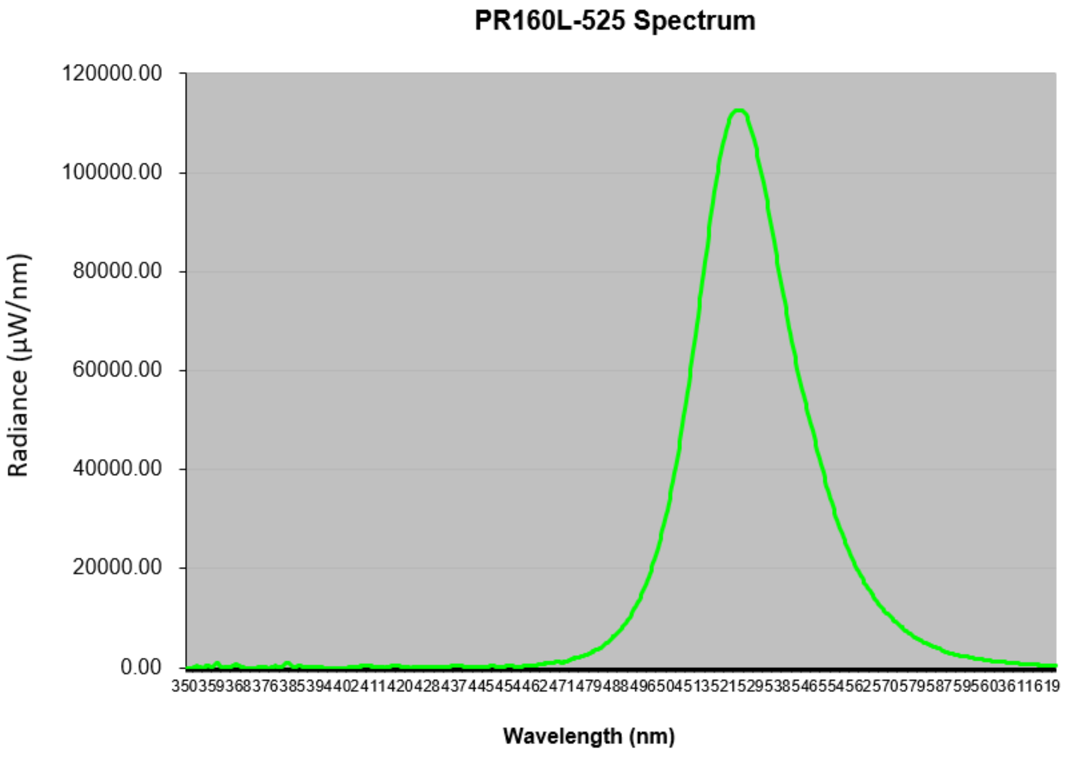

2. Reaction setup for photocatalysis

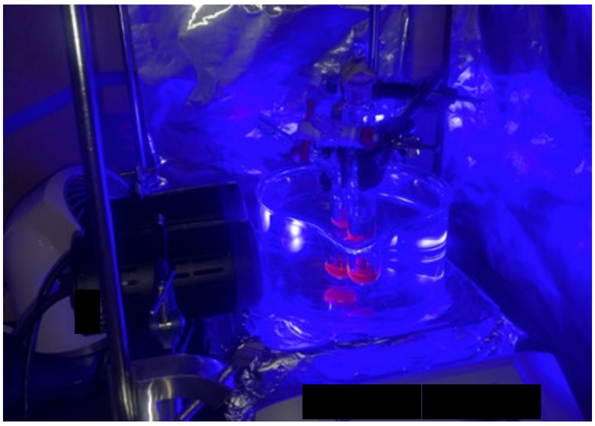

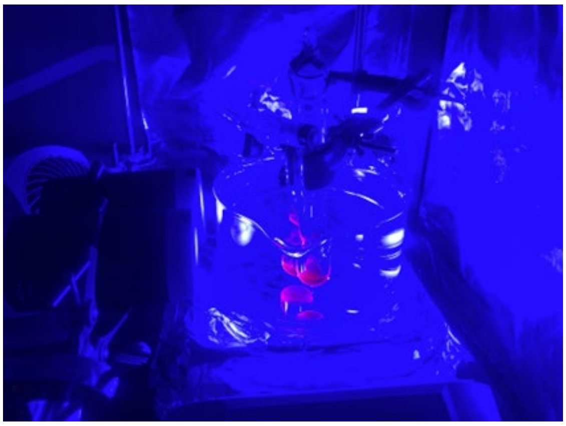

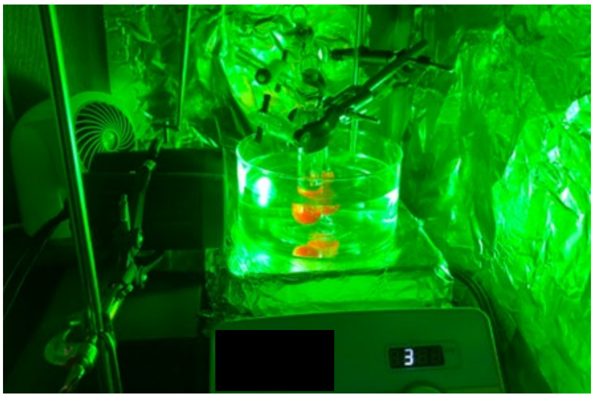

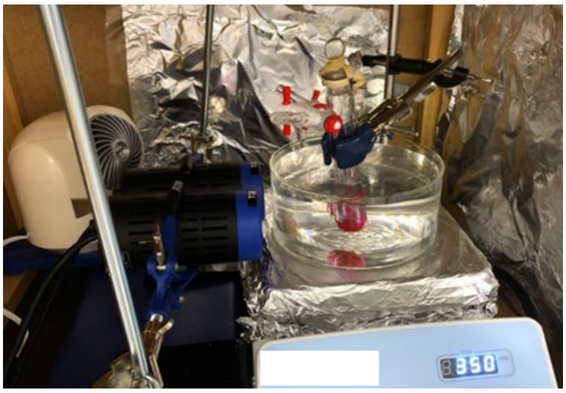

3. General procedure for the synthesis of redox-active indole N-hydroxyphthalimide esters 1

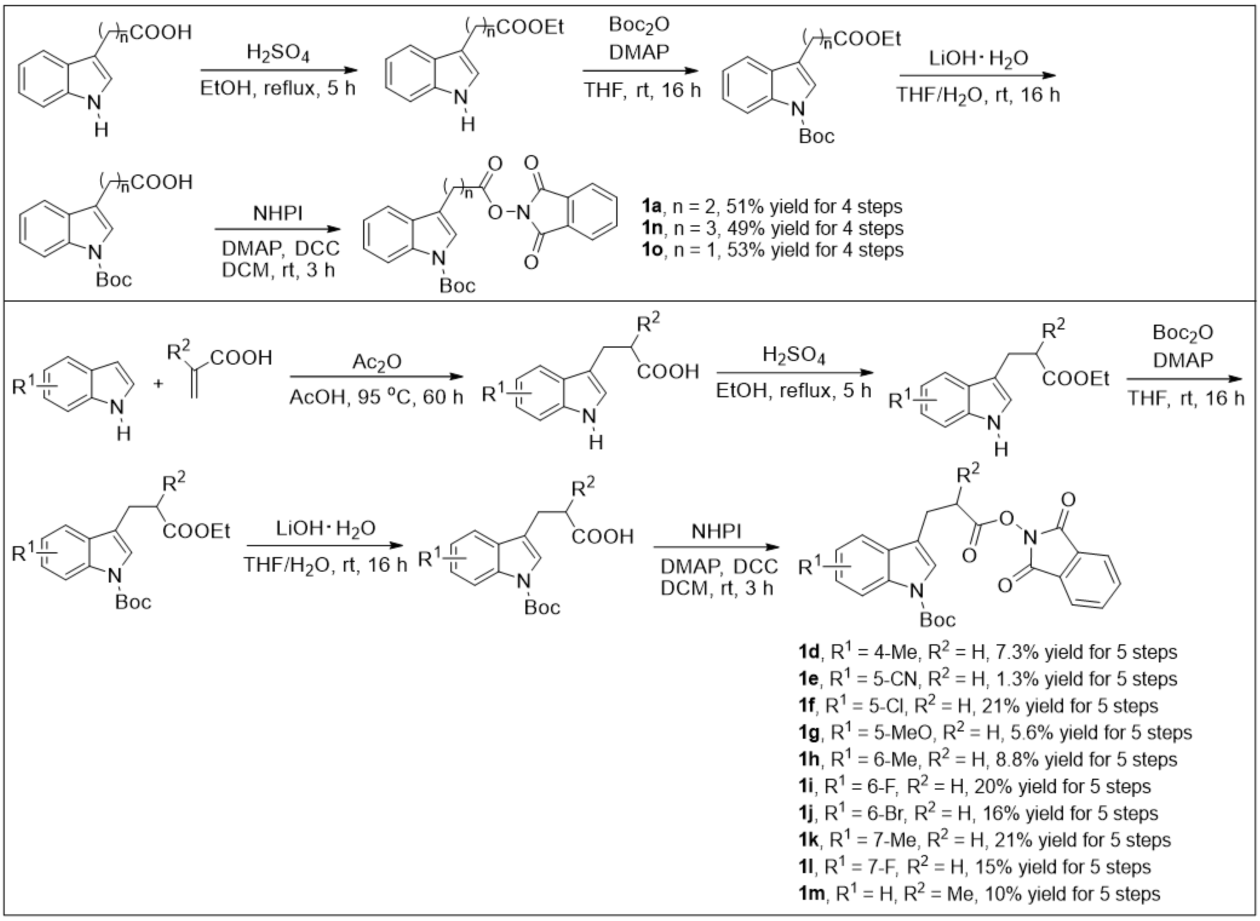

Following the modified reported procedure.[1,2]

To a solution of indole (1.0 eq.) in AcOH (0.6 mL/mol) in a round-bottom flask was added acrylic acid (2.4 eq.) and Ac_2O (2.1 eq.) at rt. The mixture was stirred at 95 °C for 60 h, which was then cooled to rt, quenched with 4 M aq. NaOH to pH ~ 7, and extracted with ethyl acetate (EtOAc). The aqueous layer was then acidified to pH ~ 2 with 6 M aq. HCl and extracted with EtOAc (3x). The combined organic layer was washed with brine and dried over anhydrous Na_2SO_4. After concentrating under reduced pressure on a RotaVap, the crude product was purified by flash column chromatography (FC) on silica gel (eluent: Hexanes/EtOAc = 5/1 ~ 1/1) to provide the product.

To a solution of the above carboxylic acid (1.0 eq.) in ethanol (0.1 M) was added concentrated sulfuric acid (1 drop/mmol of carboxylic acid). After stirring under reflux for 5 h, the reaction mixture was cooled to rt. After removing the ethanol solvent on a RotaVap, EtOAc was added to dissolve the crude product. Then sat. aq. $NaHCO_3$ was added to quenched H_2SO_4, which was followed by addition of water and extraction with EtOAc (3x). The combined organic layer was washed with brine, dried over anhydrous Na_2SO_4. After concentrating under reduced pressure on a RotaVap, the crude product was used directly in the next step without further purification.

To a solution of the above crude ethyl ester (1.0 eq.) in THF (0.2 M) was added DMAP (6 mol%) and di-tert-butyl dicarbonate (1.2 eq.) at rt. After stirring for 16 h, the reaction mixture was quenched with sat. aq. $NaHCO_3$, water was added, and extracted with EtOAc (3x). The combined organic layer was washed with brine and dried over anhydrous Na_2SO_4. After concentrating under reduced

pressure on a RotaVap, the crude product was used directly in the next step without further purification.

To a solution of the above crude N-Boc indole in THF/H_2O (3:1, 0.25 M) was added lithium hydroxide (2.3 eq.) at room temperature. After stirring for 10 h, the reaction mixture was acidified to pH 2~3 with 10% aq. HCl and extracted with EtOAc (3x). The combined organic layer was washed with H_2O and dried over anhydrous Na_2SO_4. After concentrating under reduced pressure on a RotaVap, the crude product was purified by flash column chromatography (FC) on silica gel (eluent: Hexanes/EtOAc = 5/1 ~ 1/1) to yield the desired carboxylic acid precusor.

To a solution of the above carboxylic acid precusor (1.0 eq.) in DCM (0.2 M) was added N-hydroxyphthalimide (NHPI) (1.2 eq.), DMAP (10 mmol%) and DCC (1.2 eq.) at room temperature. After stirring for 3 h, the precipitates in the reaction mixture were filtered through celite and the filtrate was concentrated under reduced pressure on a RotaVap. The crude product was purified by flash column chromatography (FC) on silica gel (eluent: Hexanes/EtOAc = 6/1 ~ 4/1) to yield the desired redox-active indole N-hydroxyphthalimide esters **1**.

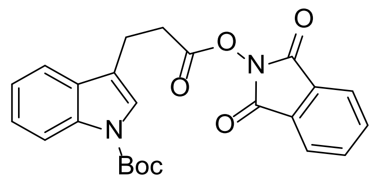

1a

*tert-butyl 3-(3-((1,3-dioxoisoindolin-2-yl)oxy)-3-oxopropyl)-1H-indole-1-carboxylate (**1a**).* Yield (24.0 mmol scale, 5.32 g, 51% yield for 4 steps). A white solid. R_f = 0.4 (Hexanes/EtOAc = 2/1). FC (Hexanes/EtOAc = 6/1). ^{1}H NMR (500 MHz, $CDCl_3$) δ 8.07 (d, J = 8.0 Hz, 1H, ArH), 7.82-7.78 (m, 2H, ArH), 7.72-7.69 (m, 2H, ArH), 7.47 (d, J = 8.0 Hz, 1H, ArH), 7.42 (s, 1H, ArH), 7.28-7.22 (m, 1H, ArH), 7.20-7.16 (m, 1H, ArH), 3.11 (t, J = 7.0 Hz, 2H, CH_2), 3.00 (t, J = 7.0 Hz, 2H, CH_2), 1.60 (s, 9H, Me). $^{13}C\{^1H\}$ NMR (126 MHz, $CDCl_3$) δ 169.0, 161.9, 149.7, 135.6, 134.8, 129.9, 128.9, 124.6, 124.0, 123.1, 122.6, 118.6, 118.1, 115.4, 83.6, 31.0, 28.2, 20.2. HRMS (ESI) Calcd. for $C_{24}H_{23}N_2O_6^{+1}$ (M^++1) requires 435.1551; Found: 435.1559.

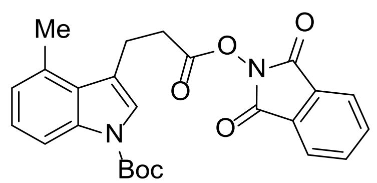

1d

*tert-butyl 3-(3-((1,3-dioxoisoindolin-2-yl)oxy)-3-oxopropyl)-4-methyl-1H-indole-1-carboxylate (**1d**).* Yield (10.0 mmol scale, 327 mg, 7.3% yield for 5 steps). A white solid. R_f = 0.4 (Hexanes/EtOAc = 2/1). FC (Hexanes/EtOAc = 6/1). ^{1}H NMR (500 MHz, $CDCl_3$) δ 1H NMR (500 MHz, CDCl3) δ 7.97 (d, J = 8.0 Hz, 1H, ArH), 7.83-7.80 (m, 2H, ArH), 7.73-7.70 (m, 2H, ArH), 7.35 (s, 1H, ArH), 7.12 (dd, J = 8.5, 7.0 Hz, 1H, ArH), 6.91 (d, J = 7.0 Hz, 1H, ArH), 3.29 (t, J = 7.5 Hz, 2H, CH_2), 3.00 (t, J = 7.5 Hz, 2H, CH_2), 2.62 (s, 3H, Me), 1.60 (s, 9H, Me). $^{13}C\{^1H\}$ NMR (126 MHz, $CDCl_3$) δ 168.9, 161.9, 149.6, 136.2, 134.8, 130.6, 128.9, 128.3, 124.6, 124.5, 124.0, 123.1, 119.1, 113.2, 83.5, 31.7, 28.2, 22.5, 20.1. HRMS (ESI) Calcd. for $C_{25}H_{25}N_2O_6^{+1}$ (M^++1) requires 449.1707; Found: 449.1713.

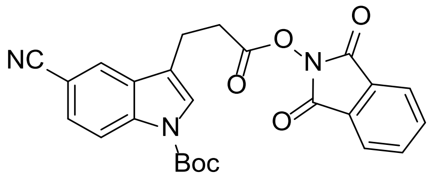

1e

tert-butyl 5-cyano-3-(3-((1,3-dioxoisoindolin-2-yl)oxy)-3-oxopropyl)-1H-indole-1-carboxylate ***(1e)****.* Yield (30.0 mmol scale, 179 mg, 1.3% yield for 5 steps). A white solid. R_f = 0.3 (Hexanes/EtOAc = 2/1). FC (Hexanes/EtOAc = 6/1 ~ 4/1). ^{1}H NMR (500 MHz, $CDCl_3$) δ 8.19 (d, *J* = 9.0 Hz, 1H, ArH), 7.85-7.79 (m, 3H, ArH), 7.74-7.72 (m, 2H, ArH), 7.55 (s, 1H, ArH), 7.51 (dd, *J* = 9.0, 1.5 Hz, 1H, ArH), 3.13 (t, *J* = 7.5 Hz, 2H, CH_2), 3.01 (t, *J* = 7.5 Hz, 2H, CH_2), 1.62 (s, 9H, Me). $^{13}C\{^1H\}$ NMR (126 MHz, $CDCl_3$) δ 168.7, 161.8, 149.0, 137.5, 134.9, 130.0, 128.9, 127.8, 125.4, 124.0, 123.6, 119.7, 117.8, 116.3, 106.0, 84.9, 30.9, 28.1, 19.8. HRMS (ESI) Calcd. for $C_{25}H_{22}N_3O_6^{+1}$ (M^++1) requires 460.1503; Found: 460.1509.

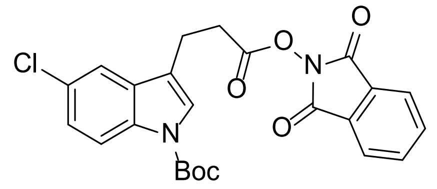

1f

tert-butyl 5-chloro-3-(3-((1,3-dioxoisoindolin-2-yl)oxy)-3-oxopropyl)-1H-indole-1-carboxylate ***(1f)****.* Yield (10.0 mmol scale, 985 mg, 21% yield for 5 steps). A white solid. R_f = 0.4 (Hexanes/EtOAc = 2/1). FC (Hexanes/EtOAc = 6/1). ^{1}H NMR (500 MHz, $CDCl_3$) δ 8.00 (d, *J* = 7.0 Hz, 1H, ArH), 7.83-7.80 (m, 2H, ArH), 7.74-7.71 (m, 2H, ArH), 7.45-7.40 (m, 2H, ArH), 7.21 (dd, *J* = 8.5, 2.0 Hz, 1H, ArH), 3.07 (t, *J* = 7.0 Hz, 2H, CH_2), 2.99 (t, *J* = 7.0 Hz, 2H, CH_2), 1.60 (s, 9H, Me). $^{13}C\{^1H\}$ NMR (126 MHz, $CDCl_3$) δ 168.8, 161.8, 149.3, 134.8, 134.0, 131.1, 128.9, 128.4, 124.7, 124.4, 124.0, 118.3, 117.4, 116.5, 84.0, 30.9, 28.2, 20.0. HRMS (ESI) Calcd. for $C_{24}H_{22}ClN_2O_6^{+1}$ (M^++1) requires 469.1161; Found: 469.1162.

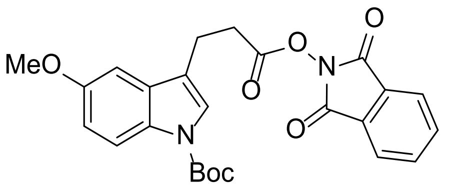

1g

tert-butyl 3-(3-((1,3-dioxoisoindolin-2-yl)oxy)-3-oxopropyl)-5-methoxy-1H-indole-1-carboxylate ***(1g)****.* Yield (20.0 mmol scale, 520 mg, 5.6% yield for 5 steps). A white solid. Rf = 0.3 (Hexanes/EtOAc = 2/1). FC (Hexanes/EtOAc = 6/1 ~ 4/1). ^{1}H NMR (500 MHz, $CDCl_3$) δ 7.95 (bs, 1H, ArH), 7.83-7.80 (m, 2H, ArH), 7.73-7.70 (m, 2H, ArH), 7.39 (s, 1H, ArH), 6.90 (d, *J* = 2.5 Hz, 1H, ArH), 6.87 (dd, *J* = 8.5, 2.5 Hz, 1H, ArH), 3.81 (s, 3H, OMe), 3.09-3.05 (m, 2H, CH_2), 3.02-2.97 (m, 2H, CH_2), 1.59 (s, 9H, Me). $^{13}C\{^1H\}$ NMR (126 MHz, $CDCl_3$) δ 169.0, 161.9, 155.9, 149.6, 134.8, 130.7, 130.3, 128.9, 124.0, 123.6, 117.9, 116.2, 113.2, 101.4, 83.4, 55.8, 30.9, 28.2, 20.1. HRMS (ESI) Calcd. for $C_{25}H_{25}N_2O_7^{+1}$ (M^++1) requires 465.1656; Found: 465.1657.

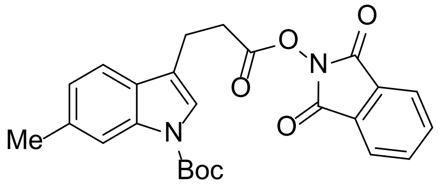

1h

*tert-butyl 3-(3-((1,3-dioxoisoindolin-2-yl)oxy)-3-oxopropyl)-6-methyl-1H-indole-1-carboxylate (**1h**)*. Yield (10.0 mmol scale, 395 mg, 8.8% yield for 5 steps). A white solid. R_f = 0.4 (Hexanes/EtOAc = 2/1). FC (Hexanes/EtOAc = 6/1). ^{1}H NMR (500 MHz, $CDCl_3$) δ 8.04 (bs, 1H, ArH), 7.92-7.90 (m, 2H, ArH), 7.83-7.80 (m, 2H, ArH), 7.46-7.39 (m, 2H, ArH), 7.12 (d, *J* = 8.0 Hz, 1H, ArH), 3.19 (t, *J* = 7.0 Hz, 2H, CH_2), 3.09 (t, *J* = 7.60 Hz, 2H, CH_2), 2.51 (s, 3H, Me), 1.70 (s, 9H, Me). ^{13}C{^{1}H} NMR (126 MHz, $CDCl_3$) δ 169.0, 161.9, 149.8, 136.1, 134.8, 134.7, 128.9, 127.6, 124.0, 122.4, 118.2, 118.1, 115.7, 83.4, 31.0, 28.2, 22.0, 20.2. HRMS (ESI) Calcd. for $C_{25}H_{25}N_2O_6^{+1}$ (M^++1) requires 449.1707; Found: 449.1714.

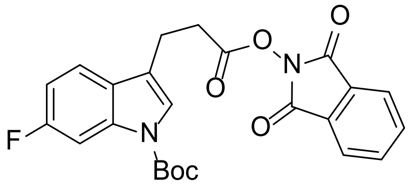

1i

*tert-butyl 3-(3-((1,3-dioxoisoindolin-2-yl)oxy)-3-oxopropyl)-6-fluoro-1H-indole-1-carboxylate (**1i**)*. Yield (10.0 mmol scale, 905 mg, 20% yield for 5 steps). A white solid. R_f = 0.4 (Hexanes/EtOAc = 2/1). FC (Hexanes/EtOAc = 6/1). ^{1}H NMR (500 MHz, $CDCl_3$) δ 7.82-7.77 (m, 3H, ArH), 7.73-7.69 (m, 2H, ArH), 7.39-7.35 (m, 2H, ArH), 6.93 (ddd, *J* = 9.0 Hz, 9.0 Hz, 2.5 Hz, 1H, ArH), 3.08 (t, *J* = 7.0 Hz, 2H, CH_2), 2.98 (t, *J* = 7.0 Hz, 2H, CH_2), 1.60 (s, 9H, Me). ^{13}C{^{1}H} NMR (126 MHz, $CDCl_3$) δ 168.9, 161.8, 161.1 (d, *J* = 240.7 Hz), 149.4, 135.8, 134.8, 128.9, 126.2, 124.0, 123.2 (d, *J* = 4.0 Hz), 119.3 (d, *J* = 10.1 Hz), 117.9, 110.8 (d, *J* = 24.4 Hz), 102.8 (d, *J* = 28.5 Hz), 84.0, 30.9, 28.2, 20.1. ^{19}F NMR (471 MHz, $CDCl_3$) δ -117.23. HRMS (ESI) Calcd. for $C_{24}H_{22}FN_2O_6^{+1}$ (M^++1) requires 453.1456; Found: 453.1464.

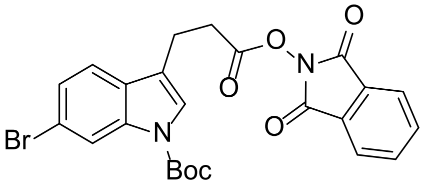

1j

*tert-butyl 6-bromo-3-(3-((1,3-dioxoisoindolin-2-yl)oxy)-3-oxopropyl)-1H-indole-1-carboxylate (**1j**)*. Yield (10.0 mmol scale, 821 mg, 16% yield for 5 steps). A white solid. R_f = 0.4 (Hexanes/EtOAc = 2/1). FC (Hexanes/EtOAc = 6/1). ^{1}H NMR (500 MHz, $CDCl_3$) δ 8.30 (bs, 1H, ArH), 7.83-7.80 (m, 2H, ArH), 7.74-7.71 (m, 2H, ArH), 7.39 (s, 1H, ArH), 7.35-7.27 (m, 2H, ArH), 3.09 (t, *J* = 7.0 Hz, 2H, CH_2), 2.98 (t, *J* = 7.0 Hz, 2H, CH_2), 1.61 (s, 9H, Me). ^{13}C{^{1}H} NMR (126 MHz, $CDCl_3$) δ 168.8, 161.8, 149.3, 136.3, 134.8, 128.9, 128.7, 125.8, 124.0, 123.5, 119.7, 118.6, 118.4, 117.9, 84.2,

30.9, 28.2, 19.99. HRMS (ESI) Calcd. for $C_{24}H_{22}BrN_2O_6^{+1}$ (M^++1) requires 513.0656; Found: 513.0658.

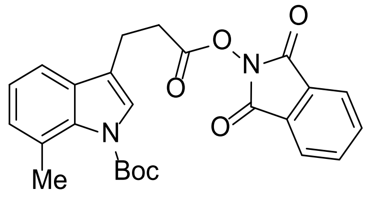

1k

*tert-butyl 3-(3-((1,3-dioxoisoindolin-2-yl)oxy)-3-oxopropyl)-7-methyl-1H-indole-1-carboxylate (**1k**)*. Yield (10.0 mmol scale, 942 mg, 21% yield for 5 steps). A white solid. R_f = 0.4 (Hexanes/EtOAc = 2/1). FC (Hexanes/EtOAc = 6/1). ^{1}H NMR (500 MHz, $CDCl_3$) δ 7.83-7.81 (m, 2H, ArH), 7.74-7.72 (m, 2H, ArH), 7.36 (s, 1H, ArH), 7.30 (d, J = 7.35 Hz, 1H, ArH), 7.12 (dd, J = 7.5 Hz, 7.5 Hz, 1H, ArH), 7.06 (d, J = 7.5 Hz, 1H, ArH), 3.09 (t, J = 6.5 Hz, 2H, CH_2), 3.00 (t, J = 6.5 Hz, 2H, CH_2), 2.56 (s, 3H, Me), 1.58 (s, 9H, Me). ^{13}C{^{1}H} NMR (126 MHz, $CDCl_3$) δ 169.03, 161.92, 149.49, 135.16, 134.84, 131.23, 128.90, 127.98, 125.74, 125.37, 124.04, 123.10, 117.75, 116.14, 83.28, 30.97, 28.09, 22.23, 20.13. HRMS (ESI) Calcd. for $C_{25}H_{24}N_2O_6Na^{+1}$ (M^++Na) requires 471.1527; Found: 471.1527.

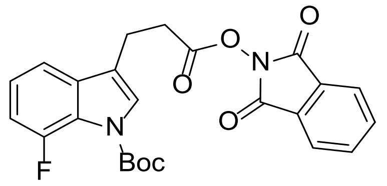

1l

*tert-butyl 3-(3-((1,3-dioxoisoindolin-2-yl)oxy)-3-oxopropyl)-7-fluoro-1H-indole-1-carboxylate (**1l**)*. Yield (10.0 mmol scale, 679 mg, 15% yield for 5 steps). A white solid. R_f = 0.4 (Hexanes/EtOAc = 2/1). FC (Hexanes/EtOAc = 6/1). ^{1}H NMR (500 MHz, $CDCl_3$) δ 7.83-7.80 (m, 2H, ArH), 7.73-7.71 (m, 2H, ArH), 7.47 (s, 1H, ArH), 7.23 (dd, J = 7.5, 1.0 Hz, 1H, ArH), 7.13 (ddd, J = 7.5 Hz, 7.5 Hz, 4.0 Hz, 1H, ArH), 6.97 (dd, J = 13.5, 7.5 Hz, 1H, ArH), 3.10 (t, J = 7.5 Hz, 2H, CH_2), 2.99 (t, J = 7.5 Hz, 2H, CH_2), 1.59 (s, 9H, Me). ^{13}C{^{1}H} NMR (126 MHz, $CDCl_3$) δ 168.85, 161.84, 150.16 (d, J = 253.6 Hz), 148.80, 134.80, 134.22 (d, J = 4.0 Hz), 128.92, 125.51, 124.01, 123.56 (d, J = 7.3 Hz), 122.30 (d, J = 9.7 Hz), 117.97 (d, J = 1.8 Hz), 114.40 (d, J = 3.8 Hz), 112.01 (d, J = 22.2 Hz), 84.10, 30.84, 28.00, 20.11. ^{19}F NMR (471 MHz, $CDCl_3$) δ -115.56. HRMS (ESI) Calcd. for $C_{24}H_{22}FN_2O_6^{+1}$ (M^++1) requires 453.1456; Found: 453.1461.

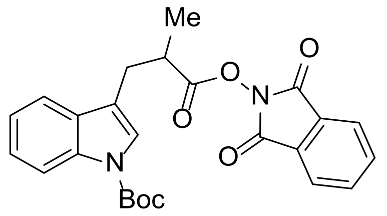

1m

*tert-butyl 3-(3-((1,3-dioxoisoindolin-2-yl)oxy)-2-methyl-3-oxopropyl)-1H-indole-1-carboxylate (**1m**)*. Yield (10.0 mmol scale, 448 mg, 10% yield for 5 steps). A colorless oily solid. R_f = 0.4 (Hexanes/EtOAc = 2/1). FC (Hexanes/EtOAc = 6/1). ^{1}H NMR (500 MHz, $CDCl_3$) δ 8.07 (d, J = 8.0

Hz, 1H, ArH), 7.79-7.76 (m, 2H, ArH), 7.69-7.66 (m, 2H, ArH), 7.49-7.46 (m, 2H, ArH), 7.24 (ddd, *J* = 8.0 Hz, 8.0 Hz, 1.0 Hz, 1H, ArH), 7.20-7.16 (m, 1H, ArH), 3.25 (dd, *J* = 14.5, 6.0 Hz, 1H, CH_2), 3.20-3.10 (m, 1H, CH), 2.86 (dd, *J* = 14.5, 7.0 Hz, 1H, CH_2), 1.60 (s, 9H, Me), 1.32 (d, *J* = 7.0 Hz, 3H, Me). $^{13}C\{^1H\}$ NMR (126 MHz, $CDCl_3$) δ 172.22, 161.90, 149.72, 135.59, 134.74, 130.30, 128.98, 124.49, 124.19, 123.92, 122.55, 118.77, 116.71, 115.39, 83.57, 37.52, 28.69, 28.23, 16.78. HRMS (ESI) Calcd. for $C_{25}H_{25}N_2O_6^{+1}$ (M^++1) requires 449.1707; Found: 449.1714.

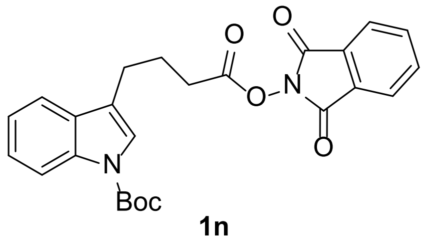

1n

tert-butyl 3-(4-((1,3-dioxoisoindolin-2-yl)oxy)-4-oxobutyl)-1H-indole-1-carboxylate ***(1n)***. Yield (10.0 mmol scale, 2.20 g, 49% yield for 4 steps). A white solid. R_f = 0.4 (Hexanes/EtOAc = 2/1). FC (Hexanes/EtOAc = 6/1). ^1H NMR (500 MHz, $CDCl_3$) δ 8.17 (d, *J* = 6.5 Hz, 1H, ArH), 7.90-7.88 (m, 2H, ArH), 7.80-7.77 (m, 2H, ArH), 7.59 (d, *J* = 7.5 Hz, 1H, ArH), 7.48 (s, 1H, ArH), 7.35 (ddd, *J* = 8.5 Hz, 6.5 Hz, 1.5 Hz, 1H, ArH), 7.30-7.26 (m, 1H, ArH), 2.89 (t, *J* = 7.5 Hz, 2H, CH_2), 2.78 (t, *J* = 7.5 Hz, 2H, CH_2), 2.22 (quin, *J* = 7.5 Hz, 2H, CH_2), 1.70 (s, 9H, Me). $^{13}C\{^1H\}$ NMR (126 MHz, $CDCl_3$) δ 169.48, 161.98, 149.78, 135.64, 134.78, 130.40, 128.91, 124.43, 123.97, 123.00, 122.50, 119.39, 118.98, 115.33, 83.48, 30.44, 28.25, 24.40, 23.86. HRMS (ESI) Calcd. for $C_{25}H_{24}N_2O_6Na^{+1}$ (M^++Na) requires 471.1527; Found: 471.1531.

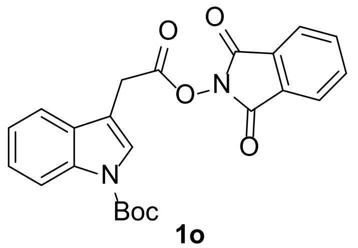

1o

tert-butyl 3-(2-((1,3-dioxoisoindolin-2-yl)oxy)-2-oxoethyl)-1H-indole-1-carboxylate ***(1o)***. Yield (10.0 mmol scale, 2.23 g, 53% yield for 4 steps). A white solid. R_f = 0.4 (Hexanes/EtOAc = 2/1). FC (Hexanes/EtOAc = 6/1). ^1H NMR (500 MHz, $CDCl_3$) δ 8.08 (d, *J* = 8.5 Hz, 1H, ArH), 7.81-7.78 (m, 2H, ArH), 7.71-7.65 (m, 2H, ArH), 7.66 (s, 1H, ArH), 7.52 (d, *J* = 7.5 Hz, 1H, ArH), 7.29-7.25 (m, 1H, ArH), 7.22 (ddd, *J* = 7.5 Hz, 6.0 Hz, 1.5 Hz, 1H, ArH), 4.02 (d, *J* = 1.5 Hz, 2H, CH_2), 1.59 (s, 9H, Me). $^{13}C\{^1H\}$ NMR (126 MHz, $CDCl_3$) δ 167.2, 161.8, 149.5, 135.4, 134.8, 129.5, 128.9, 125.1, 124.9, 124.0, 122.9, 118.9, 115.4, 110.7, 83.9, 28.2, 28.0. HRMS (ESI) Calcd. for $C_{23}H_{20}N_2O_6Na^{+1}$ (M^++Na) requires 443.1214; Found: 443.1215.

1b was prepared from indole propionic acid following the procedure below.[3]

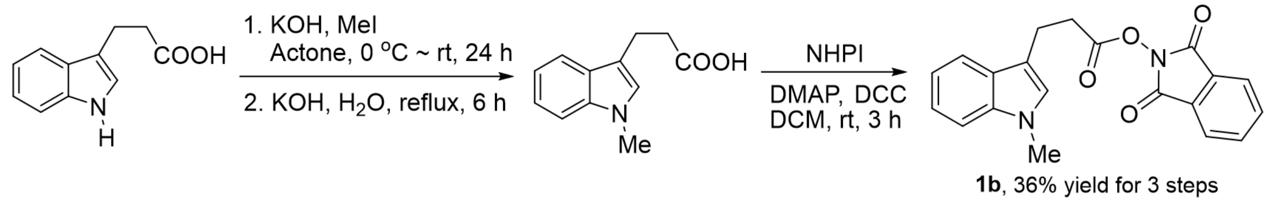

1b, 36% yield for 3 steps

To a solution of indole propionic acid (12.8 mmol) in acetone (120 mL) was added powdered KOH (76.52 mmol) at one portion and MeI (64.00 mmol) dropwisely at 0 ºC. The reaction mixture was stirred at rt for 24 h. After evaporation of the solvent on a RotaVap, the residue was dissolved in water (260 mL) and KOH (63.74 mmol) was added. The mixture was then heated to reflux for 6 h. Cooled to rt, and 6 M HCl was added until complete precipitation of a white solid. The solid was filtered and washed with hexane, affording the desired carboxylic acid intermediate (1.53 g).
To a solution of the crude carboxylic acid intermediate (1.53 g, 7.5 mmol) in DCM (37.5 mL) was added NHPI (9.0 mmol), DMAP (0.75 mmol, 10 mmol%) and DCC (9.0 mmol) at room temperature. After stirring for 3 h, the precipitates in the reaction mixture were filtered through celite and the filtrate was concentrated under reduced pressure on a RotaVap. The crude product was purified by flash column chromatography (FC) on silica gel (eluent: Hexanes/EtOAc = 4/1 ~ 2/1) to yield the desired **1b**.

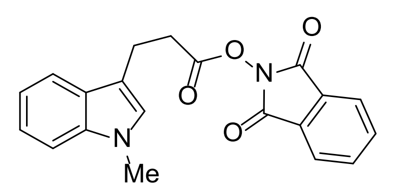

1b

*1,3-dioxoisoindolin-2-yl 3-(1-methyl-1H-indol-3-yl)propanoate (**1b**).* Yield (12.8 mmol scale, 1.61 g, 36% yield for 3 steps). A light yellow solid. R_f = 0.3 (Hexanes/EtOAc = 2/1). FC (Hexanes/EtOAc = 4/1 ~ 2/1). ^{1}H NMR (500 MHz, $CDCl_3$) δ 7.83-7.80 (m, 2H, ArH), 7.72-7.70 (m, 2H, ArH), 7.53 (d, J = 8.0 Hz, 1H, ArH), 7.23 (d, J = 8.0 Hz, 1H, ArH), 7.16 (dd, J = 8.0 Hz, 1.5 Hz, 1H, ArH), 7.06 (ddd, J = 8.0 Hz, 7.0 Hz, 1.5 Hz, 1H, ArH), 6.92 (s, 1H, ArH), 3.70 (s, 3H, Me), 3.17 (t, J = 7.0 Hz, 2H, CH_2), 2.98 (t, J = 7.0 Hz, 2H, CH_2). ^{13}C NMR (126 MHz, $CDCl_3$) δ 169.33, 161.99, 137.04, 134.76, 134.73, 128.97, 127.37, 126.77, 123.98, 121.72, 118.95, 118.57, 112.07, 109.34, 32.67, 32.16, 20.35. HRMS (ESI) Calcd. for $C_{20}H_{17}N_2O_4^{+1}$ (M^++1) requires 349.1183; Found: 349.1179.

1c was prepared from indole propionic acid following the procedure below.[4]

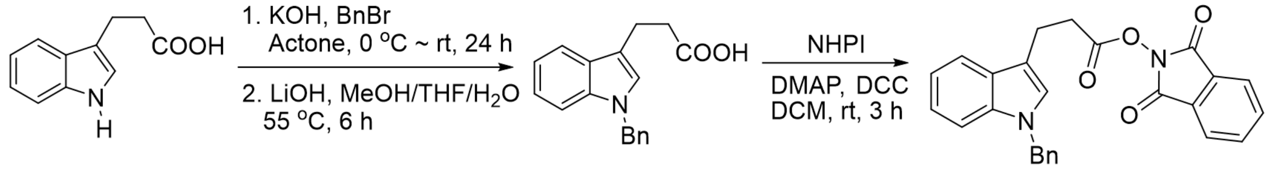

1c, 35% yield for 3 steps

To a solution of indole propionic acid (6.4 mmol) in acetone (60 mL) was added powdered KOH (38.26 mmol) at one portion and BnBr (32.00 mmol) dropwisely at 0 °C. The reaction mixture was stirred at rt for 24 h. After evaporation of the solvent on a RotaVap, the residue was dissolved in water (260 mL) and KOH (63.74 mmol) was added. The mixture was then heated to reflux for 6 h. Cooled to rt, and 6 M HCl was added until pH ~ 3, which was then extracted with EtOAc (3x). The combined organic layer was washed with brine and dried over anhydrous Na_2SO_4. After concentrating under reduced pressure on a RotaVap, the crude product was used directly in the next step without further purification.

To a solution of the above crude product (6.4 mmol) in MeOH/THF (32 mL/64 mL) was added a solution of lithium hydroxide (128 mmol, 20 eq.) in H_2O (64 mL) at room temperature. After stirring at 55 °C for 6 h, the reaction mixture was acidified to pH ~ 4 with 6.0 M HCl at 0 °C and extracted with EtOAc (3x). The combined organic layer was washed with brine and dried over anhydrous Na_2SO_4. After concentrating under reduced pressure on a RotaVap, the crude product was purified by flash column chromatography (FC) on silica gel (eluent: Hexanes/EtOAc = 5/1 ~ 2/1) to yield the carboxylic acid precusor (848 mg).

To a solution of the above carboxylic acid precusor (848 mg, 3.2 mmol) in DCM (16 mL) was added NHPI (3.8 mmol), DMAP (0.32 mmol, 10 mmol%) and DCC (3.8 mmol) at room temperature. After stirring for 3 h, the precipitates in the reaction mixture were filtered through celite and the filtrate was concentrated under reduced pressure on a RotaVap. The crude product was purified by flash column chromatography (FC) on silica gel (eluent: Hexanes/EtOAc = 6/1) to yield the desired **1c**.

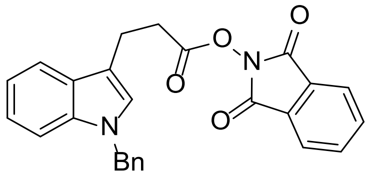

1c

*1,3-dioxoisoindolin-2-yl 3-(1-benzyl-1H-indol-3-yl)propanoate (**1c**).* Yield (6.4 mmol scale, 951 mg, 35% yield for 3 steps). A white solid. R_f = 0.4 (Hexanes/EtOAc = 2/1). FC (Hexanes/EtOAc = 6/1). ^{1}H NMR (500 MHz, $CDCl_3$) δ 7.93-7.90 (m, 2H, ArH), 7.82-7.80 (m, 2H, ArH), 7.66 (ddd, J = 8.0 Hz, 1.0 Hz, 1.0 Hz, 1H, ArH), 7.36-7.25 (m, 4H, ArH), 7.22 (ddd, J = 8.0 Hz, 7.0 Hz, 1.5 Hz, 1H, ArH), 7.19-7.15 (m, 3H, ArH), 7.12 (s, 1H, ArH), 5.34 (s, 2H, CH_2), 3.30 (t, J = 7.5 Hz, 2H, CH_2), 3.10 (t, J = 7.5 Hz, 2H, CH_2). ^{13}C NMR (126 MHz, $CDCl_3$) δ 169.28, 161.96, 137.63, 136.68, 134.75, 128.97, 128.76, 127.68, 127.56, 126.87, 126.21, 123.98, 121.95, 119.25, 118.71, 112.79, 109.88, 50.03, 32.07, 20.42. HRMS (ESI) Calcd. for $C_{26}H_{21}N_2O_4^{+1}$ (M^++1) requires 425.1496; Found: 425.1492.

1p was prepared from indole propionic acid following the following procedure.[2]

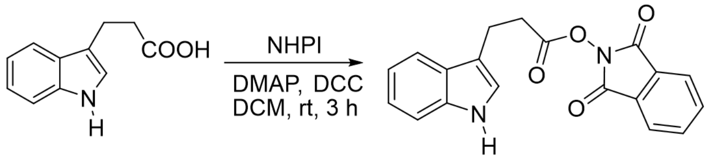

To a solution of indole propionic acid (3.0 mmol) in DCM (15 mL) was added NHPI (3.6 mmol), DMAP (0.3 mmol, 10 mmol%) and DCC (3.6 mmol) at room temperature. After stirring for 3 h, the precipitates in the reaction mixture were filtered through celite and the filtrate was concentrated under reduced pressure on a RotaVap. The crude product was purified by flash column chromatography (FC) on silica gel (eluent: Hexanes/EtOAc = 4/1 ~ 2/1) to yield the desired **1p**.

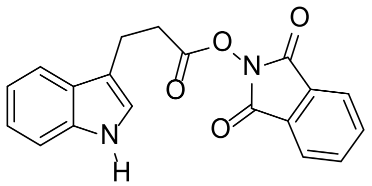

1p

*1,3-dioxoisoindolin-2-yl 3-(1H-indol-3-yl)propanoate (**1p**).* Yield (3.0 mmol scale, 572 mg, 57% yield). A yellow solid. R_f = 0.4 (Hexanes/EtOAc = 1.5/1). FC (Hexanes/EtOAc = 4/1 ~ 2/1). ^{1}H NMR (500 MHz, $CDCl_3$) δ 7.99 (d, J = 1.5 Hz, 1H, ArH), 7.82-7.79 (m, 2H, ArH), 7.72-7.69 (m, 2H, ArH), 7.54 (d, J = 8.0 Hz, 1H, ArH), 7.29 (d, J = 8.0 Hz, 1H, ArH), 7.14 (ddd, J = 8.0, 8.0, 1.5 Hz, 1H, ArH), 7.09-7.02 (m, 2H, ArH, NH), 3.18 (t, J = 7.5 Hz, 2H, CH_2), 2.98 (t, J = 7.5 Hz, 2H, CH_2). ^{13}C NMR (126 MHz, $CDCl_3$) δ 169.30, 162.02, 136.30, 134.78, 128.94, 126.96, 123.98, 122.22, 121.94, 119.53, 118.49, 113.67, 111.28, 31.91, 20.42. HRMS (ESI) Calcd. for $C_{19}H_{15}N_2O_4^{+1}$ (M^++1) requires 335.1026; Found: 335.1028.

4. Unsucessful substrates for photo-mediated radical [4+2] cycloaddtion.

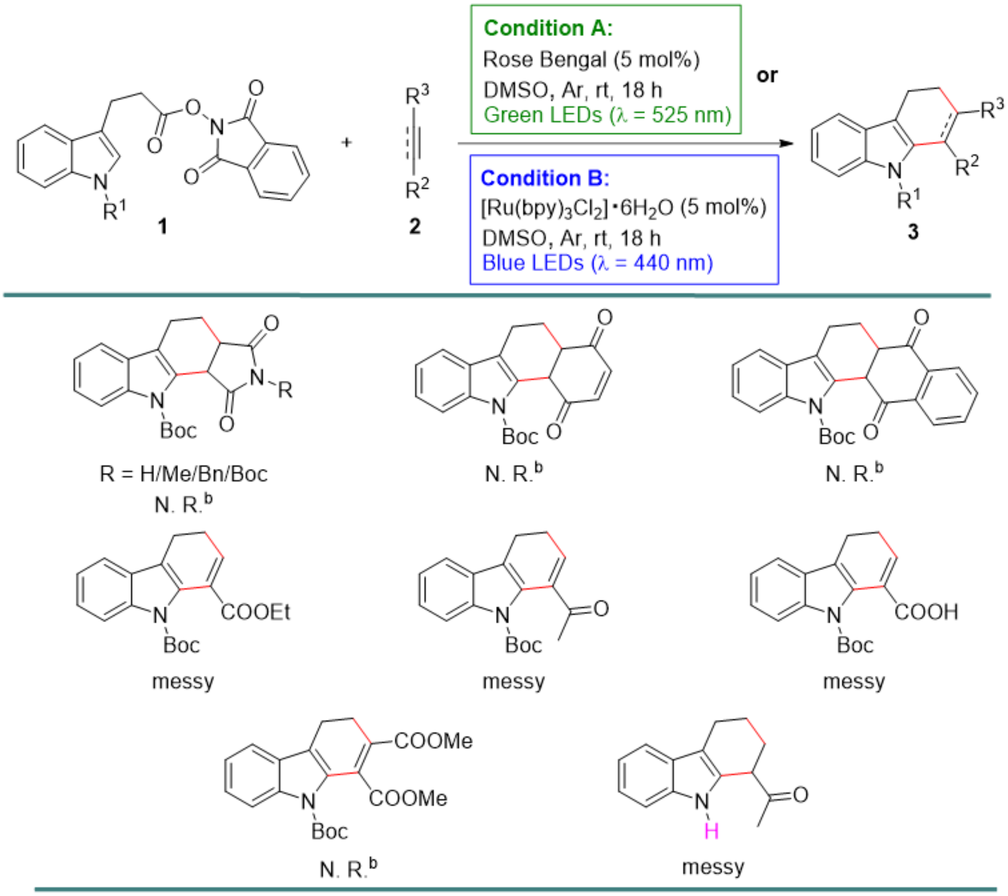

[a] The reaction was conducted with **1** (0.1 mmol), **2** (0.2 mmol) and PC (5 mol%) in DMSO (1.0 mL). N. R. = no reaction. N. D. = not detected.

[b] The reaction was also conducted with *fac*-Ir(ppy)$_3$ (5 mol%) under purple LEDs (390 nm).

Table S1. Unsucessful substrates for photo-mediated radical [4+2] cycloaddtion

5. Blue light/dark interval experiment result

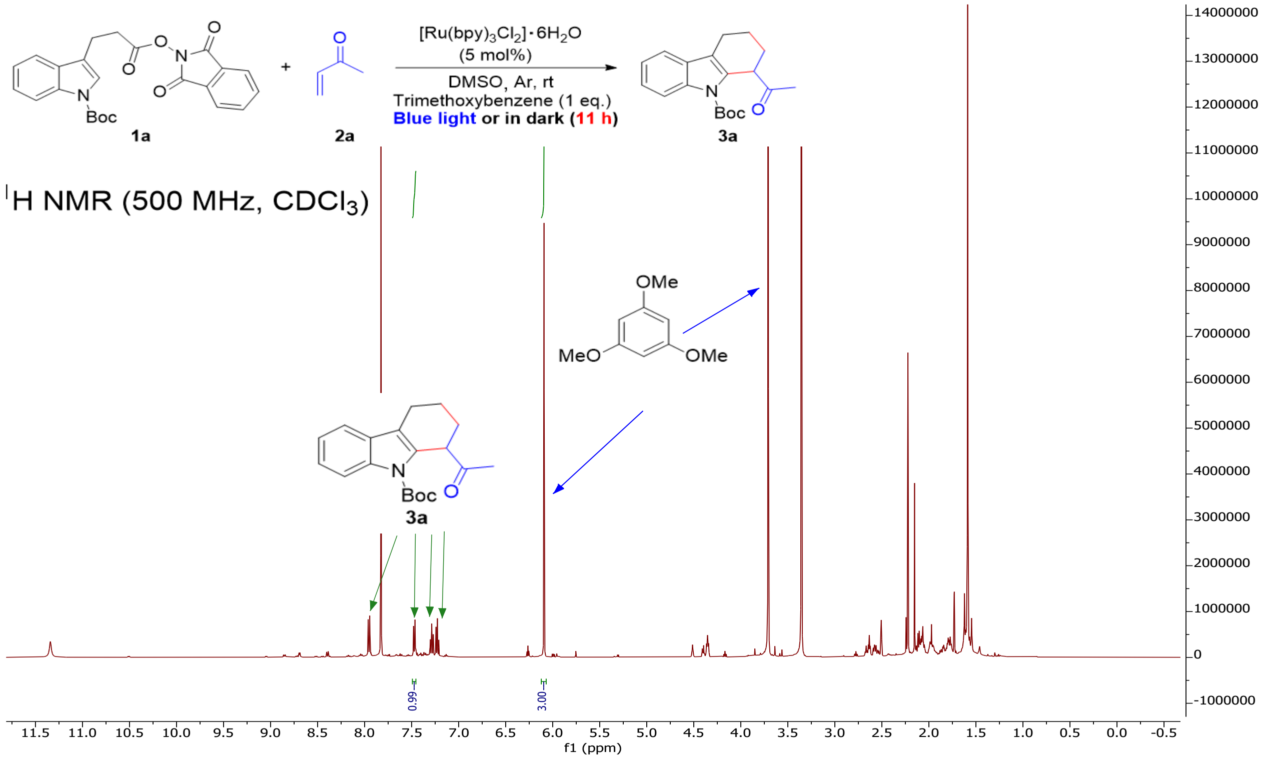

Figure S1. ^{1}H NMR spectrum of blue light/dark interval experiment after 11 hours

6. Alternative mechanism for $[Ru(bpy)_3]^{2+}$-catalyzed radical [4+2] cycloaddition

Alternatively, once the benzyl radical intermediate **III** is formed from the proposed mechanism involving oxidative quenching (Scheme 5), it could also be oxidized by the excited state of Ru(II)* to form the coresponding carbocation intermediate **IV**. Meanwhile, Ru(I) is generated, which further reduces the redox-active indole NHPI ester **1a** to form the alkyl radical species **I**, CO_2, and phthalimide anion via a single electron transfer (SET) process. This step would be favored since the oxidation potential of Ru(I) was reported as $E_{II/I} = -1.33$ V vs. SCE in CH_3CN,[5] which is comparable to the reported ones of alkyl NHPI esters ($E_{red} = -1.20 \sim -1.37$ V vs in CH_3CN).[6] Then, another carbon radical intermediate **II** is formed via the addition of the alkyl radical **I** to substrate **2a**, which further undergoes intramolecular radical addition onto the C-2 position of indole ring. The radical intermediate **III** is generated again, which could continue with the formations of Ru(I) and intermediate **IV**.

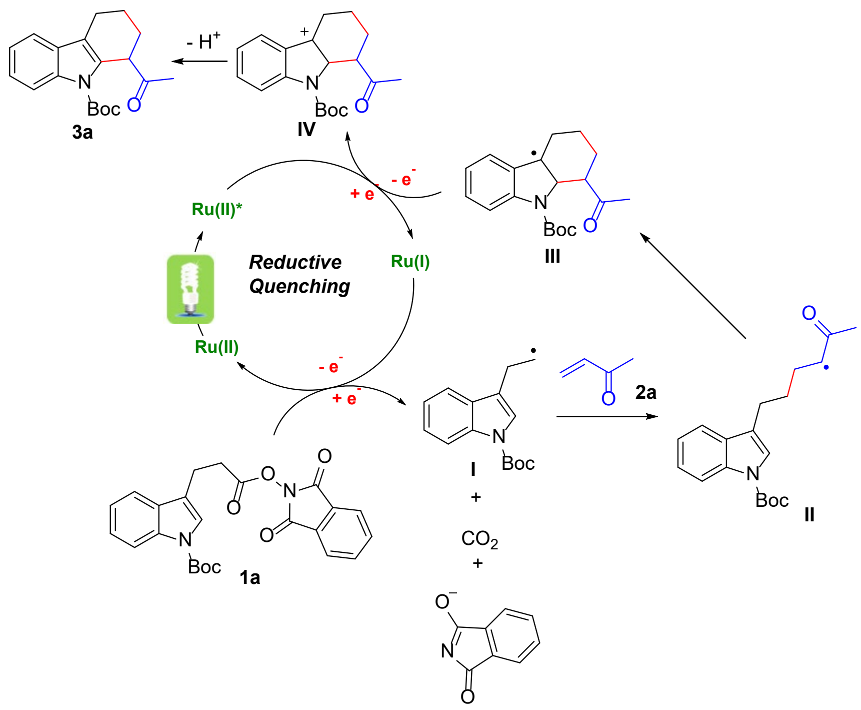

Scheme S1. Alternative mechanism for $[Ru(bpy)_3]^{2+}$-catalyzed radical [4+2] cycloaddition

7. NMR spectra charts for compounds 1 & 3-6

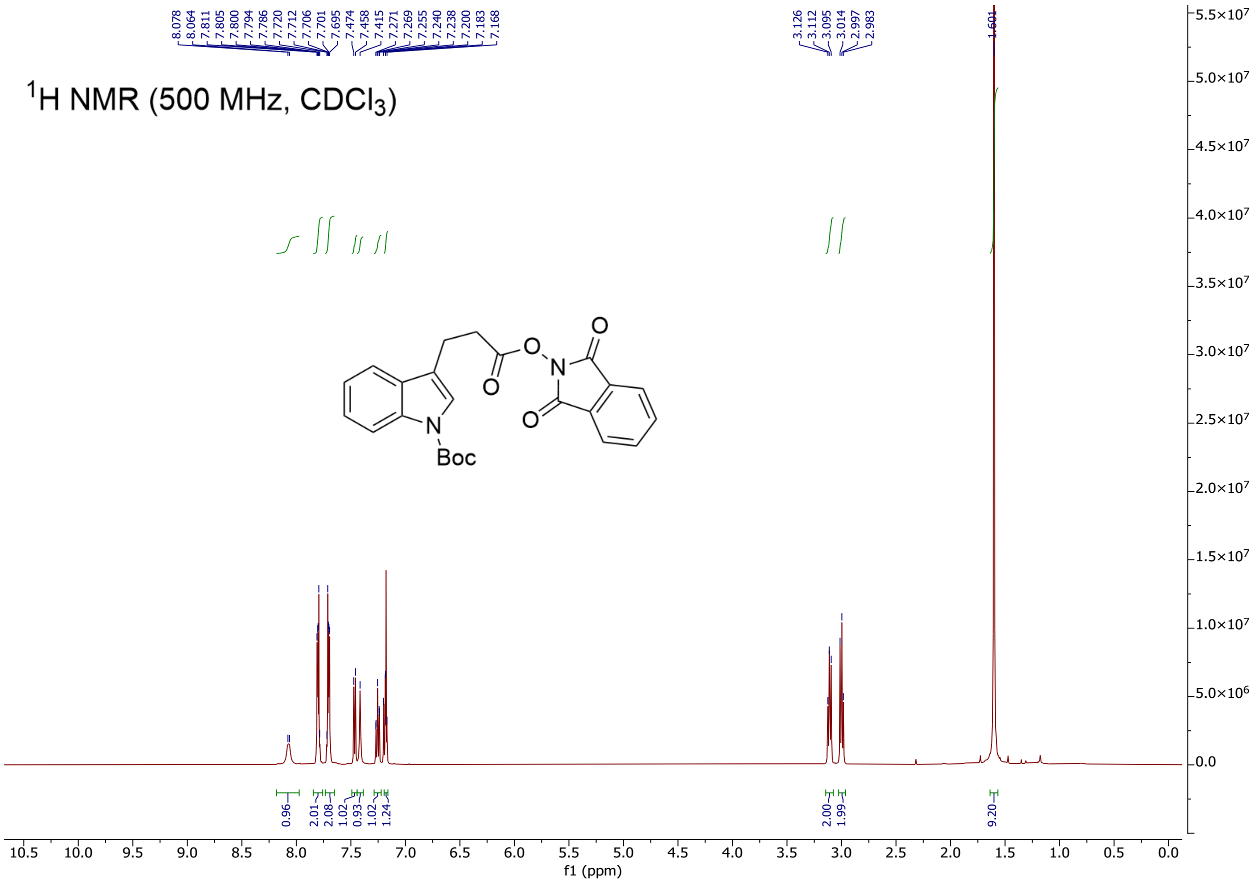

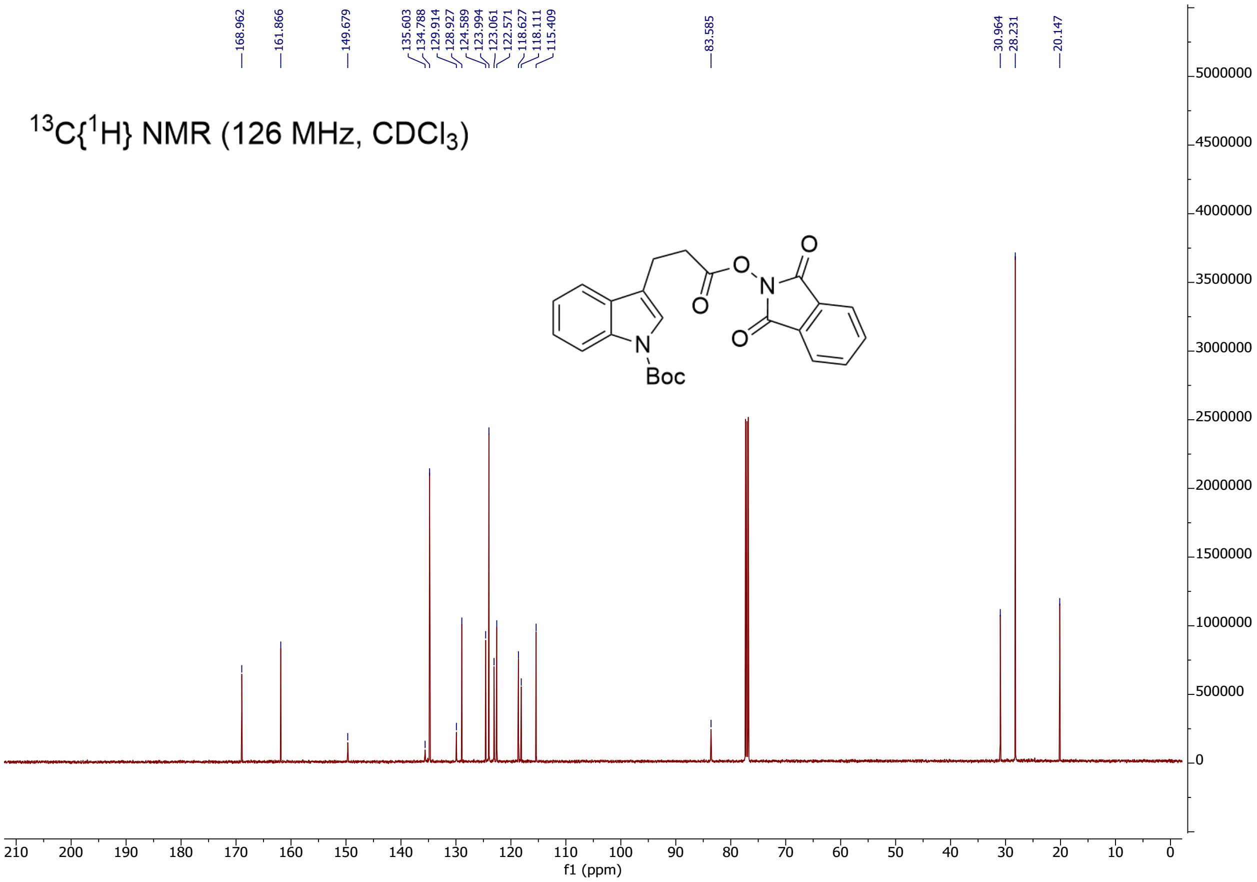

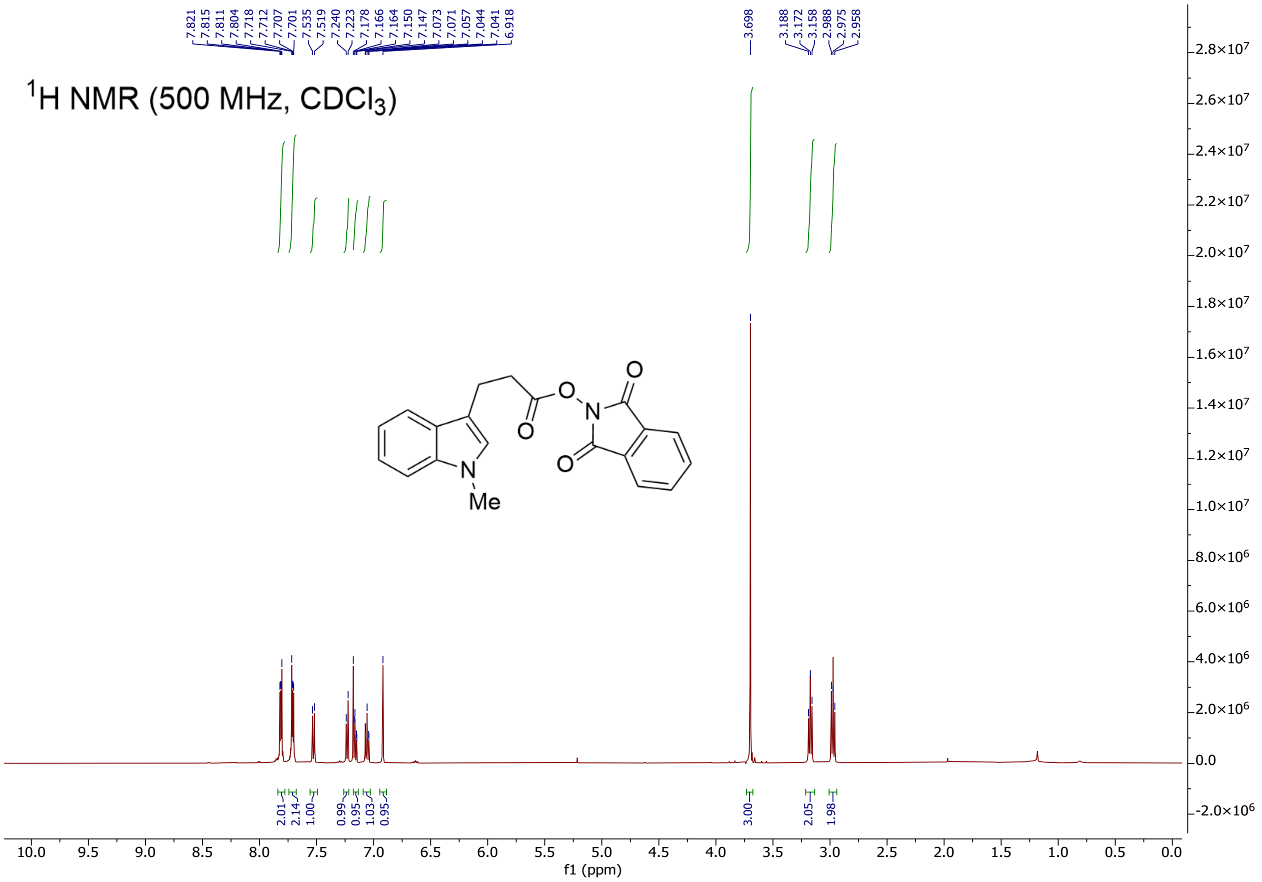
^{1}H NMR (500 MHz, CDCl$_3$)
7.821 7.815 7.811 7.804 7.718 7.712 7.707 7.701 7.535 7.519 7.240 7.223 7.178 7.166 7.164 7.150 7.147 7.073 7.071 7.057 7.044 7.041 6.918
3.698
3.188 3.172 3.158 2.988 2.975 2.958
2.01 2.14 1.00 0.99 0.95 1.03 0.95 3.00 2.05 1.98
O
N
O
O
O
N
Me
f1 (ppm)

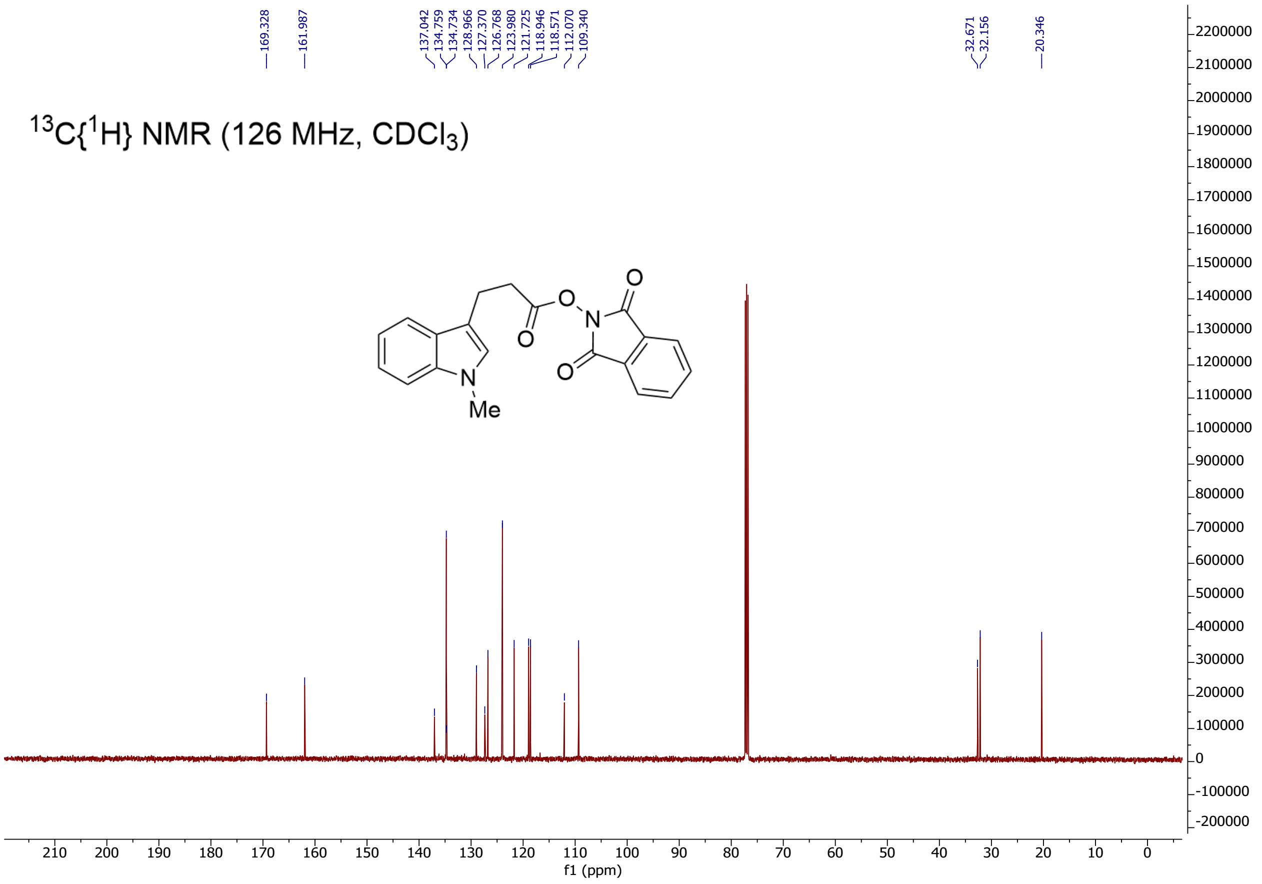
^{13}C{^{1}H} NMR (126 MHz, CDCl$_3$)
169.328
161.987
137.042 134.759 134.734 128.966 127.370 126.768 123.980 121.725 118.946 118.571 112.070 109.340
32.671 32.156
20.346
O
N
O
O
O
N
Me
f1 (ppm)

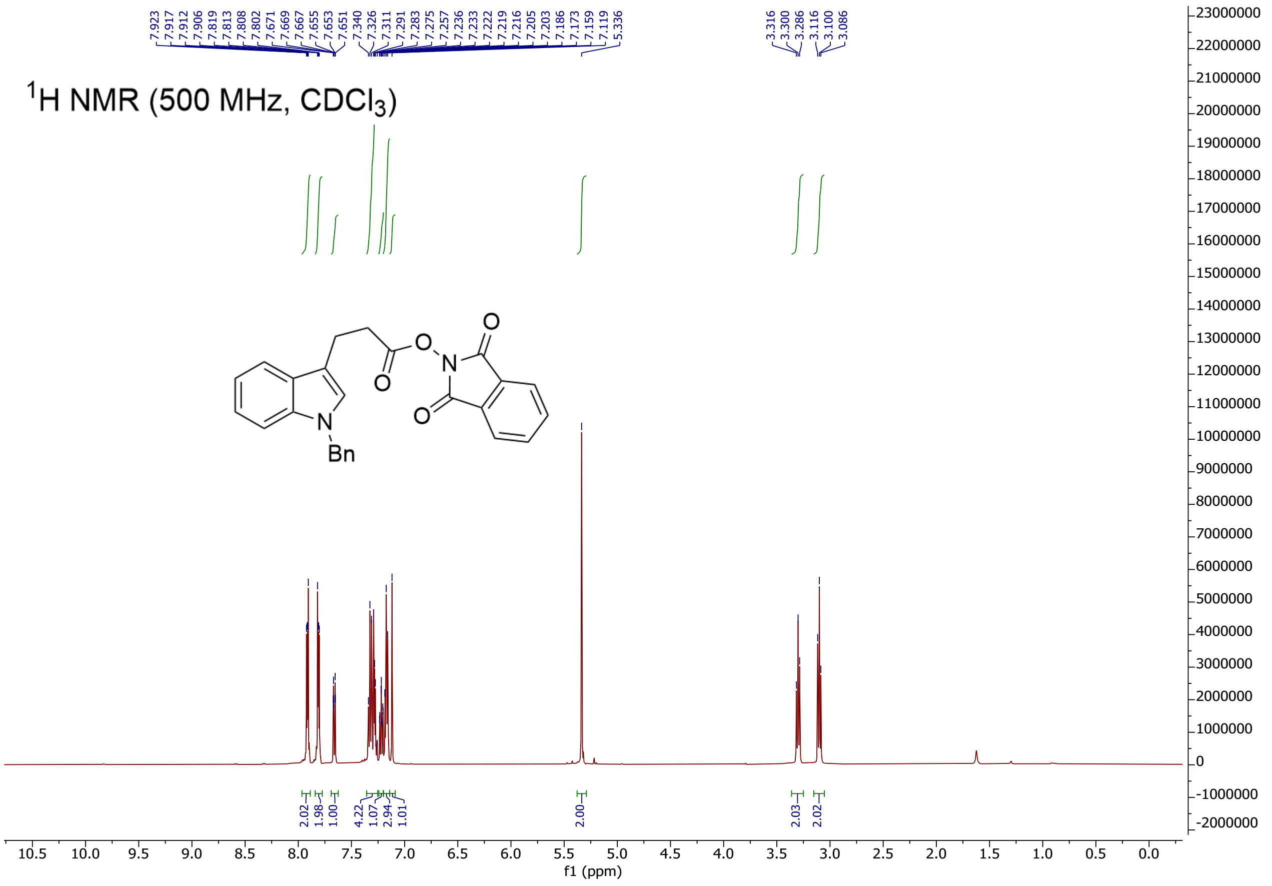

7.923
7.917
7.912
7.906
7.819
7.813
7.808
7.802
7.671
7.669
7.667
7.655
7.653
7.651
7.340
7.326
7.311
7.291
7.283
7.275
7.257
7.236
7.233
7.222
7.219
7.216
7.205
7.203
7.186
7.173
7.159
7.119
5.336
3.316
3.300
3.286
3.116
3.100
3.086
^{1}H NMR (500 MHz, CDCl$_3$)
N
Bn
O
O
N
O
O
2.02
1.98
1.00
4.22
1.07
2.94
1.01
2.00
2.03
2.02
f1 (ppm)

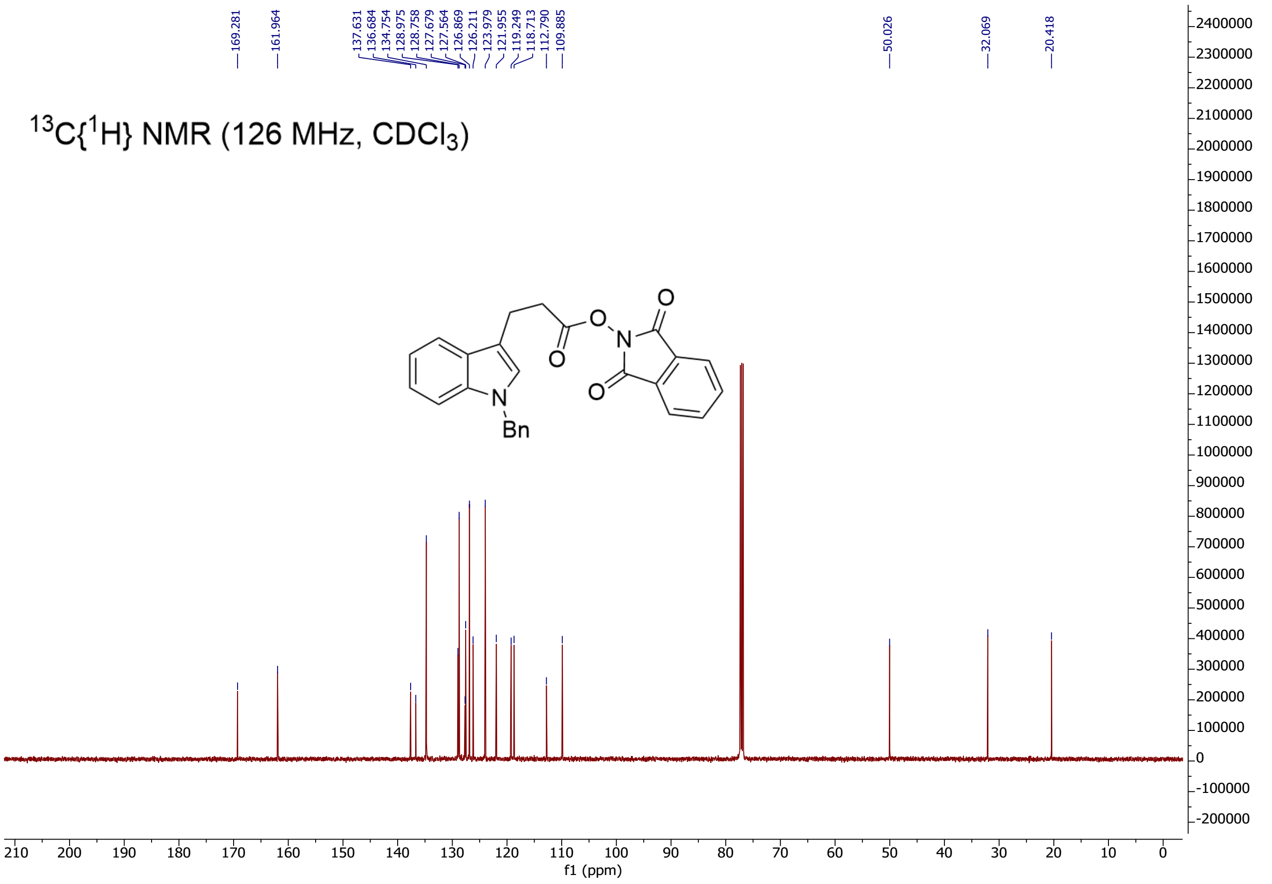

169.281
161.964
137.631
136.684
134.754
128.975
128.758
127.679
127.564
126.869
126.211
123.979
121.955
119.249
118.713
112.790
109.885
50.026
32.069
20.418
^{13}C{^{1}H} NMR (126 MHz, CDCl$_3$)
N
Bn
O
O
N
O
O
f1 (ppm)

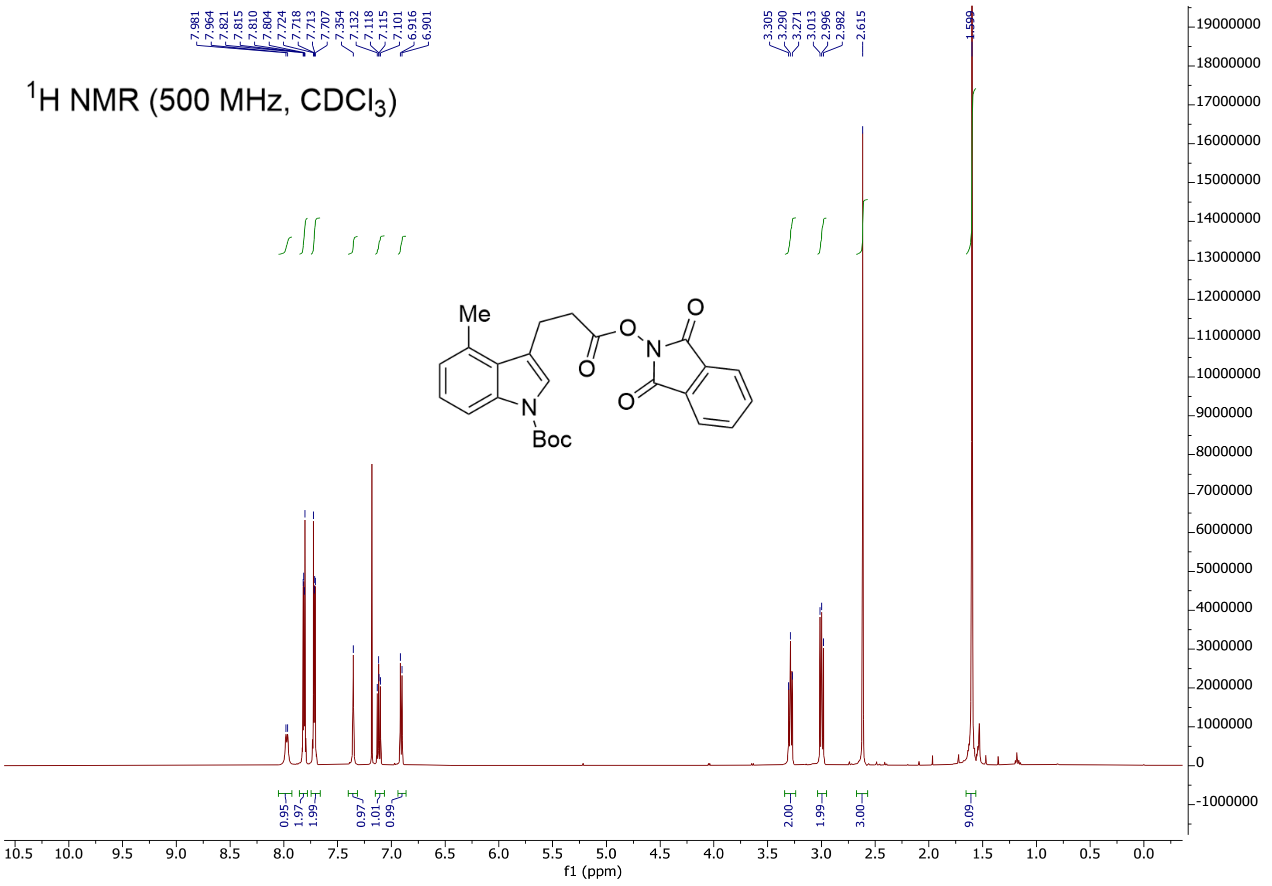

7.981
7.964
7.821
7.815
7.810
7.804
7.724
7.718
7.713
7.707
7.354
7.132
7.118
7.115
7.101
6.916
6.901
3.305
3.290
3.271
3.013
2.996
2.982
2.615
1.599
1H NMR (500 MHz, CDCl3)
Me
O
O
N
O
O
N
Boc
O
0.95
1.97
1.99
0.97
1.01
0.99
2.00
1.99
3.00
9.09
f1 (ppm)

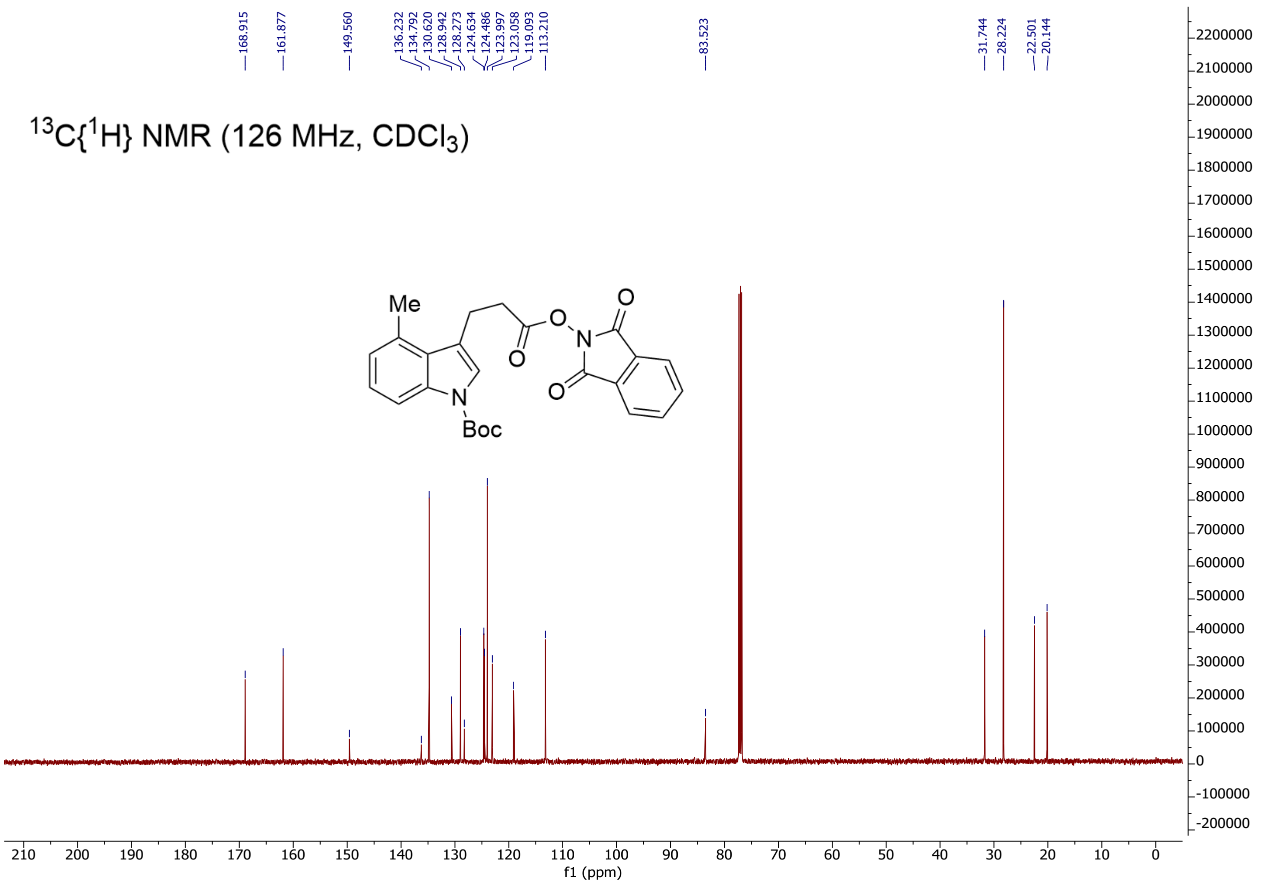

168.915
161.877
149.560
136.232
134.792
130.620
128.942
128.273
124.634
124.486
123.997
123.058
119.093
113.210
83.523
31.744
28.224
22.501
20.144
13C{1H} NMR (126 MHz, CDCl3)
Me
O
O
N
O
O
N
Boc
O
f1 (ppm)

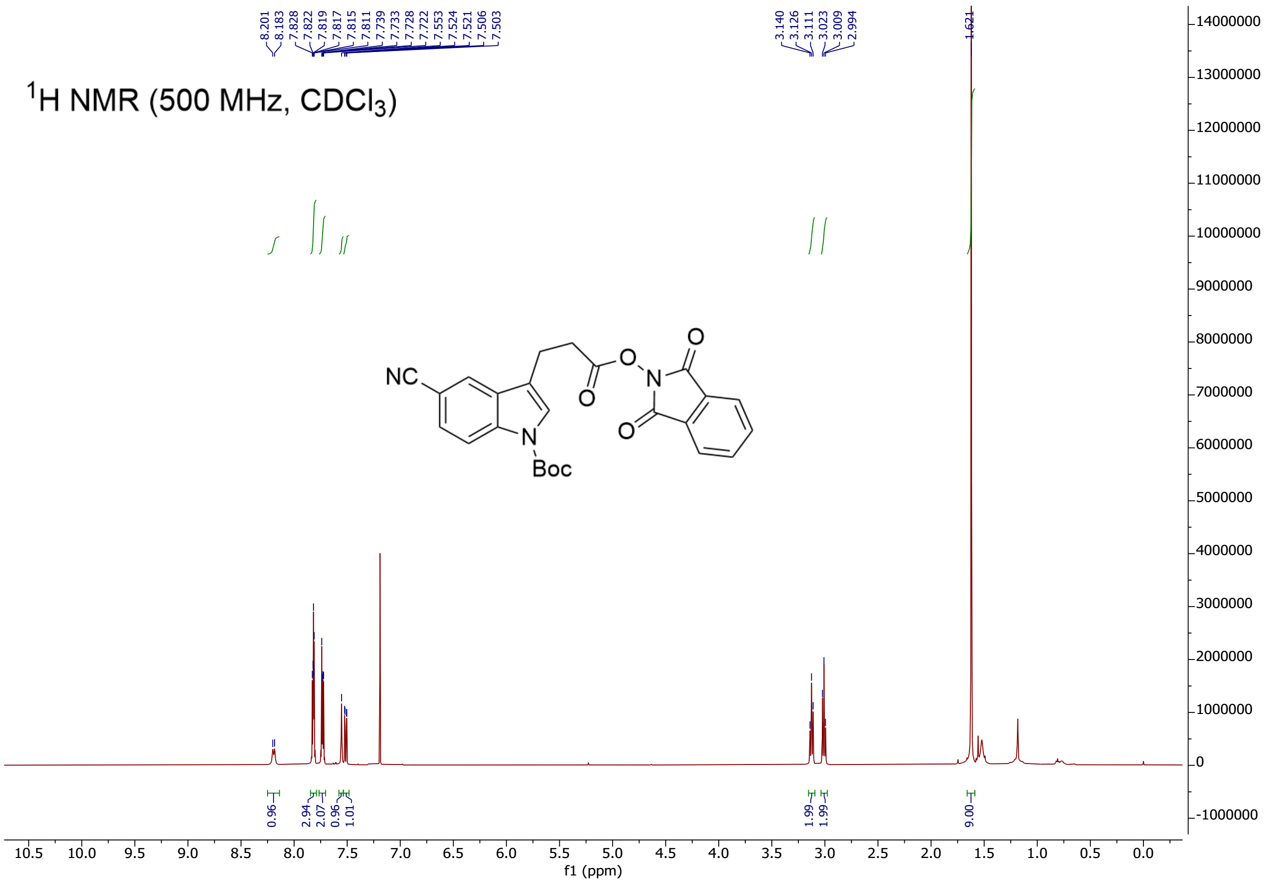

^{1}H NMR (500 MHz, $CDCl_3$)
8.201 8.183 7.828 7.822 7.819 7.817 7.815 7.811 7.739 7.733 7.728 7.722 7.553 7.524 7.521 7.506 7.503
3.140 3.126 3.111 3.023 3.009 2.994
1.621
NC
O
N
Boc
0.96 2.94 2.07 0.96 1.01 1.99 1.99 9.00
f1 (ppm)

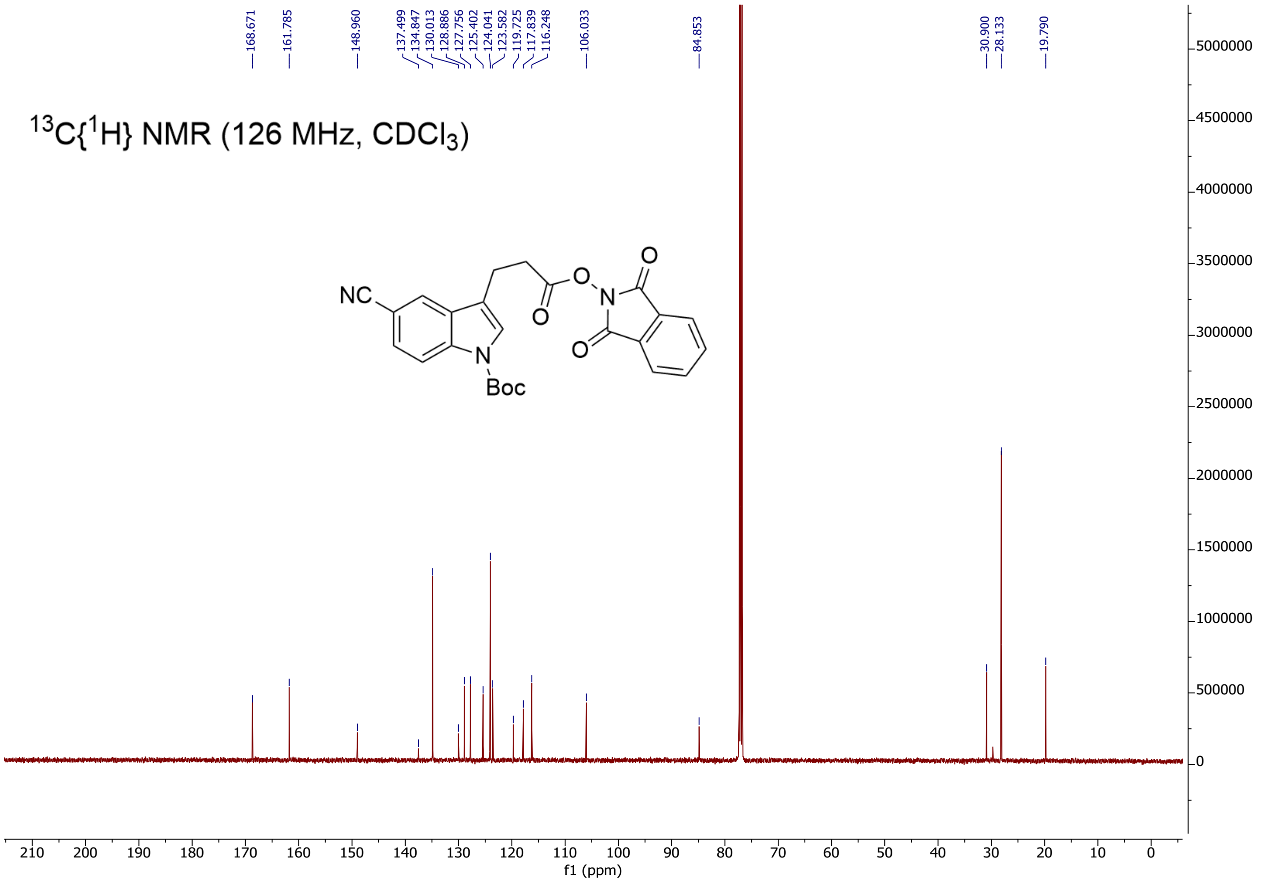

^{13}C{^{1}H} NMR (126 MHz, $CDCl_3$)
168.671 161.785 148.960 137.499 134.847 130.013 128.886 127.756 125.402 124.041 123.582 119.725 117.839 116.248 106.033 84.853 30.900 28.133 19.790
NC
O
N
Boc
f1 (ppm)

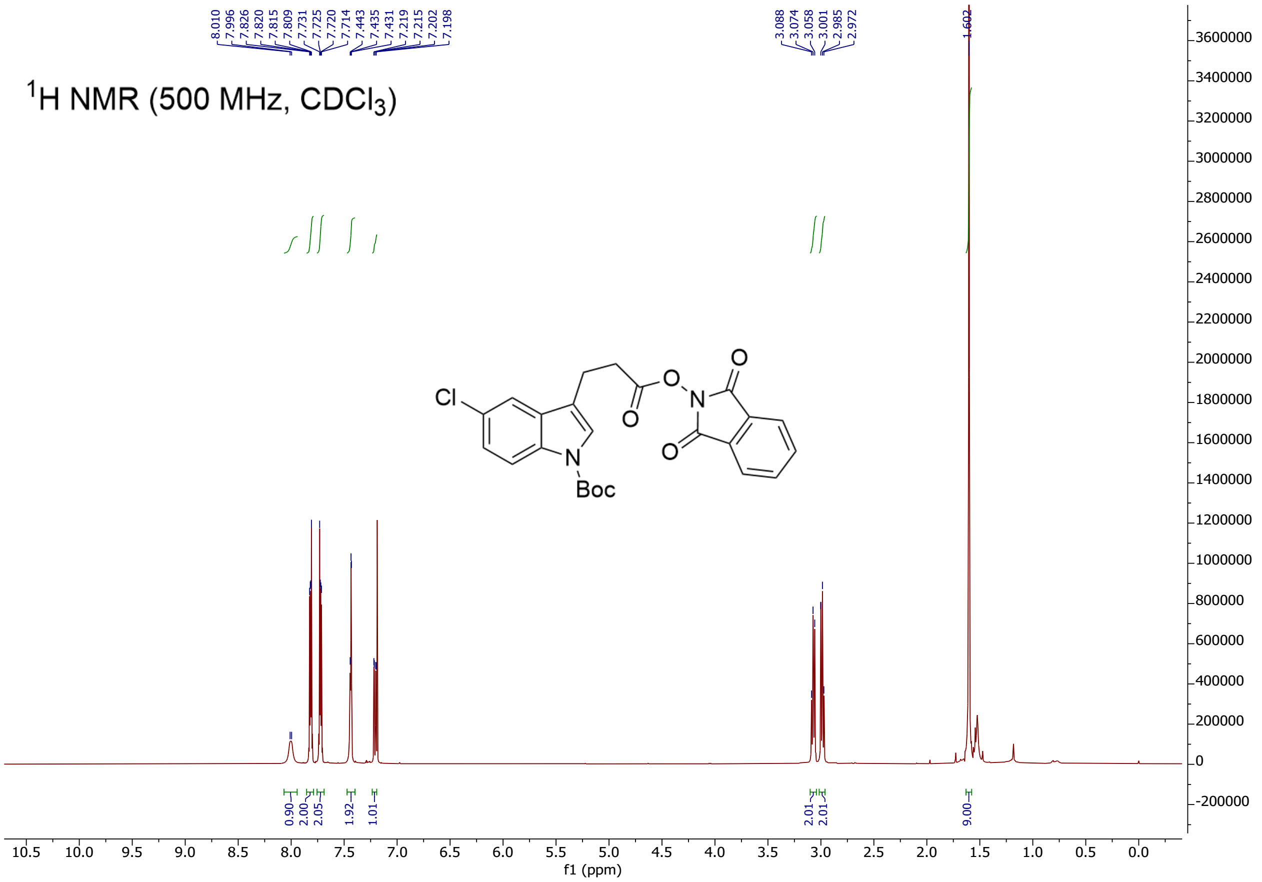

8.010
7.996
7.826
7.820
7.815
7.809
7.731
7.725
7.720
7.714
7.443
7.435
7.431
7.219
7.215
7.202
7.198
3.088
3.074
3.058
3.001
2.985
2.972
1.602
1H NMR (500 MHz, CDCl3)
Cl
N
Boc
O
O
N
O
O
0.90
2.00
2.05
1.92
1.01
2.01
2.01
9.00
f1 (ppm)

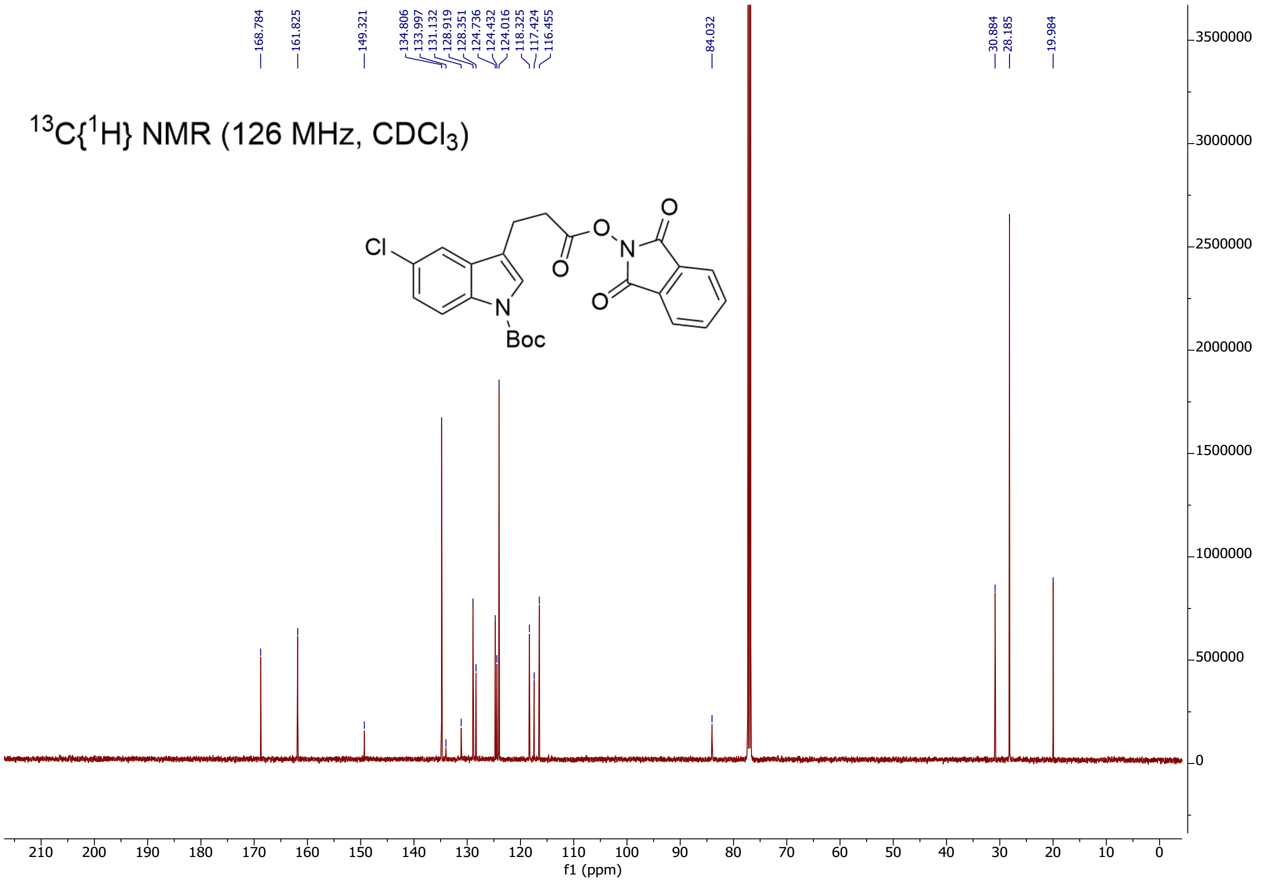

168.784
161.825
149.321
134.806
133.997
131.132
128.919
128.351
124.736
124.432
124.016
118.325
117.424
116.455
84.032
30.884
28.185
19.984
13C{1H} NMR (126 MHz, CDCl3)
Cl
N
Boc
O
O
N
O
O
f1 (ppm)

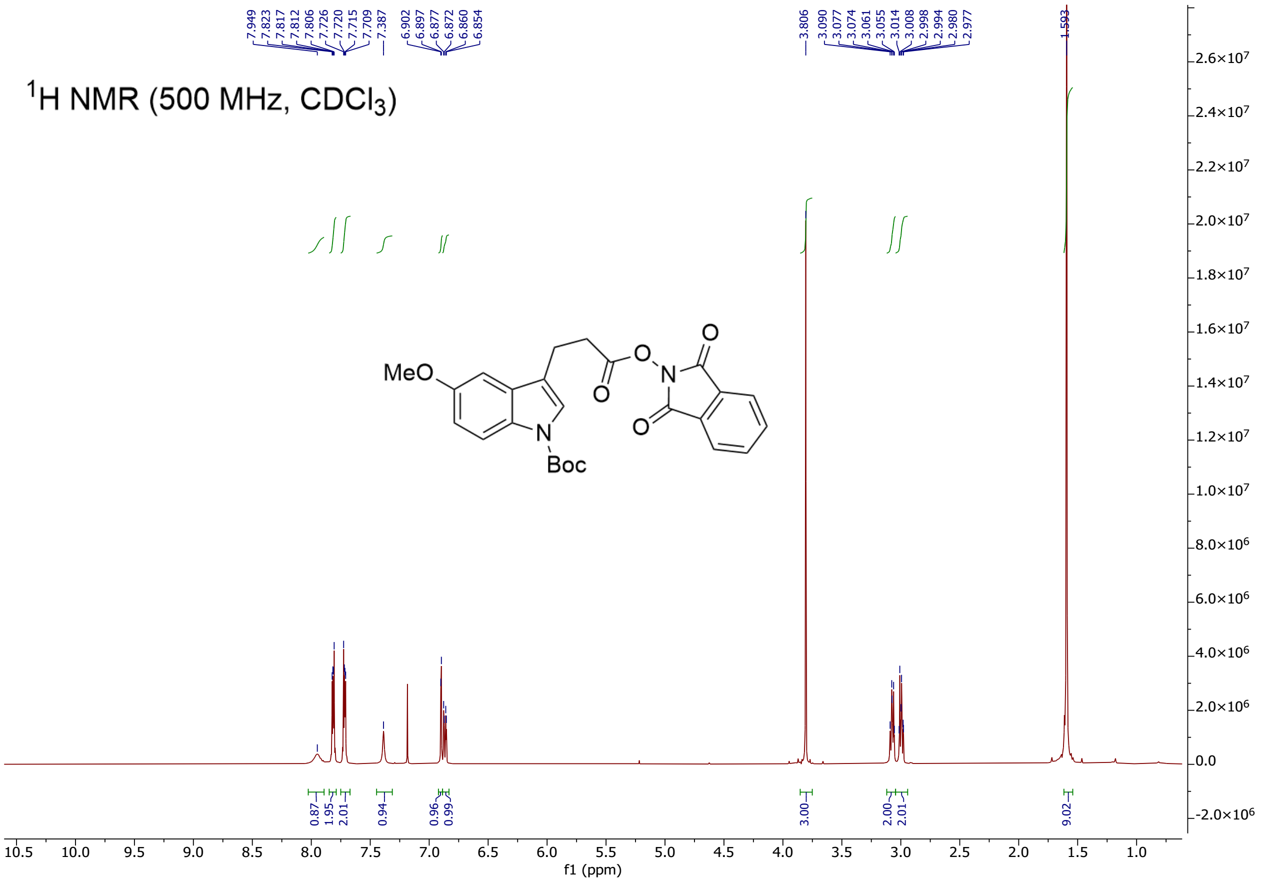

^{1}H NMR (500 MHz, CDCl$_3$)
MeO
N
Boc
O
f1 (ppm)

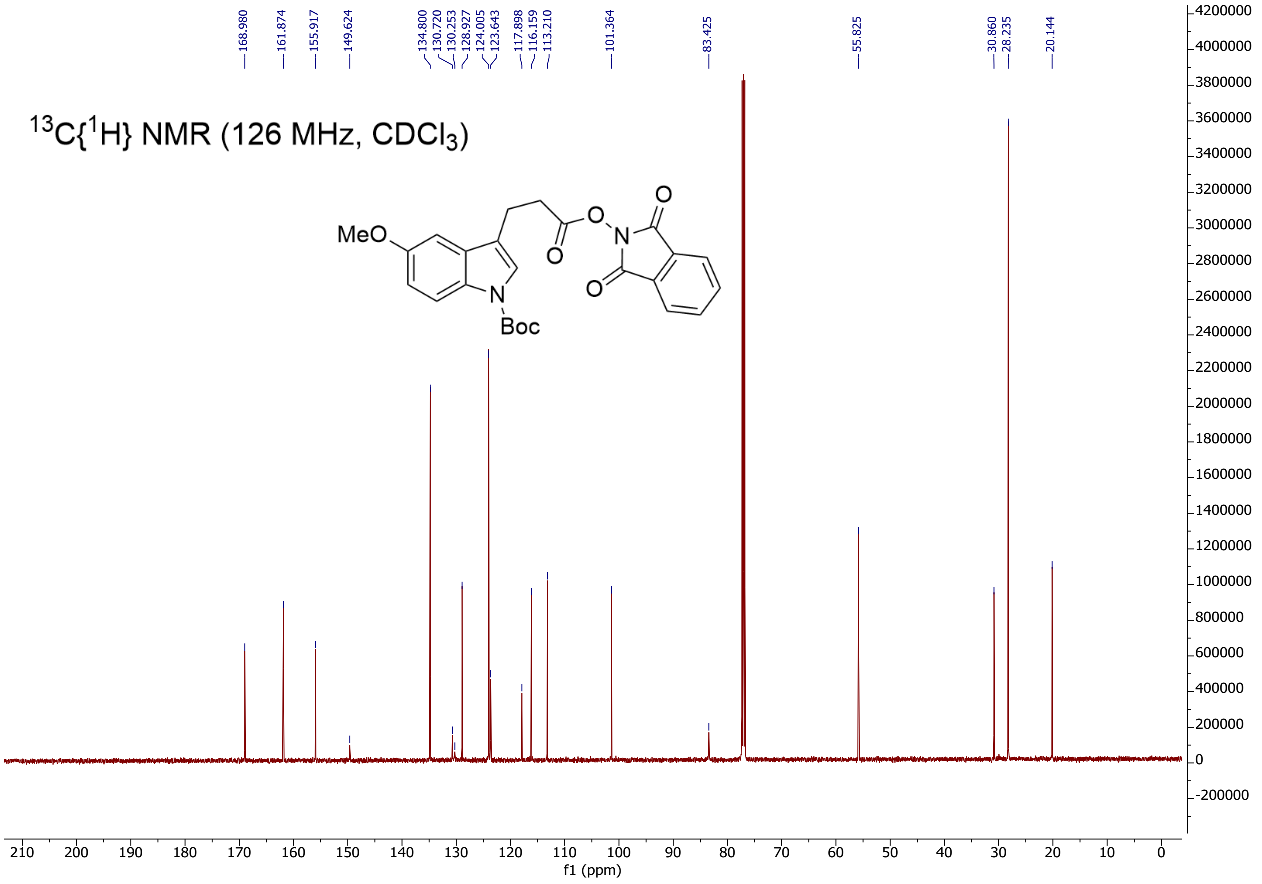

^{13}C{^{1}H} NMR (126 MHz, CDCl$_3$)
MeO
N
Boc
O
f1 (ppm)

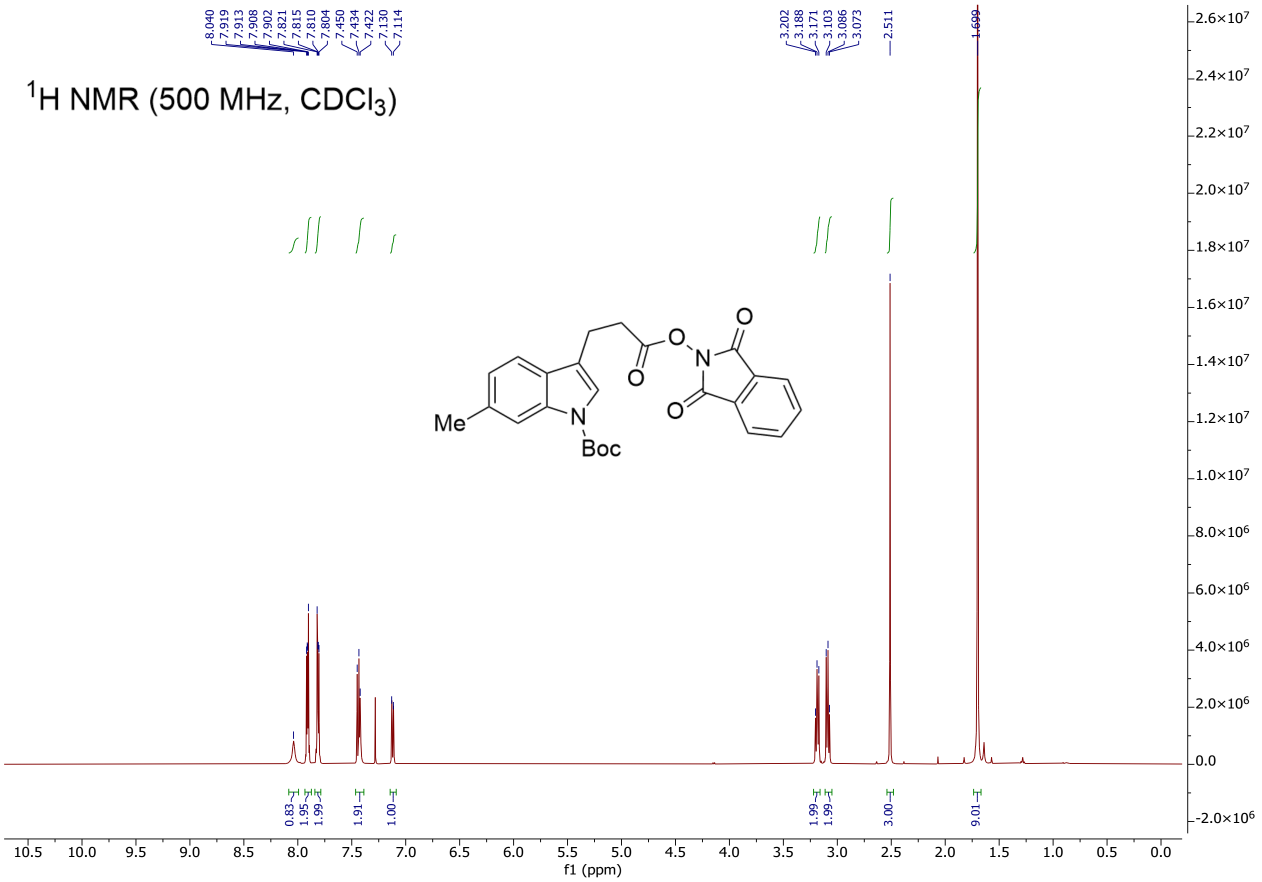

8.040
7.919
7.913
7.908
7.902
7.821
7.815
7.810
7.804
7.450
7.434
7.422
7.130
7.114
3.202
3.188
3.171
3.103
3.086
3.073
2.511
1.699
1H NMR (500 MHz, CDCl3)
Me
N
Boc
O
O
N
O
O
0.83
1.95
1.99
1.91
1.00
1.99
1.99
3.00
9.01
f1 (ppm)

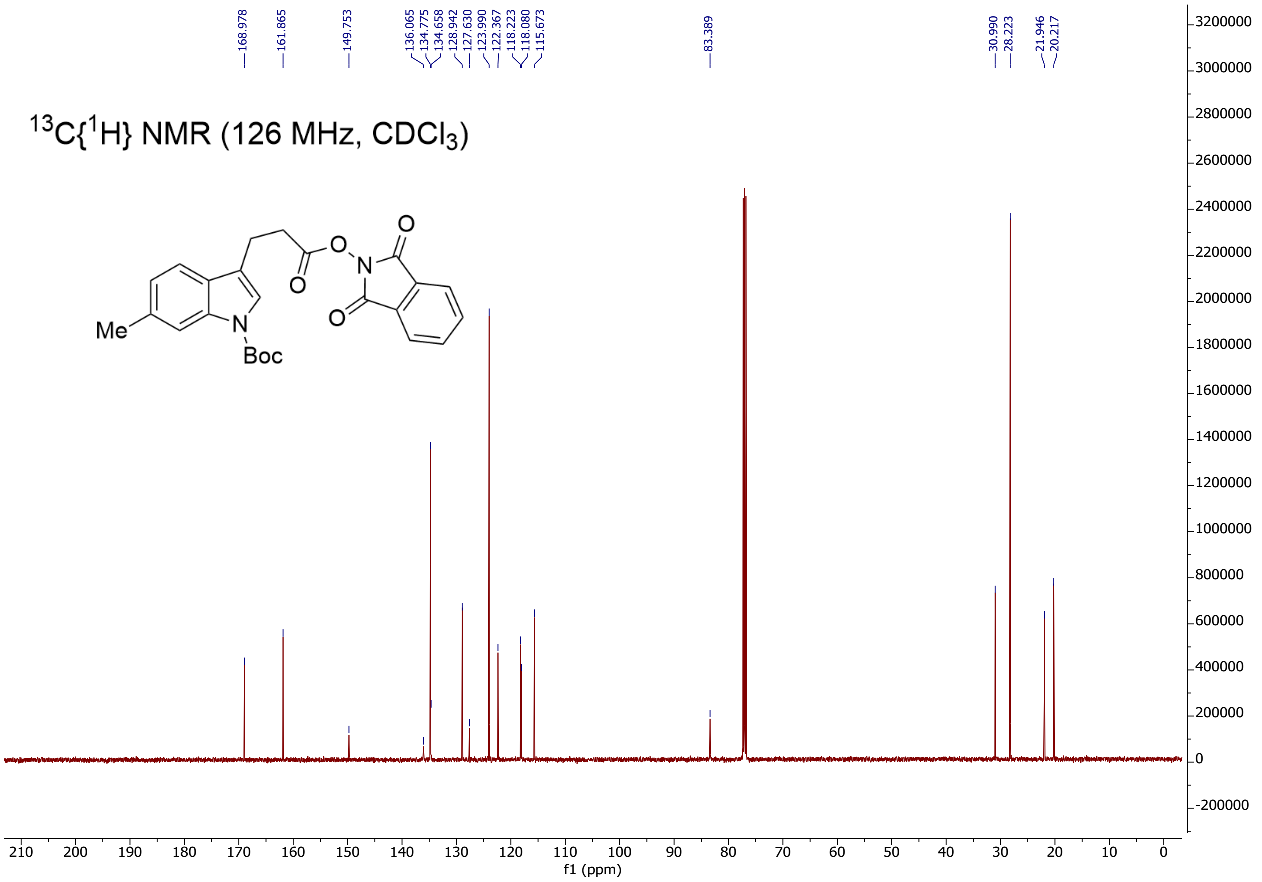

168.978
161.865
149.753
136.065
134.775
134.658
128.942
127.630
123.990
122.367
118.223
118.080
115.673
83.389
30.990
28.223
21.946
20.217
13C{1H} NMR (126 MHz, CDCl3)
Me
N
Boc
O
O
N
O
O
f1 (ppm)

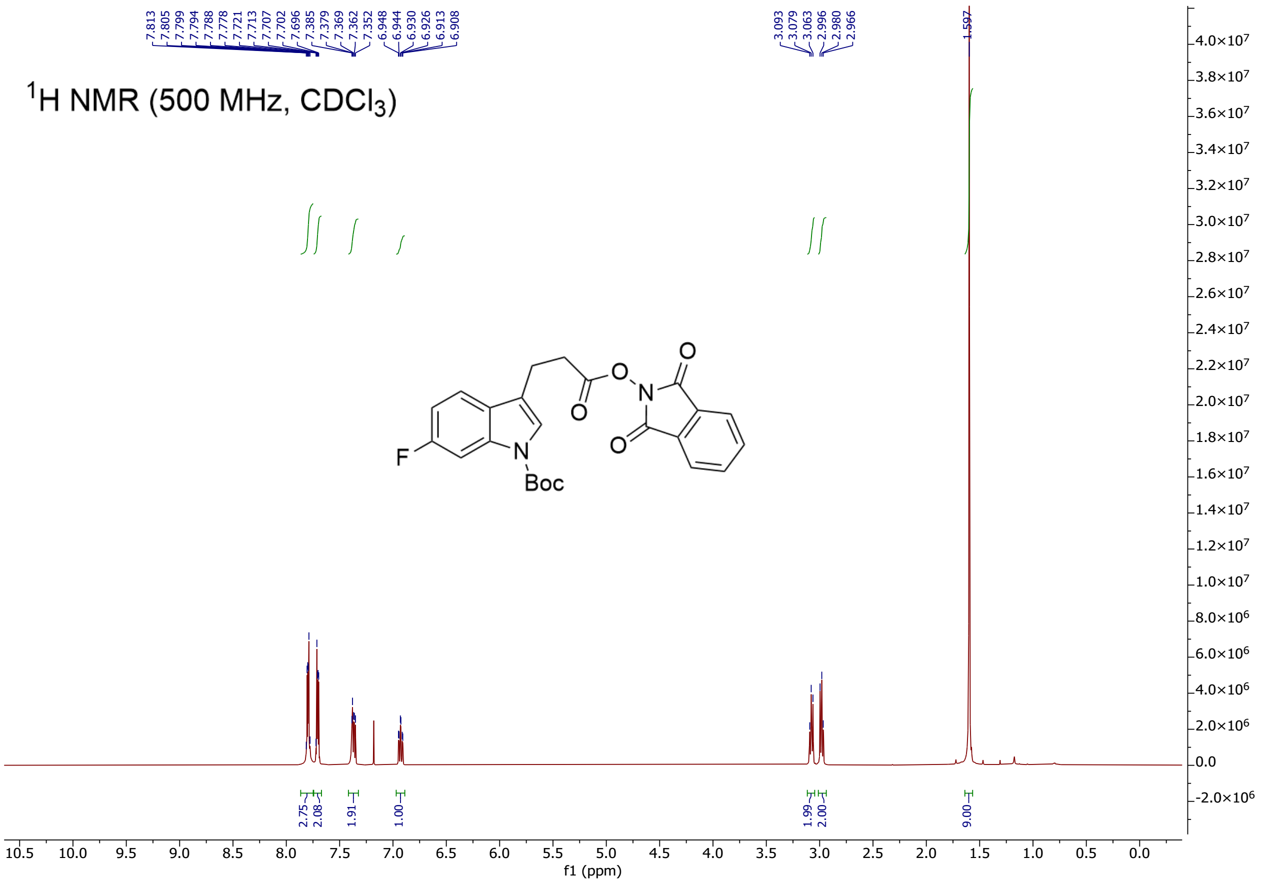
1H NMR (500 MHz, CDCl3)
F
N
Boc
O
O
N
O
O
f1 (ppm)

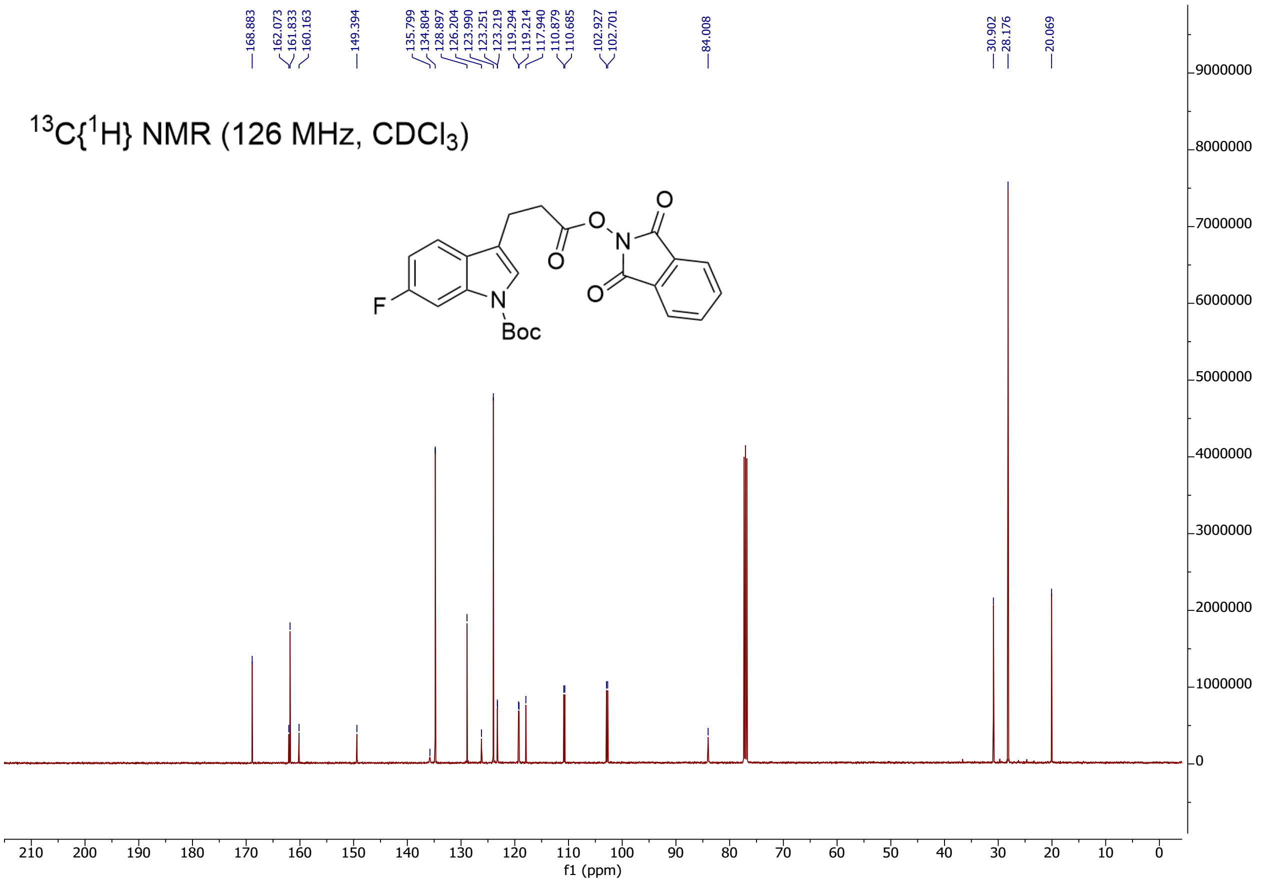
13C{1H} NMR (126 MHz, CDCl3)
F
N
Boc
O
O
N
O
O
f1 (ppm)

^{19}F NMR (471 MHz, $CDCl_3$)

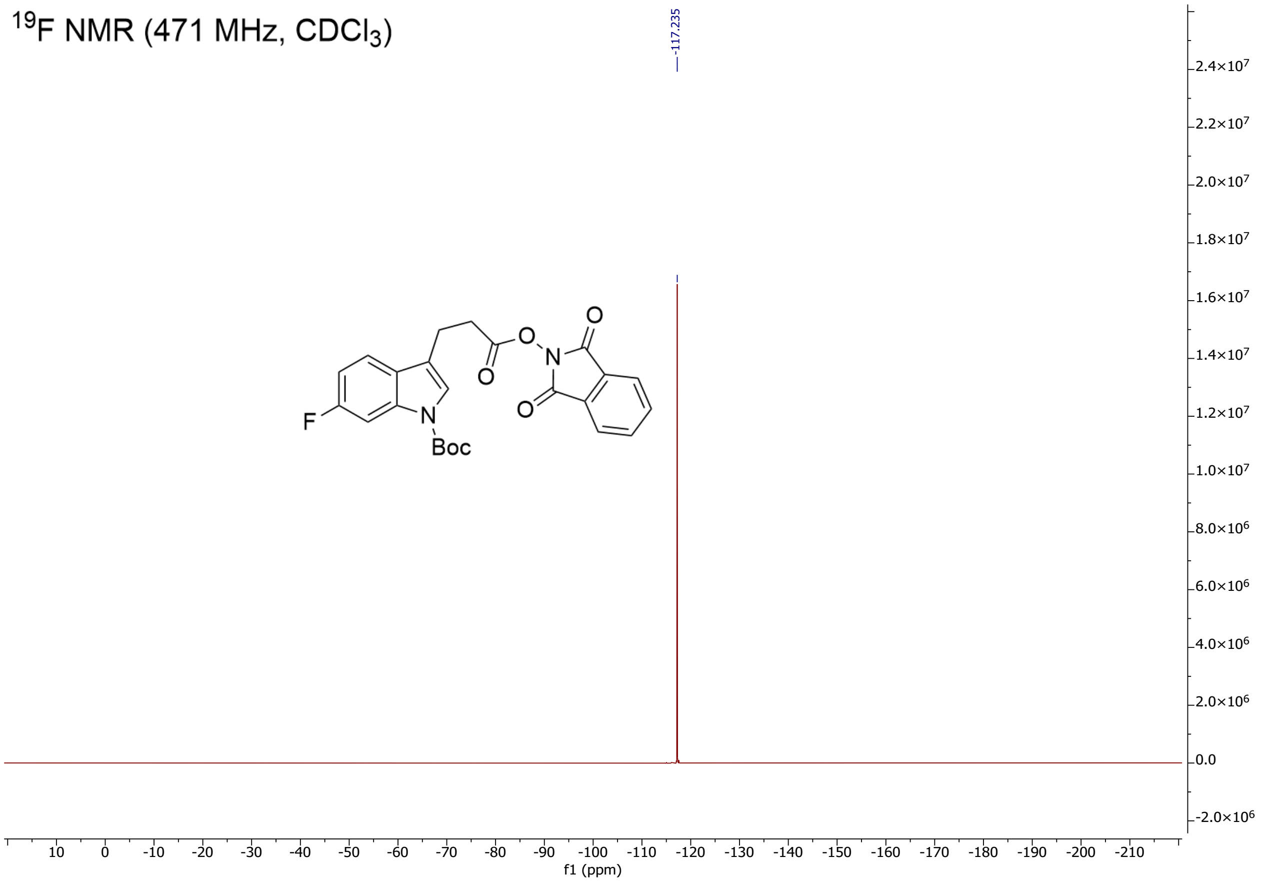

^{1}H NMR (500 MHz, $CDCl_3$)

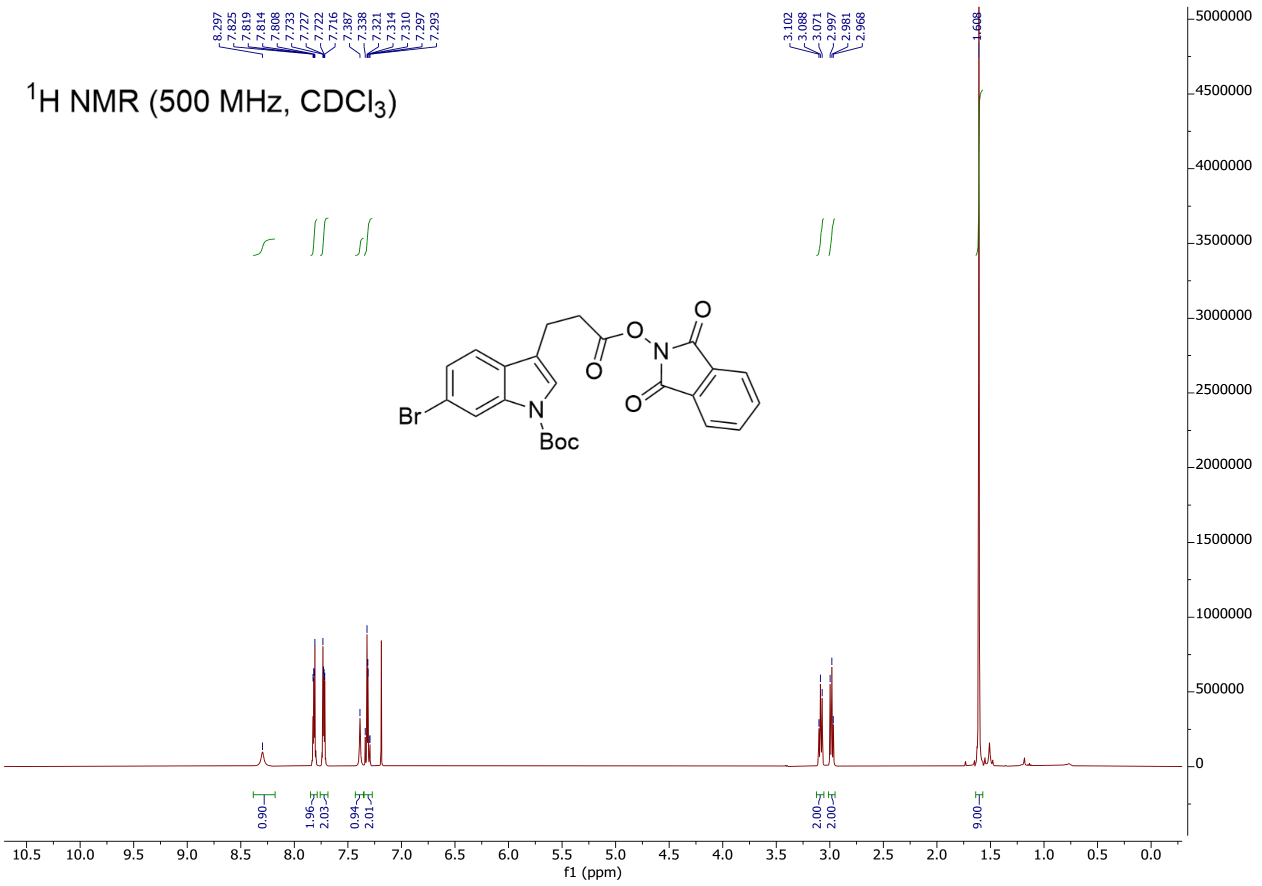

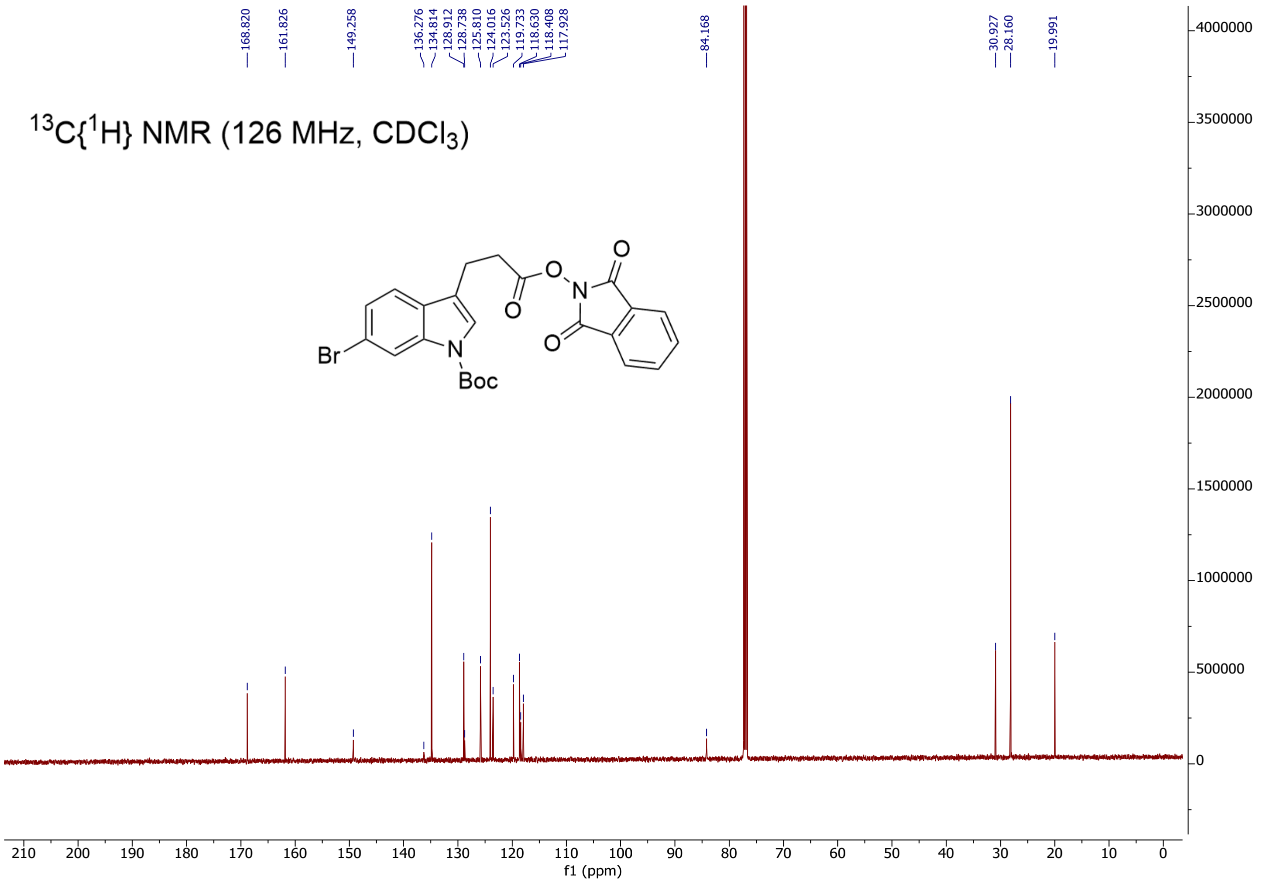
13C{1H} NMR (126 MHz, CDCl3)
168.820
161.826
149.258
136.276
134.814
128.912
128.738
125.810
124.016
123.526
119.733
118.630
118.408
117.928
84.168
30.927
28.160
19.991
Br
N
Boc
O
O
N
O
O
f1 (ppm)

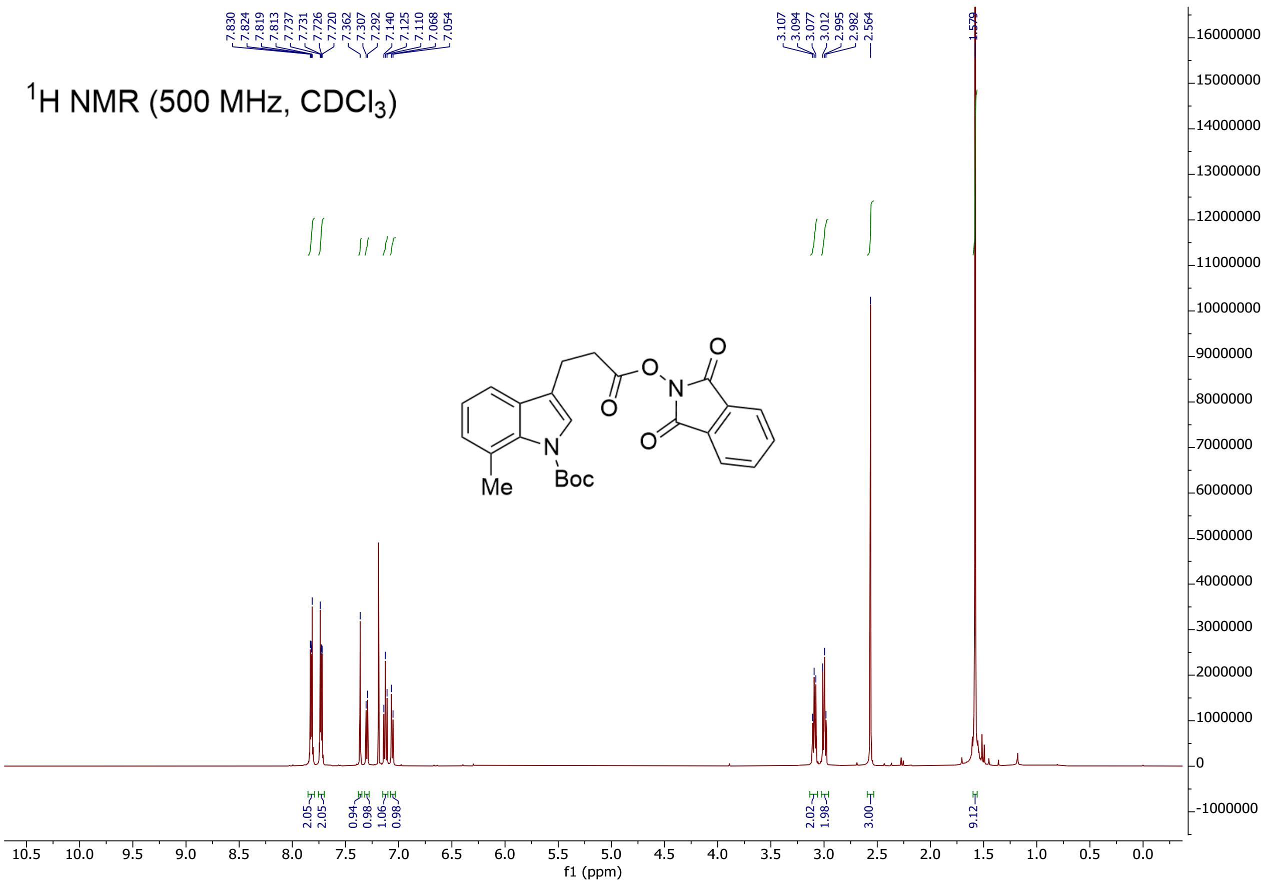
1H NMR (500 MHz, CDCl3)
7.830
7.824
7.819
7.813
7.737
7.731
7.726
7.720
7.362
7.307
7.292
7.140
7.125
7.110
7.068
7.054
3.107
3.094
3.077
3.012
2.995
2.982
2.564
1.579
Me
N
Boc
O
O
N
O
O
2.05
2.05
0.94
0.98
1.06
0.98
2.02
1.98
3.00
9.12
f1 (ppm)

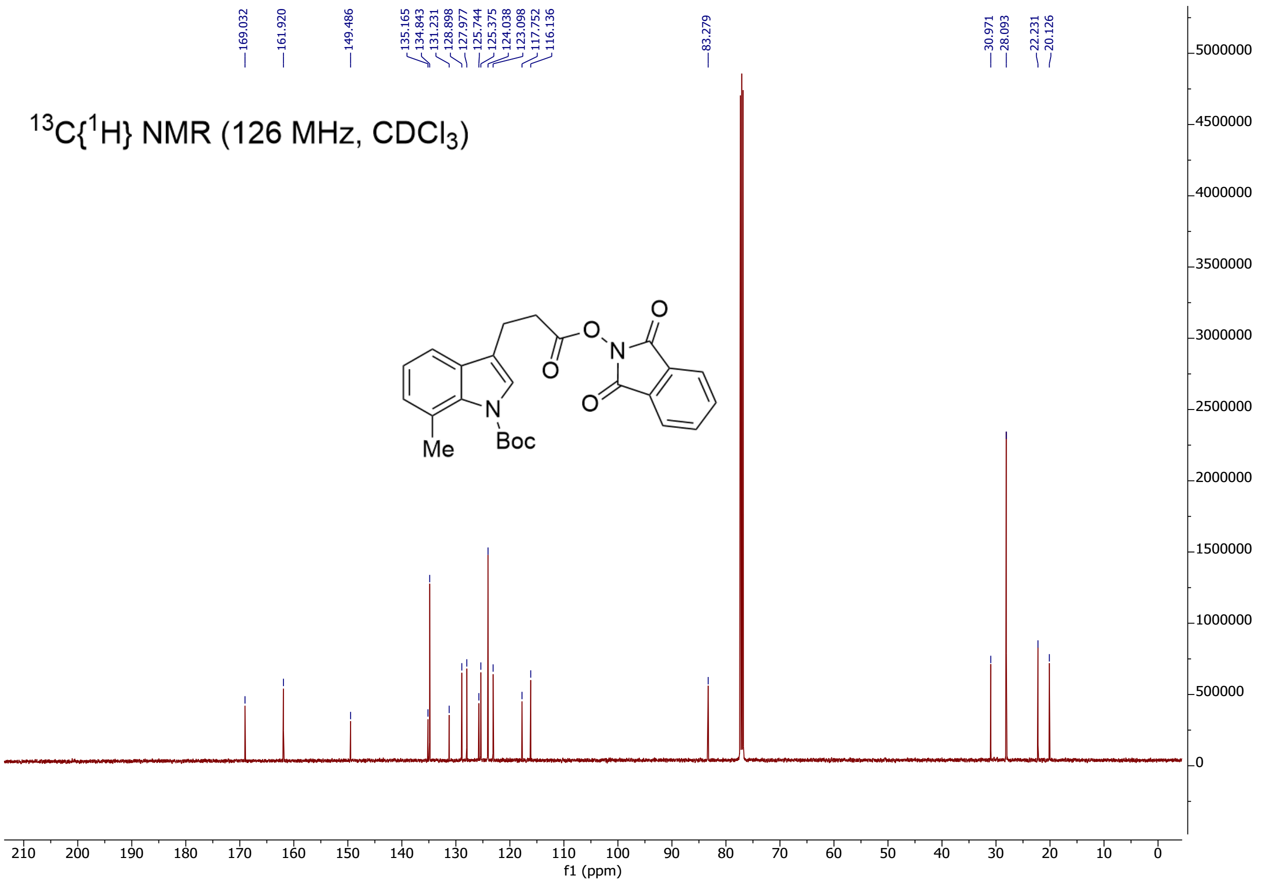
169.032
161.920
149.486
135.165
134.843
131.231
128.898
127.977
125.744
125.375
124.038
123.098
117.752
116.136
83.279
30.971
28.093
22.231
20.126
$^{13}C\{^1H\}$ NMR (126 MHz, $CDCl_3$)
Me
N
Boc
O
O
N
O
O
f1 (ppm)

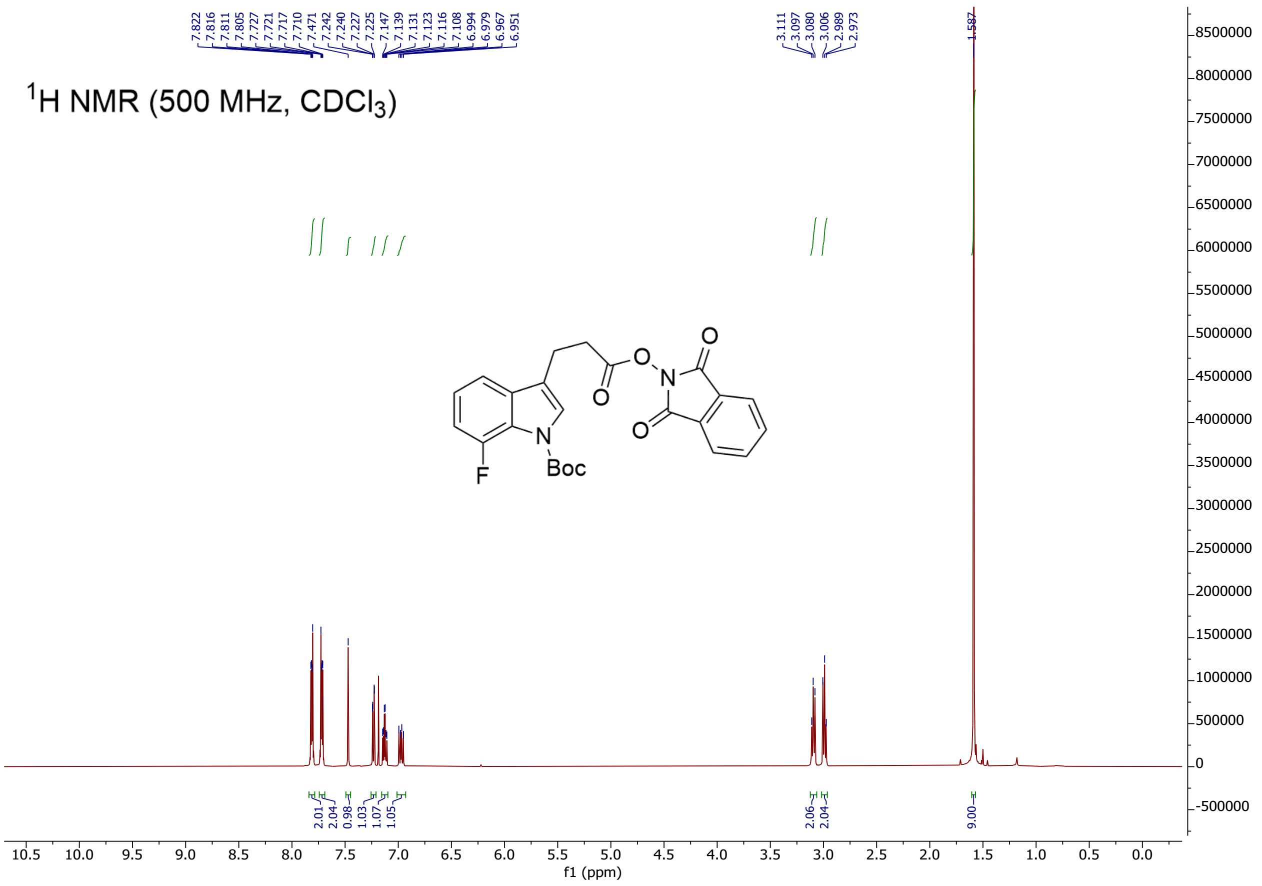
7.822
7.816
7.811
7.805
7.727
7.721
7.717
7.710
7.471
7.242
7.240
7.227
7.225
7.147
7.139
7.131
7.123
7.116
7.108
6.994
6.979
6.967
6.951
3.111
3.097
3.080
3.006
2.989
2.973
1.587
^1H NMR (500 MHz, $CDCl_3$)
F
N
Boc
O
O
N
O
O
2.01
2.04
0.98
1.03
1.07
1.05
2.06
2.04
9.00
f1 (ppm)

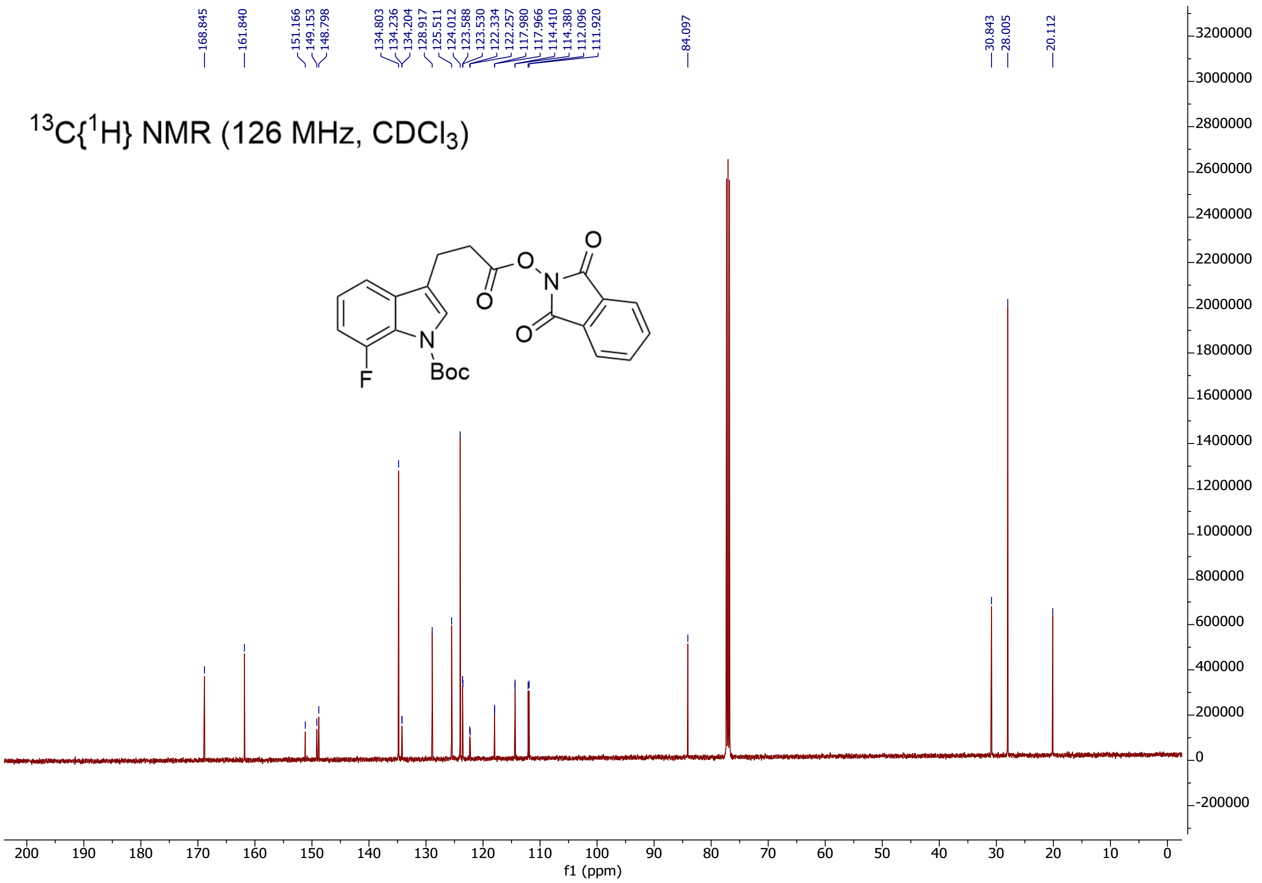

$^{13}C\{^1H\}$ NMR (126 MHz, $CDCl_3$)
168.845
161.840
151.166
149.153
148.798
134.803
134.236
134.204
128.917
125.511
124.012
123.588
123.530
122.334
122.257
117.980
117.966
114.410
114.380
112.096
111.920
84.097
30.843
28.005
20.112
F
N
Boc
O
N
f1 (ppm)

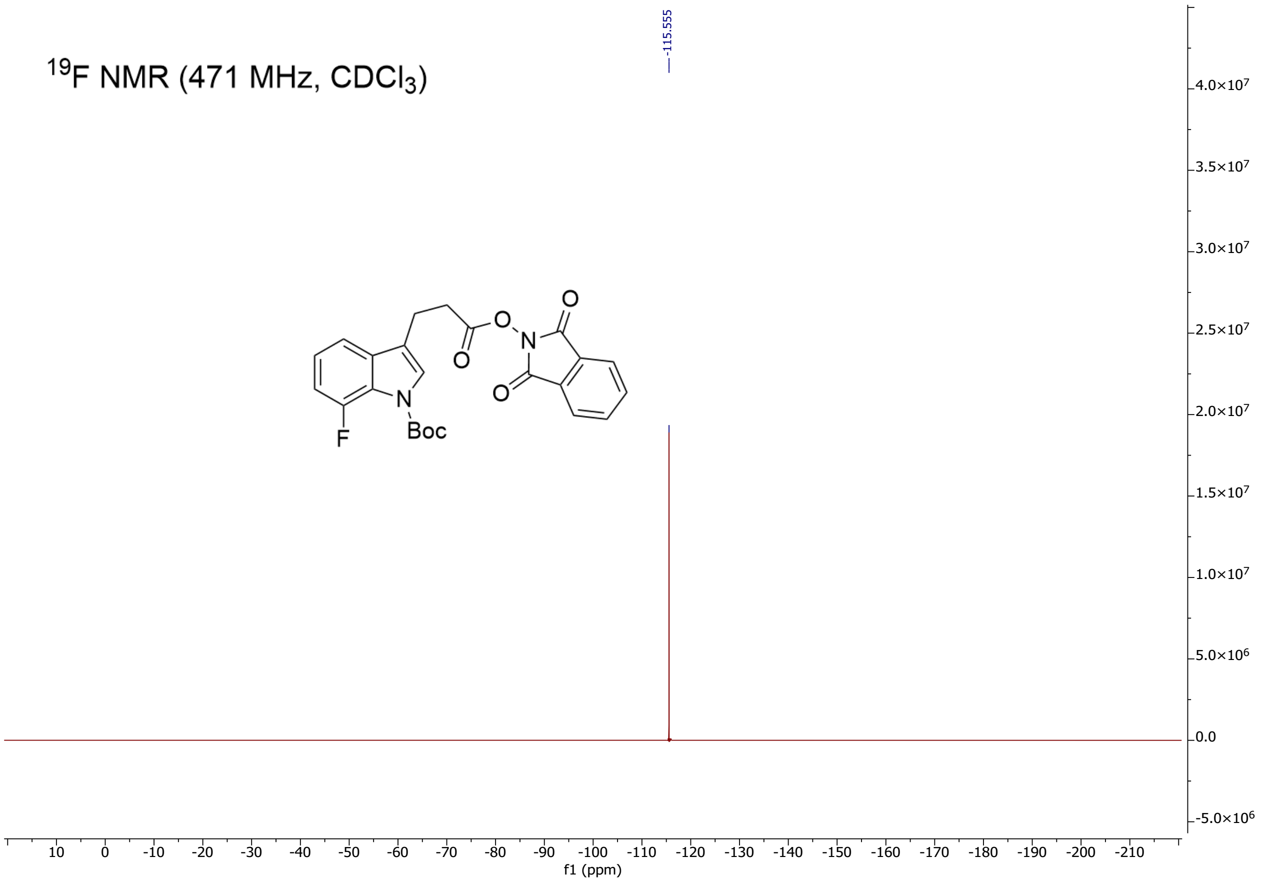

^{19}F NMR (471 MHz, $CDCl_3$)
-115.555
F
N
Boc
O
N
f1 (ppm)

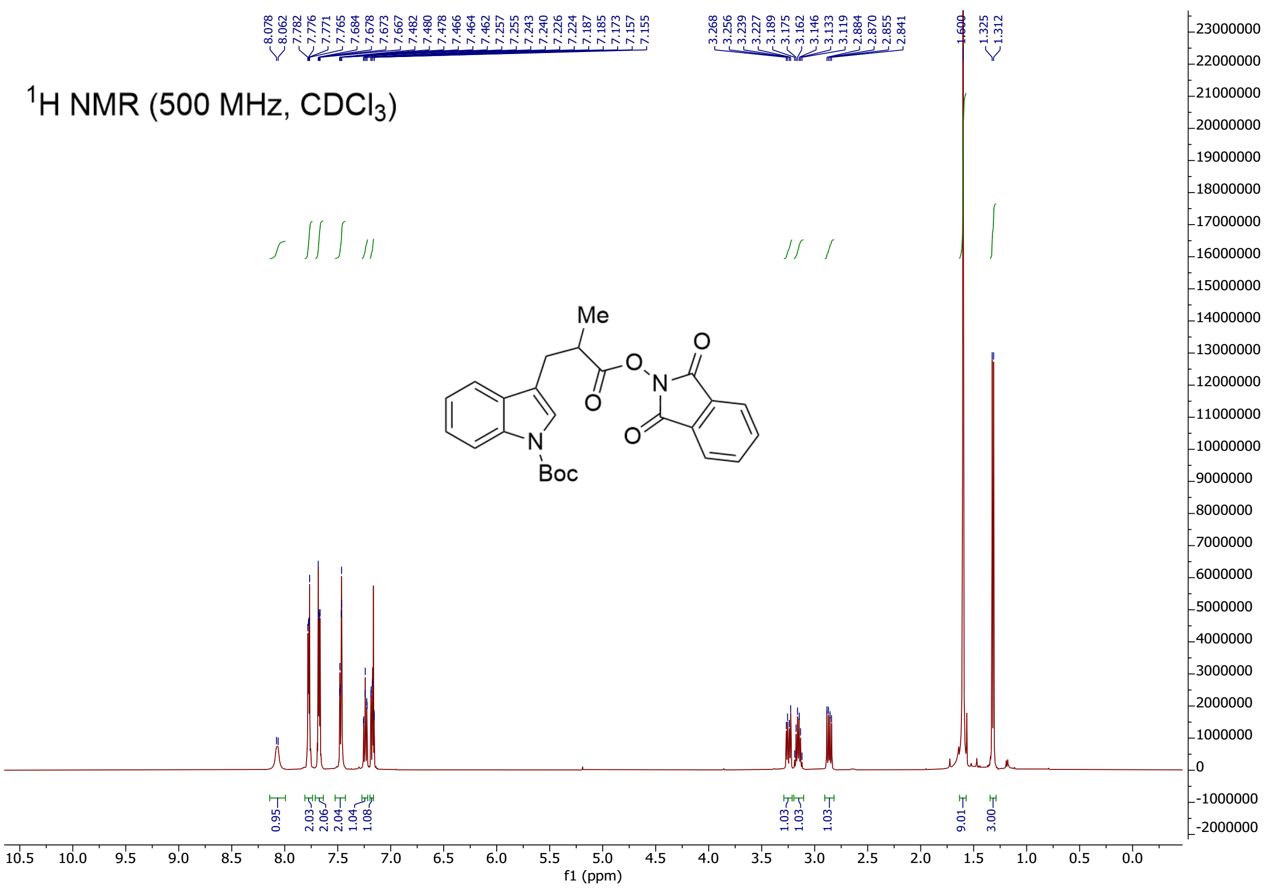
1H NMR (500 MHz, CDCl3)
Me
O
O
N
O
O
N
Boc
f1 (ppm)

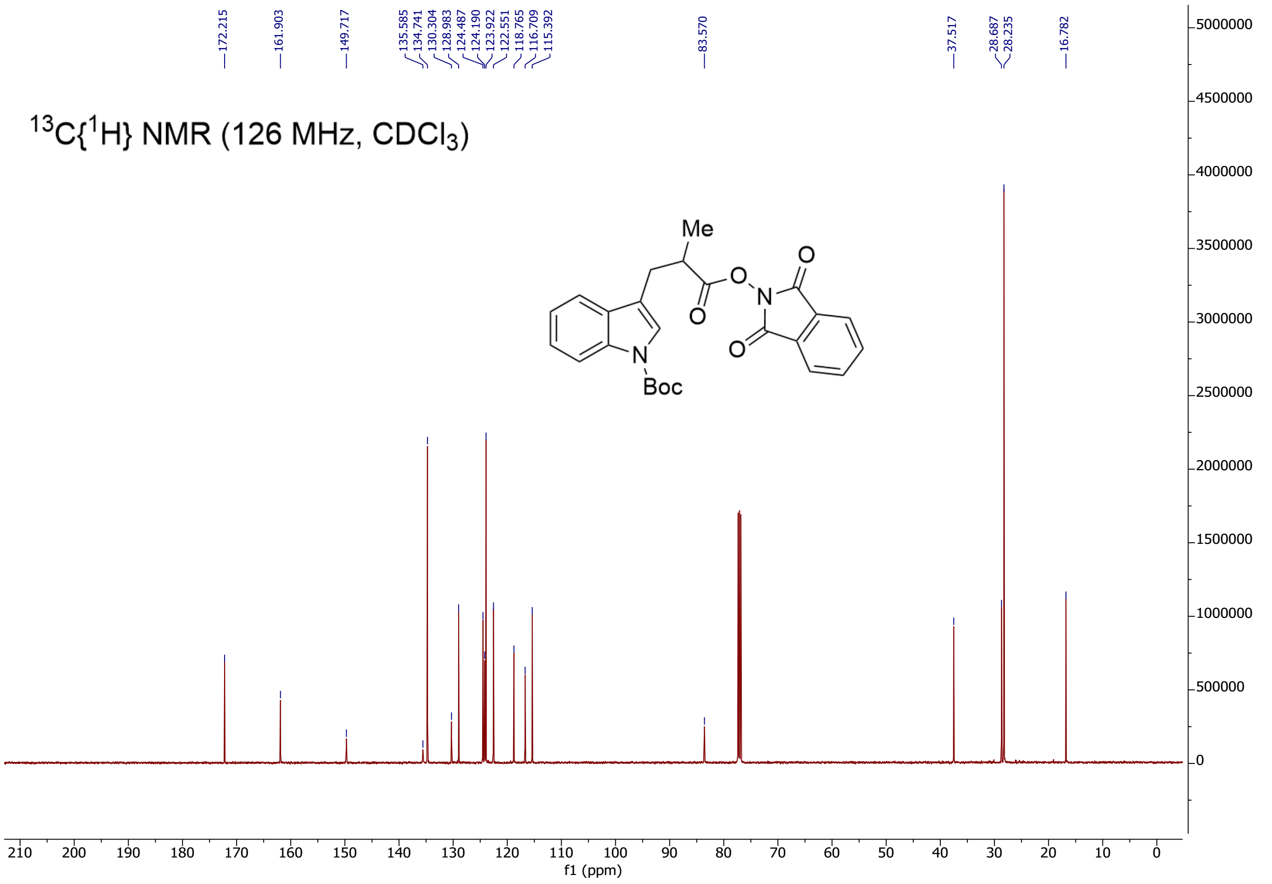
13C{1H} NMR (126 MHz, CDCl3)
172.215
161.903
149.717
83.570
37.517
28.687
28.235
16.782
Me
N
Boc
f1 (ppm)

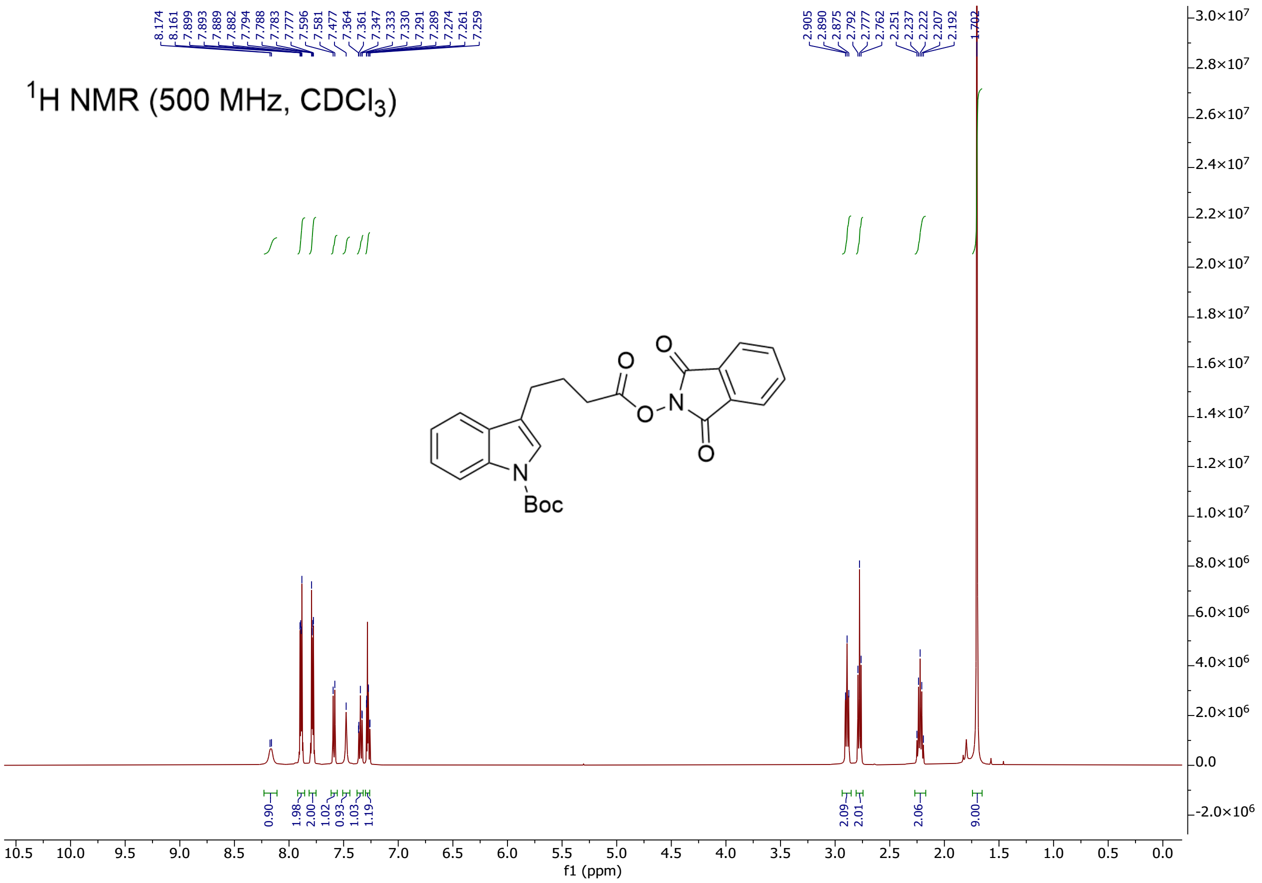

8.174 8.161 7.899 7.893 7.889 7.882 7.794 7.788 7.783 7.777 7.596 7.581 7.477 7.364 7.361 7.347 7.333 7.330 7.291 7.289 7.274 7.261 7.259
2.905 2.890 2.875 2.792 2.777 2.762 2.251 2.237 2.222 2.207 2.192 1.702
¹H NMR (500 MHz, CDCl₃)
Boc
0.90 1.98 2.00 1.02 0.93 1.03 1.19 2.09 2.01 2.06 9.00
f1 (ppm)

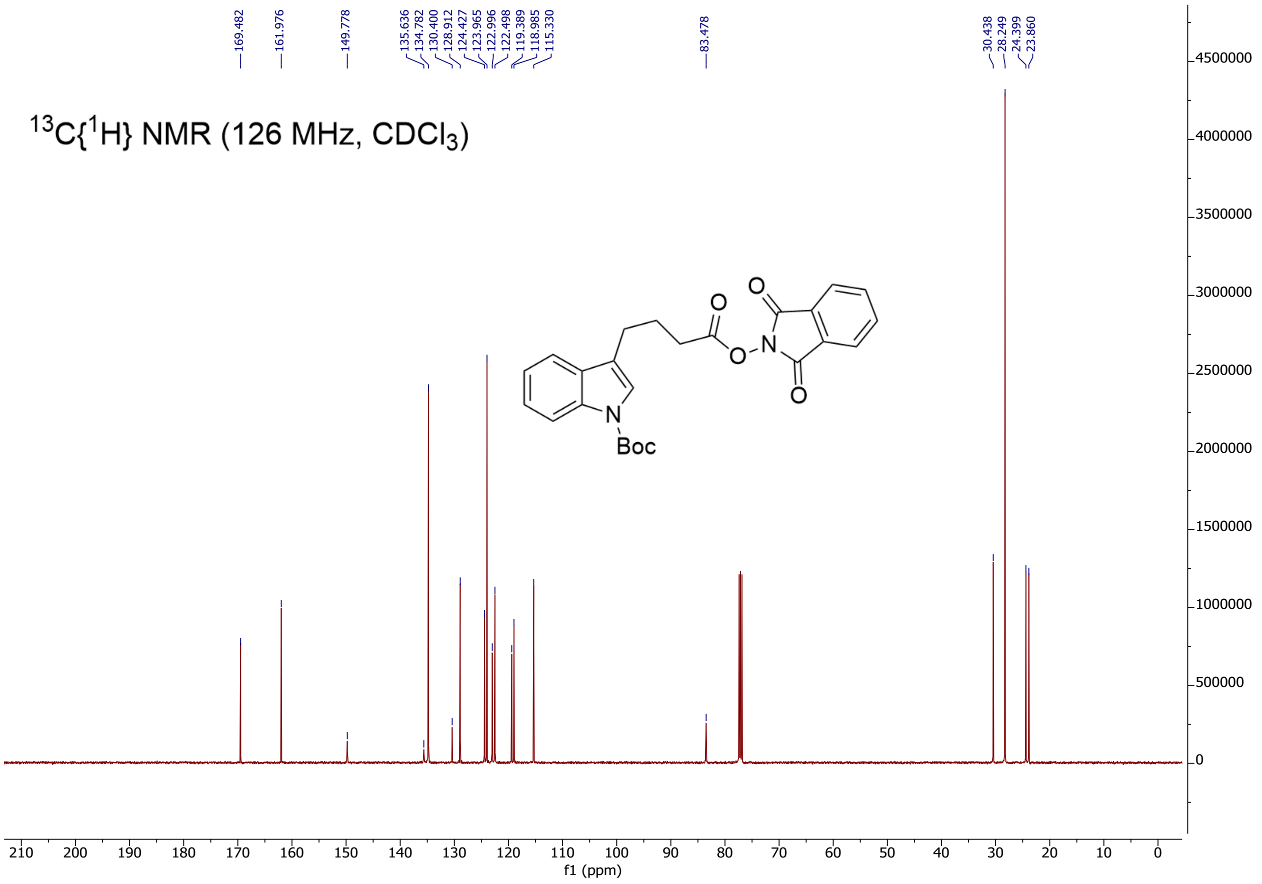

169.482 161.976 149.778 135.636 134.782 130.400 128.912 124.427 123.965 122.996 122.498 119.389 118.985 115.330 83.478 30.438 28.249 24.399 23.860
¹³C{¹H} NMR (126 MHz, CDCl₃)
Boc
f1 (ppm)

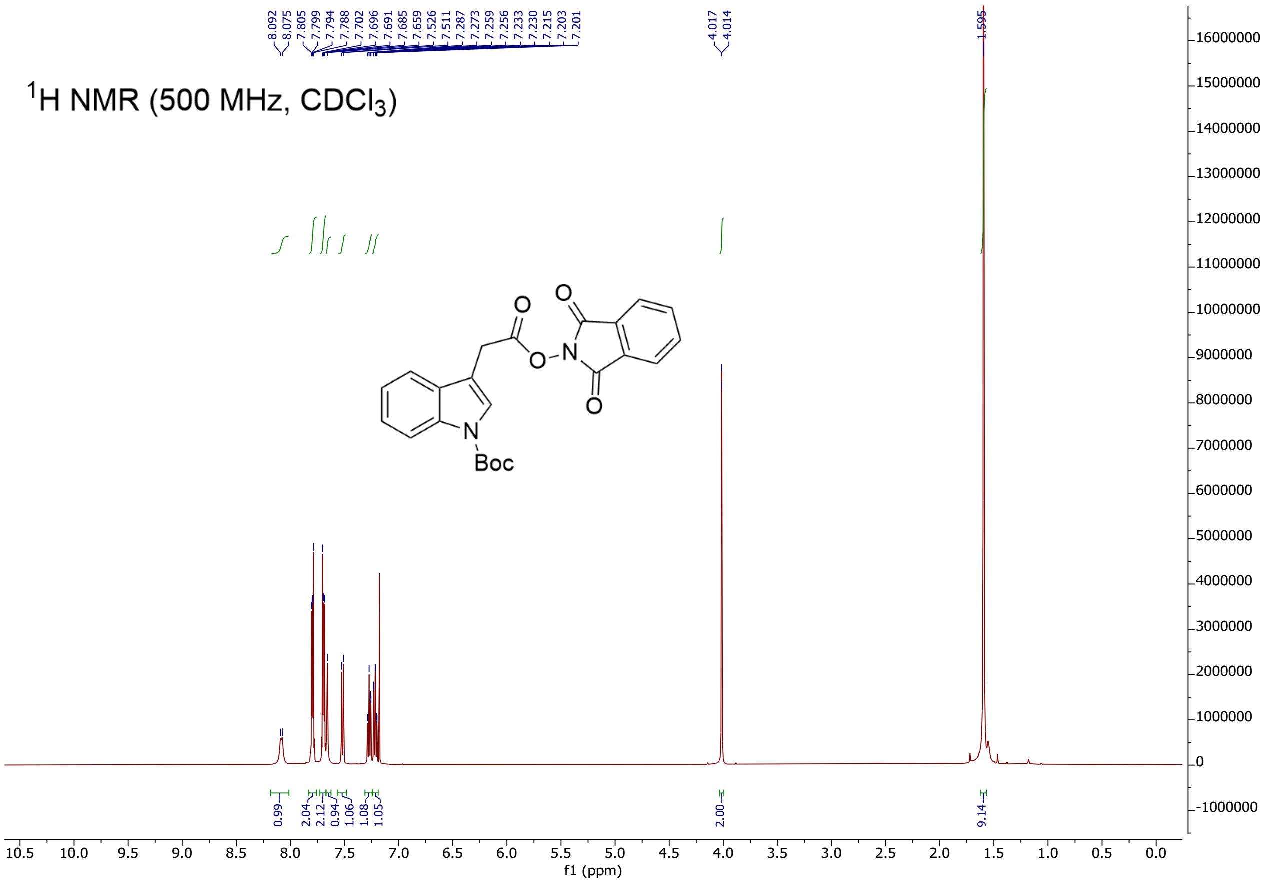

1H NMR (500 MHz, CDCl3)
Boc
f1 (ppm)

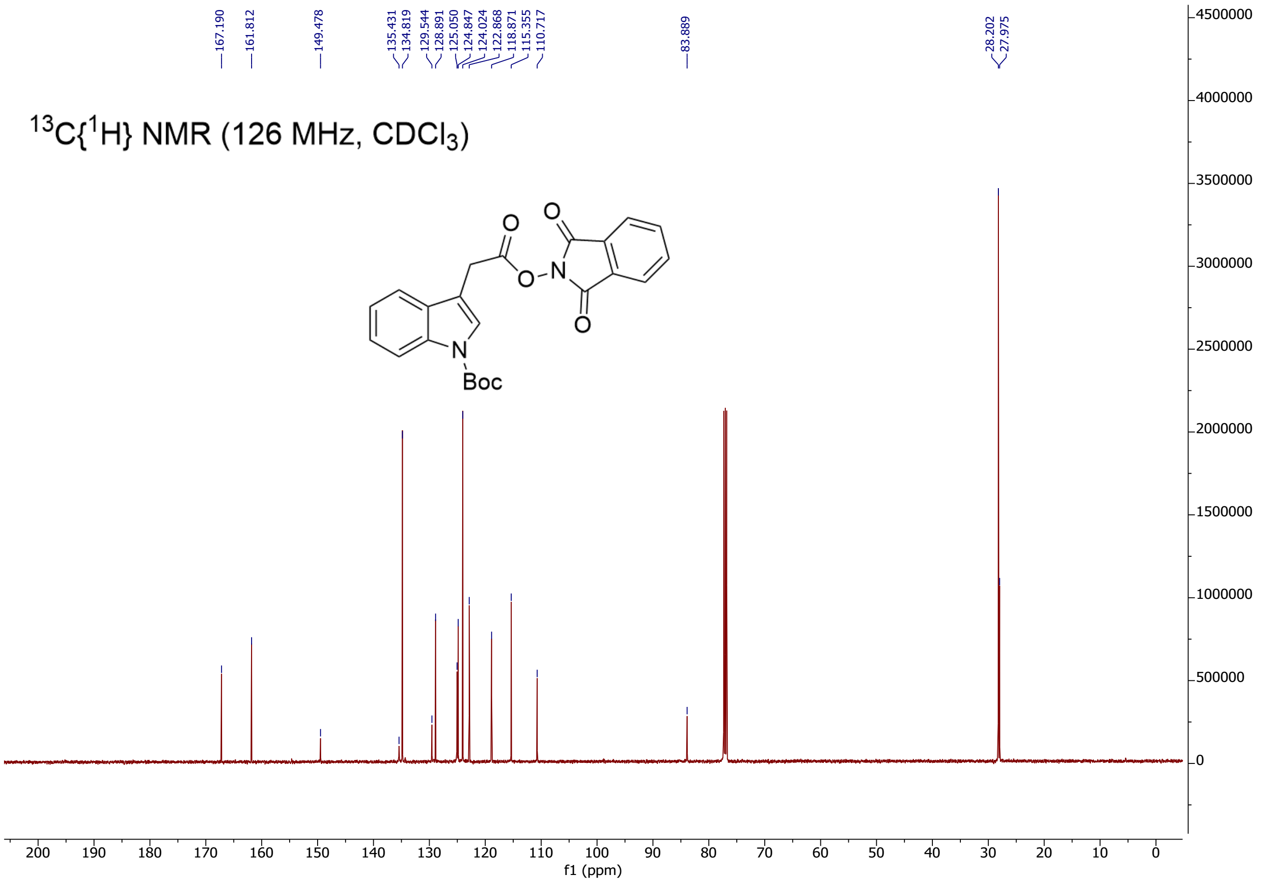

13C{1H} NMR (126 MHz, CDCl3)
Boc
f1 (ppm)

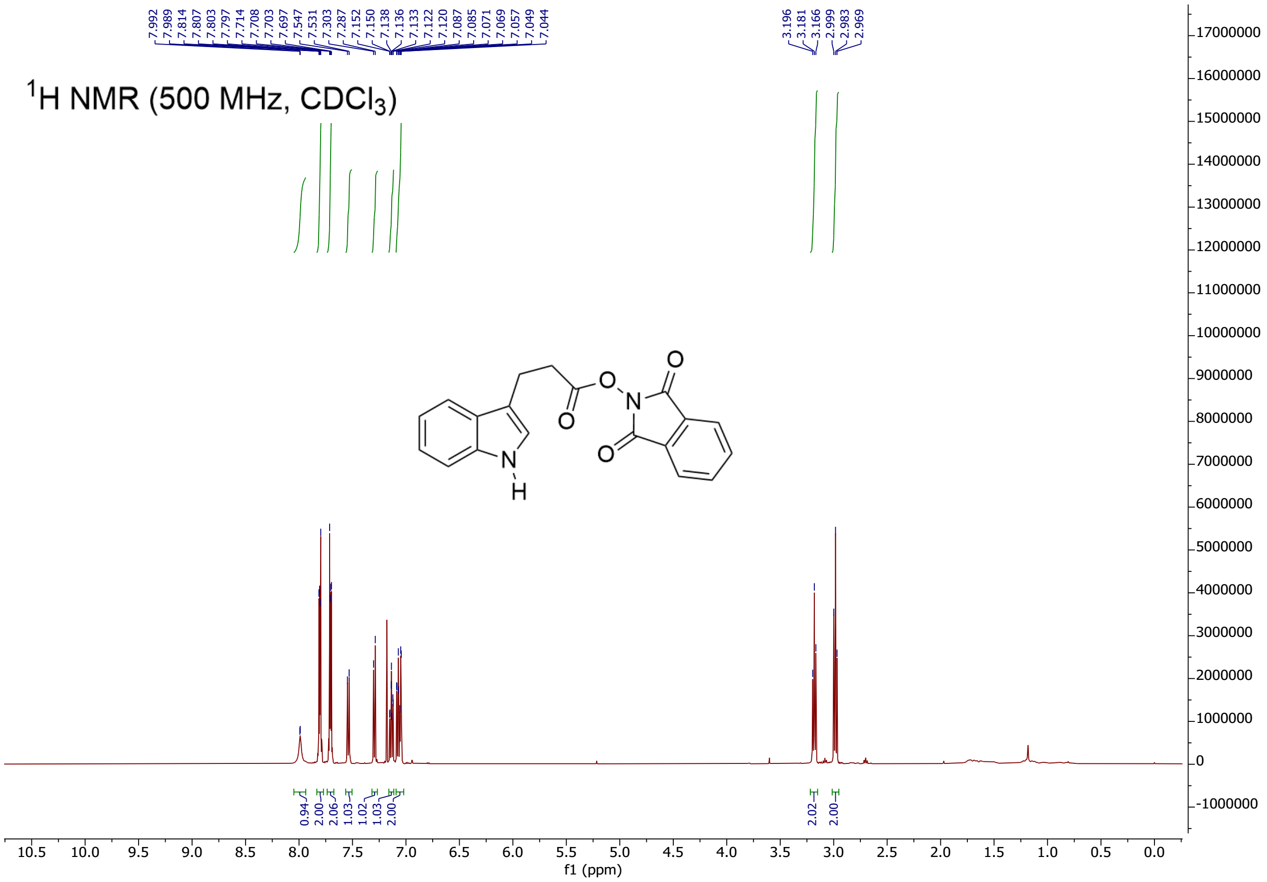
1H NMR (500 MHz, CDCl3)
7.992 7.989 7.814 7.807 7.803 7.797 7.714 7.708 7.703 7.697 7.547 7.531 7.303 7.287 7.152 7.150 7.138 7.136 7.133 7.122 7.120 7.087 7.085 7.071 7.069 7.057 7.049 7.044
3.196 3.181 3.166 2.999 2.983 2.969
0.94 2.00 2.06 1.03 1.02 1.03 2.00
2.02 2.00
O
O
N
O
O
O
N
H
f1 (ppm)

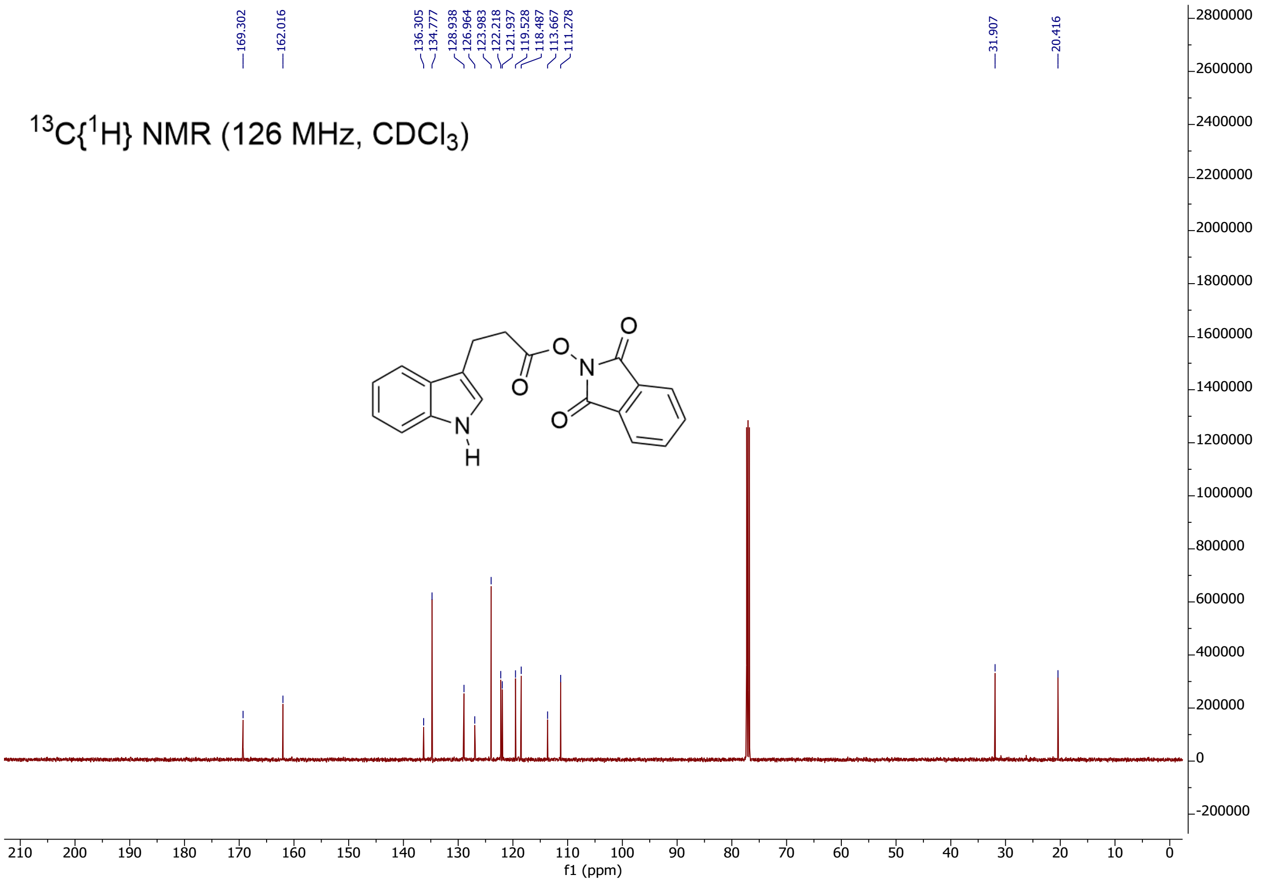
13C{1H} NMR (126 MHz, CDCl3)
169.302
162.016
136.305 134.777 128.938 126.964 123.983 122.218 121.937 119.528 118.487 113.667 111.278
31.907
20.416
O
O
N
O
O
O
N
H
f1 (ppm)

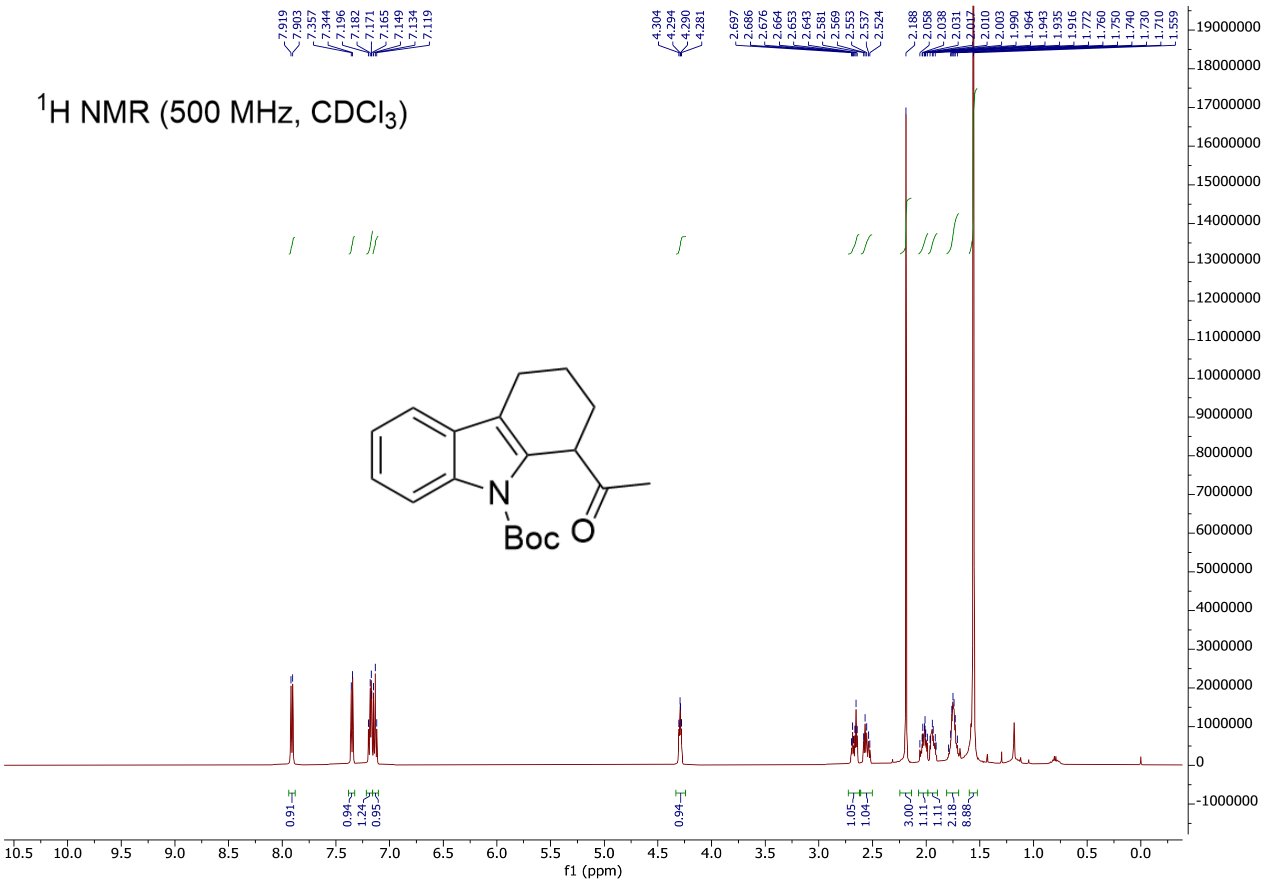
1H NMR (500 MHz, CDCl3)
7.919 7.903 7.357 7.344 7.196 7.182 7.171 7.165 7.149 7.134 7.119
4.304 4.294 4.290 4.281
2.697 2.686 2.676 2.664 2.653 2.643 2.581 2.569 2.553 2.537 2.524
2.188 2.058 2.038 2.031 2.017 2.010 2.003 1.990 1.964 1.943 1.935 1.916 1.772 1.760 1.750 1.740 1.730 1.710 1.559
N
Boc
O
0.91 0.94 1.24 0.95 0.94 1.05 1.04 3.00 1.11 1.11 2.18 8.88
f1 (ppm)

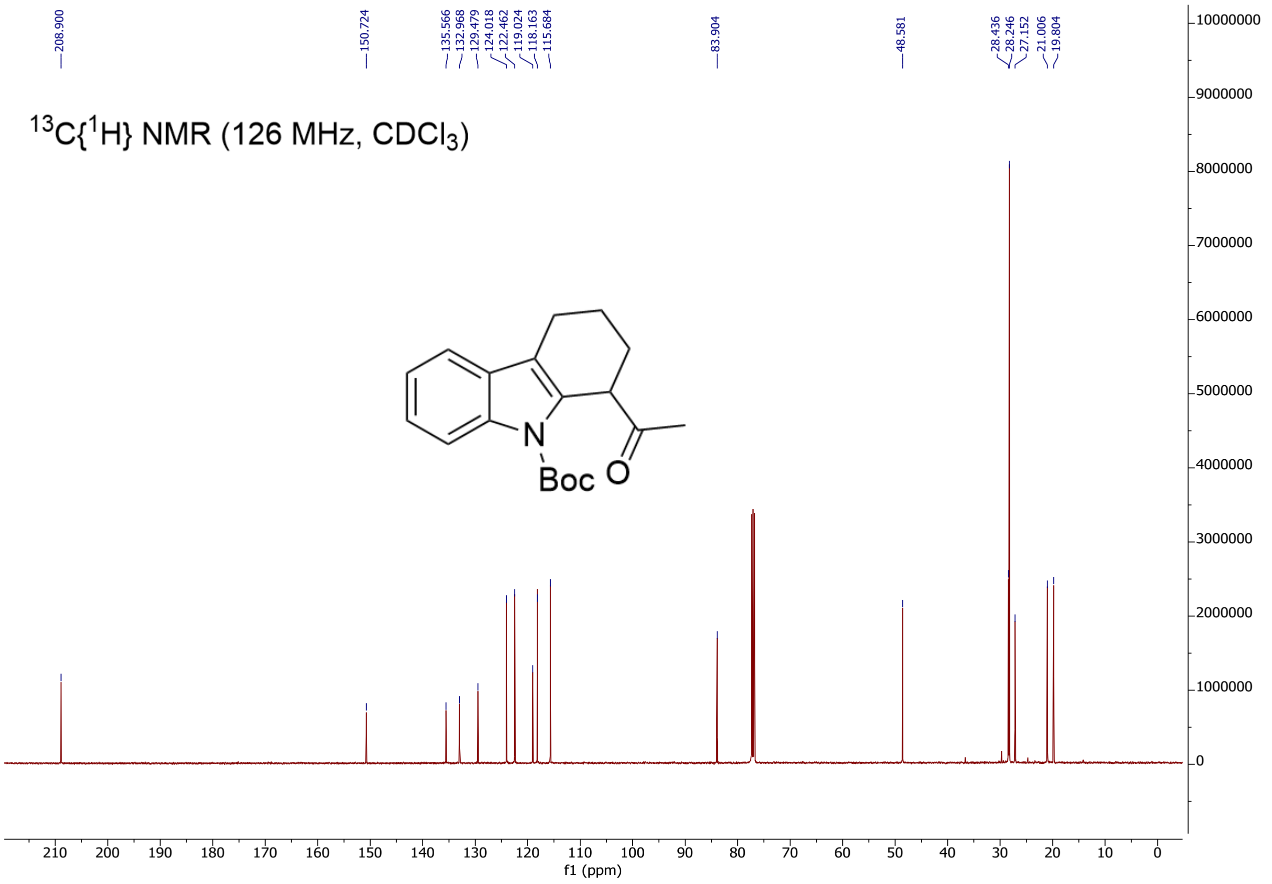
13C{1H} NMR (126 MHz, CDCl3)
208.900
150.724
135.566 132.968 129.479 124.018 122.462 119.024 118.163 115.684
83.904
48.581
28.436 28.246 27.152 21.006 19.804
N
Boc
O
f1 (ppm)

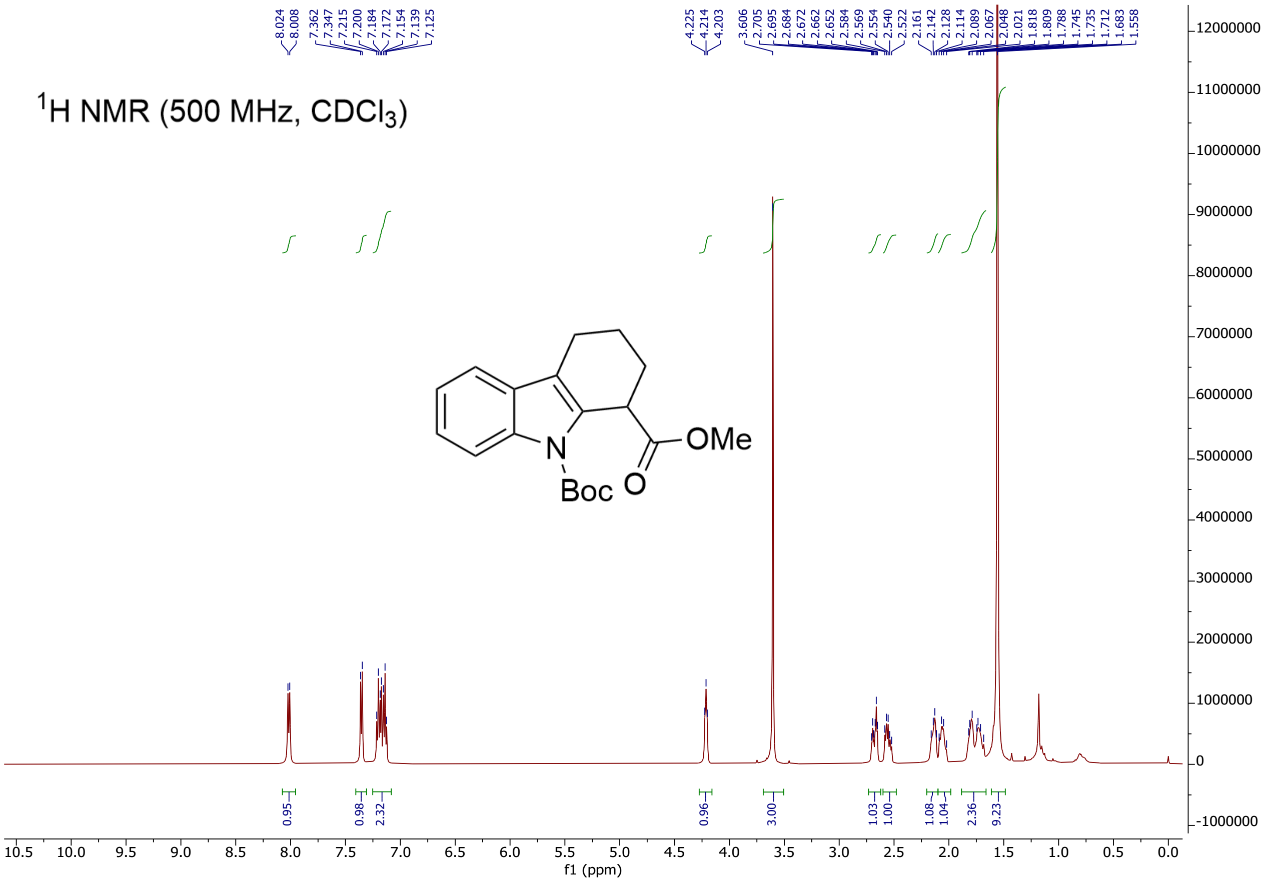

1H NMR (500 MHz, CDCl3)
OMe
N
Boc
O
f1 (ppm)

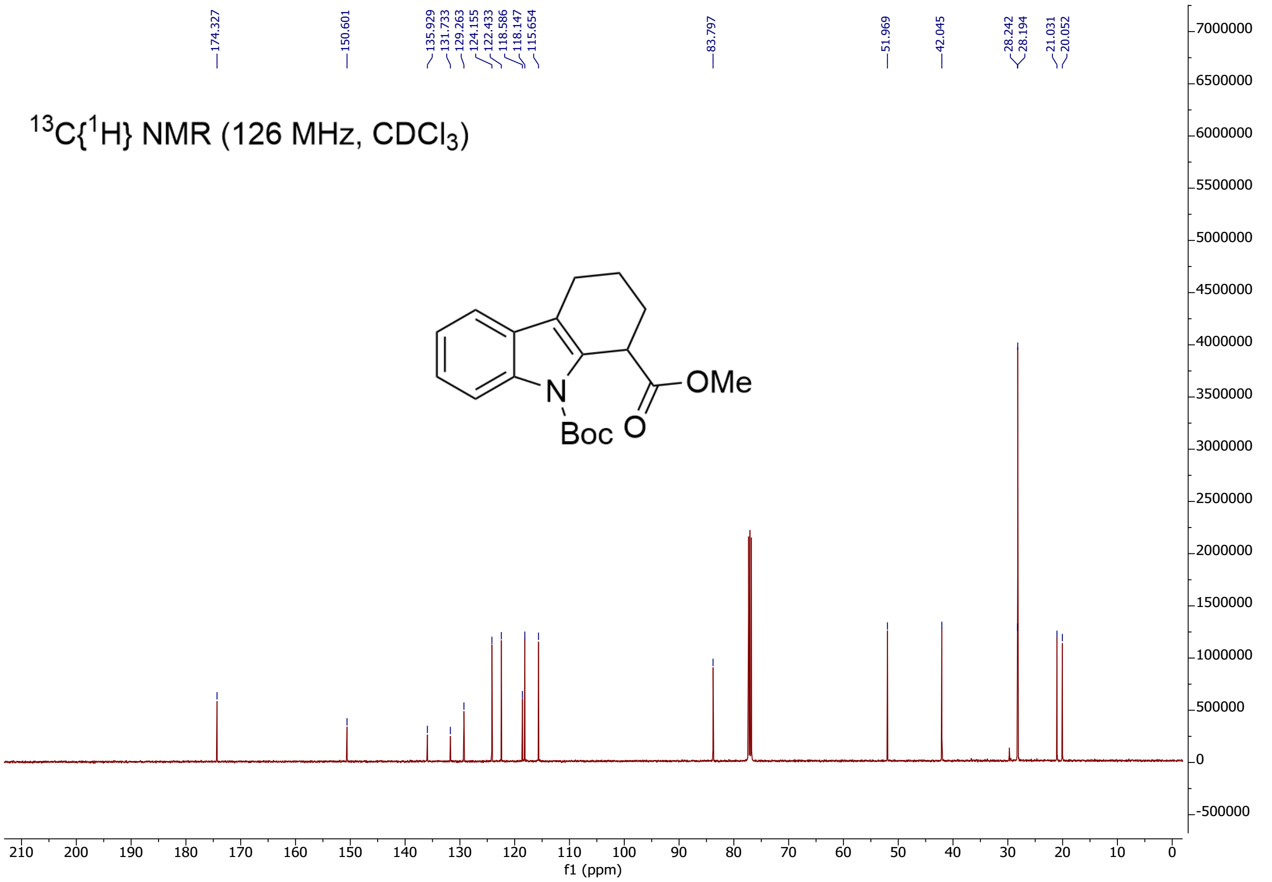

13C{1H} NMR (126 MHz, CDCl3)
OMe
N
Boc
O
f1 (ppm)

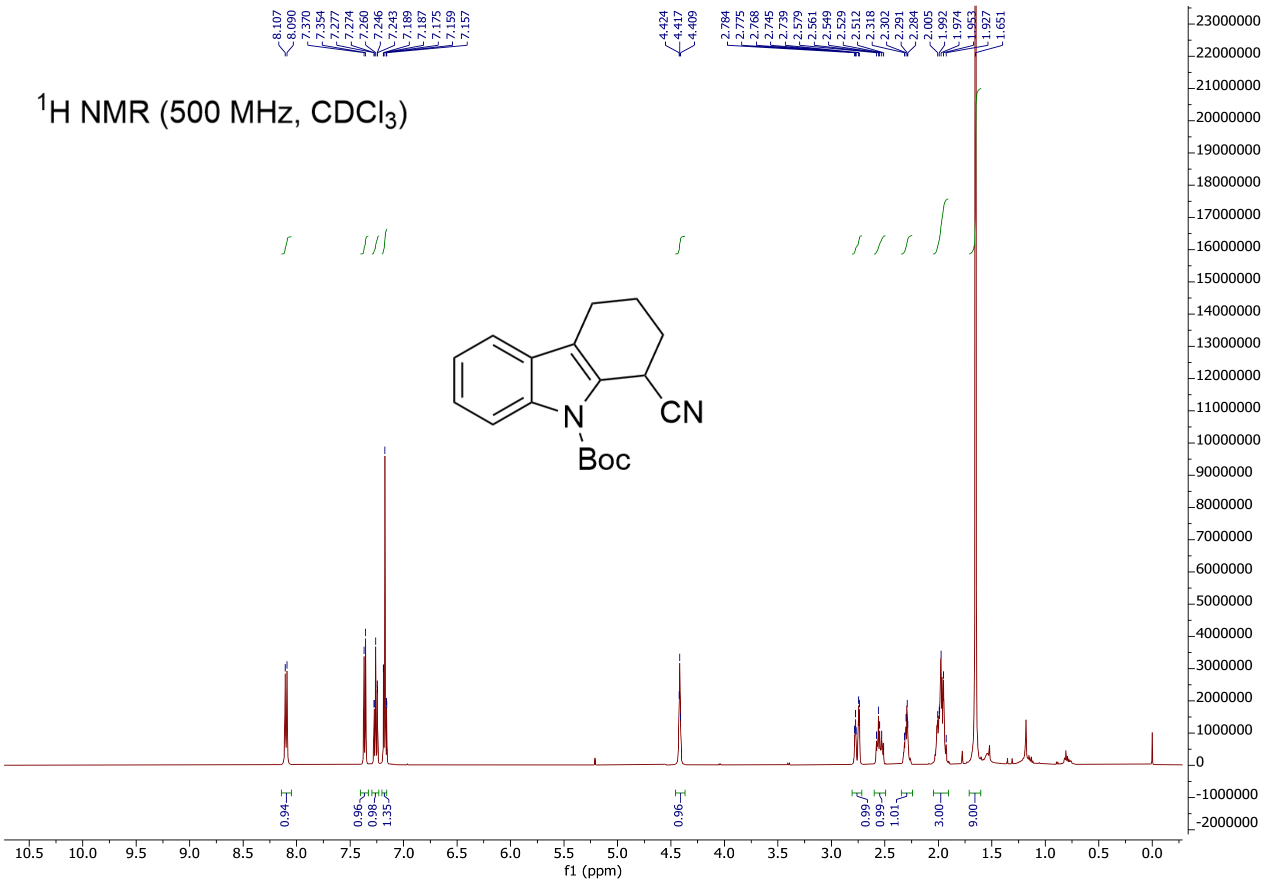

1H NMR (500 MHz, CDCl3)
8.107
8.090
7.370
7.354
7.277
7.274
7.260
7.246
7.243
7.189
7.187
7.175
7.159
7.157
4.424
4.417
4.409
2.784
2.775
2.768
2.745
2.739
2.579
2.561
2.549
2.529
2.512
2.318
2.302
2.291
2.284
2.005
1.992
1.974
1.953
1.927
1.651
0.94
0.96
0.98
1.35
0.96
0.99
0.99
1.01
3.00
9.00
CN
N
Boc
f1 (ppm)

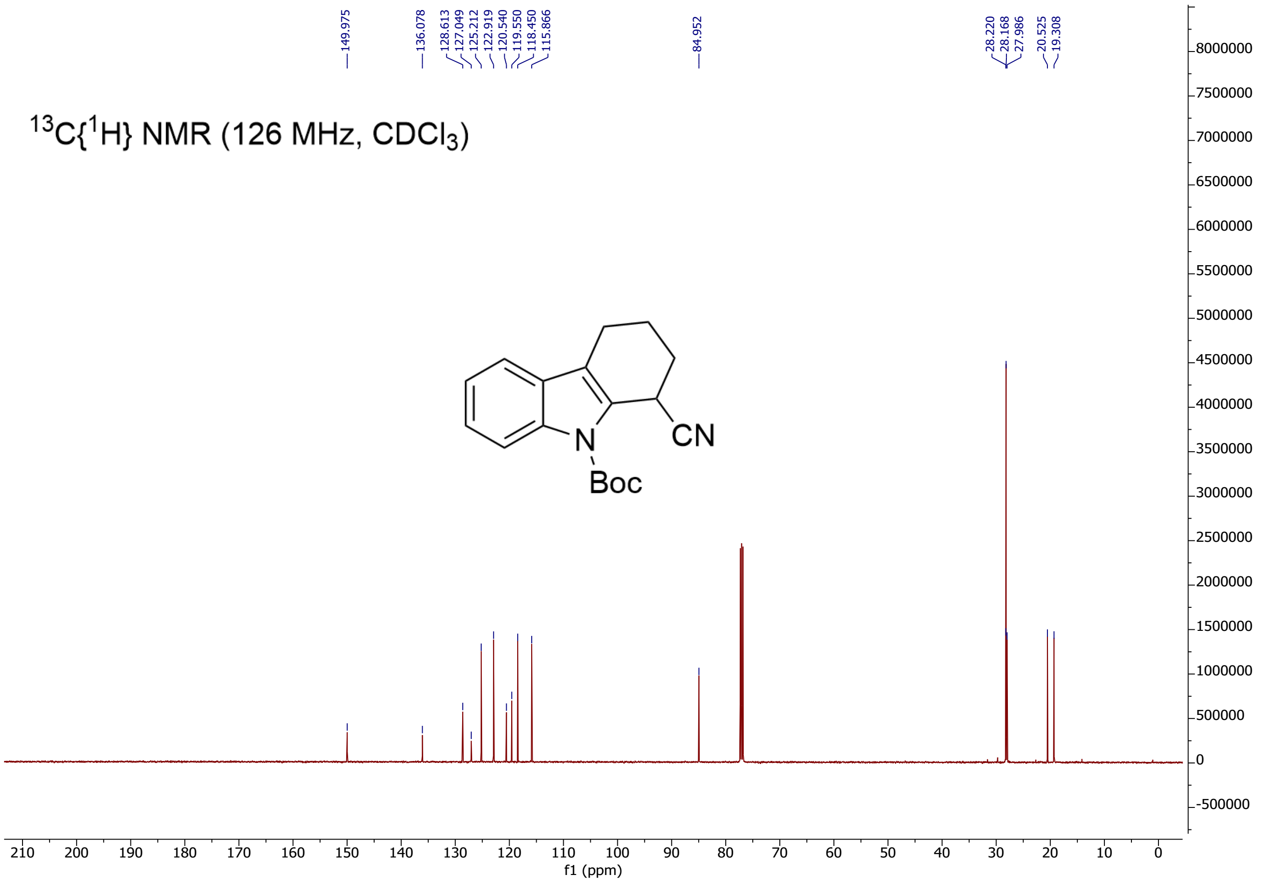

13C{1H} NMR (126 MHz, CDCl3)
149.975
136.078
128.613
127.049
125.212
122.919
120.540
119.550
118.450
115.866
84.952
28.220
28.168
27.986
20.525
19.308
CN
N
Boc
f1 (ppm)

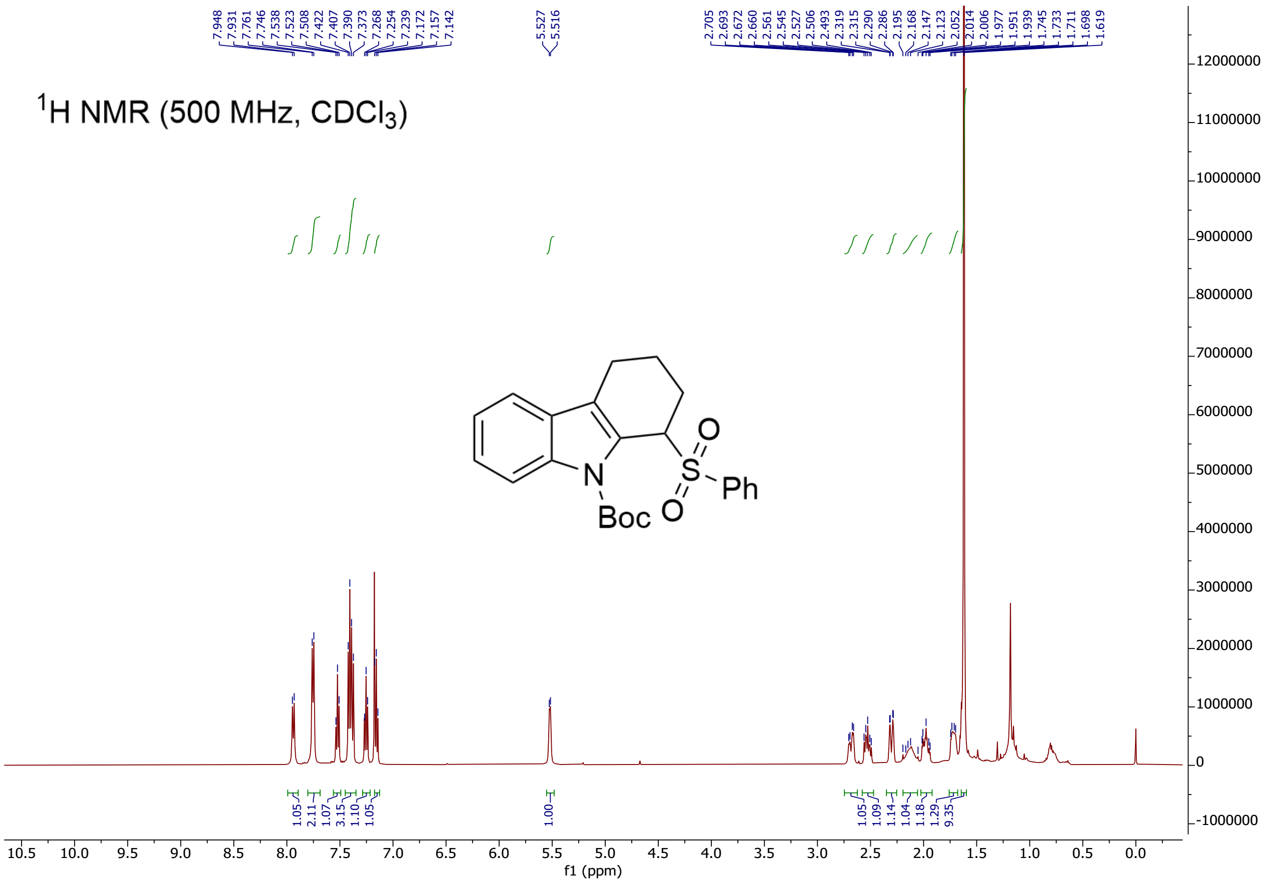

7.948
7.931
7.761
7.746
7.538
7.523
7.508
7.422
7.407
7.390
7.373
7.268
7.254
7.239
7.172
7.157
7.142
5.527
5.516
2.705
2.693
2.672
2.660
2.561
2.545
2.527
2.506
2.493
2.319
2.315
2.290
2.286
2.195
2.168
2.147
2.123
2.052
2.014
2.006
1.977
1.951
1.939
1.745
1.733
1.711
1.698
1.619
^{1}H NMR (500 MHz, CDCl$_3$)
N
Boc
S
O
O
Ph
f1 (ppm)

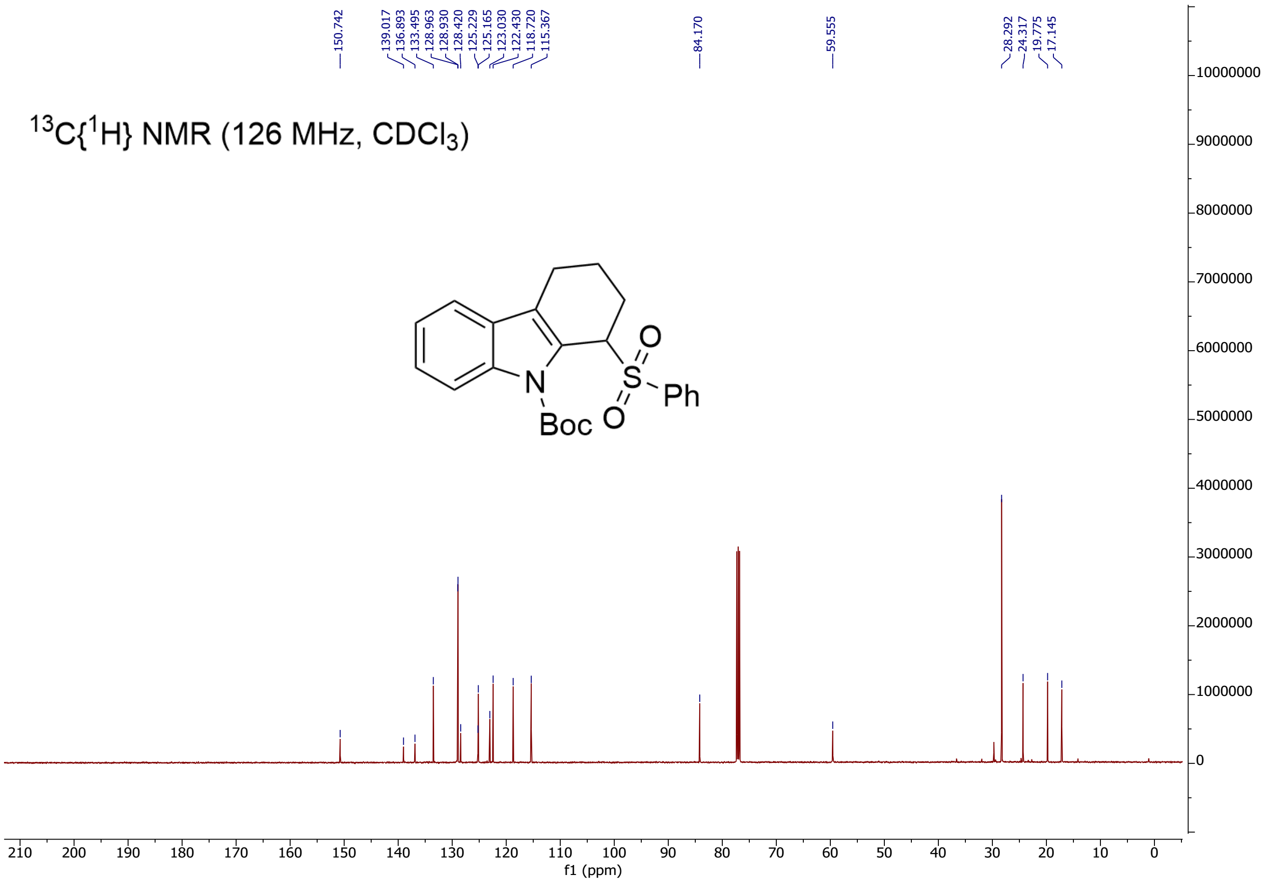

150.742
139.017
136.893
133.495
128.963
128.930
128.420
125.229
125.165
123.030
122.430
118.720
115.367
84.170
59.555
28.292
24.317
19.775
17.145
^{13}C{^{1}H} NMR (126 MHz, CDCl$_3$)
N
Boc
S
O
O
Ph
f1 (ppm)

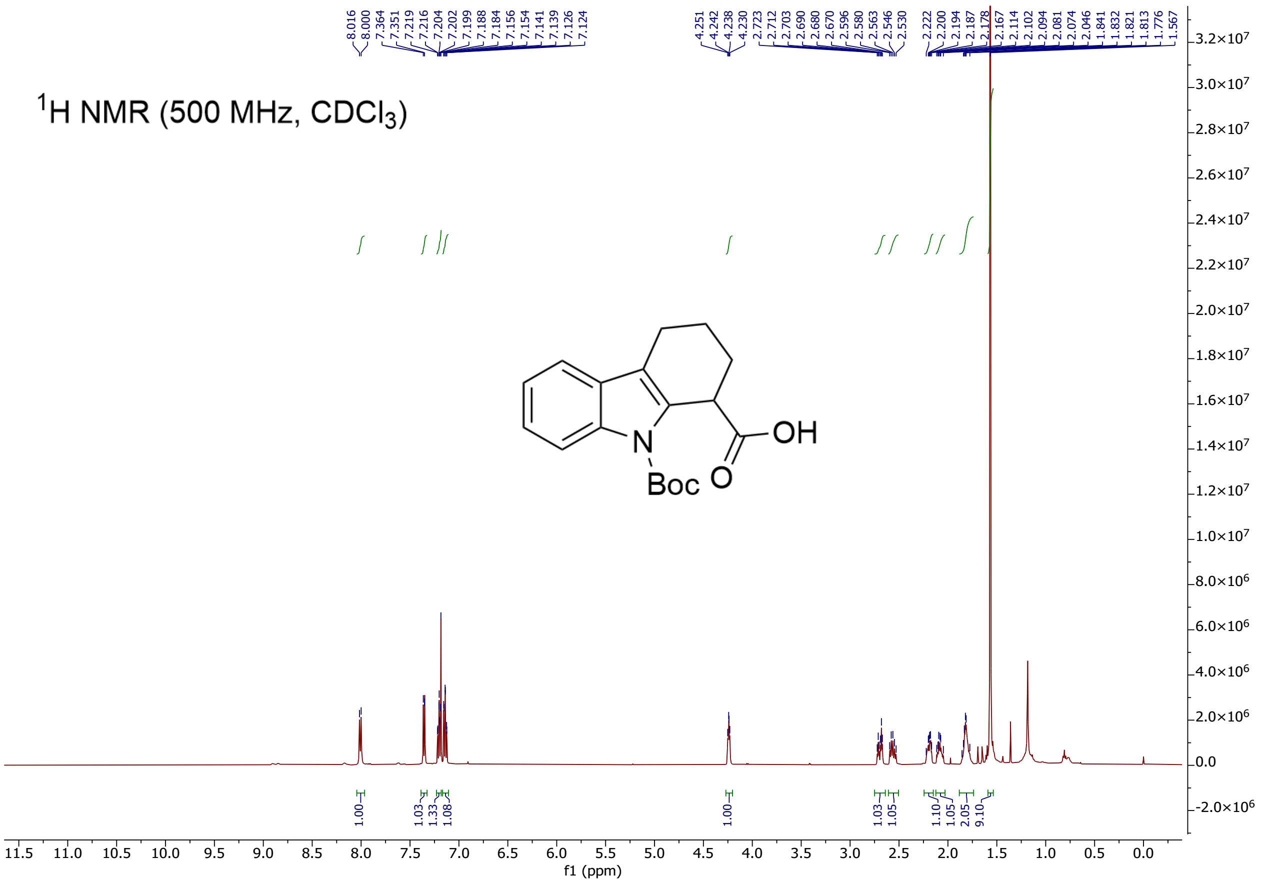

1H NMR (500 MHz, CDCl3)
OH
N
Boc
O
f1 (ppm)

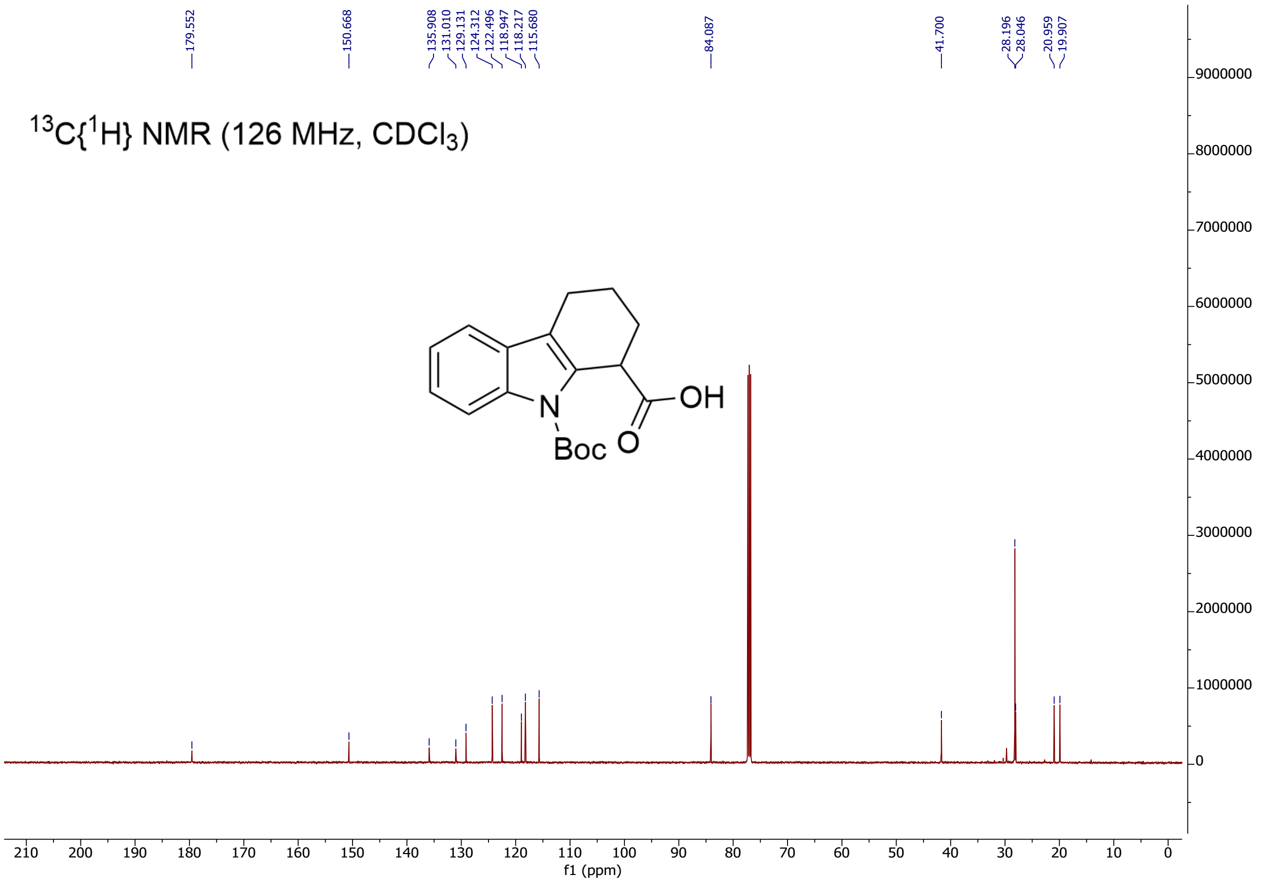

13C{1H} NMR (126 MHz, CDCl3)
OH
N
Boc
O
f1 (ppm)

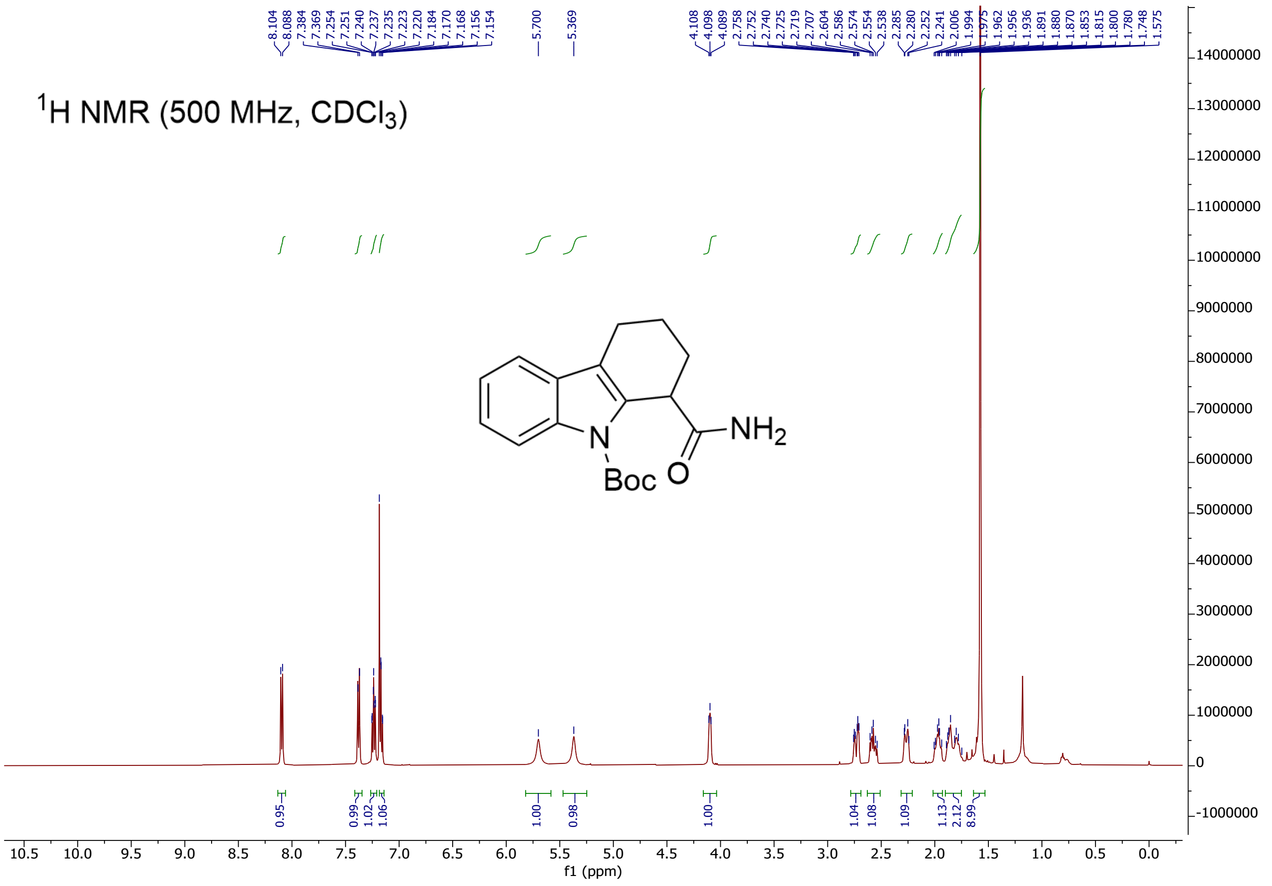

^{1}H NMR (500 MHz, $CDCl_3$)
NH2
Boc
O
N
f1 (ppm)

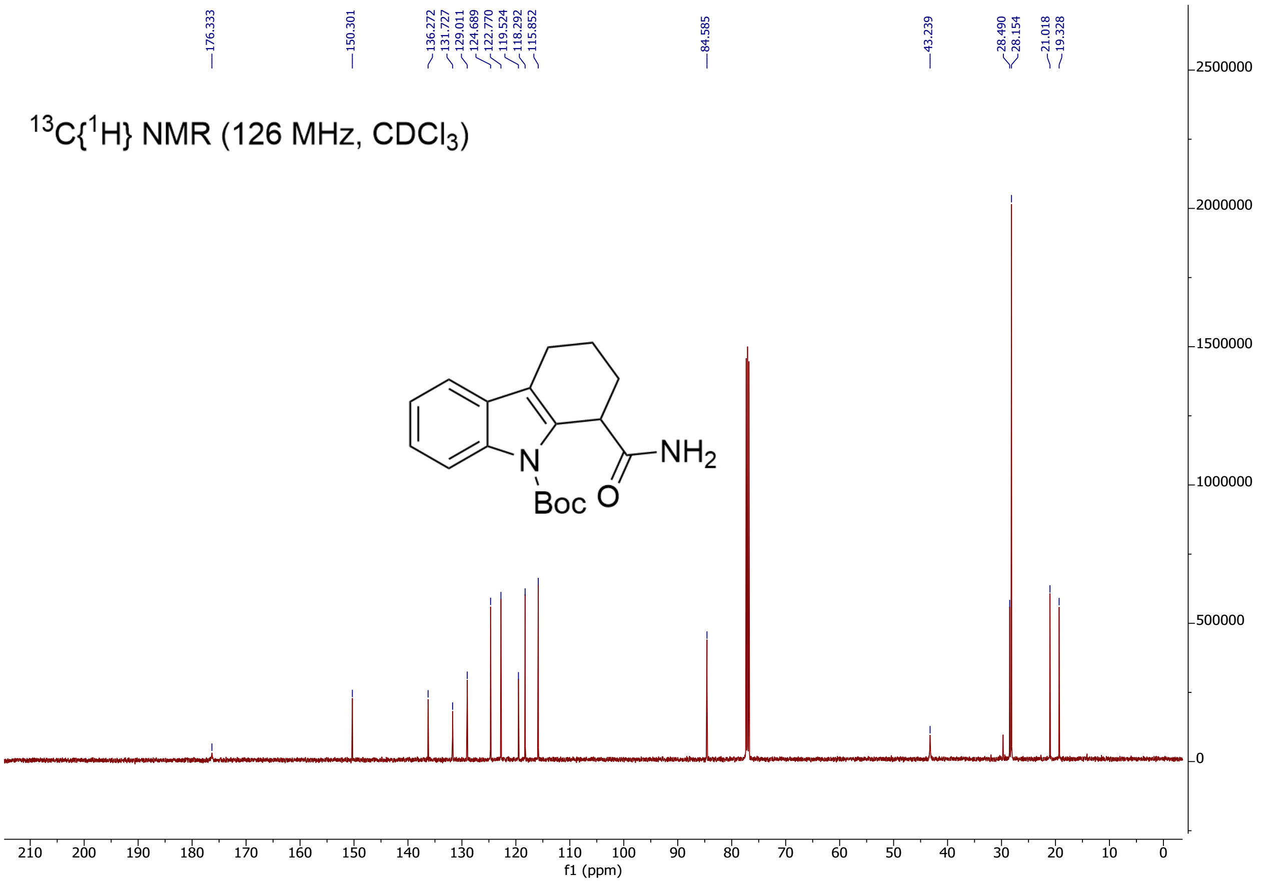

$^{13}C\{^{1}H\}$ NMR (126 MHz, $CDCl_3$)
NH2
Boc
O
N
f1 (ppm)

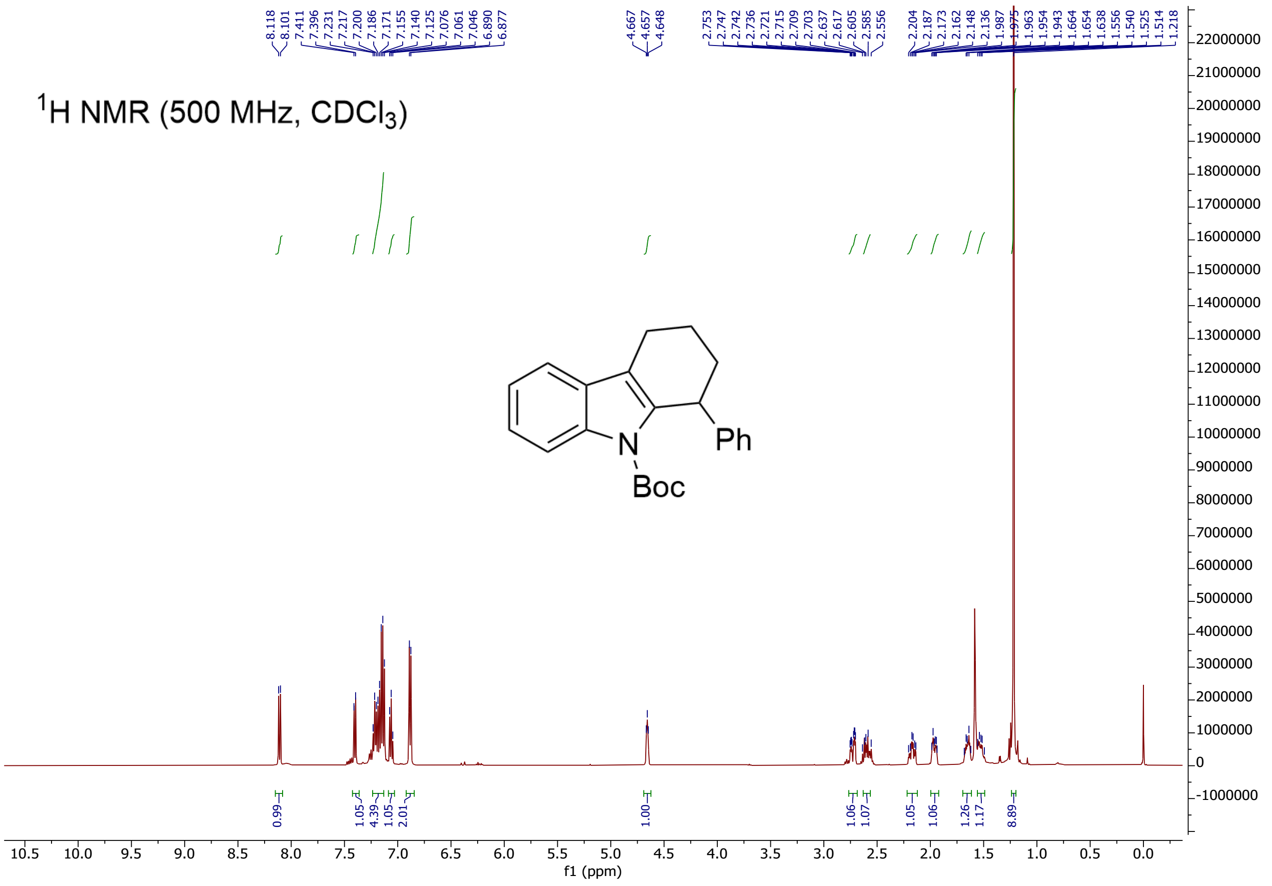

1H NMR (500 MHz, CDCl3)
N
Boc
Ph
f1 (ppm)

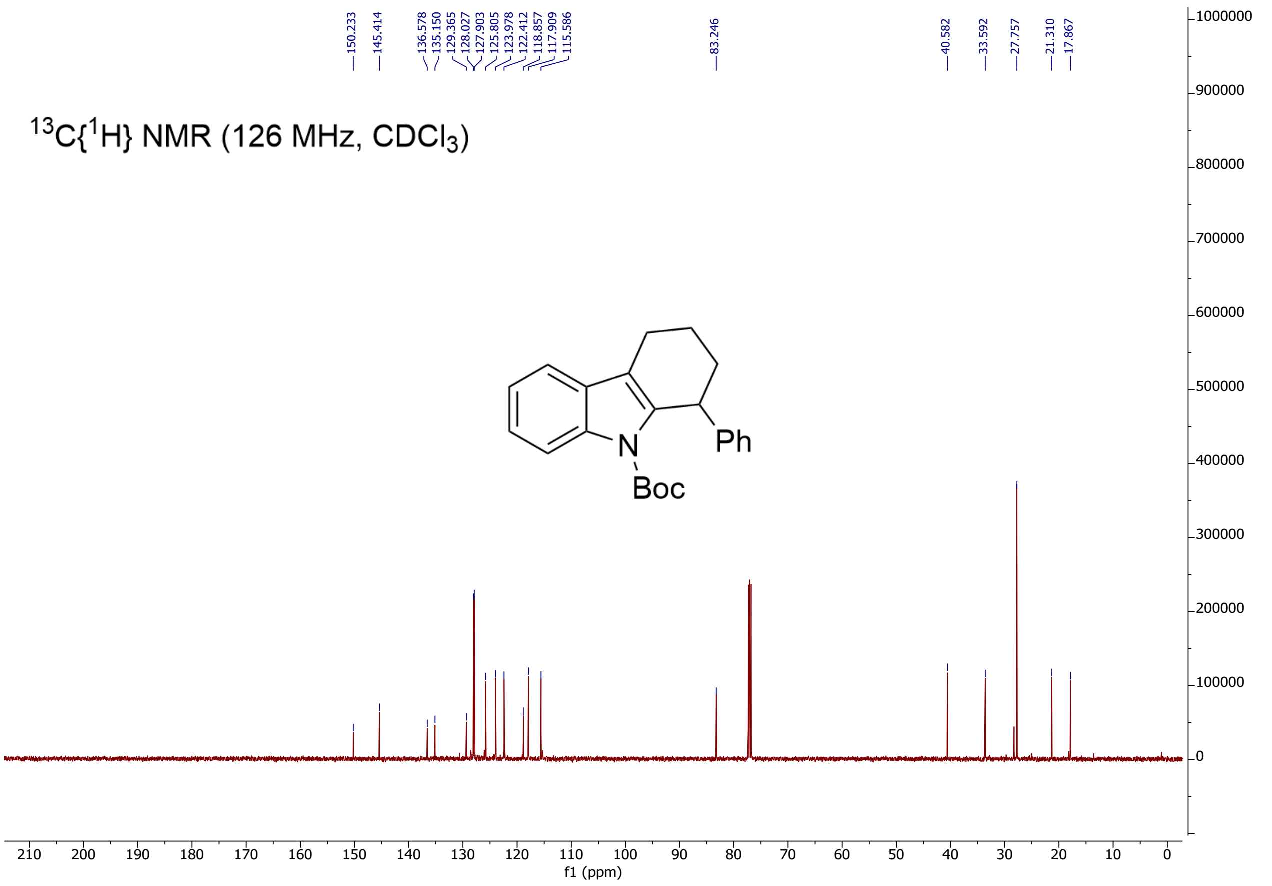

13C{1H} NMR (126 MHz, CDCl3)
N
Boc
Ph
f1 (ppm)

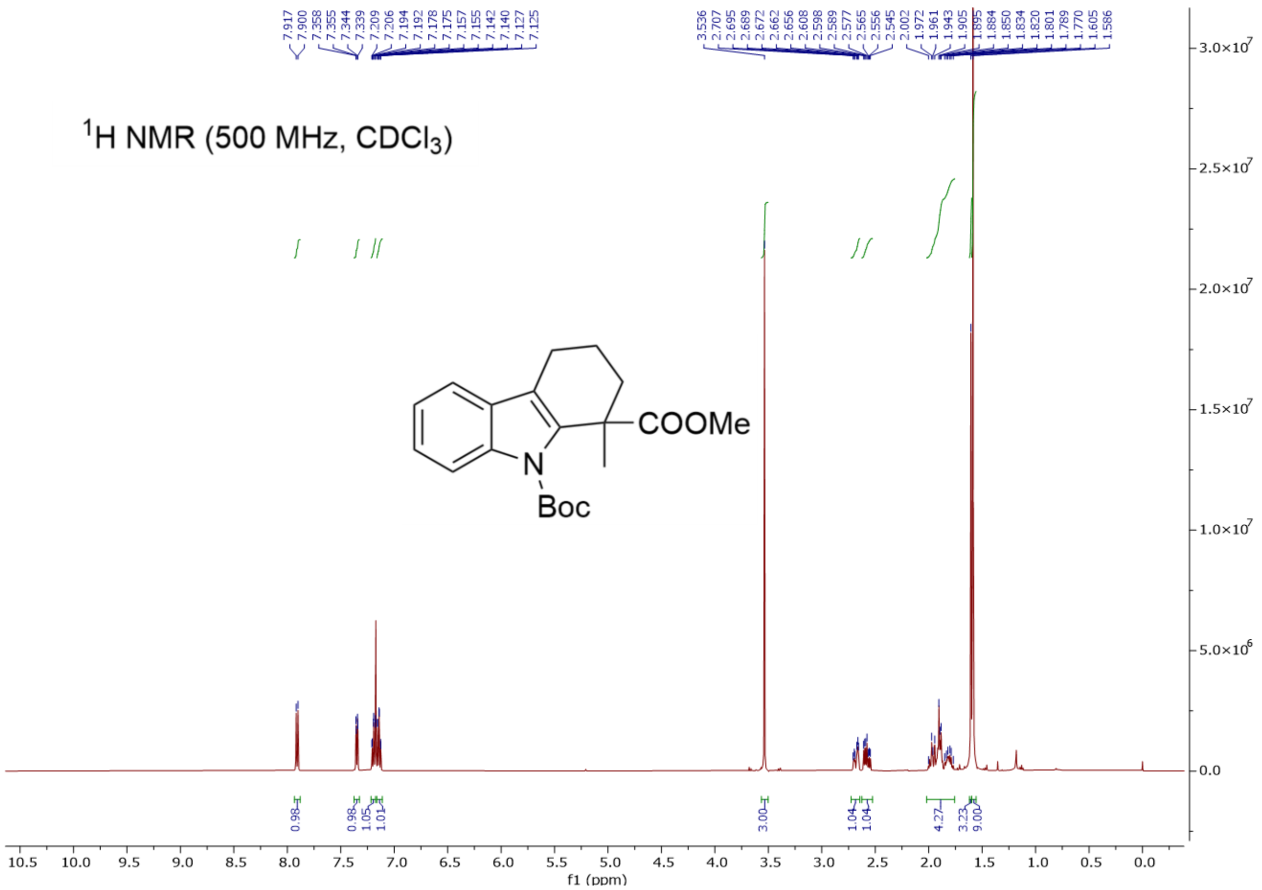

^{1}H NMR (500 MHz, CDCl$_3$)
COOMe
N
Boc
f1 (ppm)

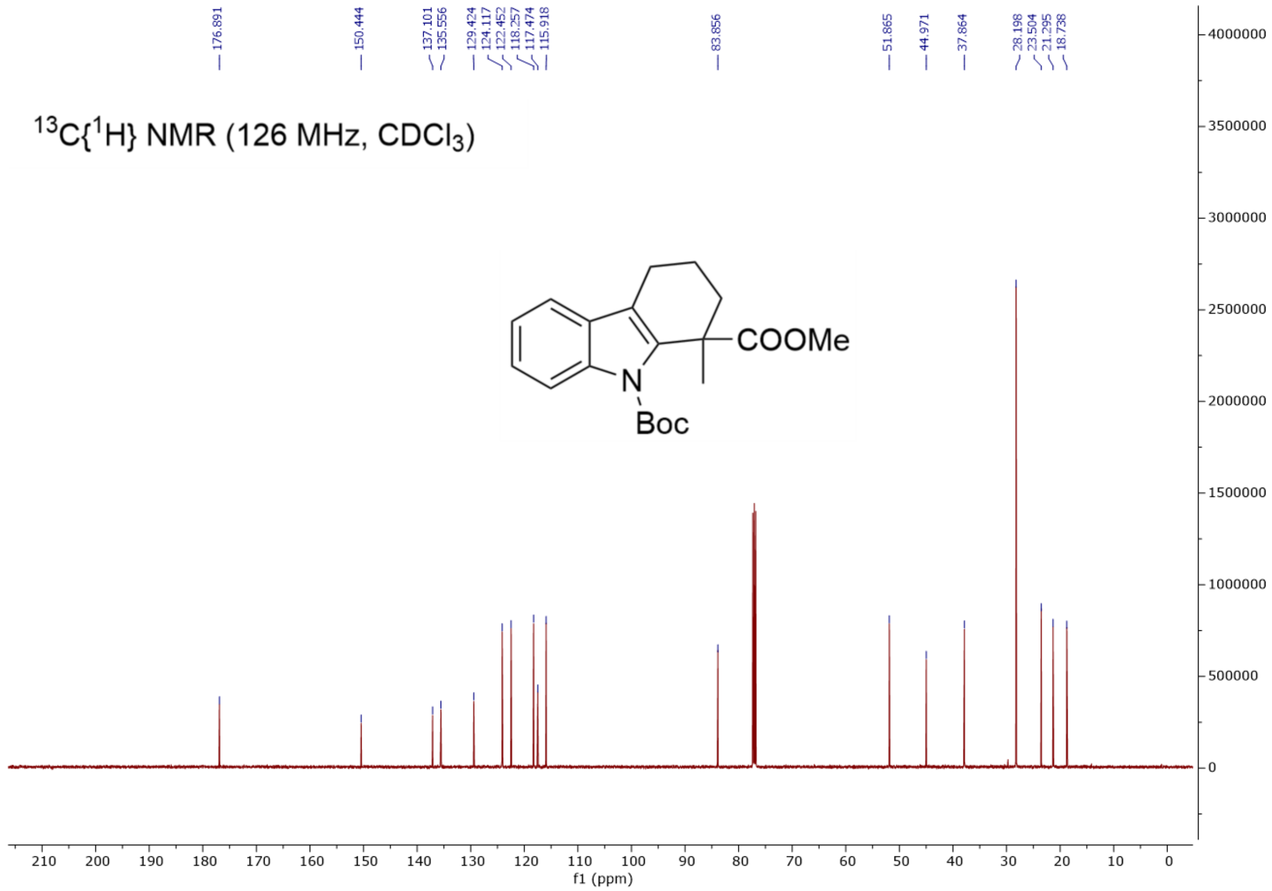

^{13}C{^{1}H} NMR (126 MHz, CDCl$_3$)
176.891
150.444
137.101
135.556
129.424
124.117
122.452
118.257
117.474
115.918
83.856
51.865
44.971
37.864
28.198
23.504
21.295
18.738
COOMe
N
Boc
f1 (ppm)

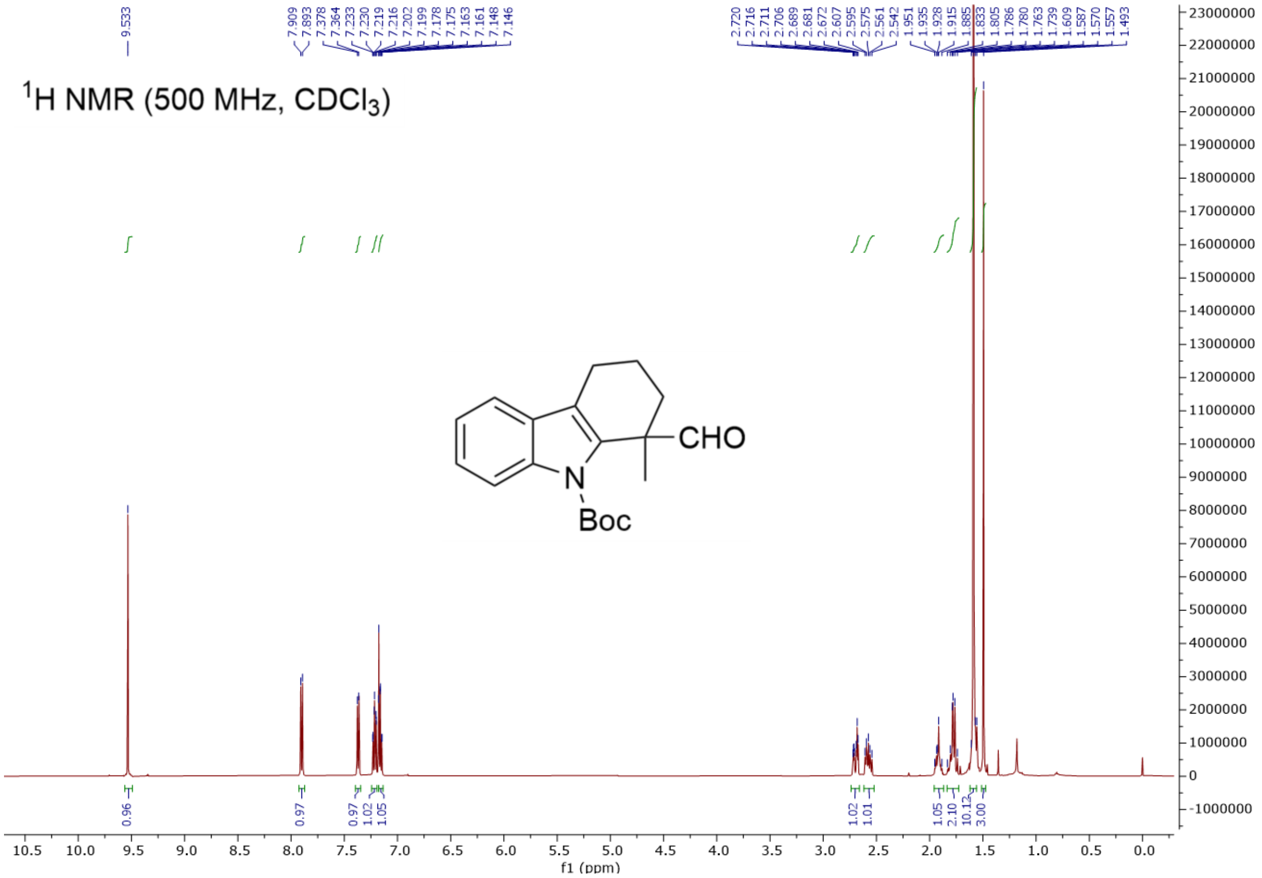

^{1}H NMR (500 MHz, CDCl$_3$)
CHO
N
Boc
f1 (ppm)

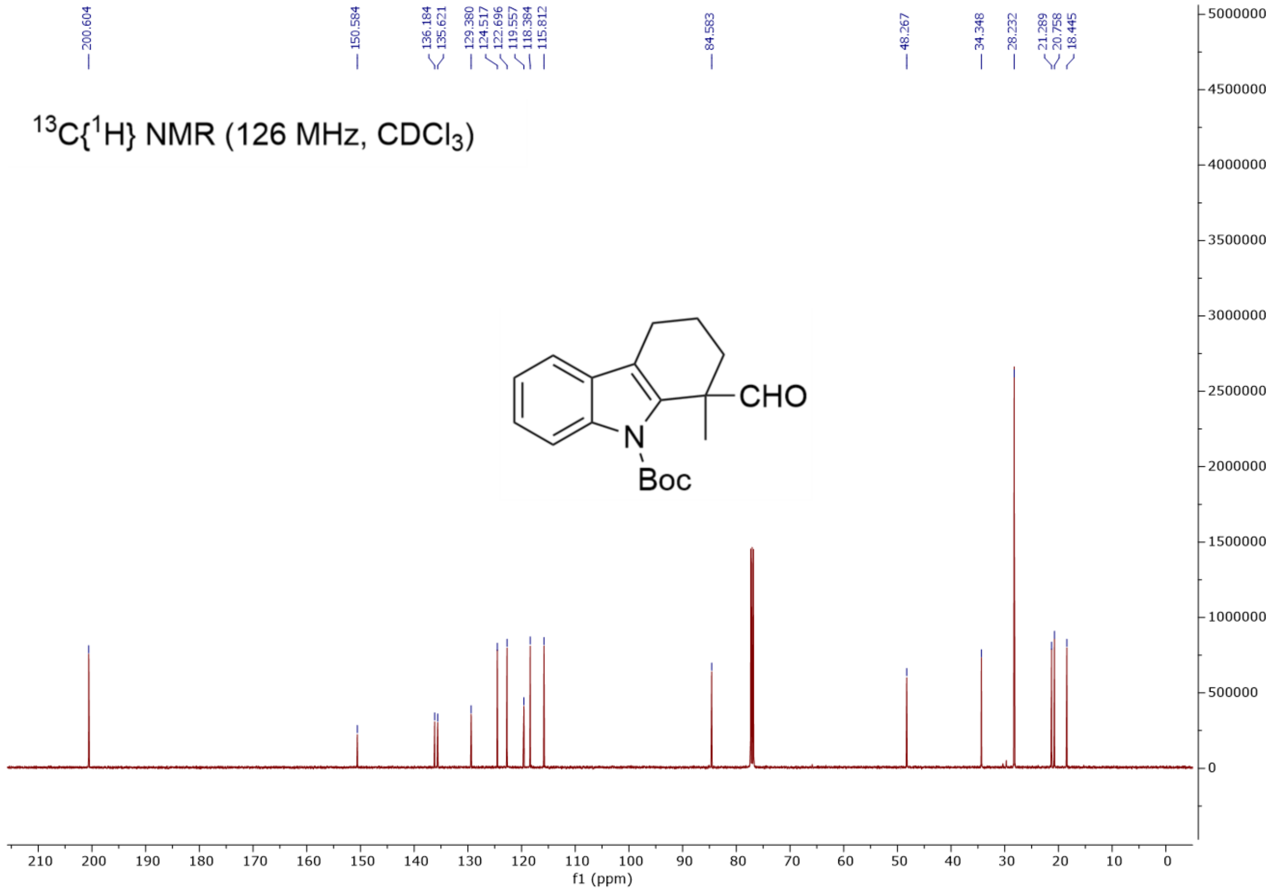

^{13}C{^{1}H} NMR (126 MHz, CDCl$_3$)
CHO
N
Boc
f1 (ppm)

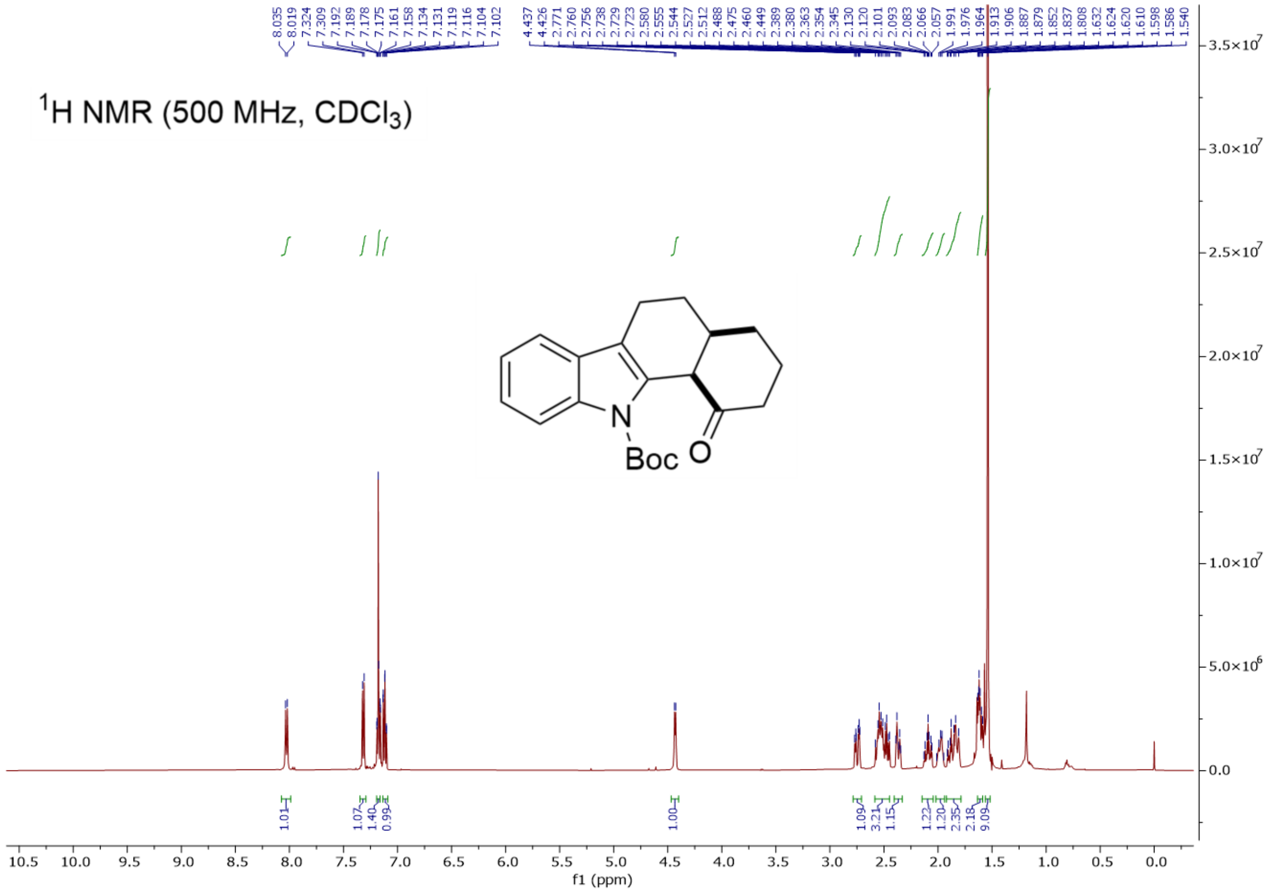

1H NMR (500 MHz, CDCl3)
Boc
O
N
f1 (ppm)

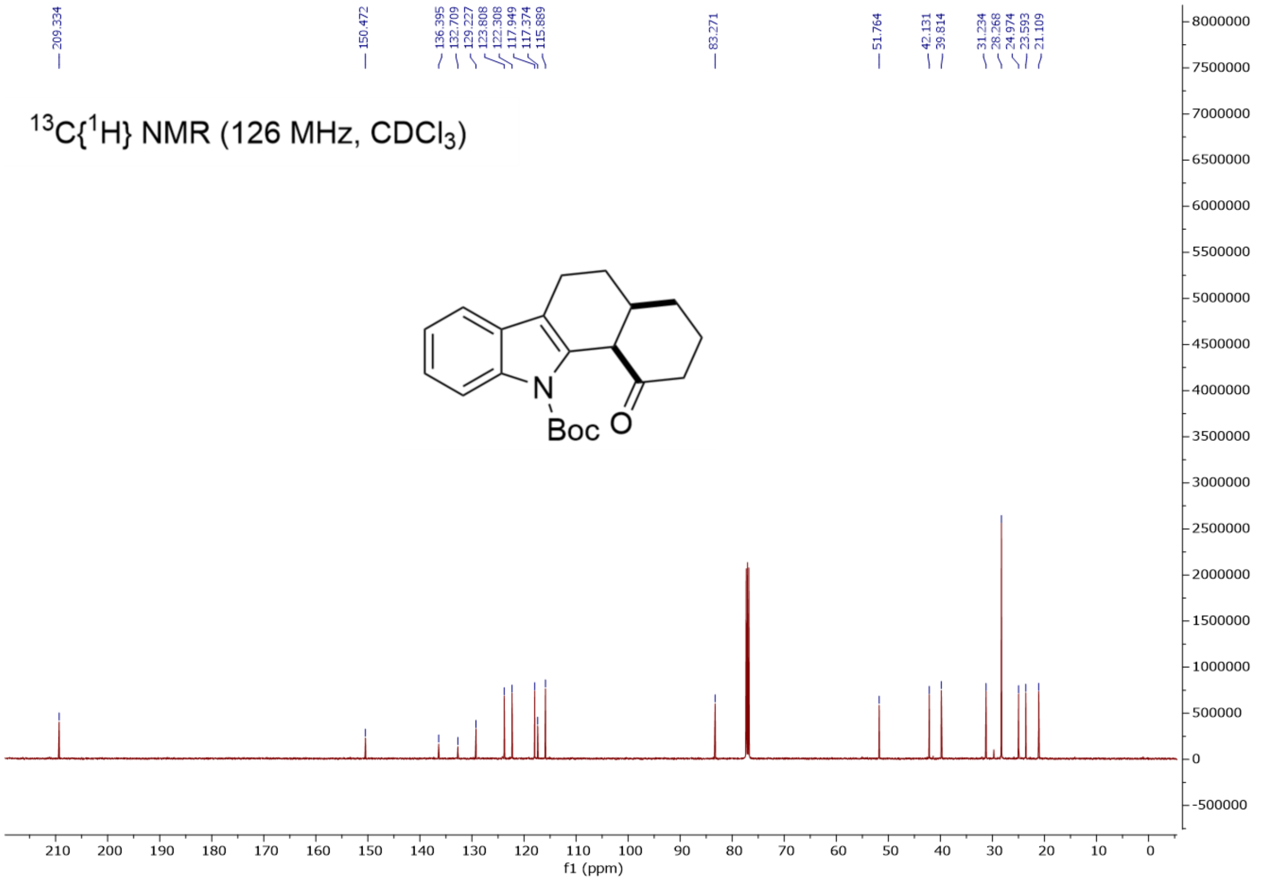

13C{1H} NMR (126 MHz, CDCl3)
Boc
O
N
f1 (ppm)

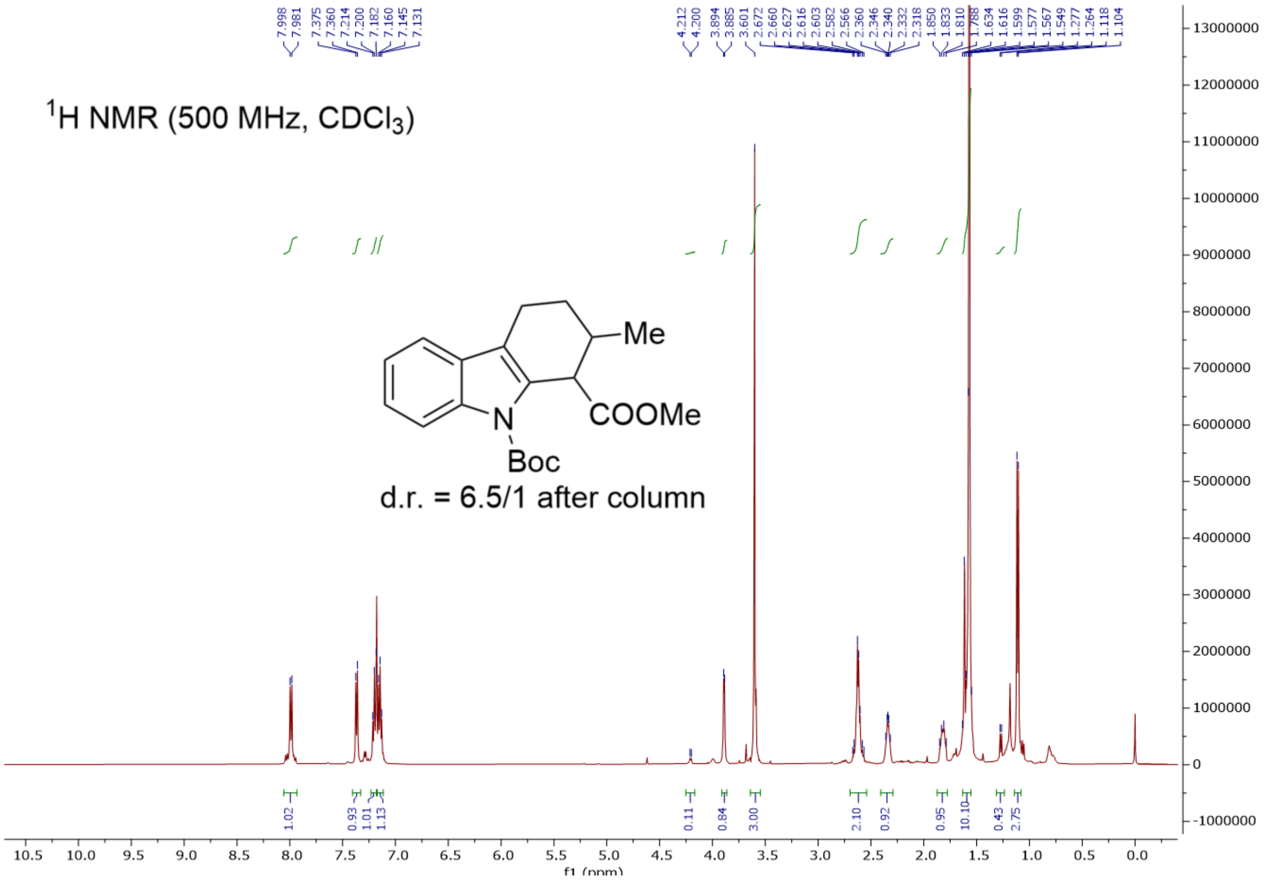

1H NMR (500 MHz, CDCl3)
Me
N
COOMe
Boc
d.r. = 6.5/1 after column
f1 (ppm)

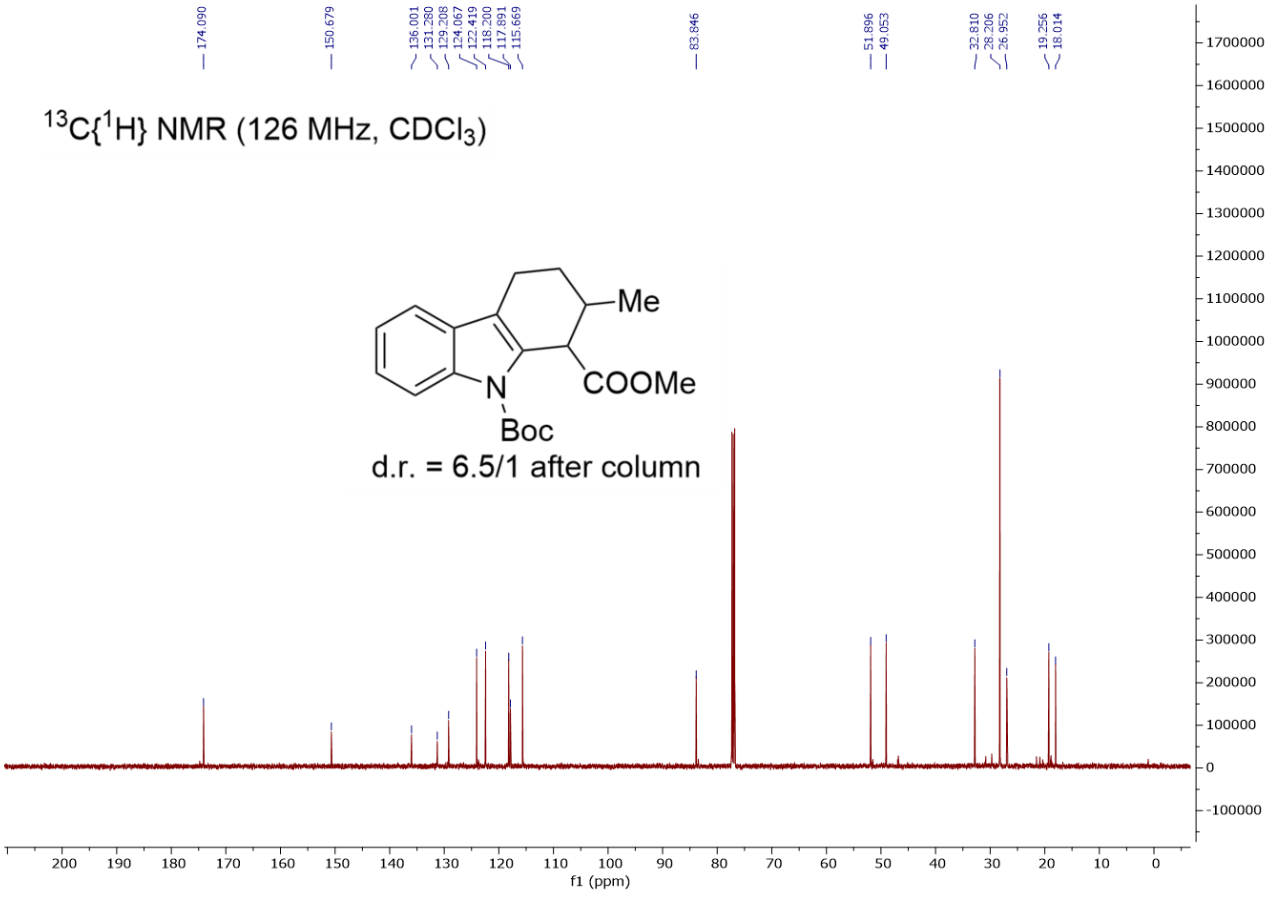

13C{1H} NMR (126 MHz, CDCl3)
Me
N
COOMe
Boc
d.r. = 6.5/1 after column
f1 (ppm)

Initial ¹H NMR to determine the d.r. value

¹H NMR (500 MHz, $CDCl_3$)

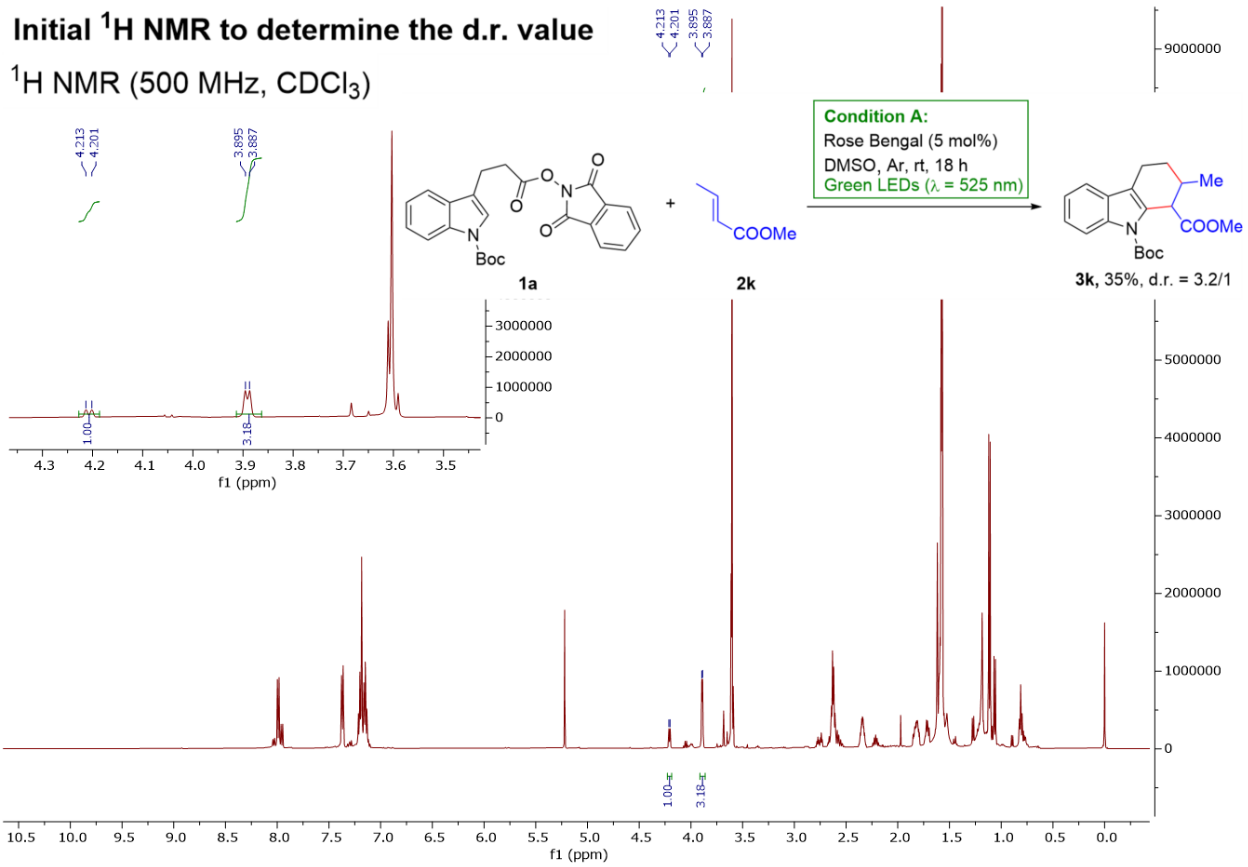

Initial ¹H NMR to determine the d.r. value

¹H NMR (500 MHz, $CDCl_3$)

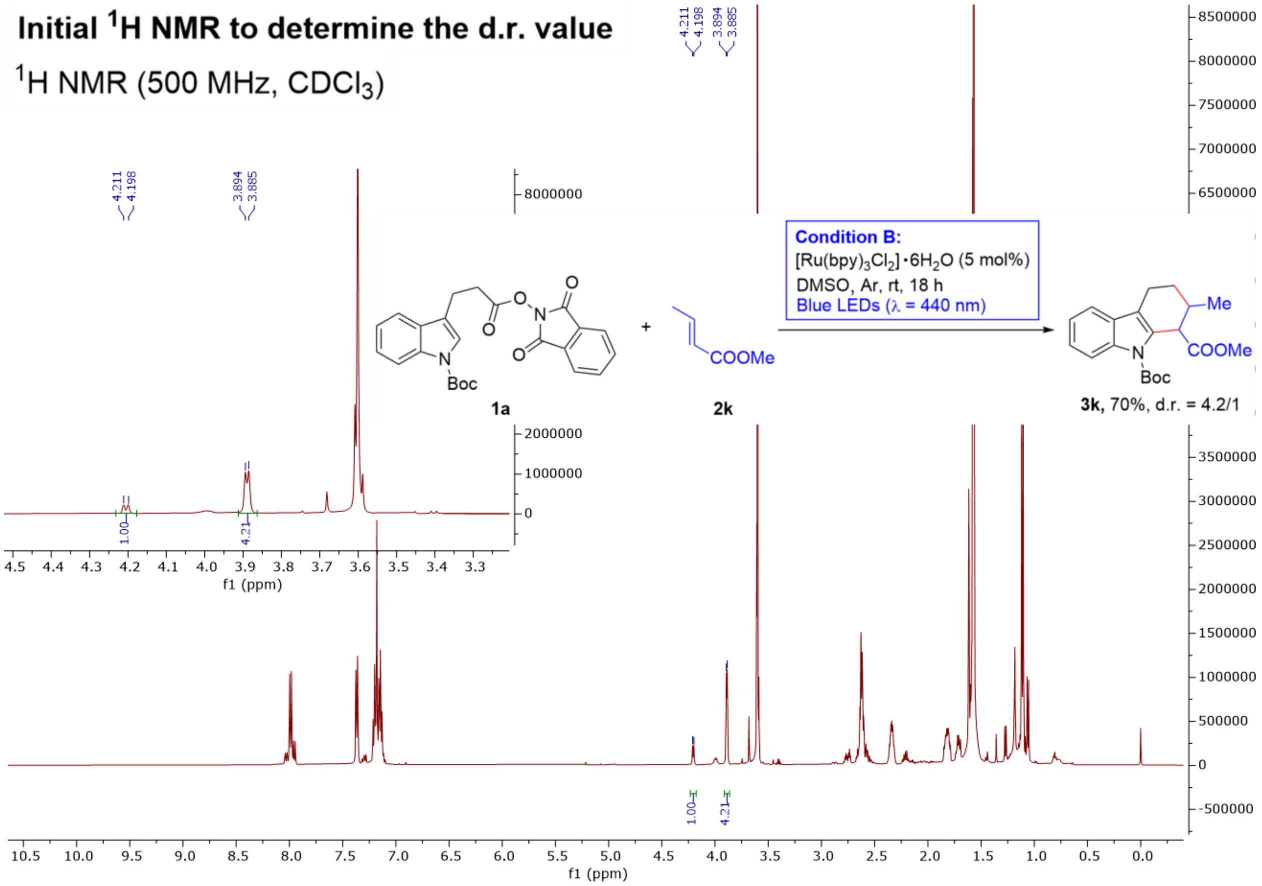

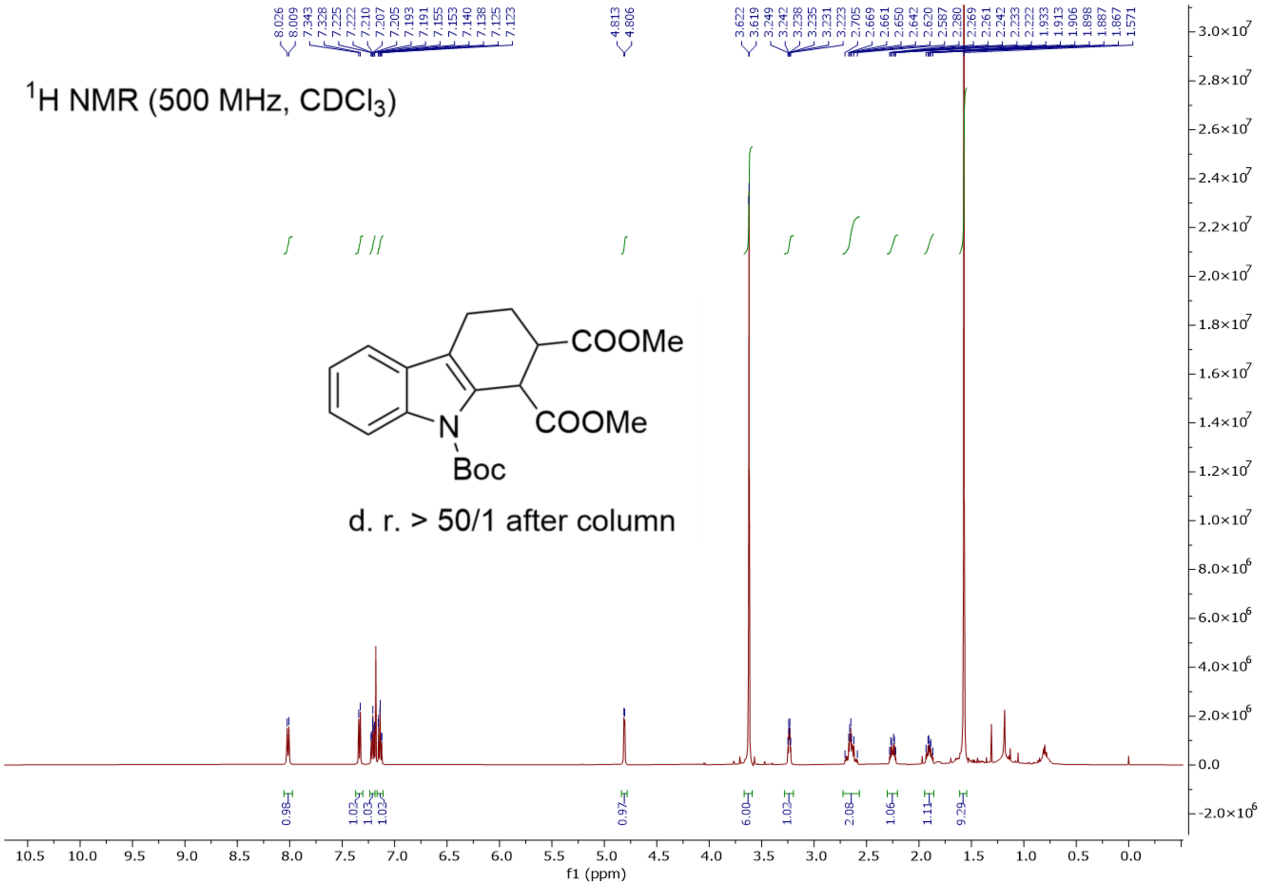

^{1}H NMR (500 MHz, $CDCl_3$)
COOMe
COOMe
N
Boc
d. r. > 50/1 after column
8.026 8.009 7.343 7.328 7.225 7.222 7.210 7.207 7.205 7.193 7.191 7.155 7.153 7.140 7.138 7.125 7.123
4.813 4.806
3.622 3.619 3.249 3.242 3.238 3.235 3.231 3.223 2.705 2.669 2.661 2.650 2.642 2.620 2.587 2.280 2.269 2.261 2.242 2.233 2.222 1.933 1.913 1.906 1.898 1.887 1.867 1.571
0.98 1.02 1.03 1.02 0.97 6.00 1.02 2.08 1.06 1.11 9.29
f1 (ppm)

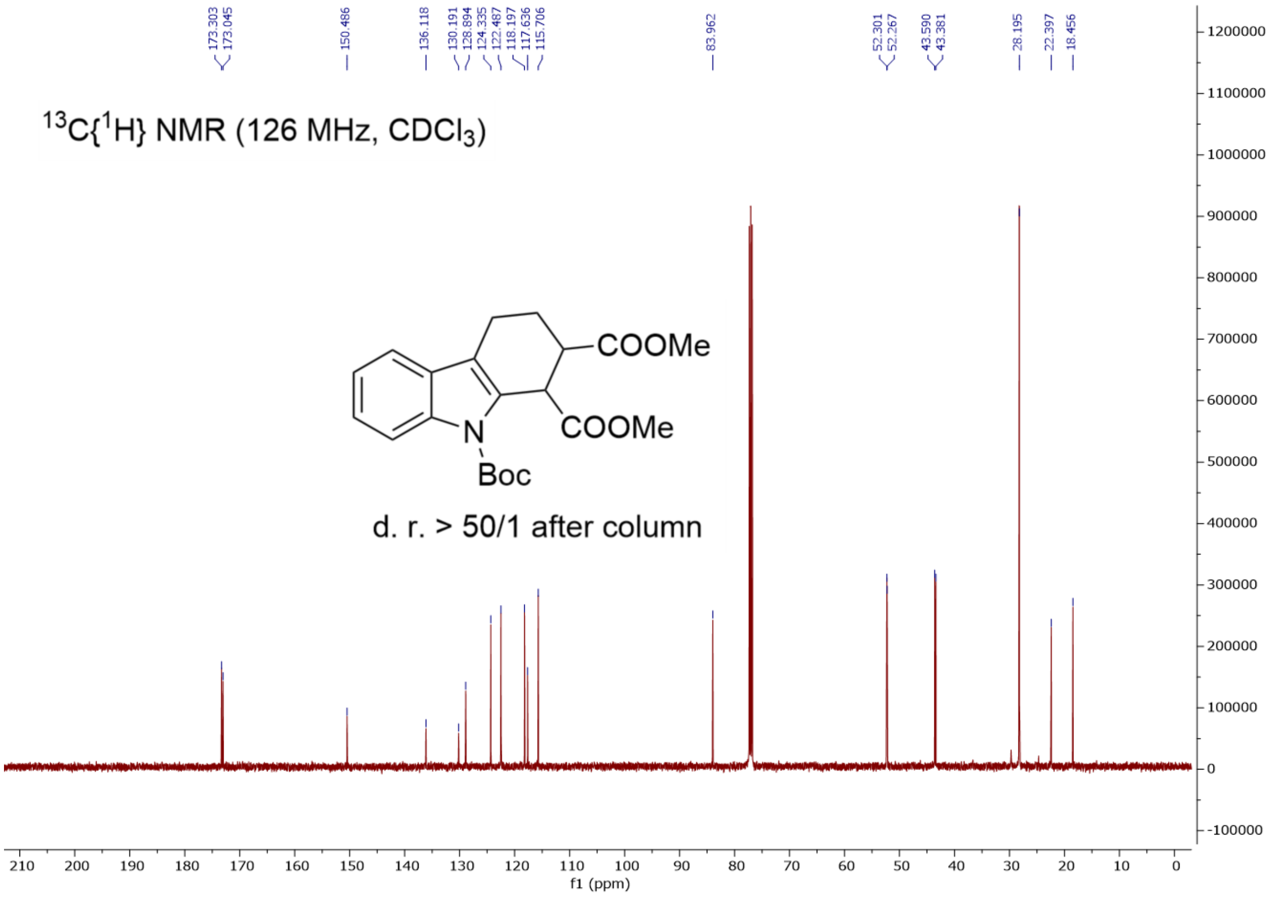

$^{13}C\{^1H\}$ NMR (126 MHz, $CDCl_3$)
COOMe
COOMe
N
Boc
d. r. > 50/1 after column
173.303 173.045 150.486 136.118 130.191 128.894 124.335 122.487 118.197 117.636 115.706 83.962 52.301 52.267 43.590 43.381 28.195 22.397 18.456
f1 (ppm)

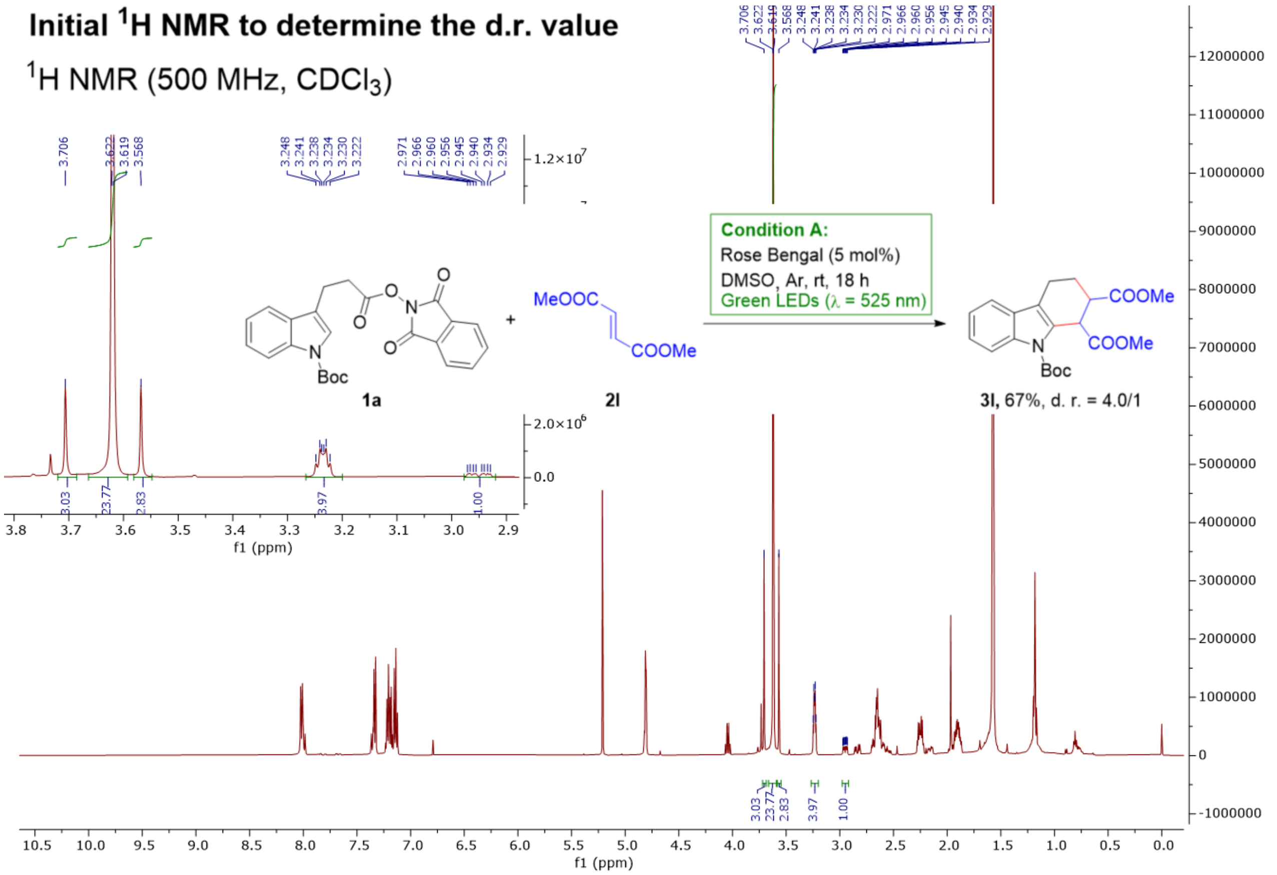
Initial 1H NMR to determine the d.r. value
1H NMR (500 MHz, CDCl3)
Condition A:
Rose Bengal (5 mol%)
DMSO, Ar, rt, 18 h
Green LEDs (λ = 525 nm)
1a
2l
3l, 67%, d. r. = 4.0/1
f1 (ppm)

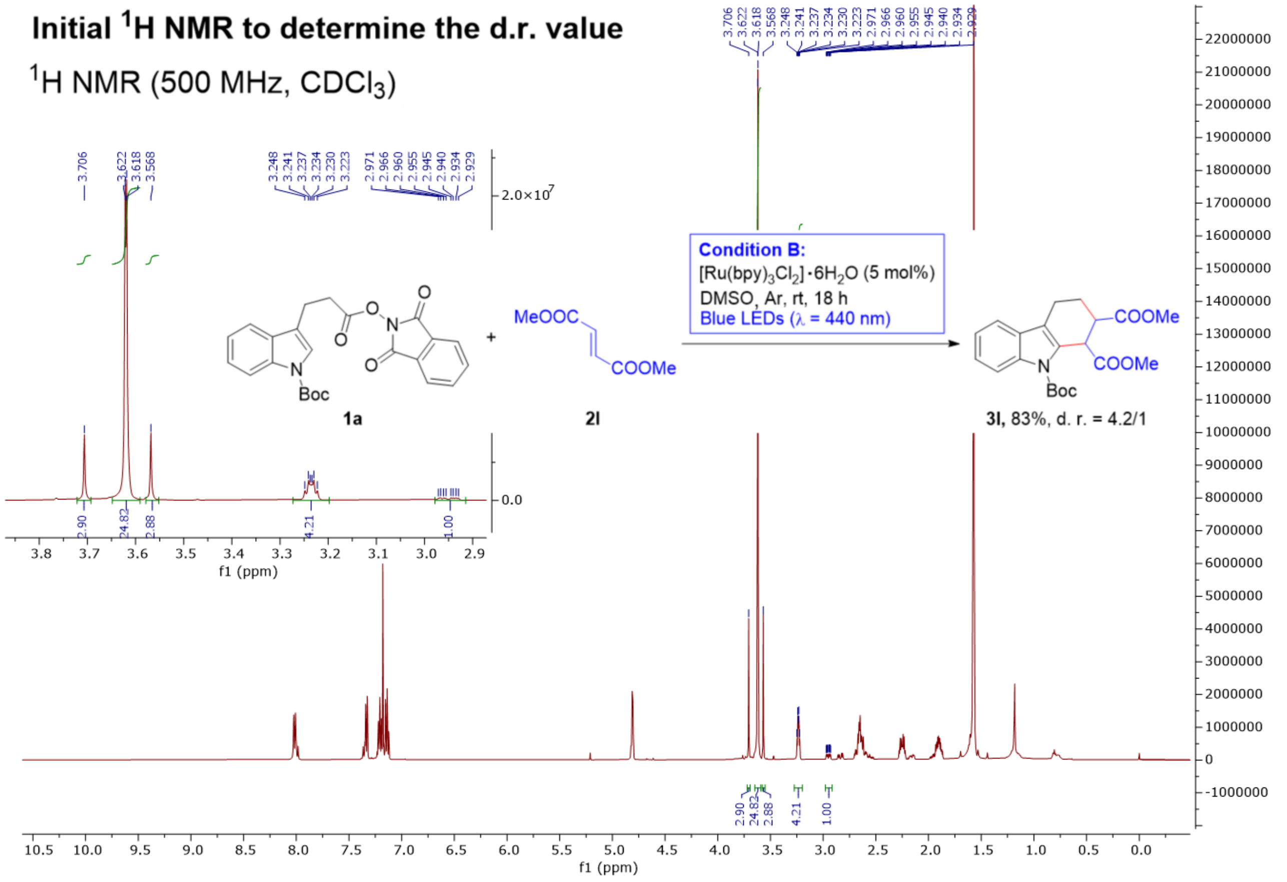
Initial 1H NMR to determine the d.r. value
1H NMR (500 MHz, CDCl3)
Condition B:
[Ru(bpy)3Cl2]·6H2O (5 mol%)
DMSO, Ar, rt, 18 h
Blue LEDs (λ = 440 nm)
1a
2l
3l, 83%, d. r. = 4.2/1
f1 (ppm)

Initial ^{1}H NMR to determine the d.r. value

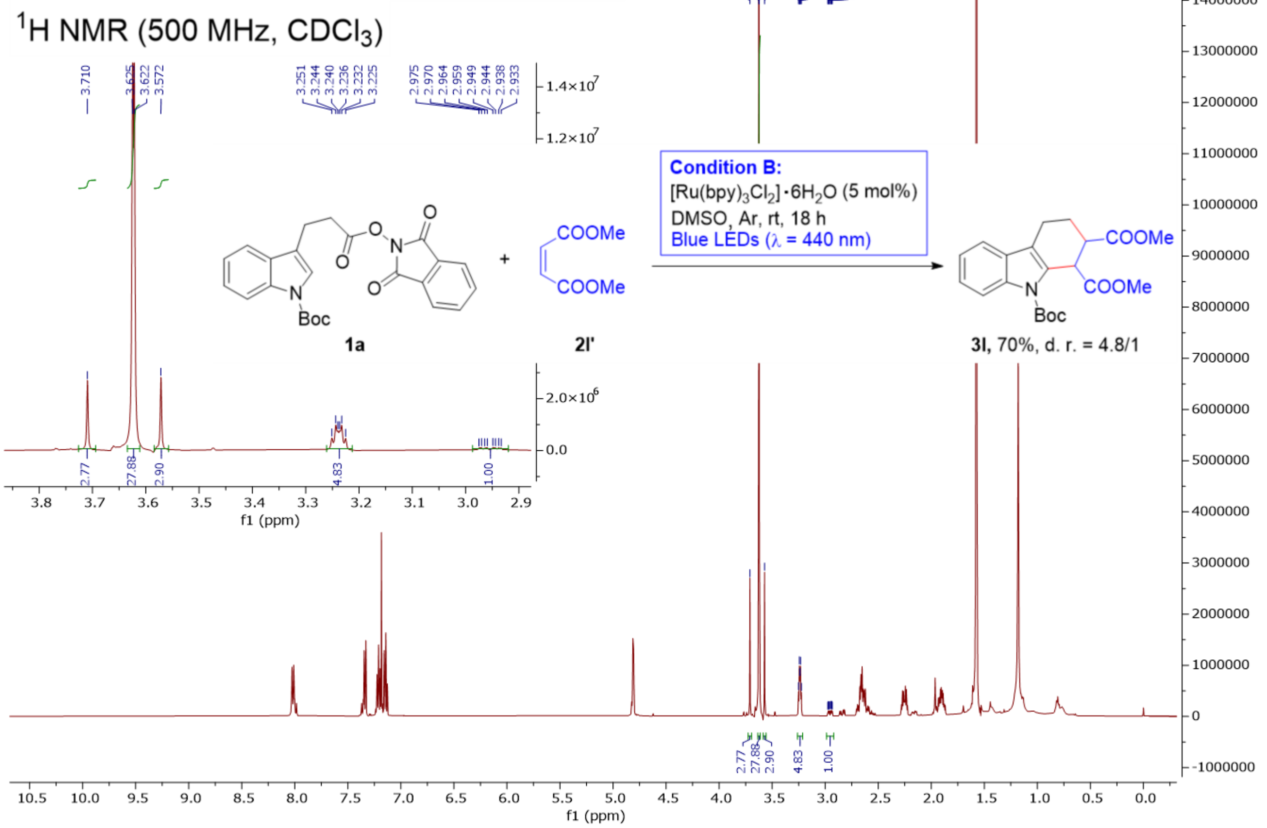

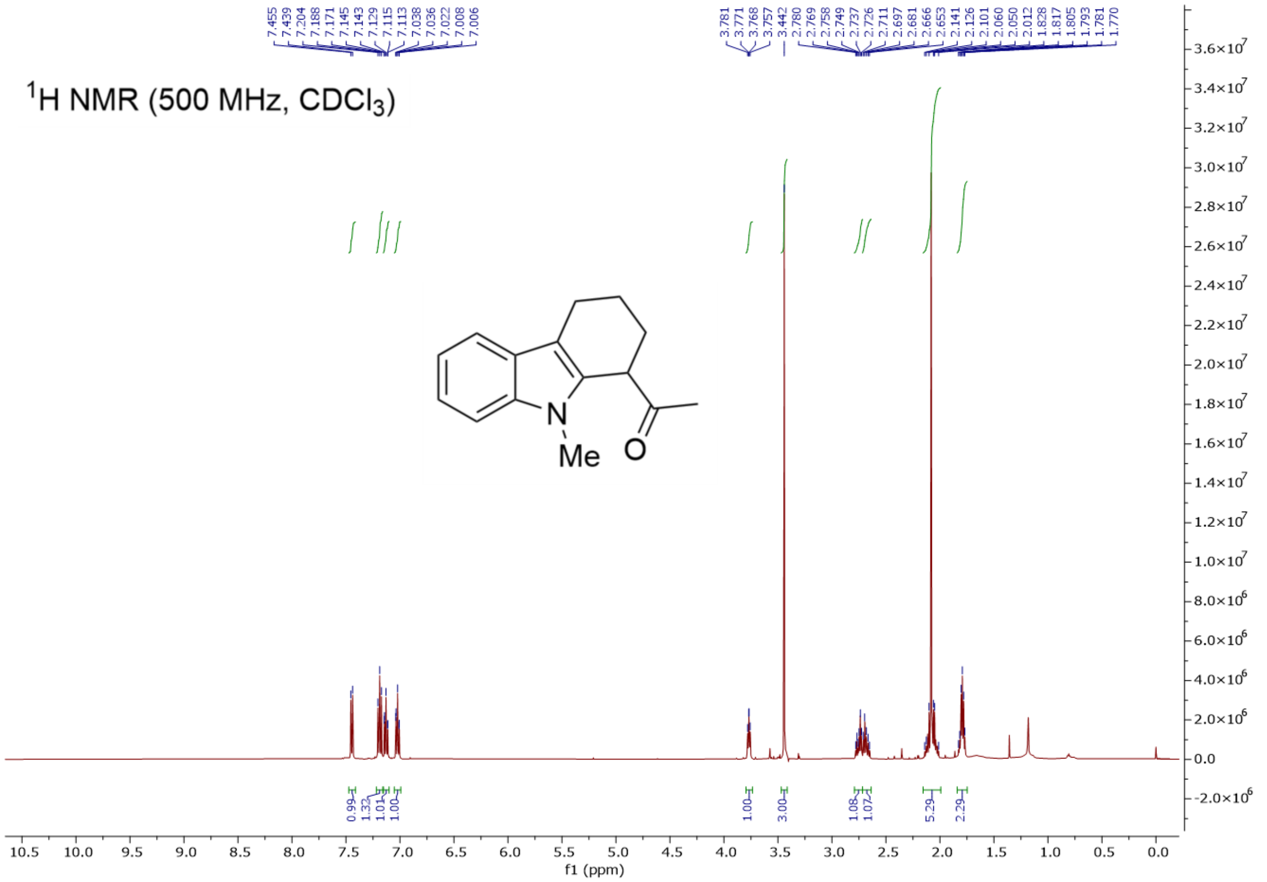

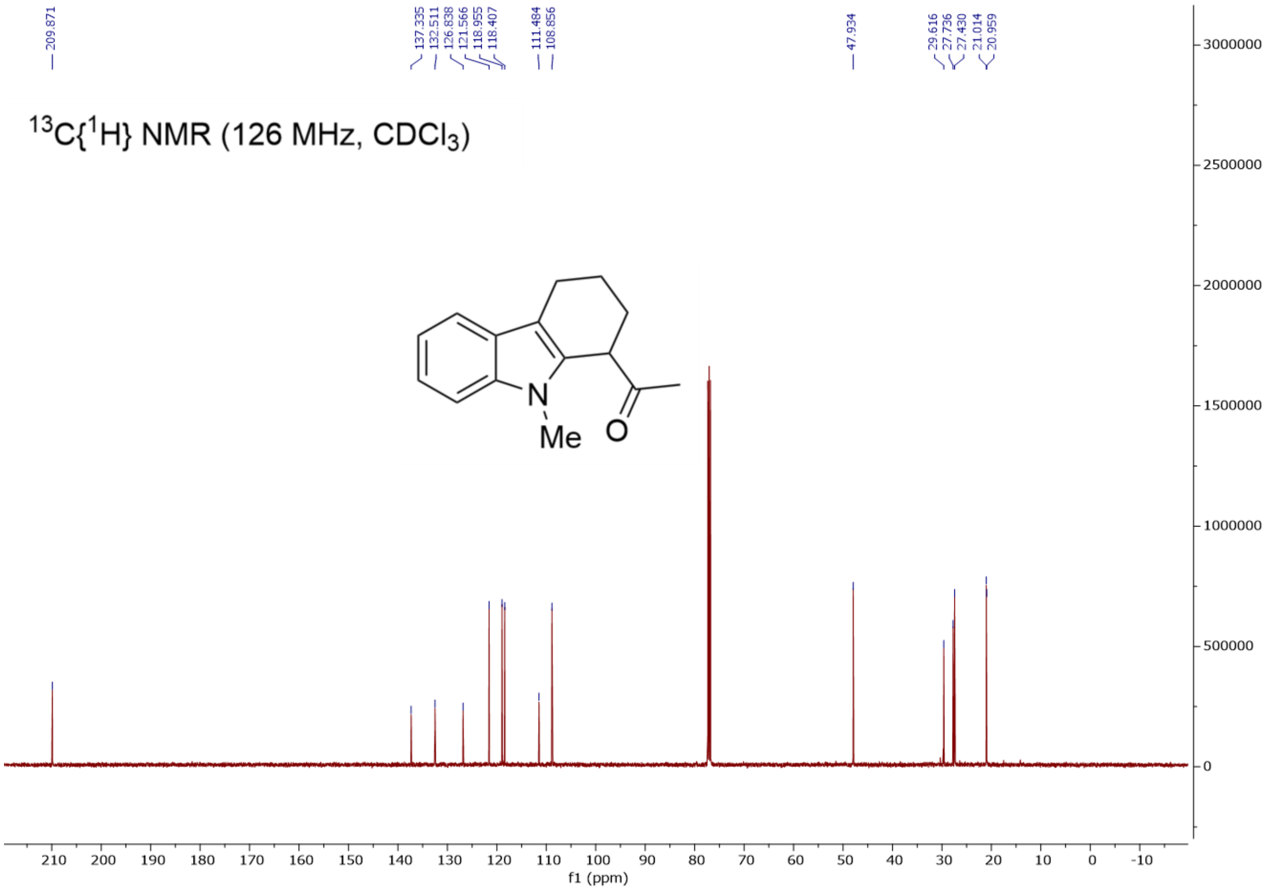

13C{1H} NMR (126 MHz, CDCl3)
209.871
137.335
132.511
126.838
121.566
118.955
118.407
111.484
108.856
47.934
29.616
27.736
27.430
21.014
20.959
N
Me
O
f1 (ppm)

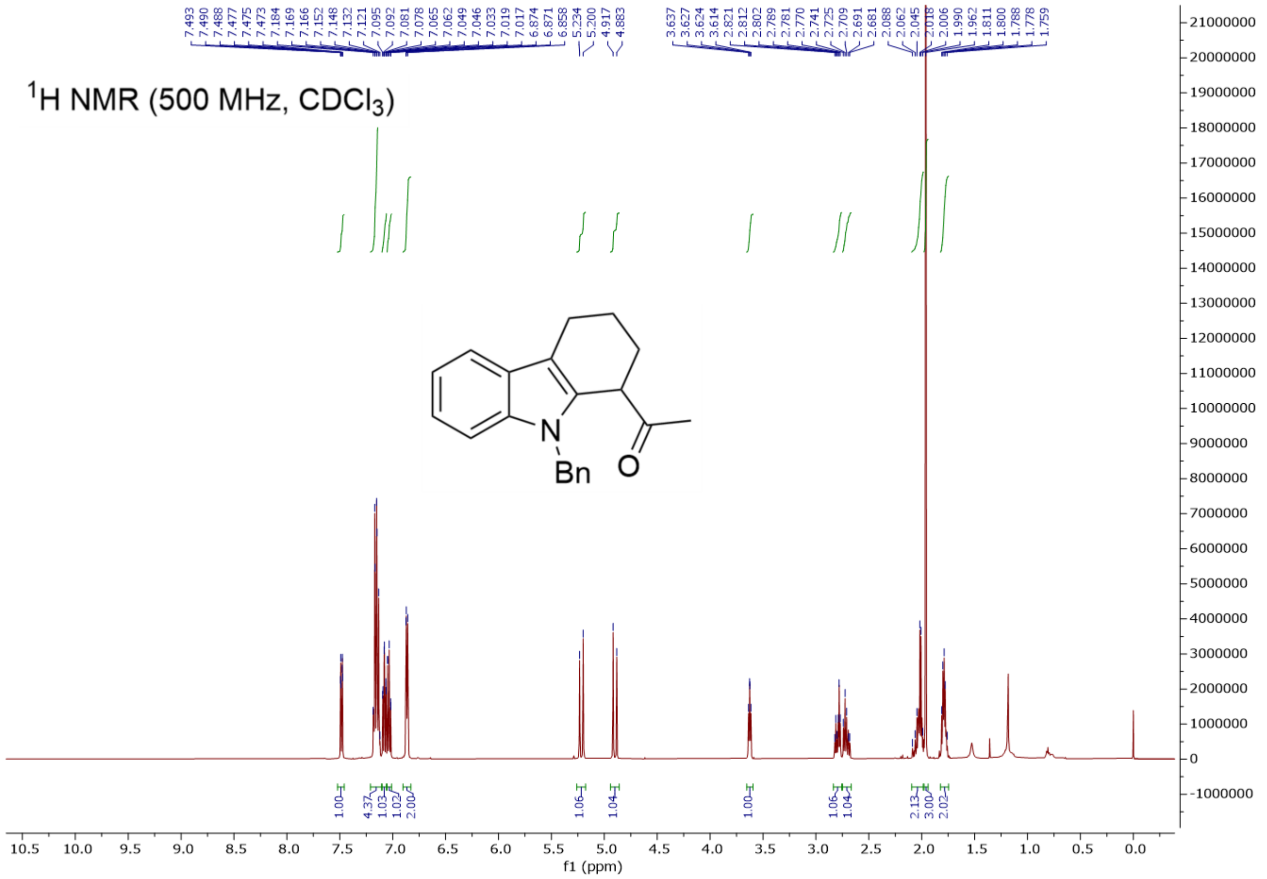

1H NMR (500 MHz, CDCl3)
N
Bn
O
1.00
4.37
1.03
1.02
2.00
1.06
1.04
1.00
1.06
1.04
2.13
3.00
2.02
f1 (ppm)

$^{13}C\{^{1}H\}$ NMR (126 MHz, $CDCl_3$)

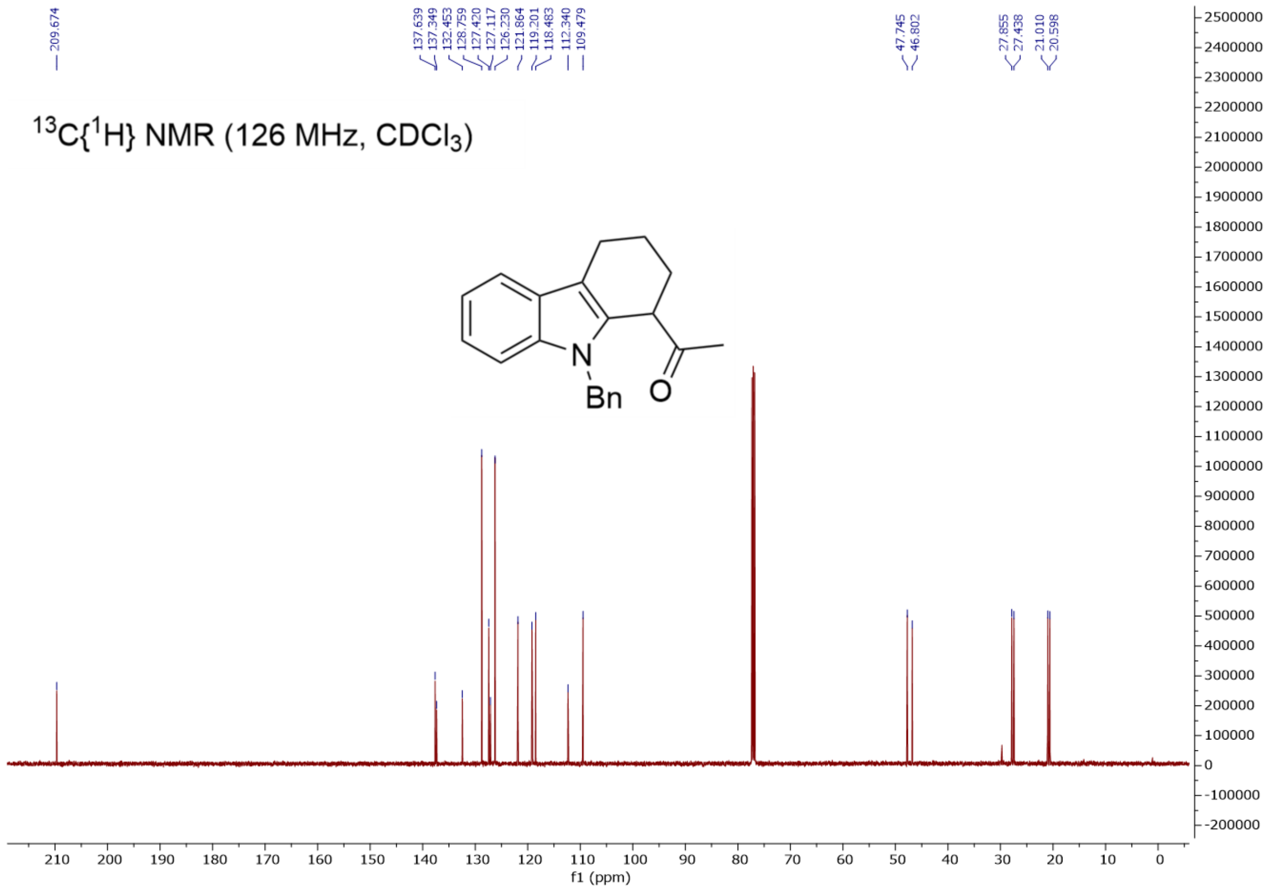

^{1}H NMR (500 MHz, $CDCl_3$)

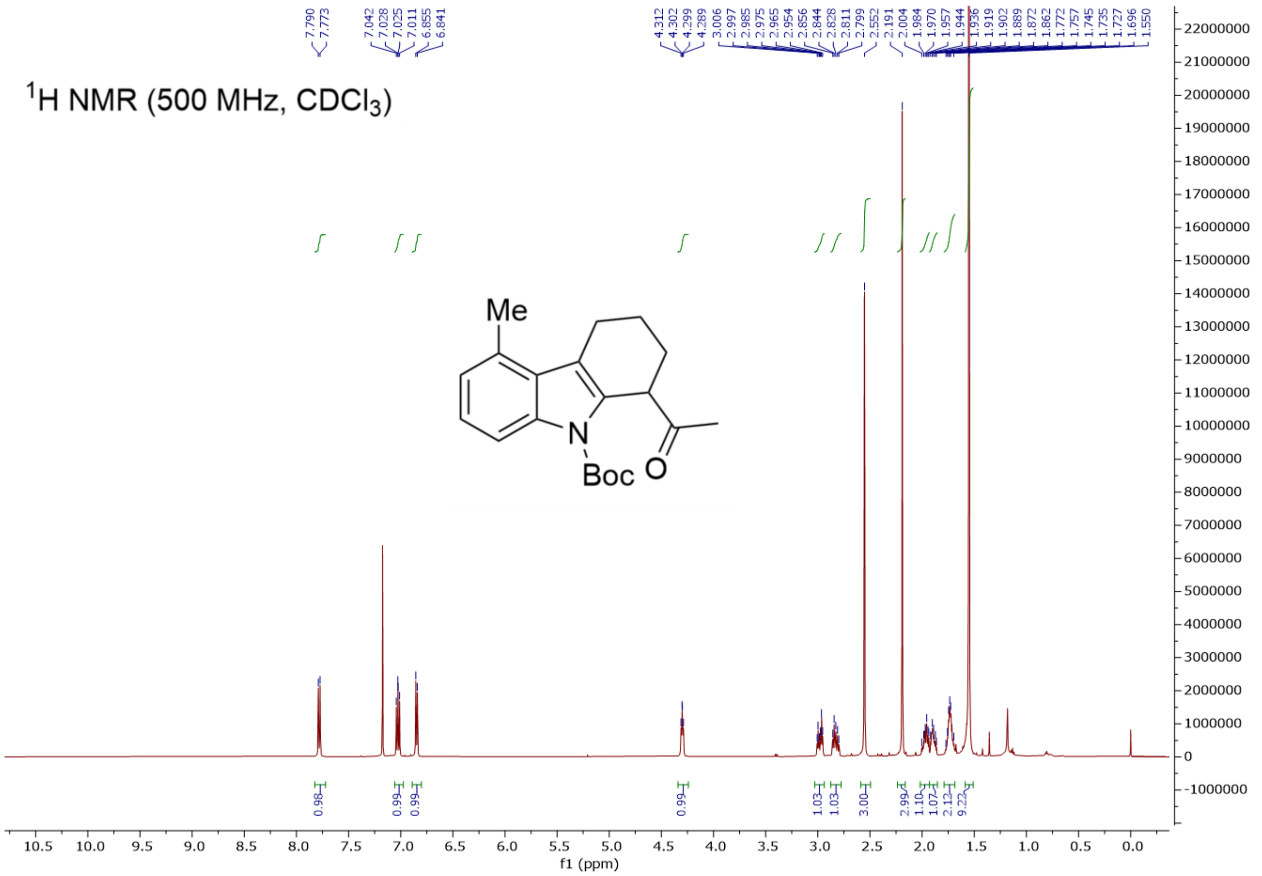

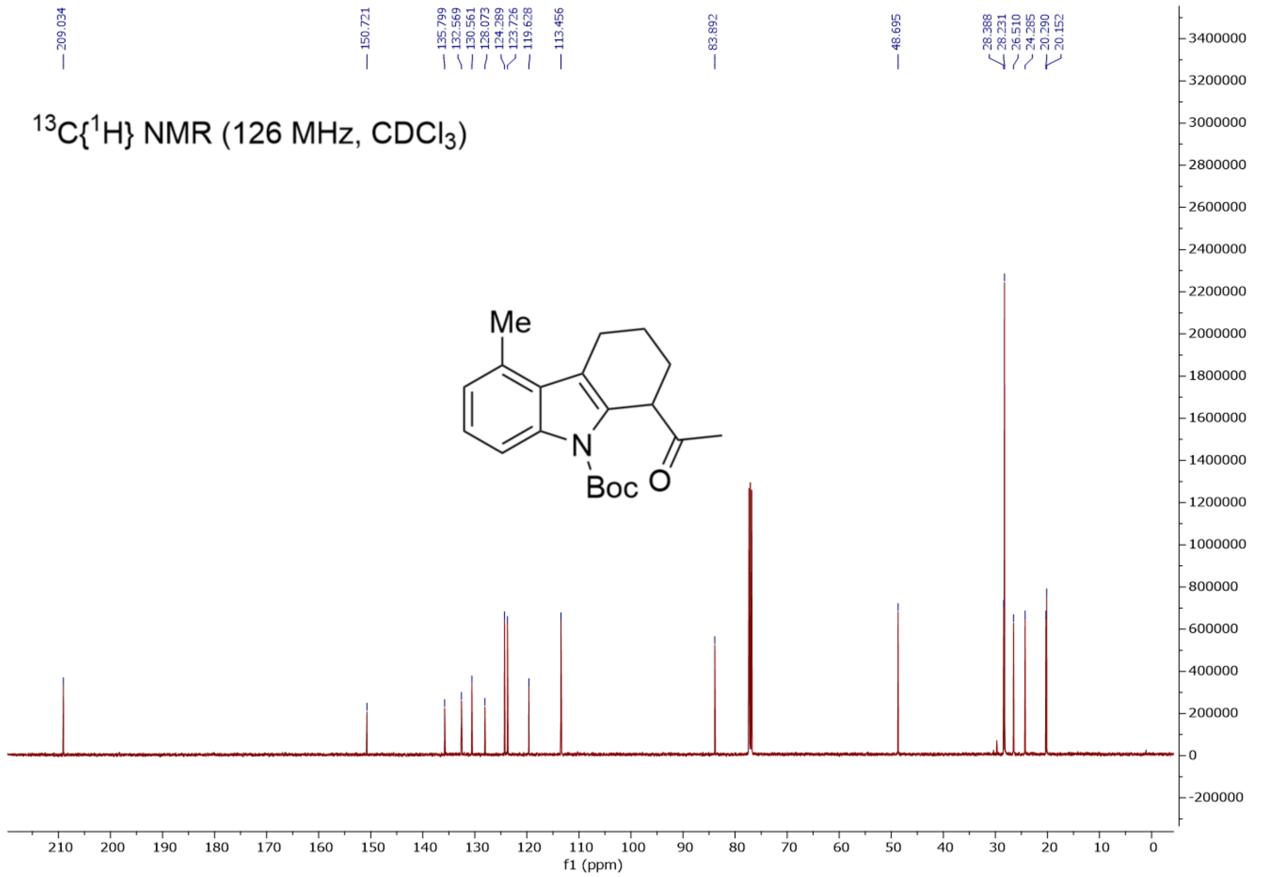

209.034
150.721
135.799
132.569
130.561
128.073
124.289
123.726
119.628
113.456
83.892
48.695
28.388
28.231
26.510
24.285
20.290
20.152
$^{13}C\{^1H\}$ NMR (126 MHz, $CDCl_3$)
Me
N
Boc
O
f1 (ppm)

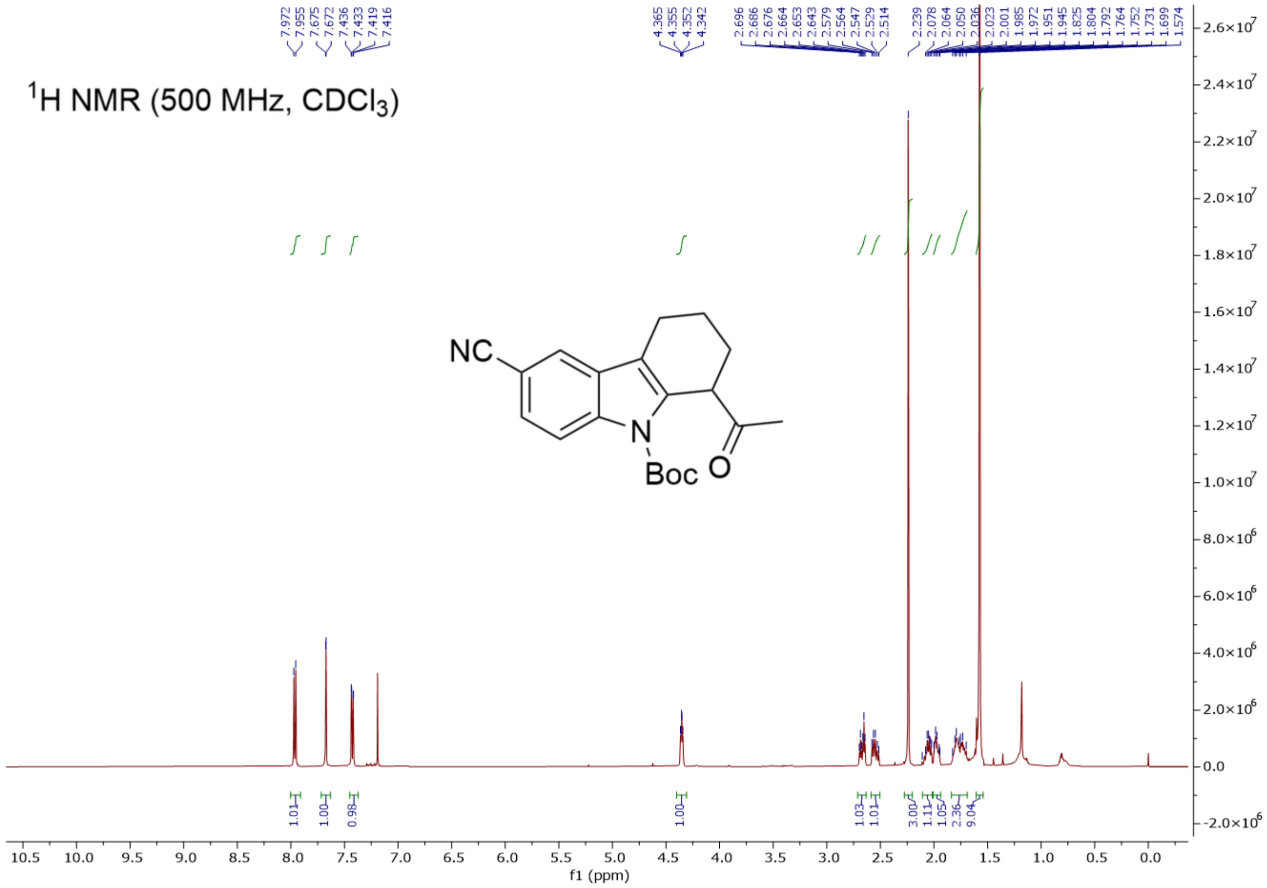

7.972
7.955
7.675
7.672
7.436
7.433
7.419
7.416
4.365
4.355
4.352
4.342
2.696
2.686
2.676
2.664
2.653
2.643
2.579
2.564
2.547
2.529
2.514
2.239
2.078
2.064
2.050
2.036
2.023
2.001
1.985
1.972
1.951
1.945
1.825
1.804
1.792
1.764
1.752
1.731
1.699
1.574
^1H NMR (500 MHz, $CDCl_3$)
NC
N
Boc
O
1.01
1.00
0.98
1.00
1.03
1.01
3.00
1.11
1.05
2.36
9.04
f1 (ppm)

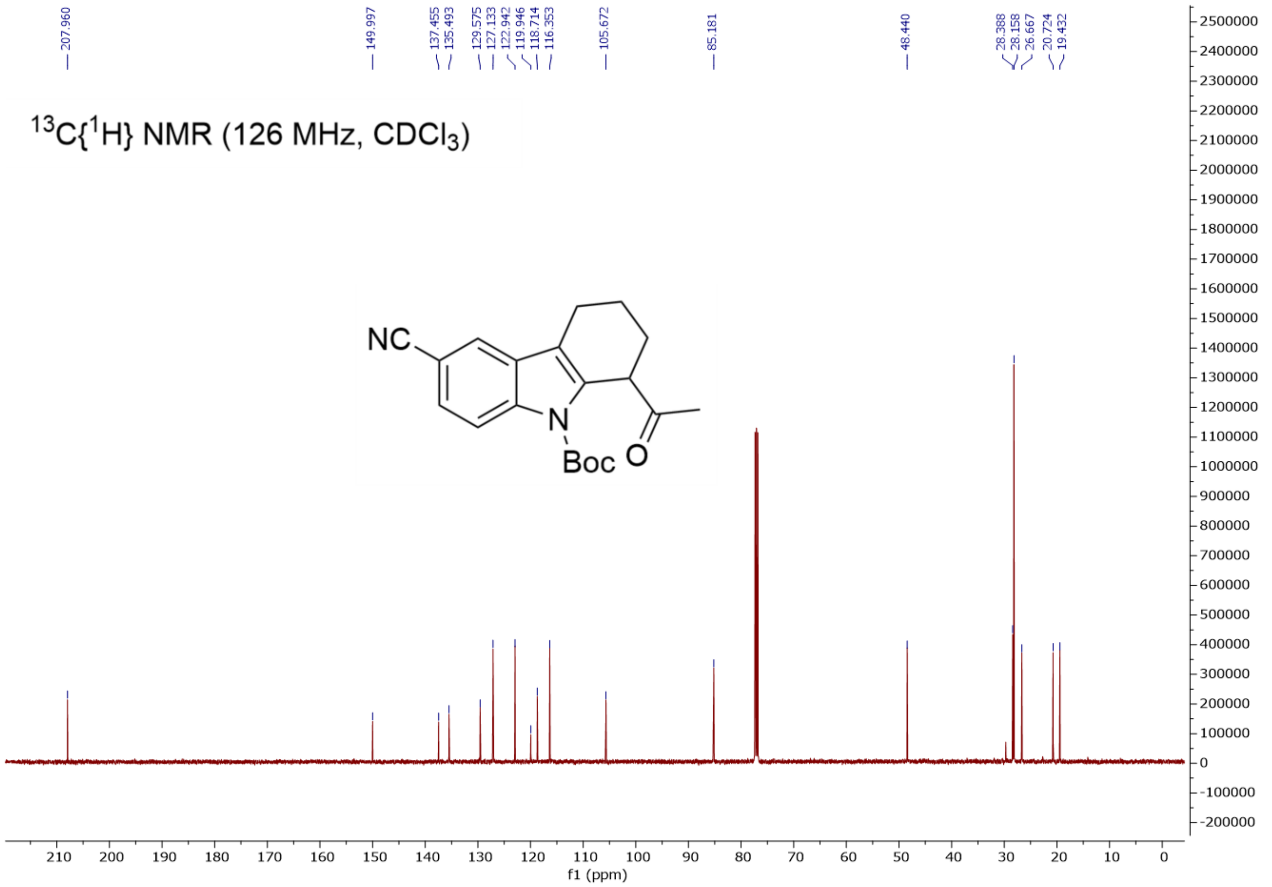

$^{13}C\{^1H\}$ NMR (126 MHz, $CDCl_3$)
NC
N
Boc
O
f1 (ppm)

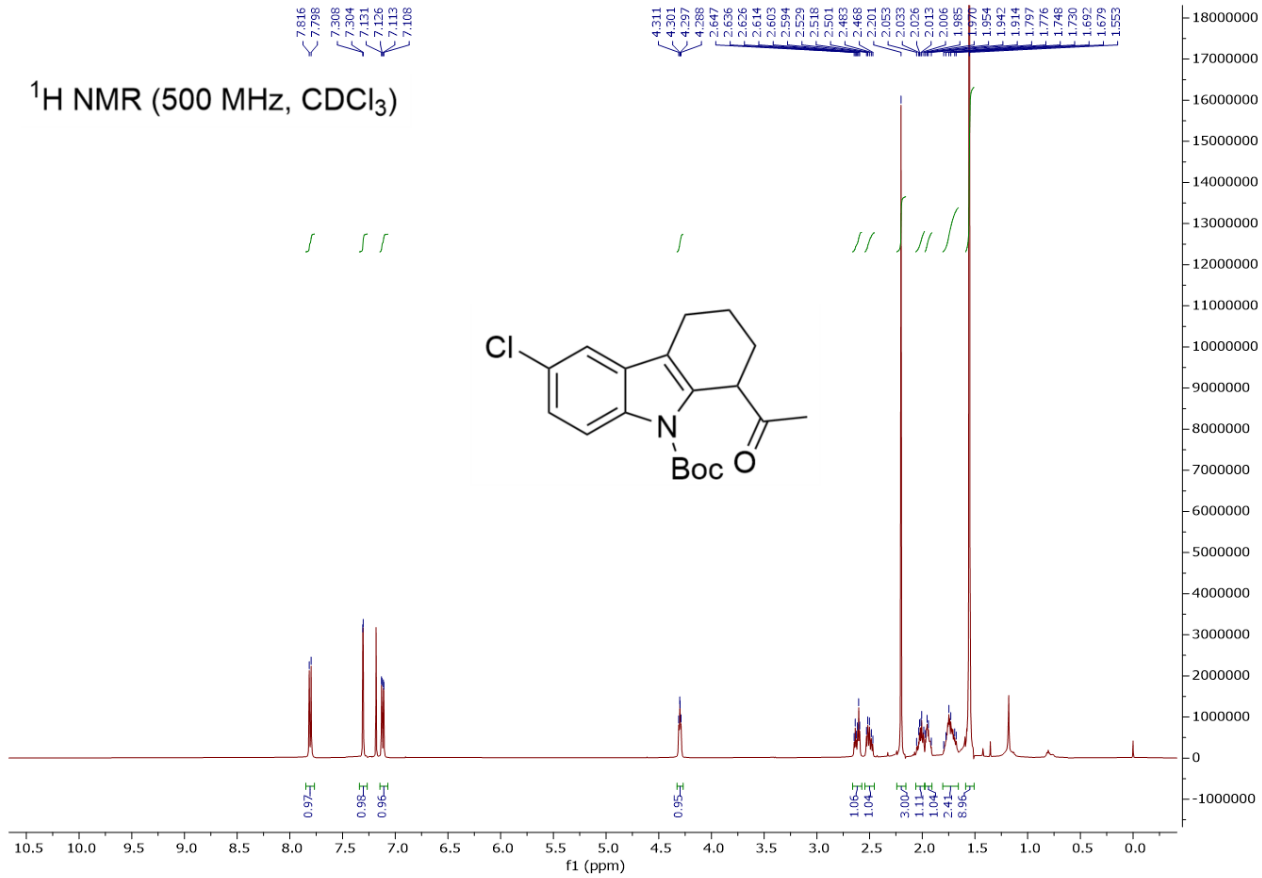

^{1}H NMR (500 MHz, $CDCl_3$)
Cl
N
Boc
O
f1 (ppm)

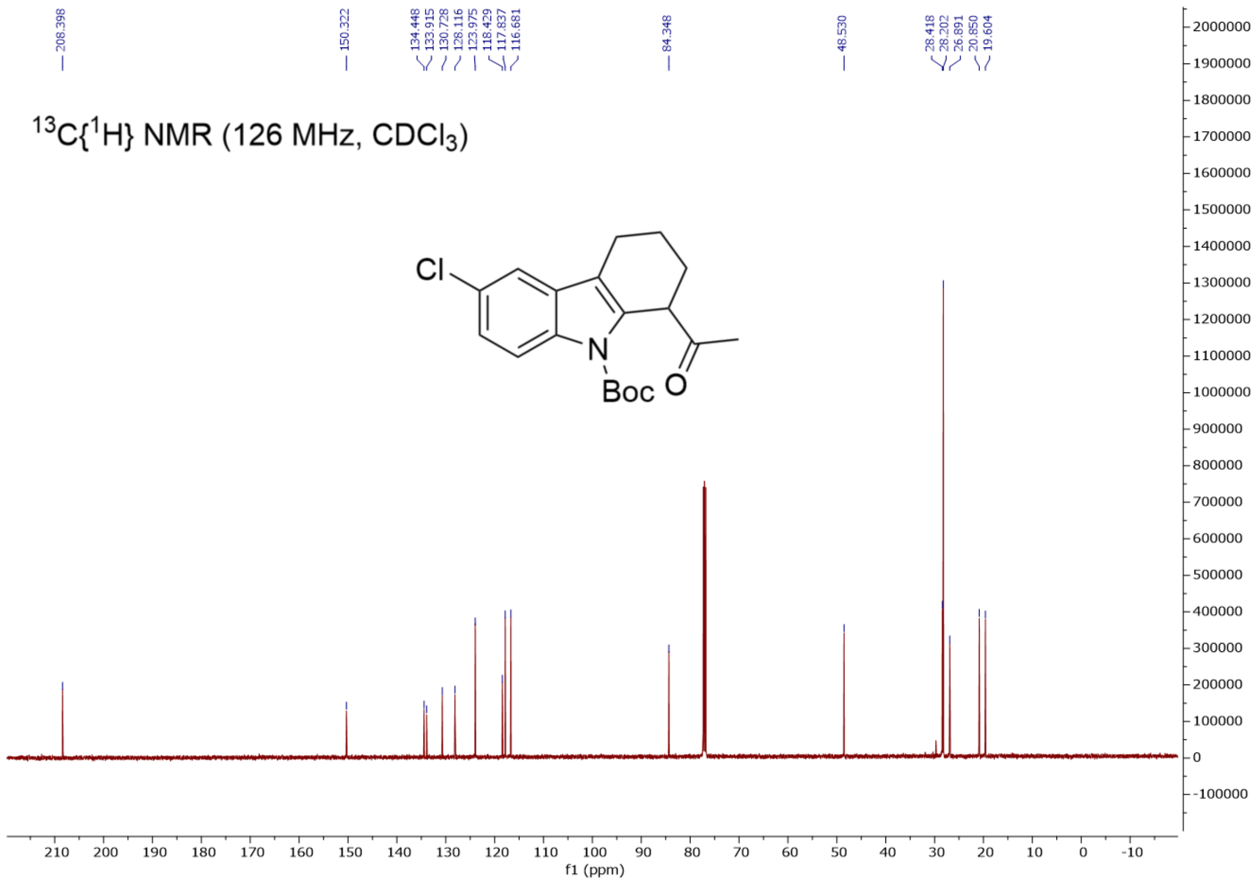
13C{1H} NMR (126 MHz, CDCl3)
Cl
N
Boc
O
f1 (ppm)

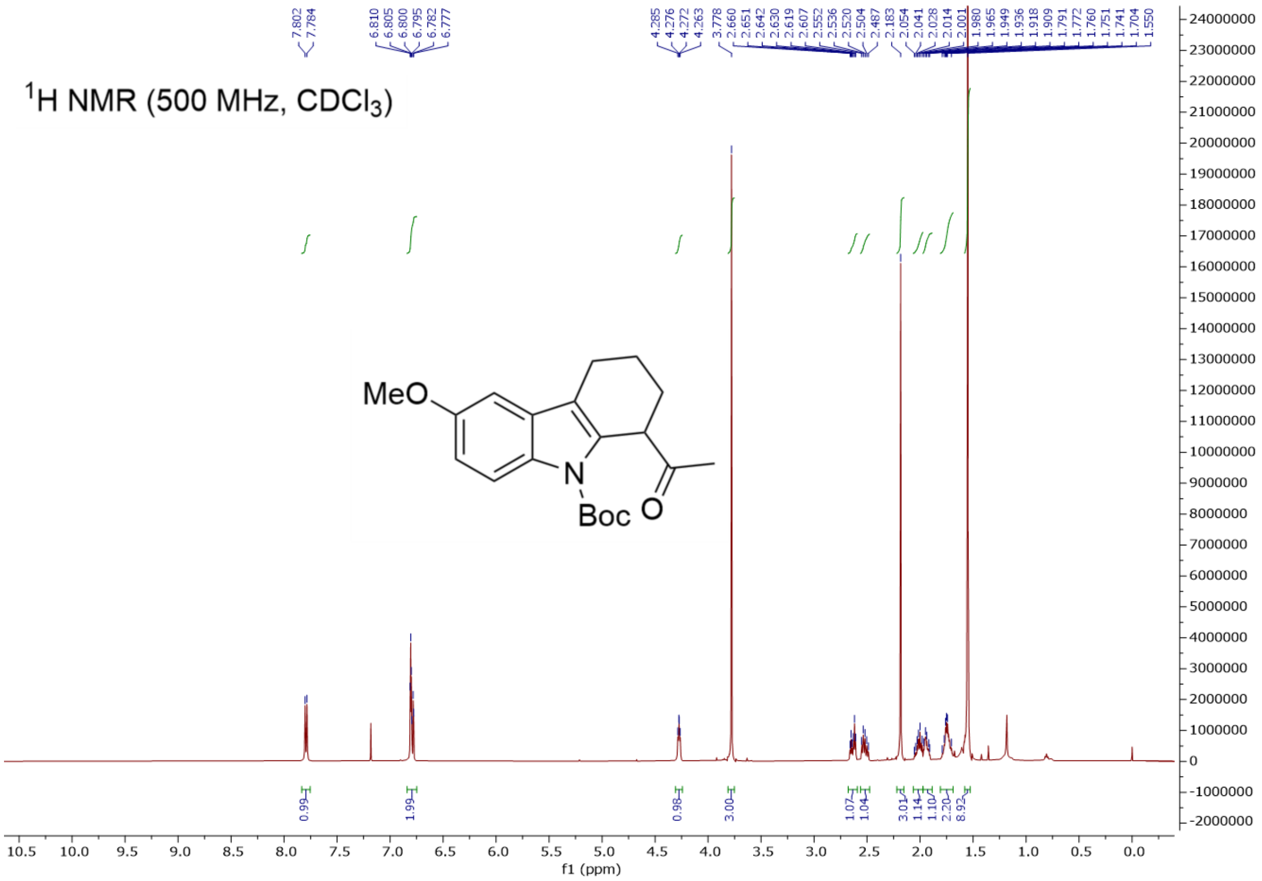
1H NMR (500 MHz, CDCl3)
MeO
N
Boc
O
f1 (ppm)

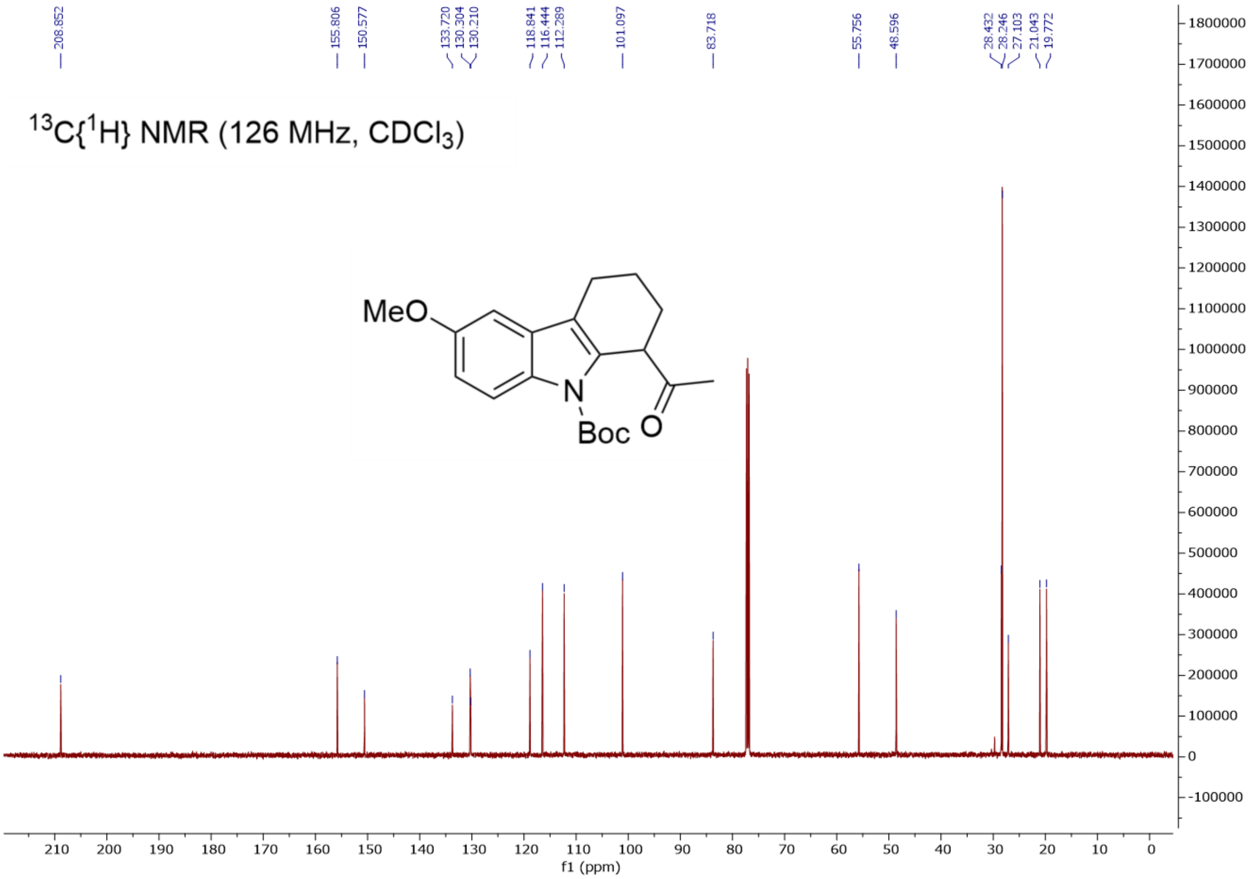
^{13}C{^{1}H} NMR (126 MHz, CDCl$_3$)
208.852
155.806
150.577
133.720
130.304
130.210
118.841
116.444
112.289
101.097
83.718
55.756
48.596
28.432
28.246
27.103
21.043
19.772
MeO
N
Boc
O
f1 (ppm)

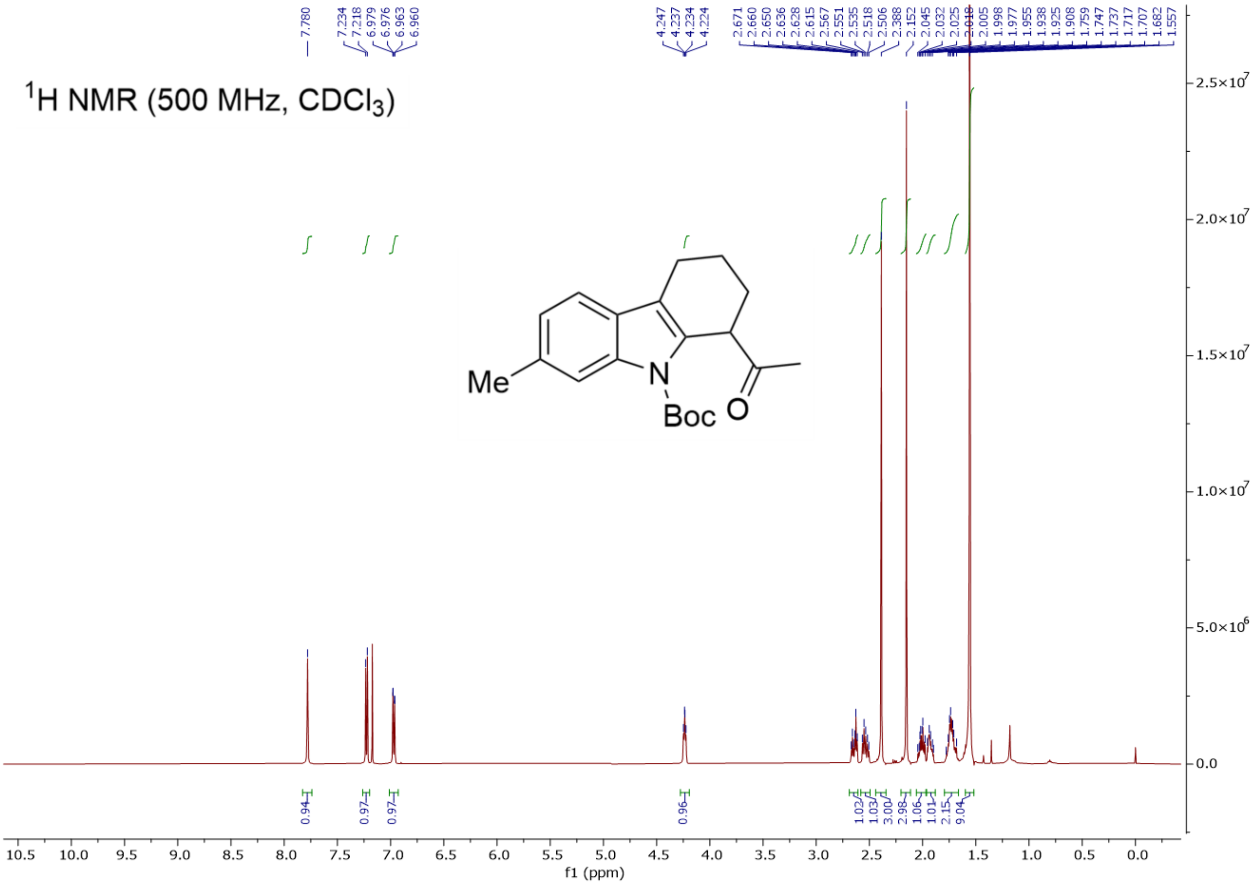
^{1}H NMR (500 MHz, CDCl$_3$)
7.780
7.234
7.218
6.979
6.976
6.963
6.960
4.247
4.237
4.234
4.224
Me
N
Boc
O
f1 (ppm)

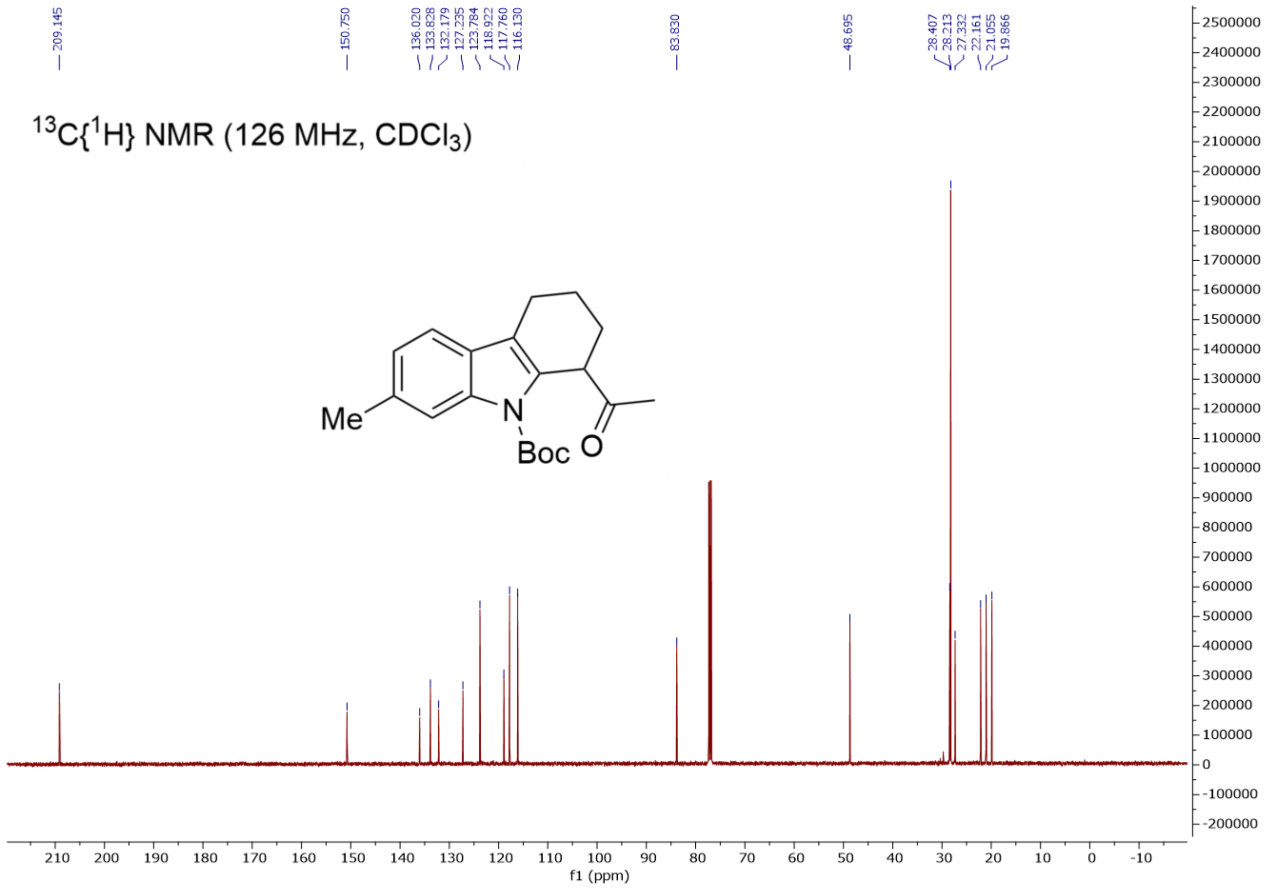
209.145
150.750
136.020
133.828
132.179
127.235
123.784
118.922
117.760
116.130
83.830
48.695
28.407
28.213
27.332
22.161
21.055
19.866
13C{1H} NMR (126 MHz, CDCl3)
Me
N
Boc
O
f1 (ppm)

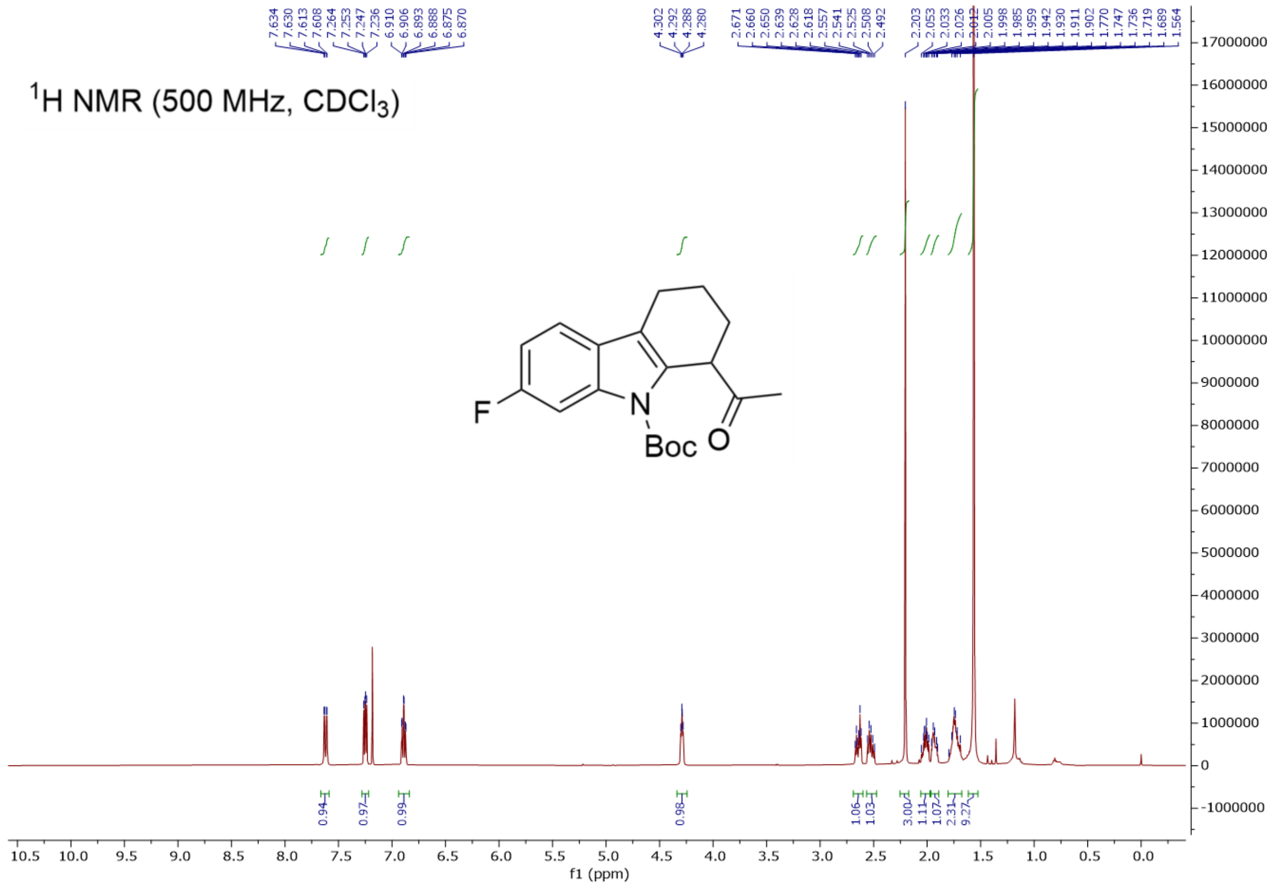
7.634
7.630
7.613
7.608
7.264
7.253
7.247
7.236
6.910
6.906
6.893
6.888
6.875
6.870
4.302
4.292
4.288
4.280
2.671
2.660
2.650
2.639
2.628
2.618
2.557
2.541
2.525
2.508
2.492
2.203
2.053
2.033
2.026
2.012
2.005
1.998
1.985
1.959
1.942
1.930
1.911
1.902
1.770
1.747
1.736
1.719
1.689
1.564
1H NMR (500 MHz, CDCl3)
F
N
Boc
O
0.94
0.97
0.99
0.98
1.06
1.03
3.00
1.11
1.07
2.31
9.27
f1 (ppm)

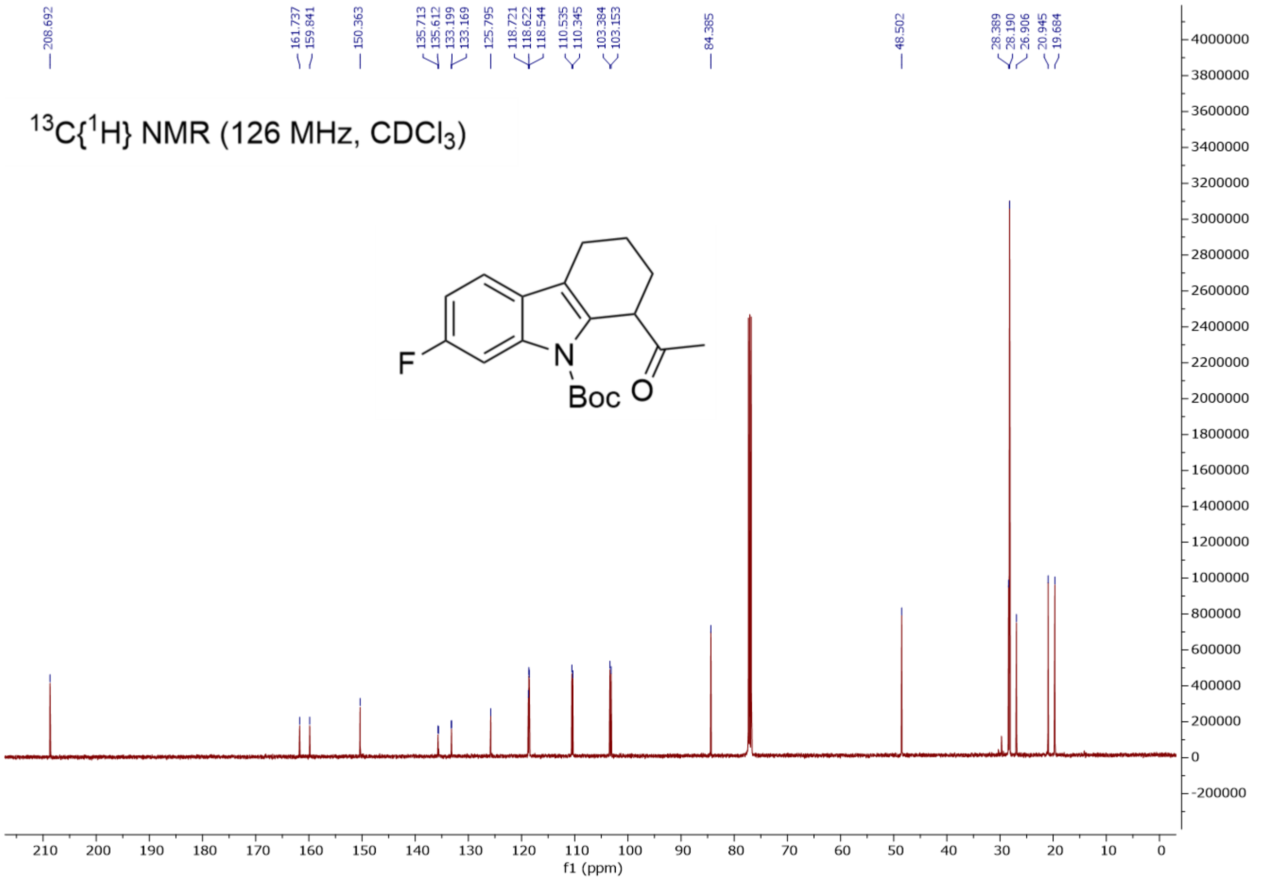
208.692
161.737
159.841
150.363
135.713
135.612
133.199
133.169
125.795
118.721
118.622
118.544
110.535
110.345
103.384
103.153
84.385
48.502
28.389
28.190
26.906
20.945
19.684
$^{13}C\{^1H\}$ NMR (126 MHz, $CDCl_3$)
F
N
Boc
O
f1 (ppm)

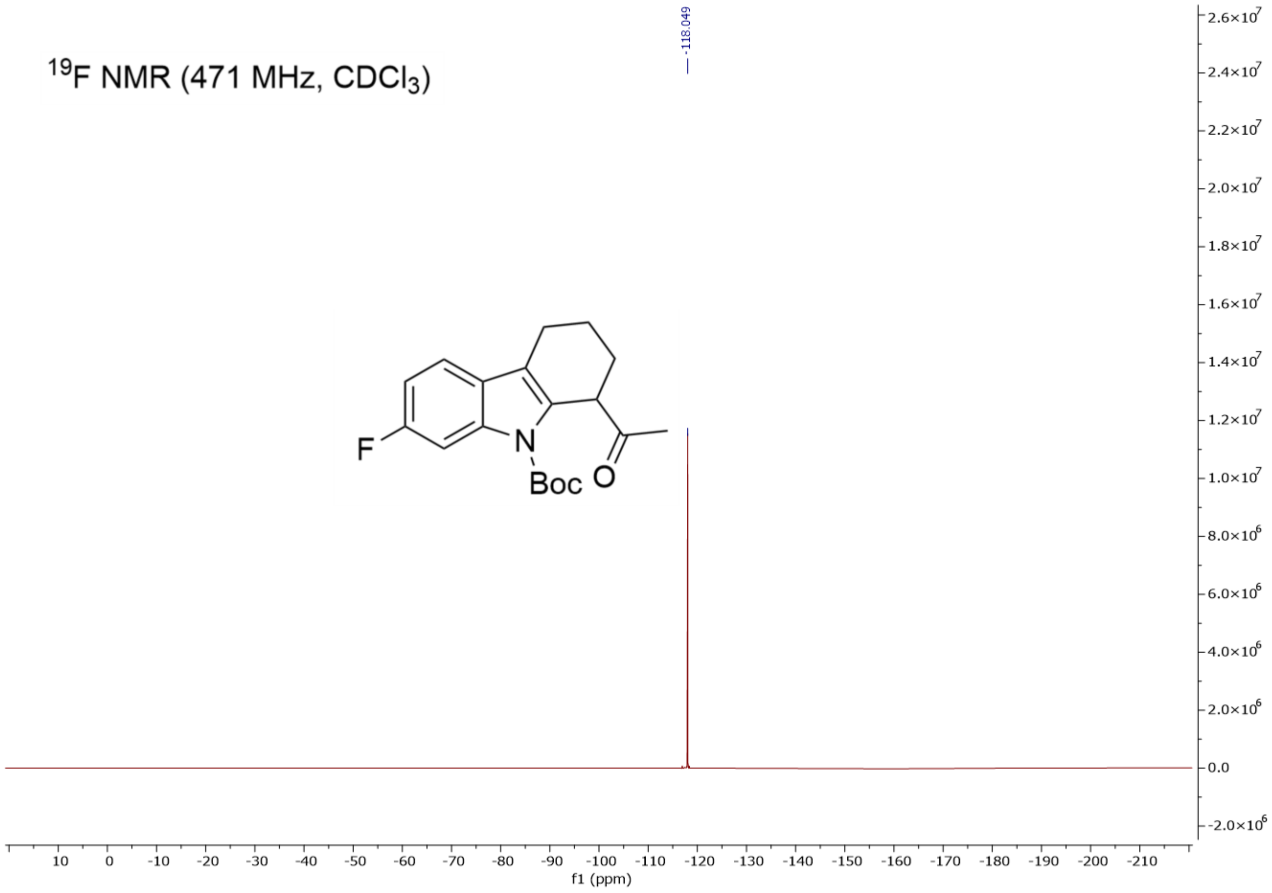
-118.049
^{19}F NMR (471 MHz, $CDCl_3$)
F
N
Boc
O
f1 (ppm)

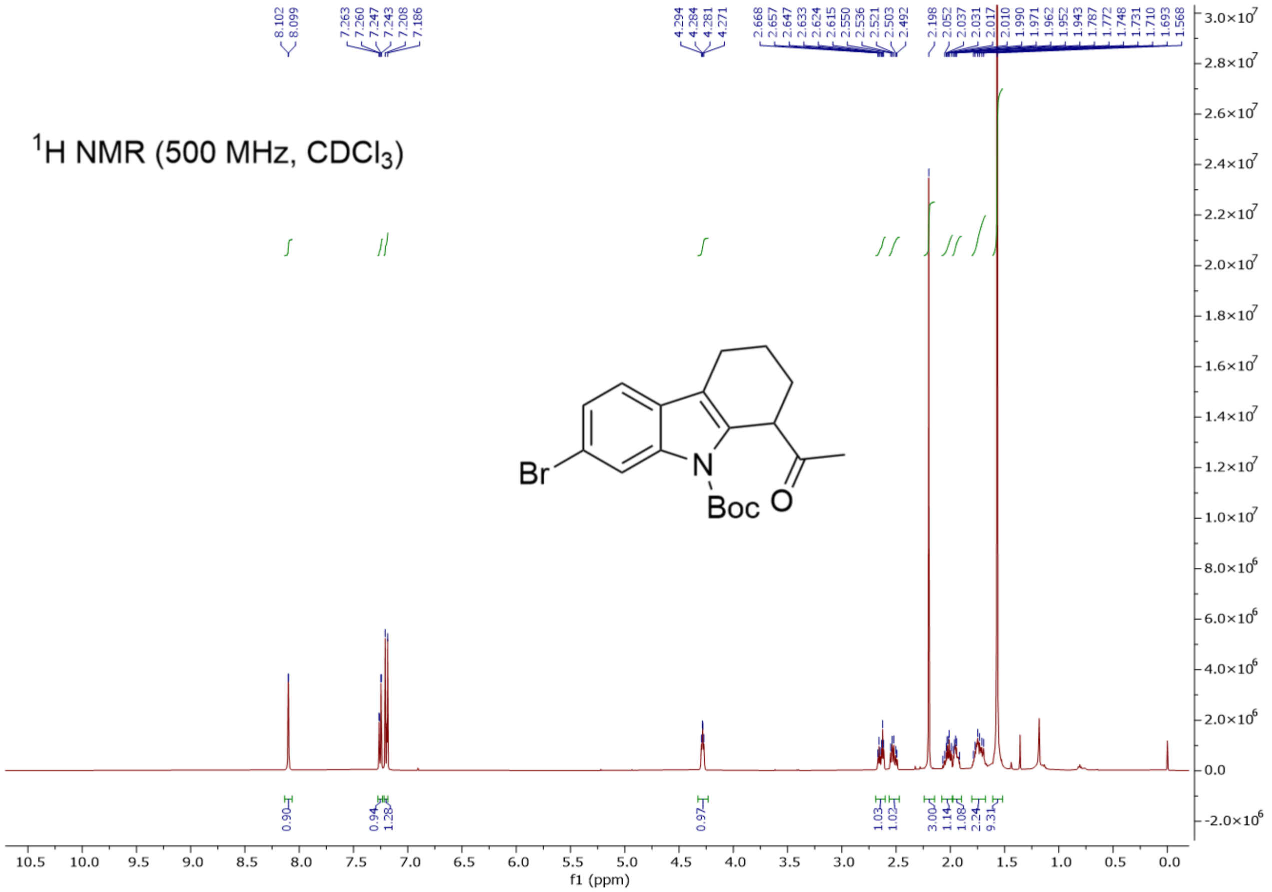

1H NMR (500 MHz, CDCl3)
Br
N
Boc
O
f1 (ppm)

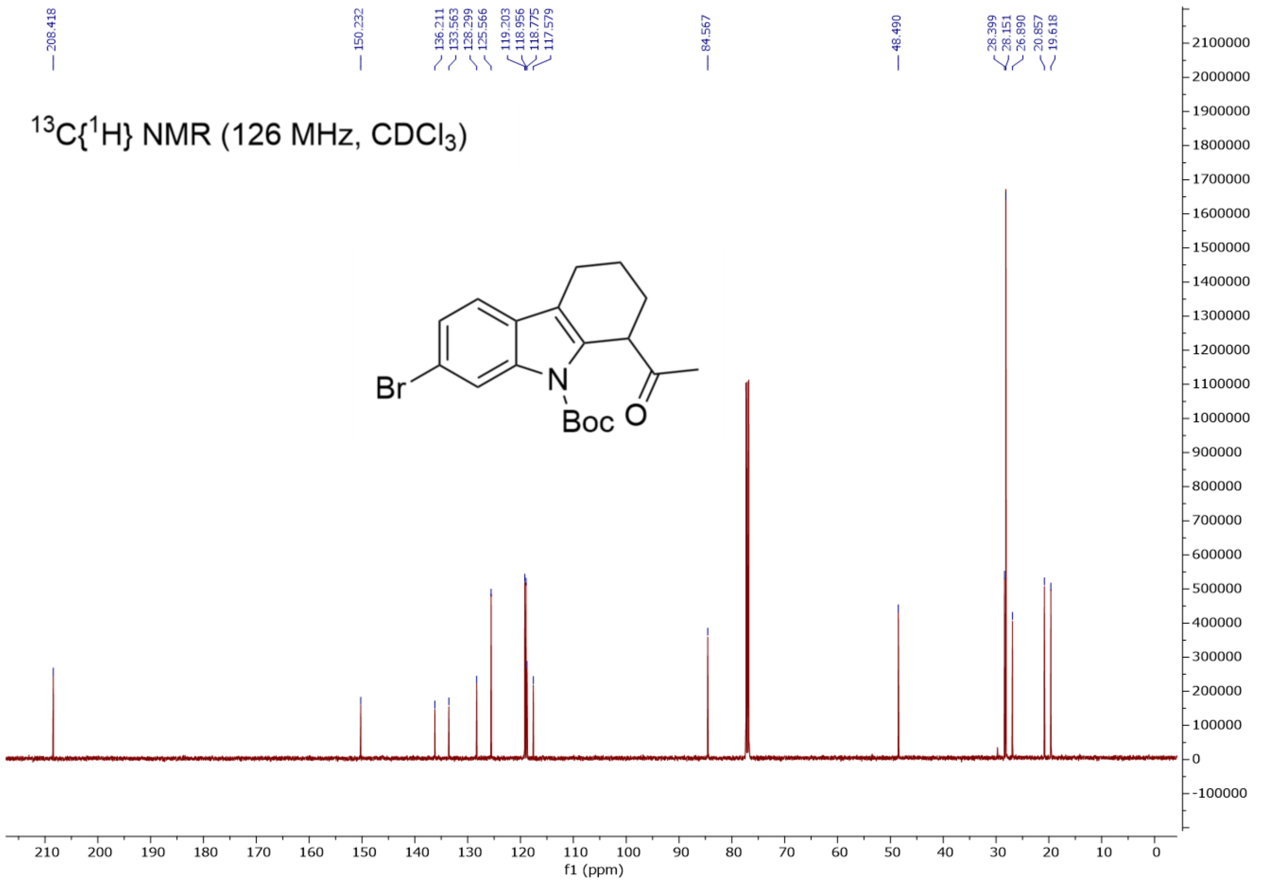

13C{1H} NMR (126 MHz, CDCl3)
Br
N
Boc
O
f1 (ppm)

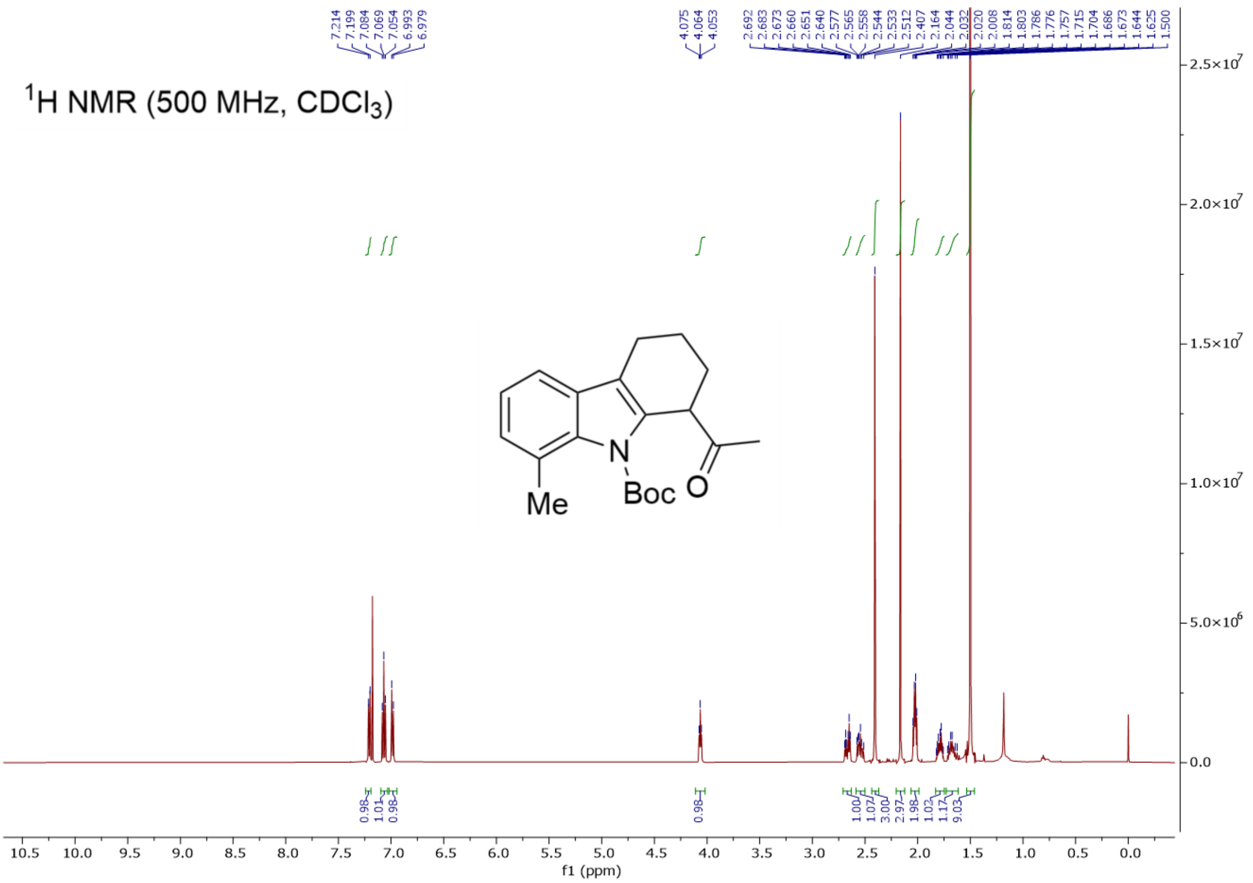

1H NMR (500 MHz, CDCl3)
7.214
7.199
7.084
7.069
7.054
6.993
6.979
4.075
4.064
4.053
2.692
2.683
2.673
2.660
2.651
2.640
2.577
2.565
2.558
2.544
2.533
2.512
2.407
2.164
2.044
2.032
2.020
2.008
1.814
1.803
1.786
1.776
1.757
1.715
1.704
1.686
1.673
1.644
1.625
1.500
Me
N
Boc
O
f1 (ppm)

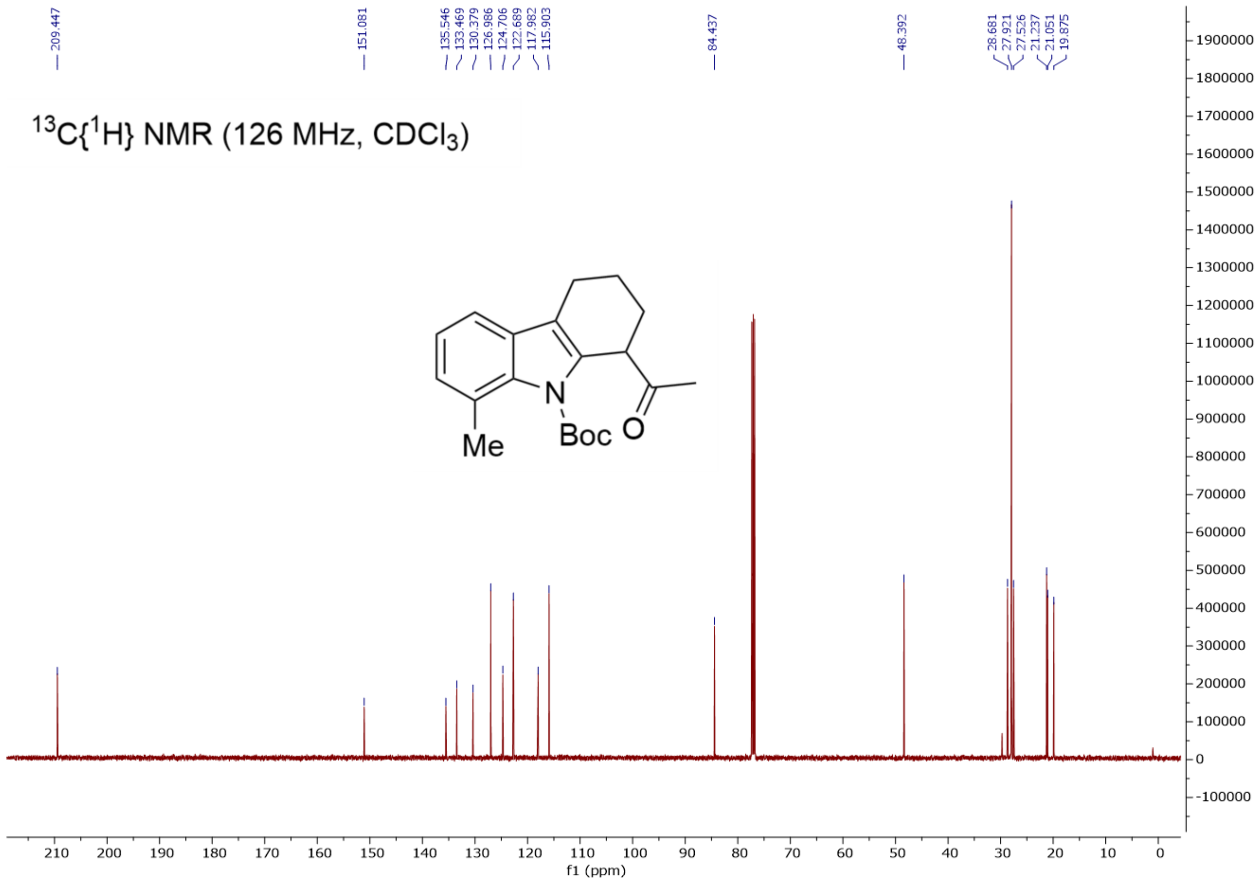

13C{1H} NMR (126 MHz, CDCl3)
209.447
151.081
135.546
133.469
130.379
126.986
124.706
122.689
117.982
115.903
84.437
48.392
28.681
27.921
27.526
21.237
21.051
19.875
Me
N
Boc
O
f1 (ppm)

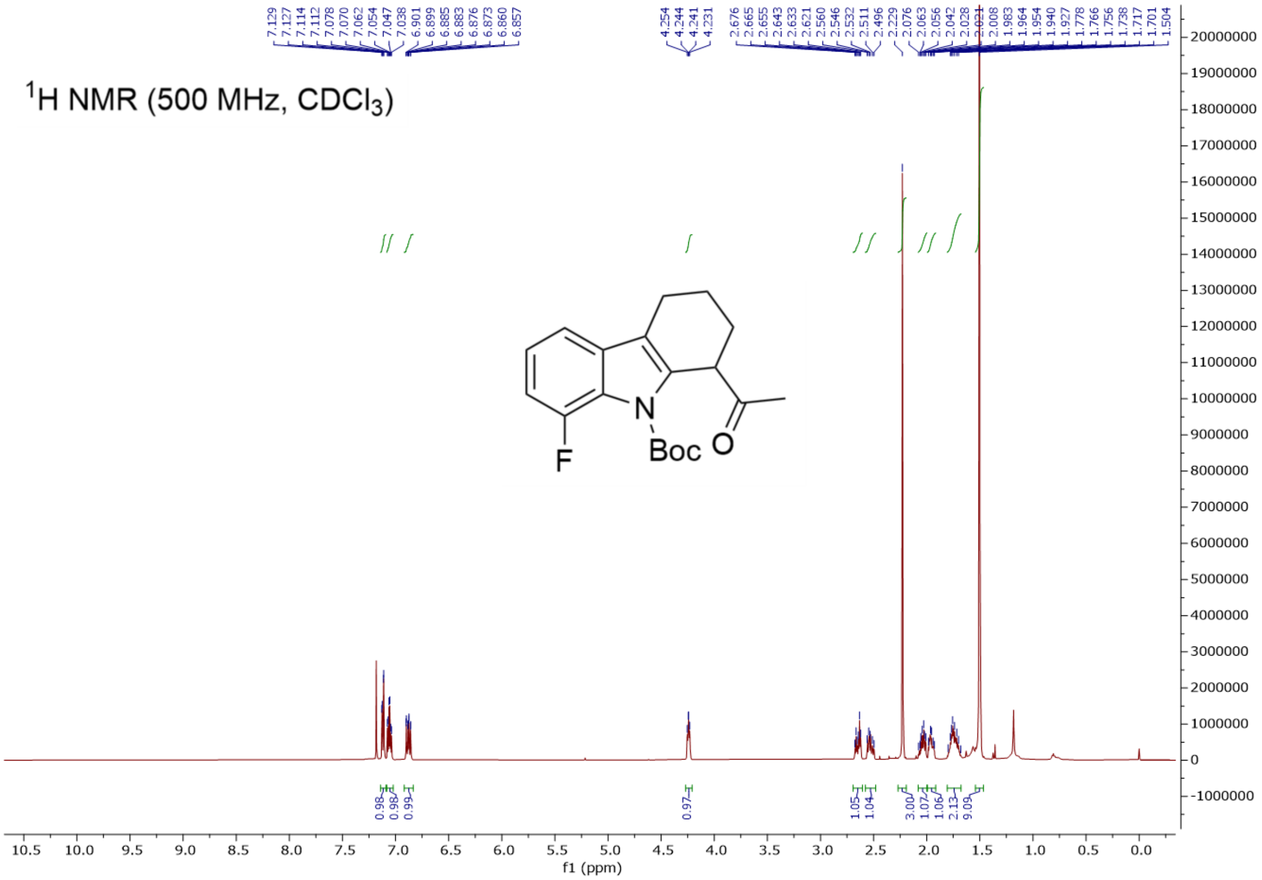

^{1}H NMR (500 MHz, $CDCl_3$)
F
N
Boc
O

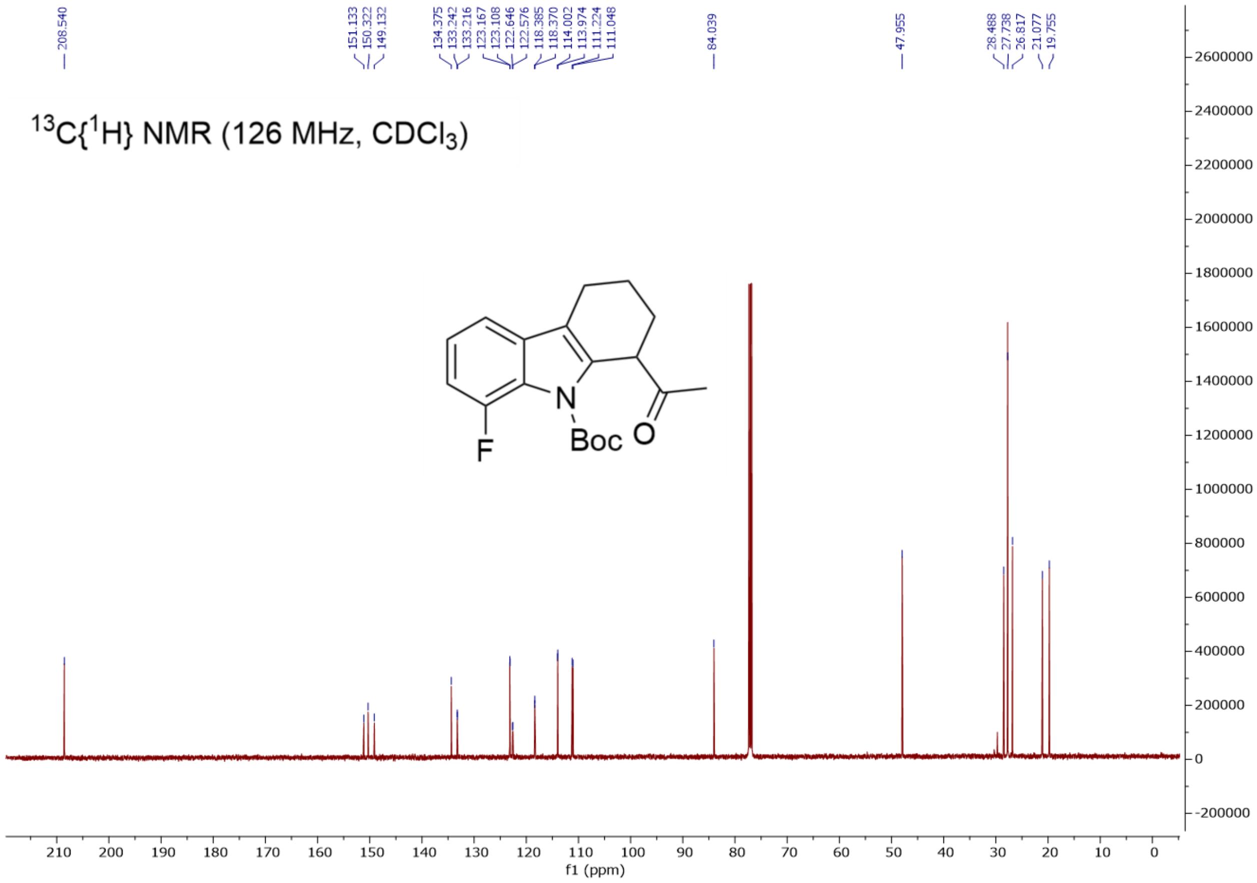

$^{13}C\{^{1}H\}$ NMR (126 MHz, $CDCl_3$)
F
N
Boc
O

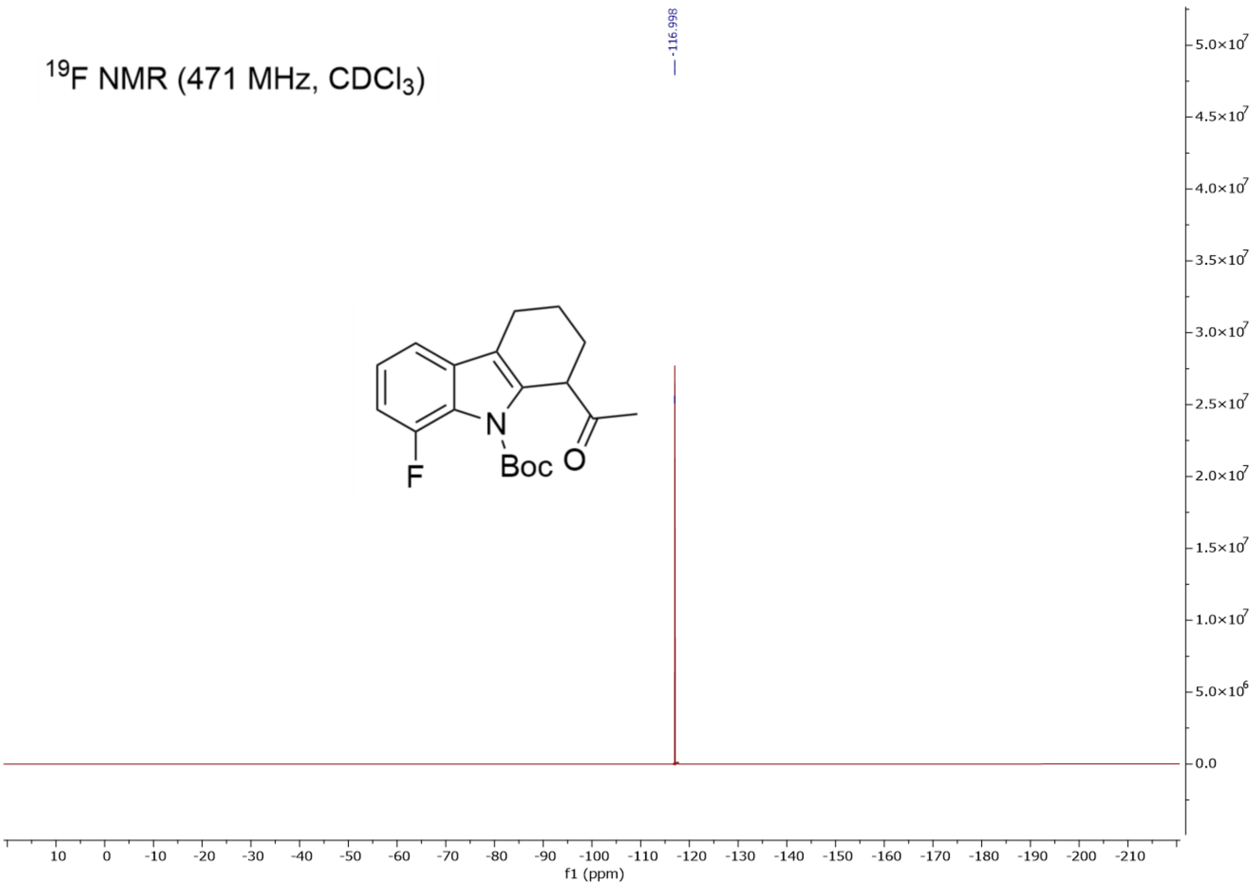

19F NMR (471 MHz, CDCl3)
-116.998
F
N
Boc
O
f1 (ppm)

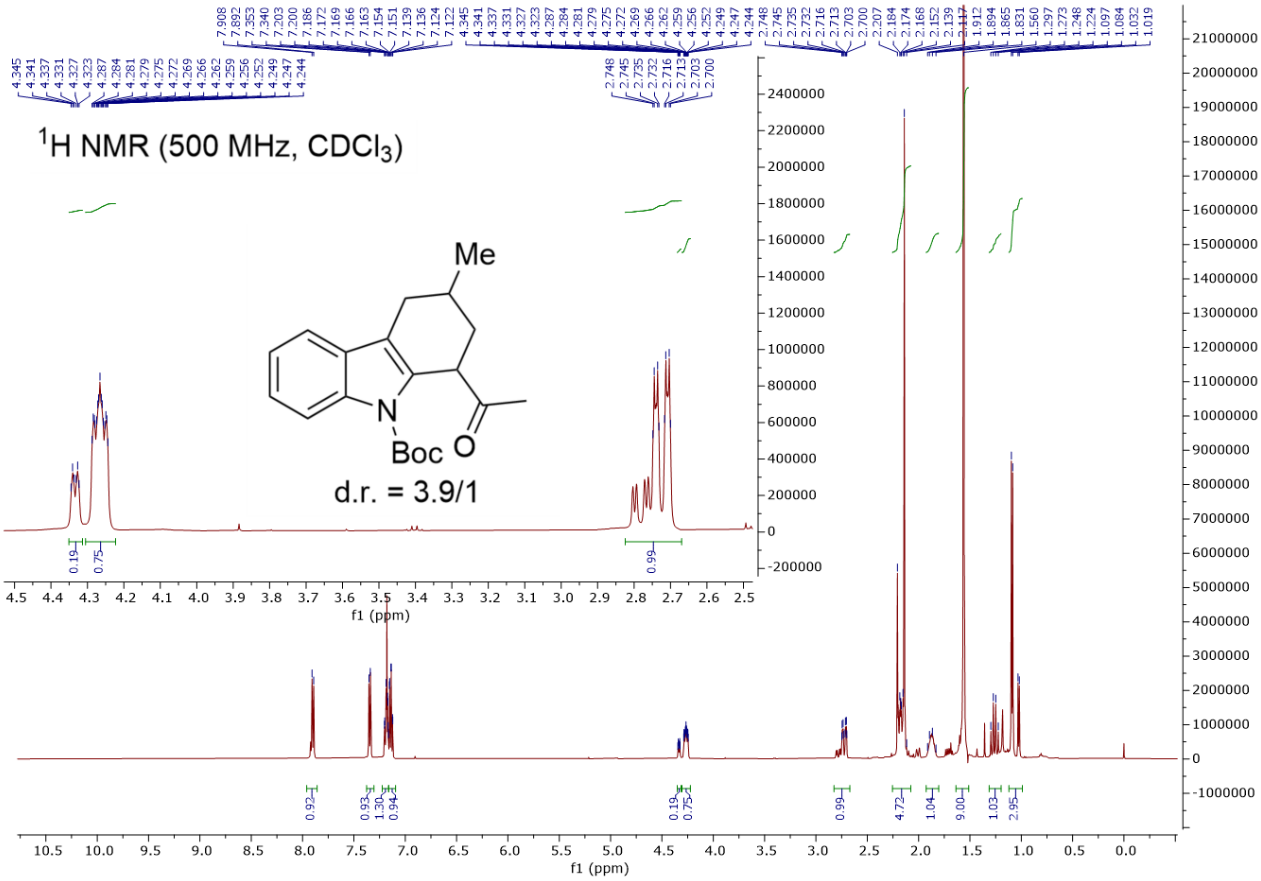

1H NMR (500 MHz, CDCl3)
Me
N
Boc
O
d.r. = 3.9/1
f1 (ppm)

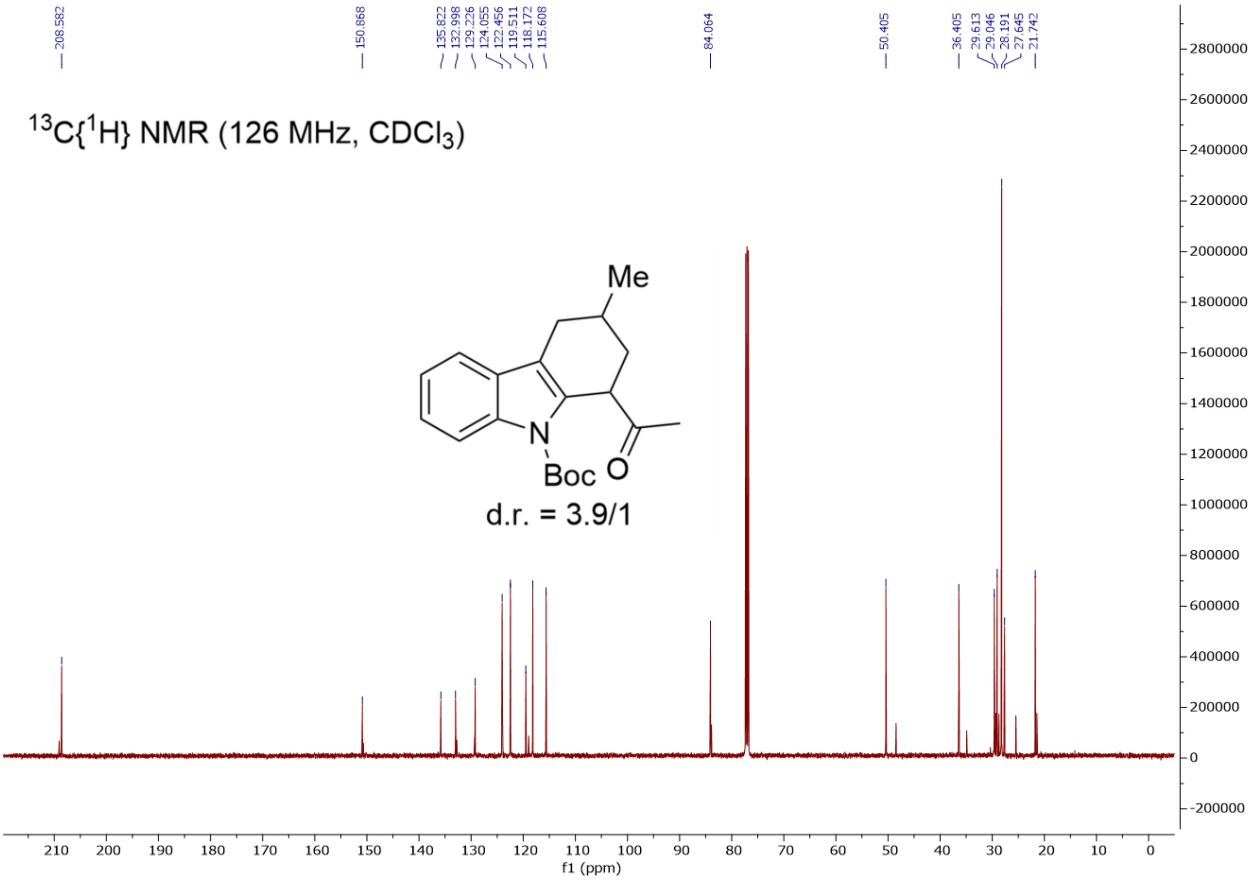

208.582
150.868
135.822
132.998
129.226
124.055
122.456
119.511
118.172
115.608
84.064
50.405
36.405
29.613
29.046
28.191
27.645
21.742
$^{13}C\{^1H\}$ NMR (126 MHz, $CDCl_3$)
Me
N
Boc
O
d.r. = 3.9/1
f1 (ppm)

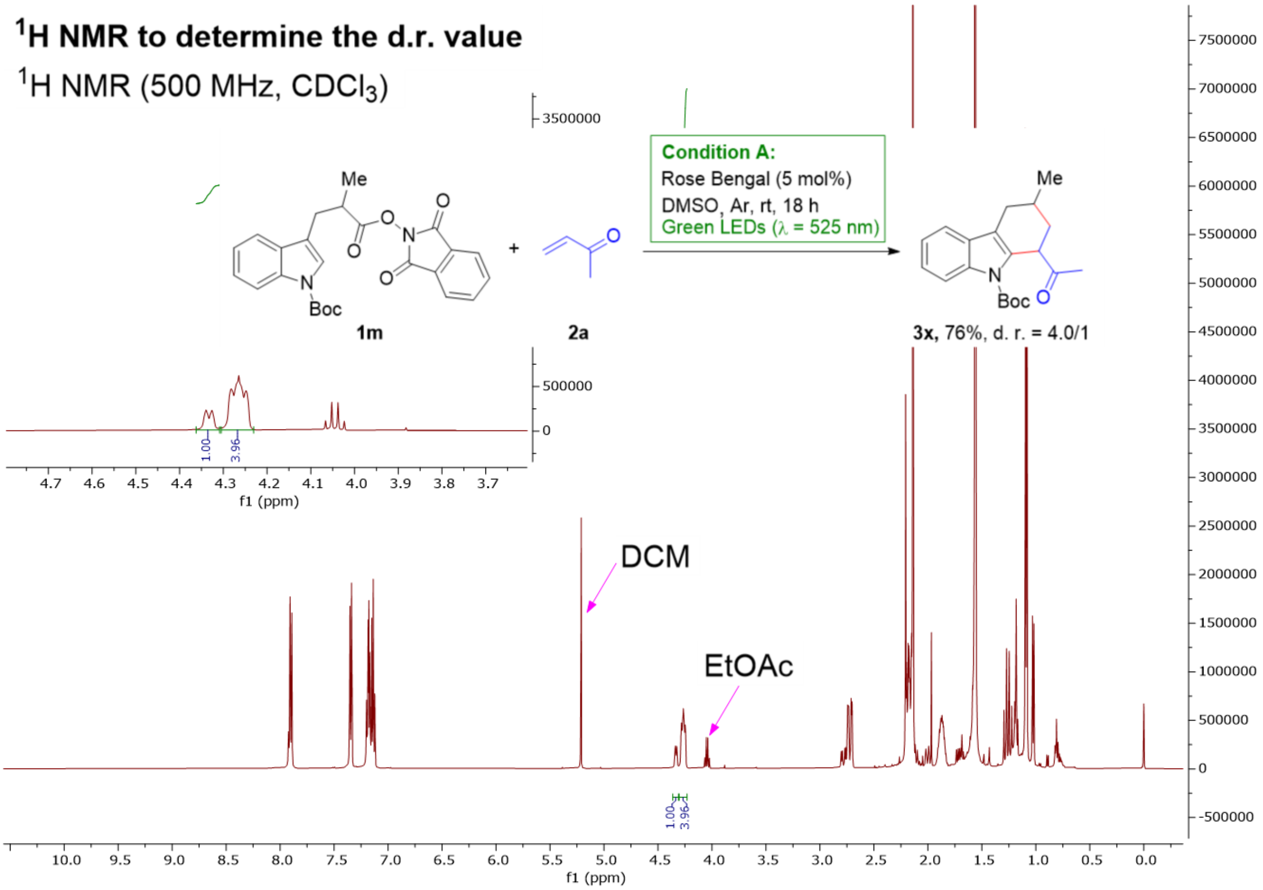

^{1}H NMR to determine the d.r. value
^{1}H NMR (500 MHz, $CDCl_3$)
Condition A:
Rose Bengal (5 mol%)
DMSO, Ar, rt, 18 h
Green LEDs (λ = 525 nm)
1m
2a
3x, 76%, d. r. = 4.0/1
1.00
3.96
DCM
EtOAc
f1 (ppm)

^{1}H NMR to determine the d.r. value

^{1}H NMR (500 MHz, $CDCl_3$)

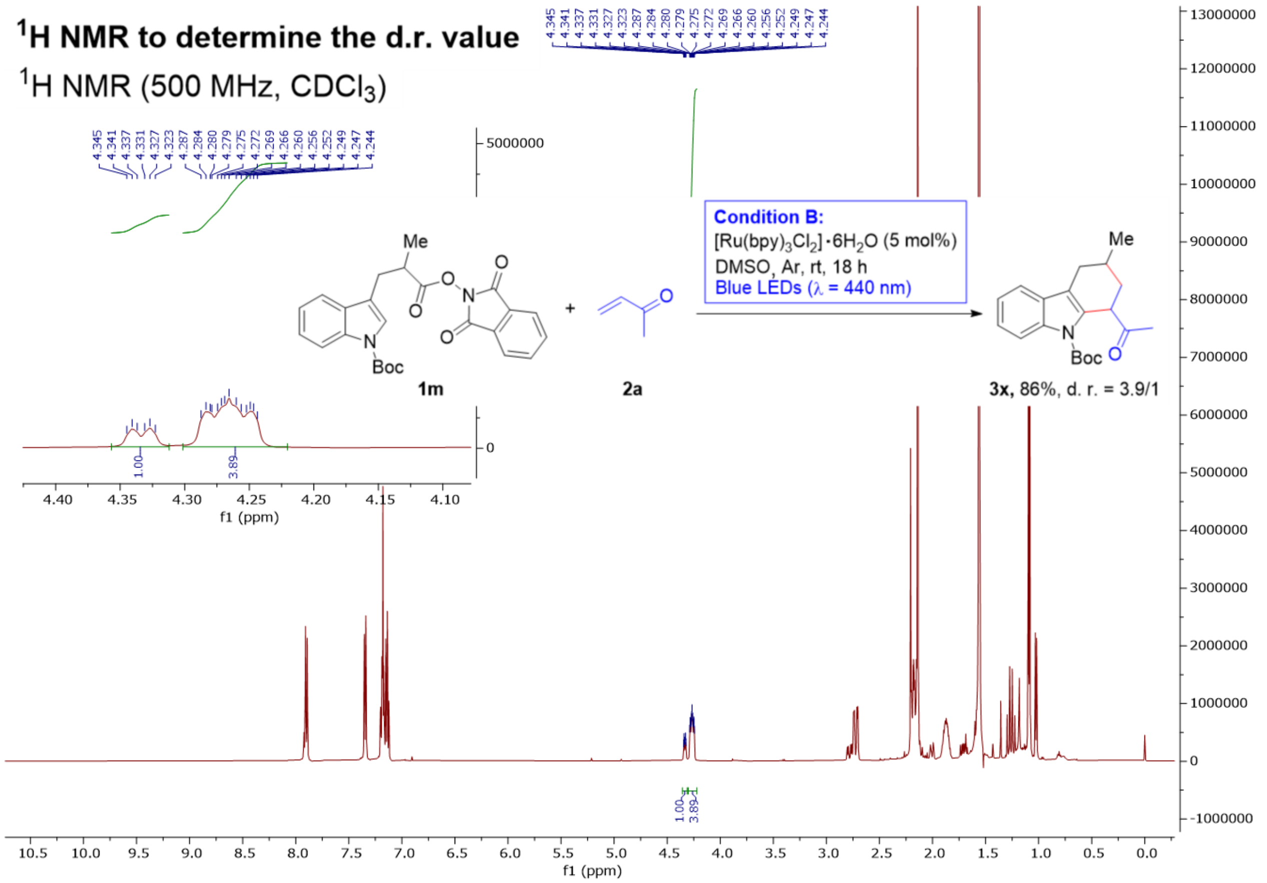

^{1}H NMR (500 MHz, $CDCl_3$)

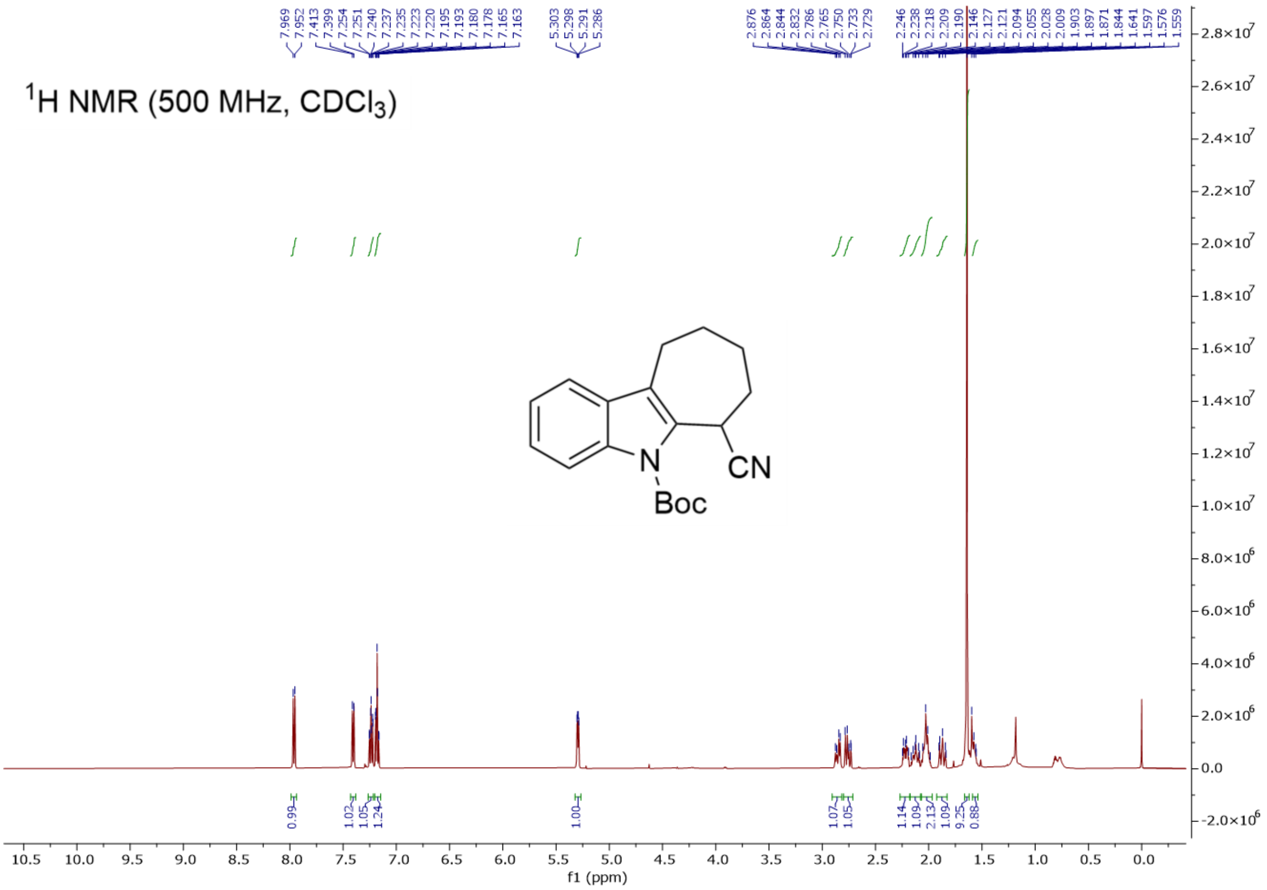

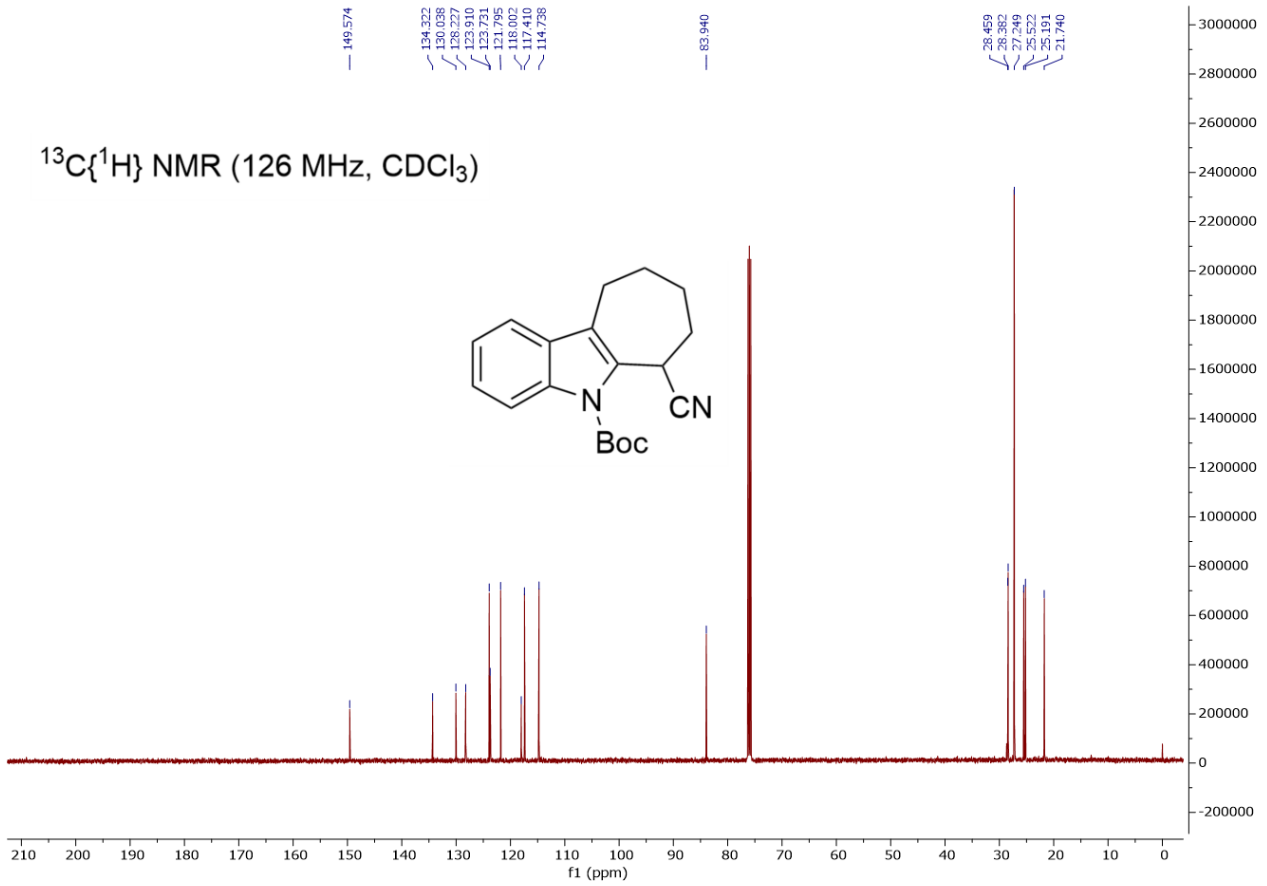

$^{13}C\{^1H\}$ NMR (126 MHz, $CDCl_3$)
N
Boc
CN
f1 (ppm)

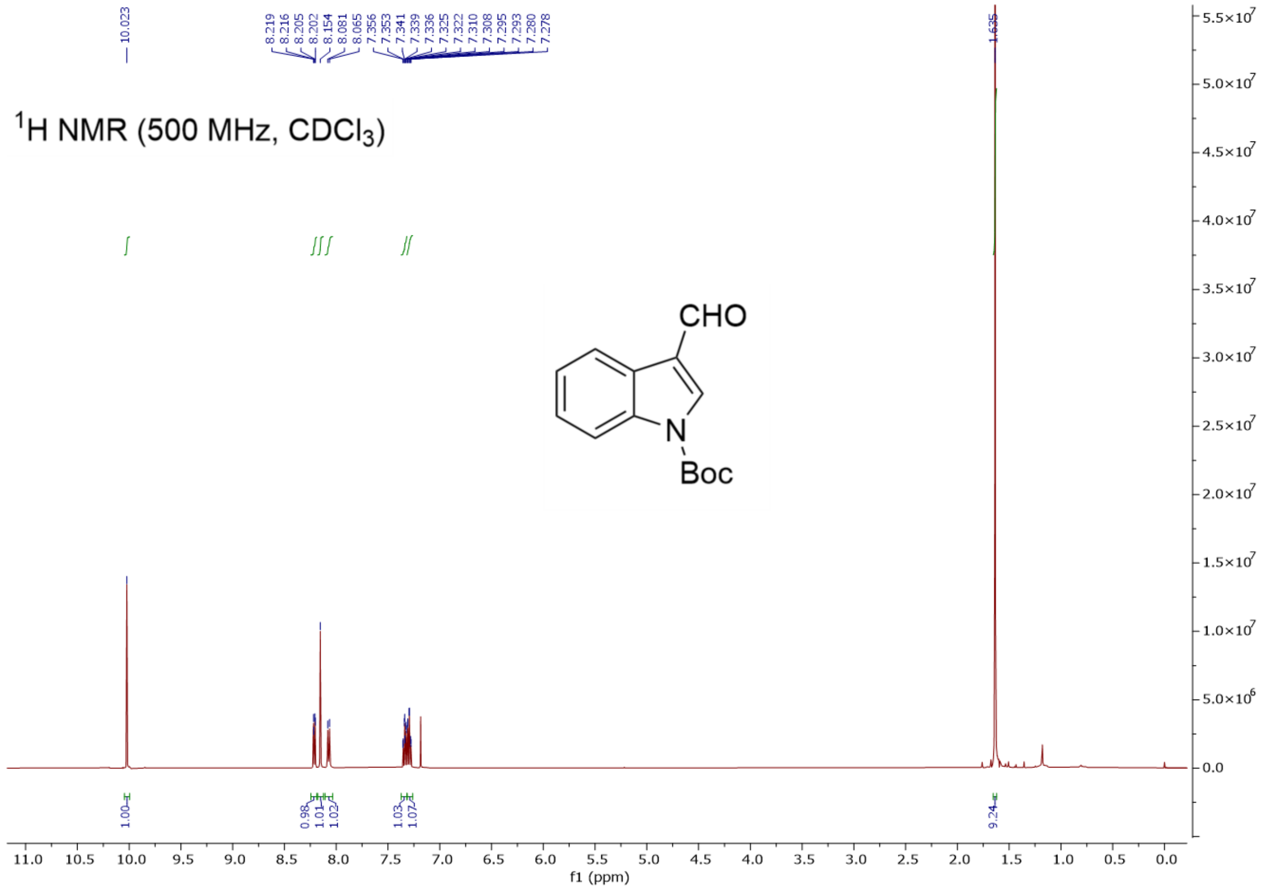

^1H NMR (500 MHz, $CDCl_3$)
CHO
N
Boc
f1 (ppm)

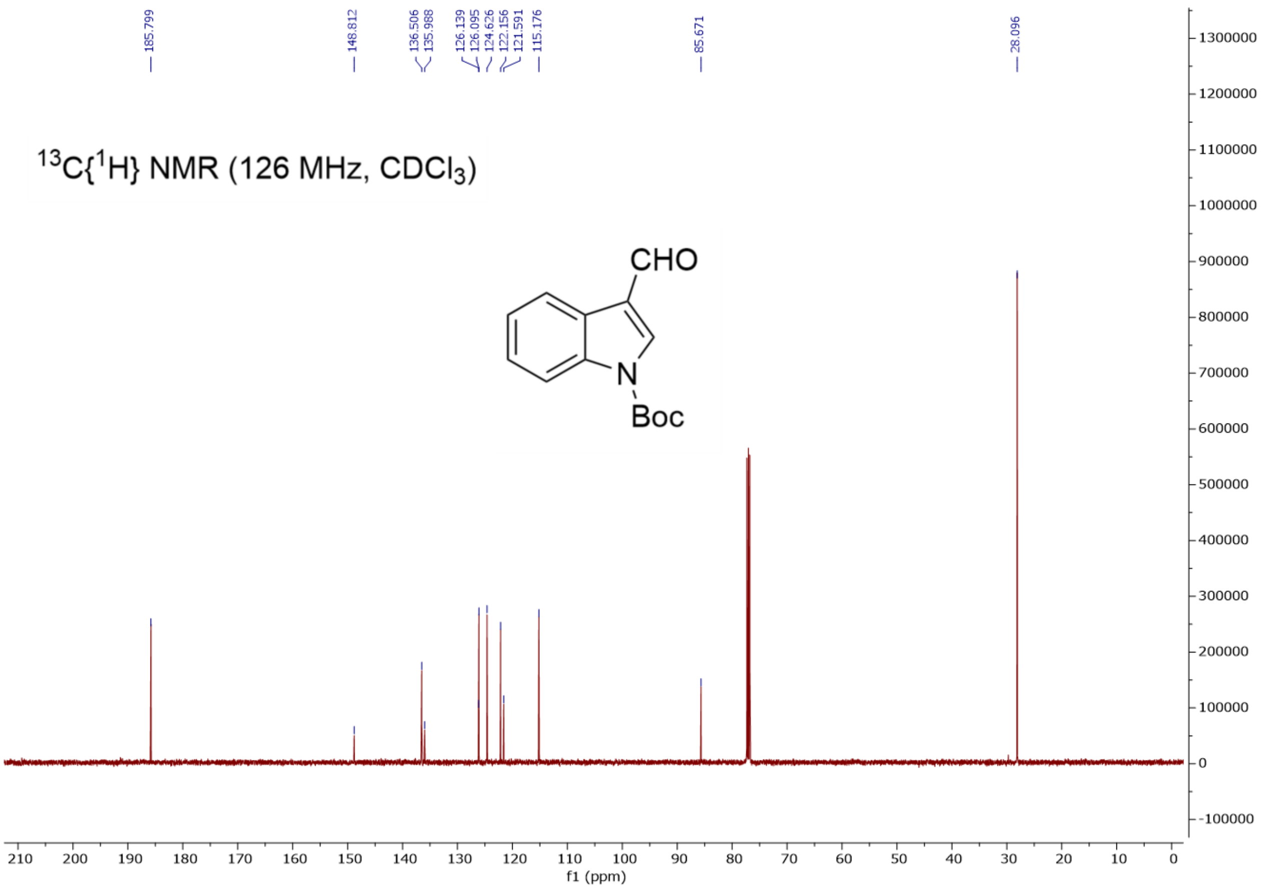

185.799
148.812
136.506
135.988
126.139
126.095
124.626
122.156
121.591
115.176
85.671
28.096
$^{13}C\{^1H\}$ NMR (126 MHz, $CDCl_3$)
CHO
N
Boc
f1 (ppm)

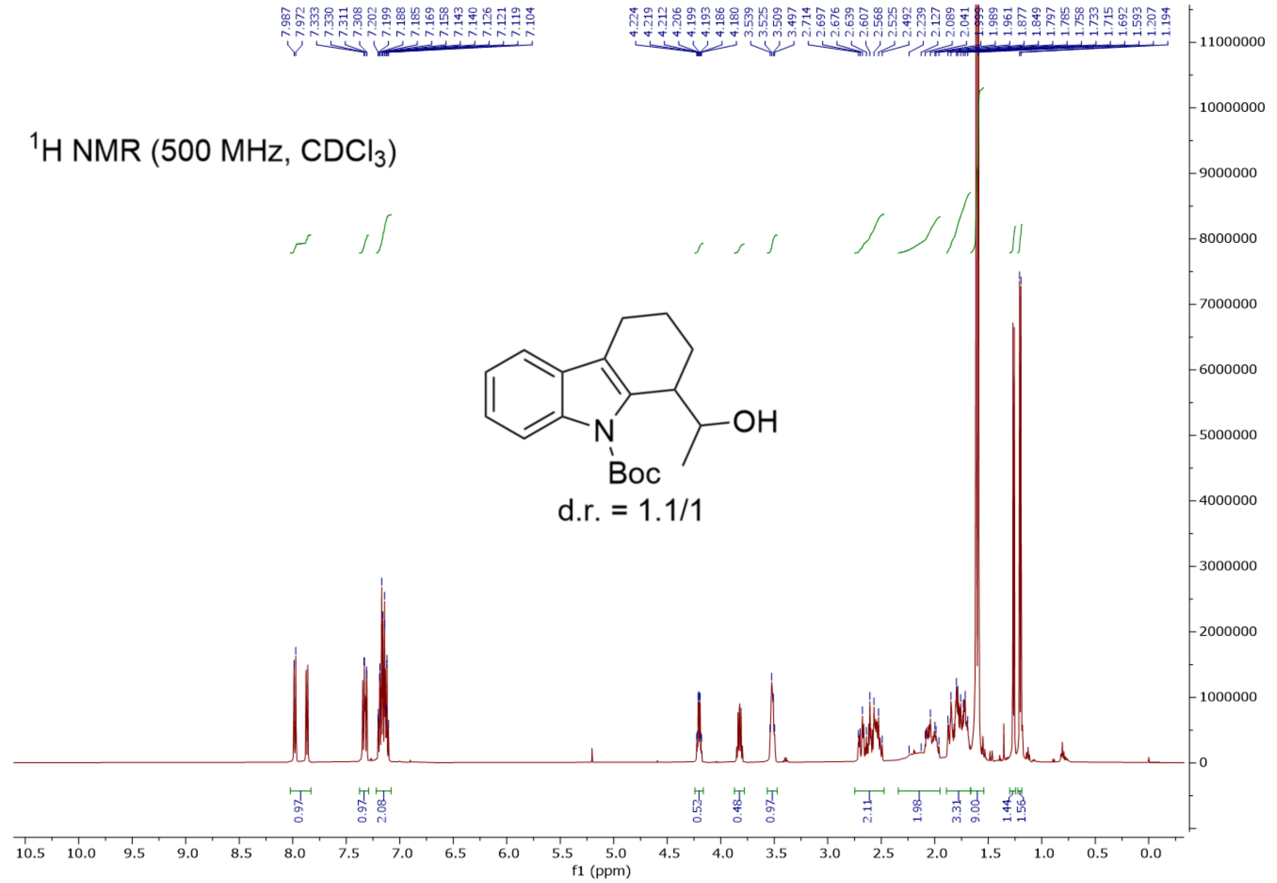

^1H NMR (500 MHz, $CDCl_3$)
OH
N
Boc
d.r. = 1.1/1
f1 (ppm)

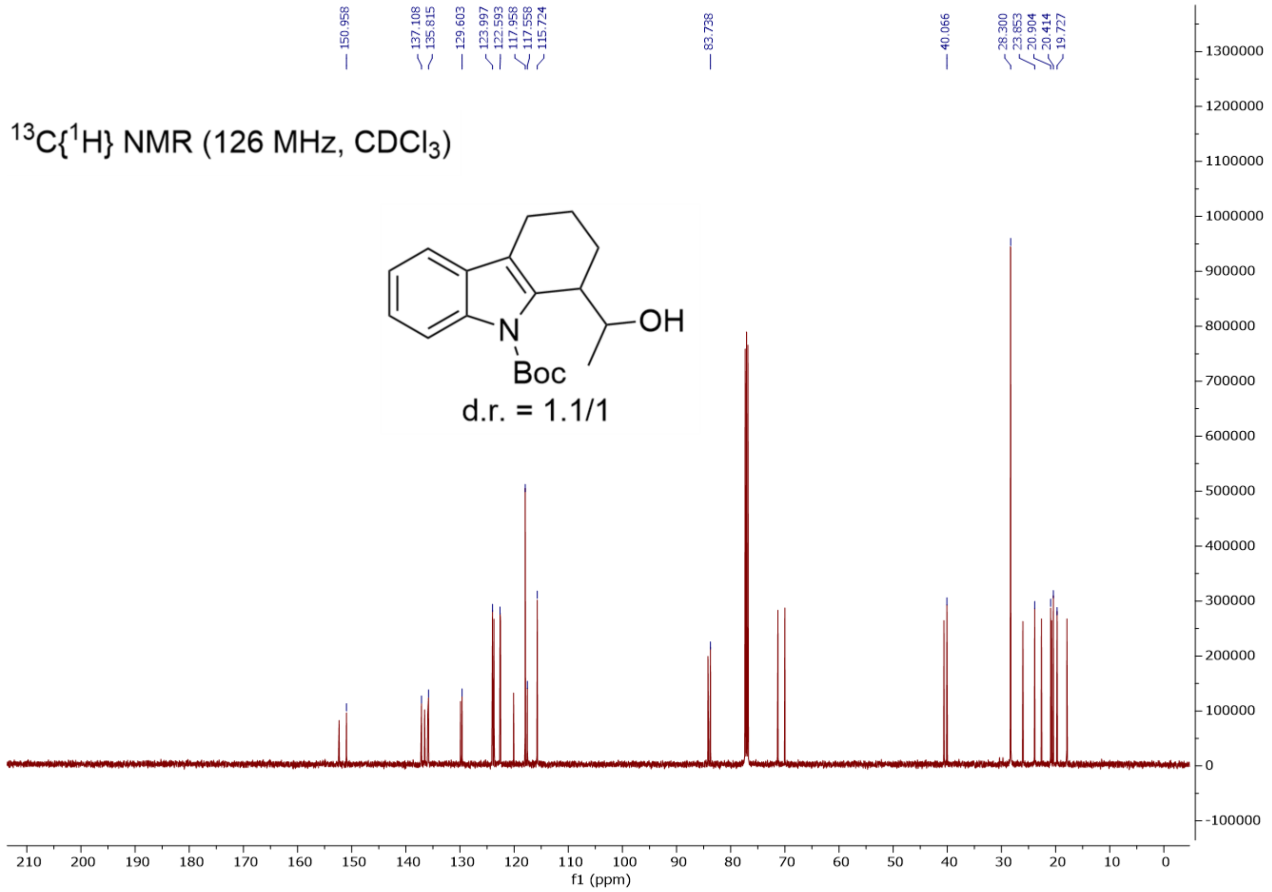

150.958
137.108
135.815
129.603
123.997
122.593
117.958
117.558
115.724
83.738
40.066
28.300
23.853
20.904
20.414
19.727
$^{13}C\{^1H\}$ NMR (126 MHz, $CDCl_3$)
OH
N
Boc
d.r. = 1.1/1
f1 (ppm)

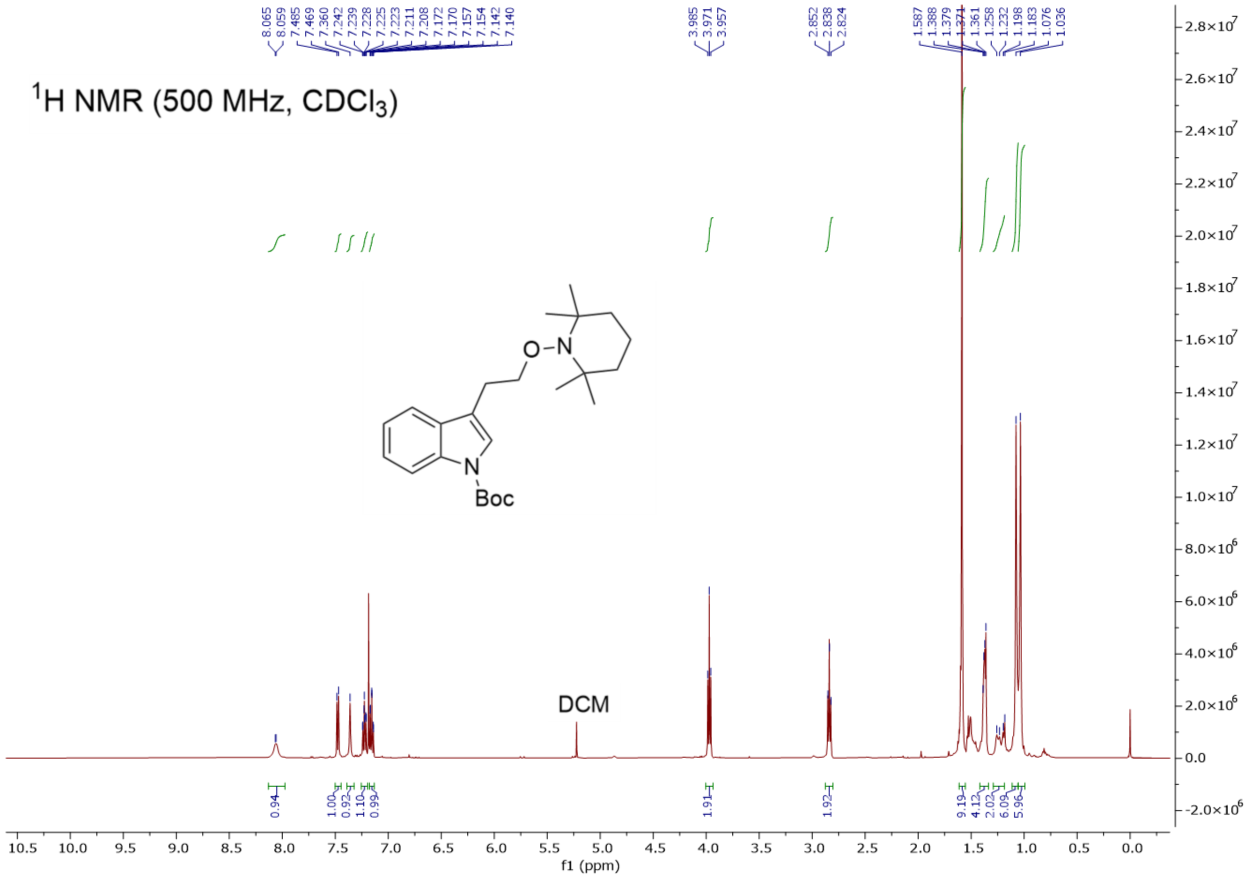

8.065
8.059
7.485
7.469
7.360
7.242
7.239
7.228
7.225
7.223
7.211
7.208
7.172
7.170
7.157
7.154
7.142
7.140
3.985
3.971
3.957
2.852
2.838
2.824
1.587
1.388
1.379
1.371
1.361
1.358
1.232
1.198
1.183
1.076
1.036
^1H NMR (500 MHz, $CDCl_3$)
O
N
N
Boc
DCM
0.94
1.00
0.92
1.10
0.99
1.91
1.92
9.19
4.13
2.02
6.09
5.96
f1 (ppm)

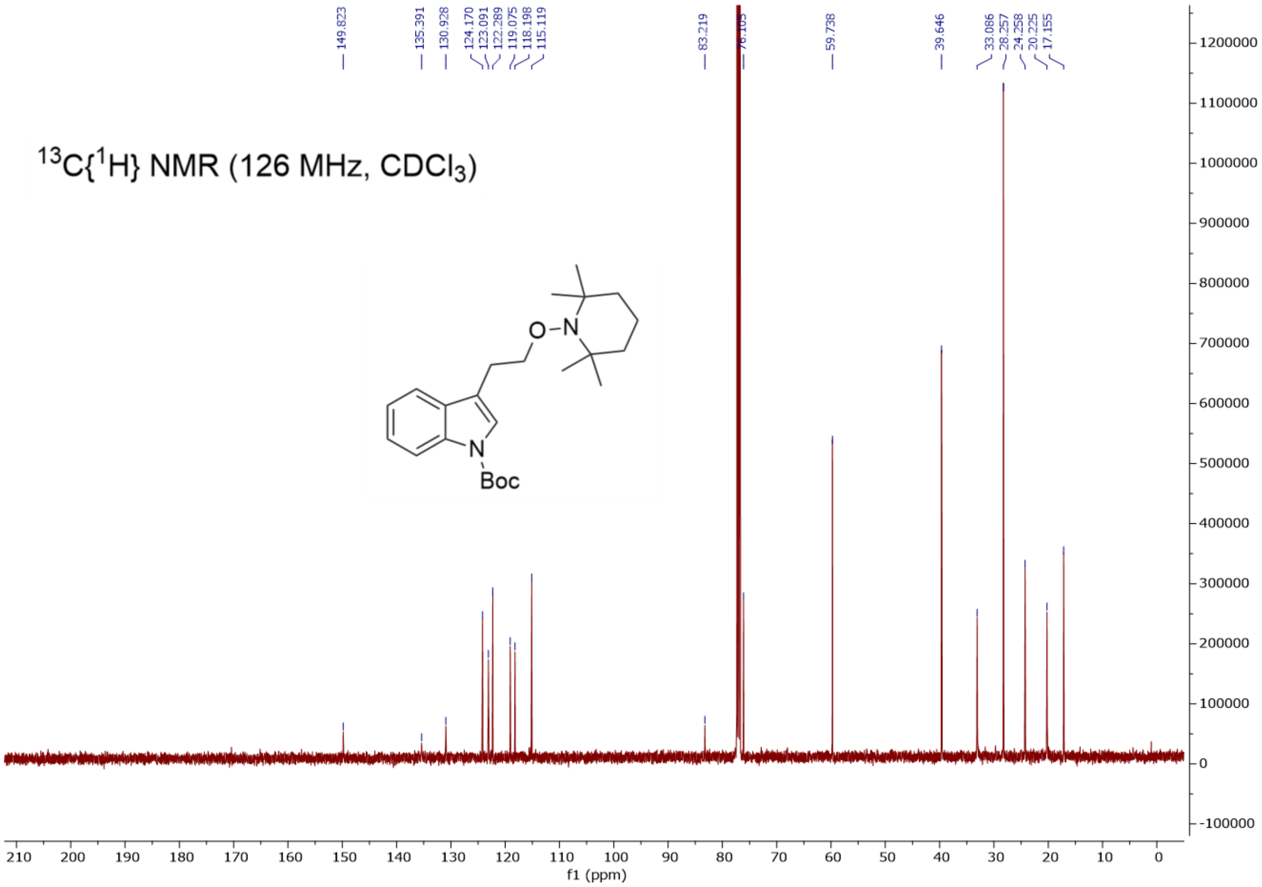

$^{13}C\{^{1}H\}$ NMR (126 MHz, $CDCl_3$)
149.823
135.391
130.928
124.170
123.091
122.289
119.075
118.198
115.119
83.219
59.738
39.646
33.086
28.257
24.258
20.225
17.155
O
N
N
Boc
f1 (ppm)

7. References

(1) Mei, L.; Moutet, J.; Stull, S. M.; Gianetti, T. L. Synthesis of CF_3-Containing Spirocyclic Indolines via a Red-Light-Mediated Trifluoromethylation/Dearomatization Cascade. *J. Org. Chem.* **2021**, *86*, 10640−10653.

(2) Okanishi, Y.; Takemoto, O.; Kawahara, S.; Hayashi, S.; Takanami, T.; Yoshimitsu, T. Red-Light-Promoted Radical Cascade Reaction to Access Tetralins and Dialins Enabled by Zinc(II)porphyrin, A Light-Flexible Catalyst. *Org. Lett.* **2024**, *26*, 3929−3934.

(3) Prandi, C.; Occhiato, E. G.; Tabasso, S.; Bonfante, P.; Novero, M.; Scarpi, D.; Bova, M. E.; Miletto, I. New Potent Fluorescent Analogues of Strigolactones: Synthesis and Biological Activity in Parasitic Weed Germination and Fungal Branching. *Eur. J. Org. Chem.* **2011**, 3781−3793.

(4) Shiina, I.; Umezaki, Y.; Kuroda, N.; Iizumi, T.; Nagai, S.; Katoh, T. MNBA-Mediated β-Lactone Formation: Mechanistic Studies and Application for the Asymmetric Total Synthesis of Tetrahydrolipstatin. *J. Org. Chem.* **2012**, *77*, 4885−4901.

(5) Bock, C. R.; Connor, J. A.; Gutierrez, A. R.; Meyer, T. J.; Whitten, D. G.; Sullivan, B. P.; Nagle, J. K. Estimation of excited-state redox potentials by electron-transfer quenching. Application of electron-transfer theory to excited-state redox processes. *J. Am. Chem. Soc.* **1979**, *101*, 4815–4824.

(6) (a) Okada, K.; Okamoto, K.; Oda, M. A new and practical method of decarboxylation: photosensitized decarboxylation of N-acyloxyphthalimides via electron-transfer mechanism. *J. Am. Chem. Soc.* **1988**, *110*, 8736–8738. (b) Pratsch, G.; Lackner, G. L.; Overman, L. E. Constructing Quaternary Carbons from N -(Acyloxy)Phthalimide Precursors of Tertiary Radicals Using Visible-Light Photocatalysis. *J. Org. Chem.* **2015**, *80*, 6025–6036. (c) Lackner, G. L.; Quasdorf, K. W.; Pratsch, G.; Overman, L. E. Fragment Coupling and the Construction of Quaternary Carbons Using Tertiary Radicals Generated from Tert -Alkyl N -Phthalimidoyl Oxalates by Visible-Light Photocatalysis. *J. Org. Chem.* **2015**, *80*, 6012–6024. (d) Zhang, J. J.; Yang, J. C.; Guo, L. N.; Duan, X. H. Visible-Light-Mediated Dual Decarboxylative Coupling of Redox-Active Esters with α,β-Unsaturated Carboxylic Acids. *Chem. - Eur. J.* **2017**, *23*, 10259–10263. (e) Han, S.; Chen, Z.; Guo, Y.; Chen, J.; Wang, Z.; Zeng, Y. F. Access to Tetrahydrocarbazoles via a Photocatalyzed Cascade Decarboxylation/Addition/Cyclization Reaction. *Org. Chem. Front.* **2024**, *11*, 6694–6699.
